# Synthesis of Enantiopure [3]Cyclorubicenes

**DOI:** 10.1002/anie.202520880

**Published:** 2025-11-10

**Authors:** Moritz P. Schuldt, Frank Rominger, Michael Mastalerz

**Affiliations:** ^1^ Organisch‐Chemisches Institut Ruprecht‐Karls‐Universität Heidelberg Im Neuenheimer Feld Heidelberg 270 69120 Germany

**Keywords:** Chiroptical properties, Nanohoops, Rubicene, Stereoselective, Synthesis

## Abstract

The interest in chiral nanohoops and nanotubes as well as their chiroptical properties has steadily increased in recent years. The synthesis of pentagon containing enantiopure [3]cyclorubicenes, which are cutouts and potential precursors of chiral Haeckelite nanotubes is presented, based on enantiopure “corner units”. The resulting enantiopure nanohoops were studied for their electronic and chiroptical properties with one of the highest dissymmetry values reported for CPPs of up to 2.3 × 10^−2^.

## Graphical abstract

Herein, we present a high yielding synthesis of enantiopure [3]cyclorubicene, which is a potential precursor for a Haeckelite nanotube. This macrocycle was studied for its electronic and chiroptical properties and exhibits one of the highest dissymmetry values reported for a nanohoop.

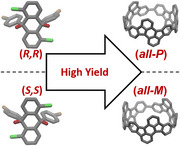

## Introduction

Since the first successful syntheses of cycloparaphenylenes (CPPs) by Jasti's and Itami's groups were reported,^[^ ^1^, ^2^ ^]^ the field has rapidly grown and a large number of CPPs or better nanohoops of various sizes from different building blocks have been generated; all having unique physical and chemical properties.^[^ ^3^, ^4^, ^5^, ^6^, ^7^ ^]^ Besides the strategies of Jasti and Itami based on kinked building blocks (dihydroxycyclohexadienes or dihydroxycyclohexanes),^[^ ^1^, ^8^, ^9^, ^10^, ^11^, ^12^, ^13^ ^]^ formation of macrocyclic intermediates via square‐planar platinum (II) or digold(I) complexes have been reported and allow the formation of nanohoops of different sizes due to other inherent angles of the bridging units.^[^ ^14^, ^15^, ^16^, ^17^, ^18^, ^19^, ^20^, ^21^ ^]^

**Scheme 1:**
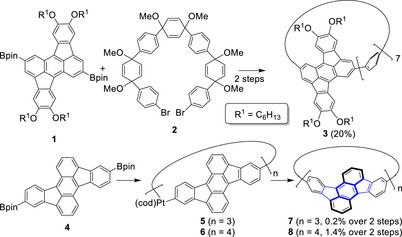
Previous strategies for the synthesis of rubicene containing CPP derivatives **3**, **7**, and **8** from their respective precursors **1**, **2**, and **4**. The 5|6|5 fused ring system of **7** is marked in blue.^[^ ^33^, ^34^ ^]^

Among the vast number of structurally different nanohoops there are also chiral ones.^[^ ^22^, ^23^, ^24^ ^]^ The most simple approach to synthesize chiral nanohoops is to use intrinsically chiral units such as helicenes^[^ ^25^, ^26^ ^]^ as building blocks. Another possibility of introducing chirality into nanohoops is the use of larger prochiral polycyclic aromatic units such as chrysene,^[^ ^27^ ^]^ 3,9‐substituted phenanthrene,^[^ ^28^ ^]^ 2,6‐substituted anthracene,^[^ ^29^ ^]^ dibenzopentalene,^[^ ^30^, ^31^ ^]^ anthanthrene^[^ ^32^ ^]^ or both 2,9‐ and 5,12‐substituted rubicene (see Scheme 1).^[^ ^33^, ^34^ ^]^ These aromatic units lose their mirror plane due to being bent in the cyclic nanohoop and therefore become chiral. In most cases the racemic or more complex mixtures of a number of stereoisomers that were generated during macrocyclization were separated by HPLC or supercritical fluid chromatography, either using polysaccharide^[^ ^33^, ^35^, ^36^ ^]^ or cholesterol^[^ ^27^, ^28^, ^29^, ^32^, ^33^, ^34^, ^37^ ^]^ based chiral column materials. Nevertheless, by this strategy also other fascinating hoop‐type compounds with topological chirality such as lemniscates,^[^ ^35^, ^38^ ^]^ knots^[^ ^39^ ^]^ or Möbius strips^[^ ^26^, ^40^ ^]^ were achieved.

More elegant than separation by chiral HPLC, is the enantiomeric resolution via chiral derivatization and separation of the resulting diastereomers of nanohoops,^[^ ^31^ ^]^ allowing in principle to work on larger scale more efficiently.

Probably the most elegant and efficient strategy to chiral nanohoops is the introduction of the chiral information by using chiral catalysts to obtain one enantiomer in excess^[^ ^25^, ^40^, ^41^, ^42^, ^43^ ^]^ as for instance for a helicene‐containing CPP derivative with ee's >99%.^[^ ^25^ ^]^ However, this kind of strategy is so far not applicable for nanohoops, where chirality is based only on bent prochiral PAH units. In this respect, the Esser group introduced a strategy to use bent chiral diketones, that after nanohoop formation gets “planarized” to dibenzopentalene units to give a planar chiral nanohoop.^[^ ^30^ ^]^ During the eight‐step synthesis, including the re‐aromatization, the chiral information was preserved to obtain both enantiomers of the resulting nanohoop in overall 7%–12% yield. To the best of our knowledge, this is the only example of such a strategy that has been reported so far.

**Figure 1:**
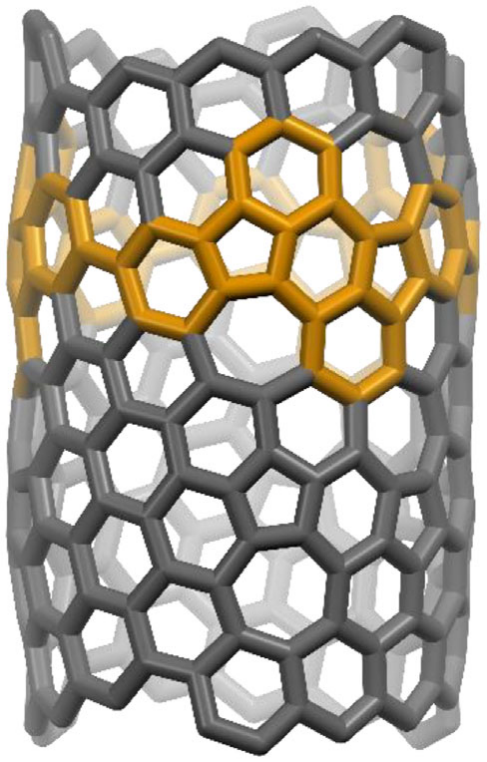
Haeckelite nanotube (MM2 optimized). The contained [3]cyclorubicene is highlighted in orange.

We got interested in rubicene‐based nanohoops, where the rubicenes are embedded via their 5,12‐positions (see structure **7** in Scheme 1), because these are potential synthetic starting materials for still elusive chiral Haeckelite carbon nanotubes as depicted in Figure 1. Although [3]cyclorubicene **7** and [4]cyclorubicene **8** were already synthesized by the platinum‐based strategy in 0.2% and 1.4% yields,^[^ ^33^ ^]^ this method gave insufficient amounts of substance for an analysis of their chiroptical properties. Unfortunately, the smaller [3]cyclorubicene that comes with lower yields, has the higher calculated barrier (51.8 kcal mol^−1^) for the inversion of a single rubicene unit,^[^ ^33^ ^]^ which corresponds to a half‐life of 1997 years at 200 °C and thus preservation of chirality under ambient conditions. This is more interesting for a potential Haeckelite synthesis. In contrast, the [4]cyclorubicenes is optically less stable with a barrier of 25.8 kcal mol^−1^.^[^ ^33^ ^]^

Here, we present a synthesis of enantiopure [3]cyclorubicenes with significantly increased yields of 9.3%–10% over 6 steps.

## Results and Discussion

**Scheme 2:**
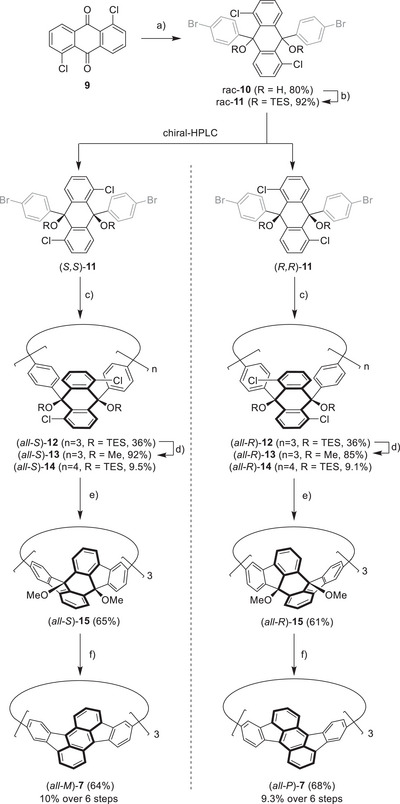
Synthesis of enantiopure **7**. Reagents and conditions: a) (i) 1,4‐dibromobenzene (2.5 equiv.), *n*BuLi (2.5 equiv.), THF, −84 °C, 30 min; (ii) **9**, THF, −84 °C→r.t., o.n.; b) NaH (4 equiv.), TESCl (6 equiv), dioxane, 50 °C→115 °C, o.n.; c) Ni(COD)_2_ (2.5 equiv.), 2,2′‐bipyridyl (2.5 equiv.), THF, 65 °C, o.n.; d) (i) NBu_4_F·3H_2_O (9 equiv.), THF, r.t., 2 h; (ii) NaH (12 equiv.), MeI (18 equiv.), THF, r.t., o.n.; e) **14**, Pd(PCy_3_)_2_Cl_2_ (60 mol%), K_2_CO_3_ (30 equiv.), DMAc, 135 °C, o.n. f) Et_3_SiH (150 equiv.), TfOH (12 equiv.), DCM, r.t., 10 min.

The synthesis of [3]cyclorubicene described herein is based on chiral building block **11** (Scheme 2) that will be re‐aromatized after macrocyclization, similar to the strategy introduced by Esser and coworkers.^[^ ^30^ ^]^

**Figure 2:**
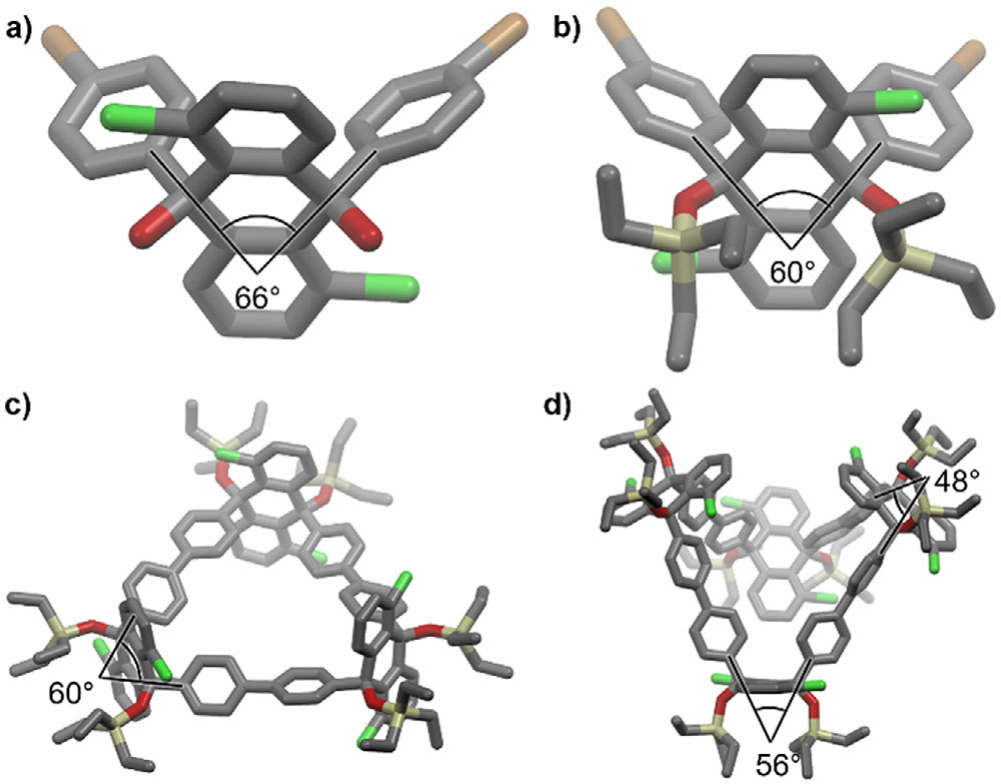
Single crystal X‐ray structure of a) *rac*‐**10**; b) (*S,S*)‐**11**; c) (*all‐S*)‐**12** and d) (*all‐S*)‐**14**. The internal angles of the corner units are highlighted. Carbon: grey, hydrogen: white, chlorine: green, silicon: beige, oxygen: red. For *rac*‐**10** only the (*R,R*) isomer is depicted.

In the first step, racemic diol **10** was synthesized in 80% yield. The simple purification protocol by washing with dichloromethane allowed making large scale quantities of up to 6.8 g of the diol. Next step was the protection with chlorotriethylsilane (TESCl) in the presence of sodium hydride to give *rac*‐**11** in 92% yield. Again, purification was simply achieved by washing with methanol. The introduction of the triethylsilyl protecting groups increased the solubility in nonpolar solvents. Therefore, chiral resolution was achieved at this stage by automated high‐performance liquid chromatography (HPLC) giving both enantiomers of **11** in ee's >99% (for experimental details, see Supporting Information). The (*S,S*)‐enantiomer was eluted first and its absolute stereochemistry was clearly assigned by single crystal X‐ray diffraction analysis of high quality single crystals (see Figure 2b).

The enantiopure building blocks (*S,S*)‐**11** and (*R,R*)‐**11** were then used in a Yamamoto coupling to form the corresponding trimeric macrocycles (*all‐S*)‐ or (*all‐R*)‐**12** in 36% yields each. The formation of the trimer was confirmed by MALDI‐TOF mass spectrometry (*m/z* 1843.5866 and 1843.5886; calculated for [M‐OTES]^+^: *m/z* 1843.5873) and single crystal X‐ray diffraction, which also confirmed the absolute configuration of (*all‐S*)‐**12** (see Figure 2c). During the Yamamoto coupling the formation of larger macrocycles was also observed (see gel permeation chromatogram and MS of the crude mixture in the Supporting Information) and the tetramers (*all‐S*)‐**14** and (*all‐R*)‐**14** were isolated in 9.1% and in 9.5% yield, respectively. Besides identification of the tetrameric structure by MS, it was also confirmed by single crystal X‐ray diffraction (see Figure 2d). The high selectivity toward the trimer formation can be explained by the small angle of the corner units, which is 66° for the diol **10** and is even reduced further to 60° by the introduction of the triethylsilyl protecting groups in **11,** as can be seen from single crystal X‐ray structures (Figure 2a,b). This angle matches closely with the optimal internal angle for a trimeric macrocycle (of 60°), which is also observed for **12** (Figure 2c). Notably, tetramer **14** shows even narrower angles (of 48° and 56°, see Figure 2d) than the building block, due to its tub conformation. In contrast, the square platinum complexes previously used for the synthesis of cyclorubicenes **7** and **8** have much larger angles of 90°, preferring a tetrameric structure (see also discussion below).^[^ ^33^, ^44^ ^]^

**Figure 3:**
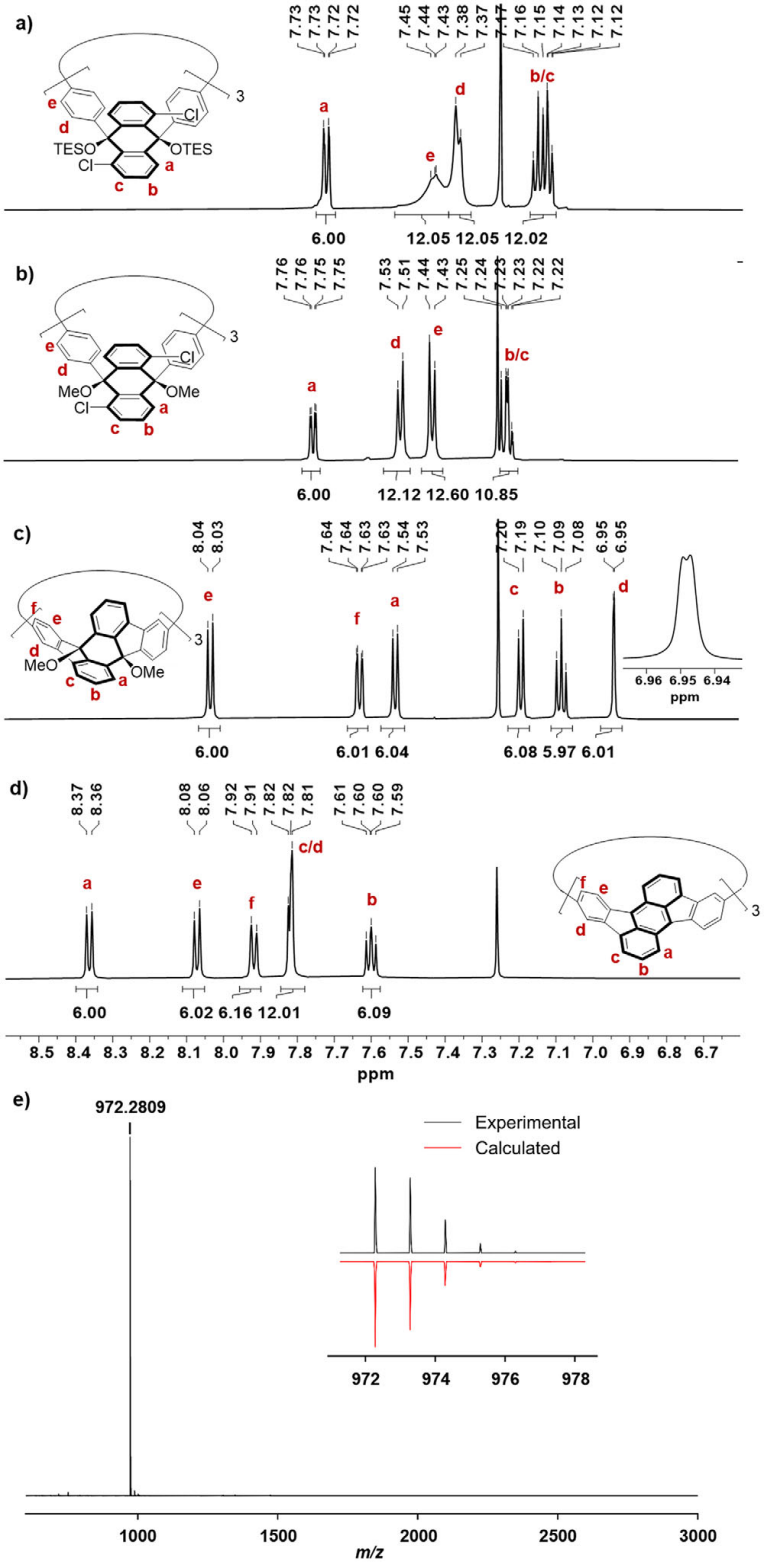
a)–d) Aromatic region of the ^1^H NMR spectra (600 MHz, CDCl_3_, 298K) of a) **12**, b) **13**, c) **15** (with zoom in for the signal of the characteristic proton H^d^) and d) **7** with signal assignment (for full spectra see Supporting Information). e) MALDI‐TOF mass spectrum (DCTB, pos. mode) of [3]cyclorubicene **7**.

Although tetrameric macrocycles *(all‐S)*‐ and *(all‐P)*‐**14** were isolated in reasonable amounts, the synthesis was continued with the trimeric macrocycles exclusively, because the targeted [3]cyclorubicene **7** is conformational much more stable than the corresponding [4]cyclorubicene **8**.^[^ ^33^ ^]^ First attempts to react the TES protected compounds in CH activation reactions failed and thus needed to be transetherified to methyl ethers were the reaction was reported to work in high yields for a simple rubicene.^[^ ^45^, ^46^ ^]^ The corresponding methyl ethers (*all‐S*)‐ and (*all‐R*)‐**13** were isolated in 92% and 85% yield. It is worth mentioning that while the phenyl protons H^d^ and H^e^ show broadened signals in the TES protected starting material (at 7.38 and 7.44 ppm, see Figure 3a), they show sharp doublets in the OMe products (at 7.52 and 7.44 ppm, see Figure 3b), as the bridging phenylene units now can freely rotate due to the reduced size of the protecting groups. The molecular structure and absolute stereochemistry of **13** was confirmed by single crystal X‐ray diffraction analysis (see Supporting Information).

**Figure 4:**
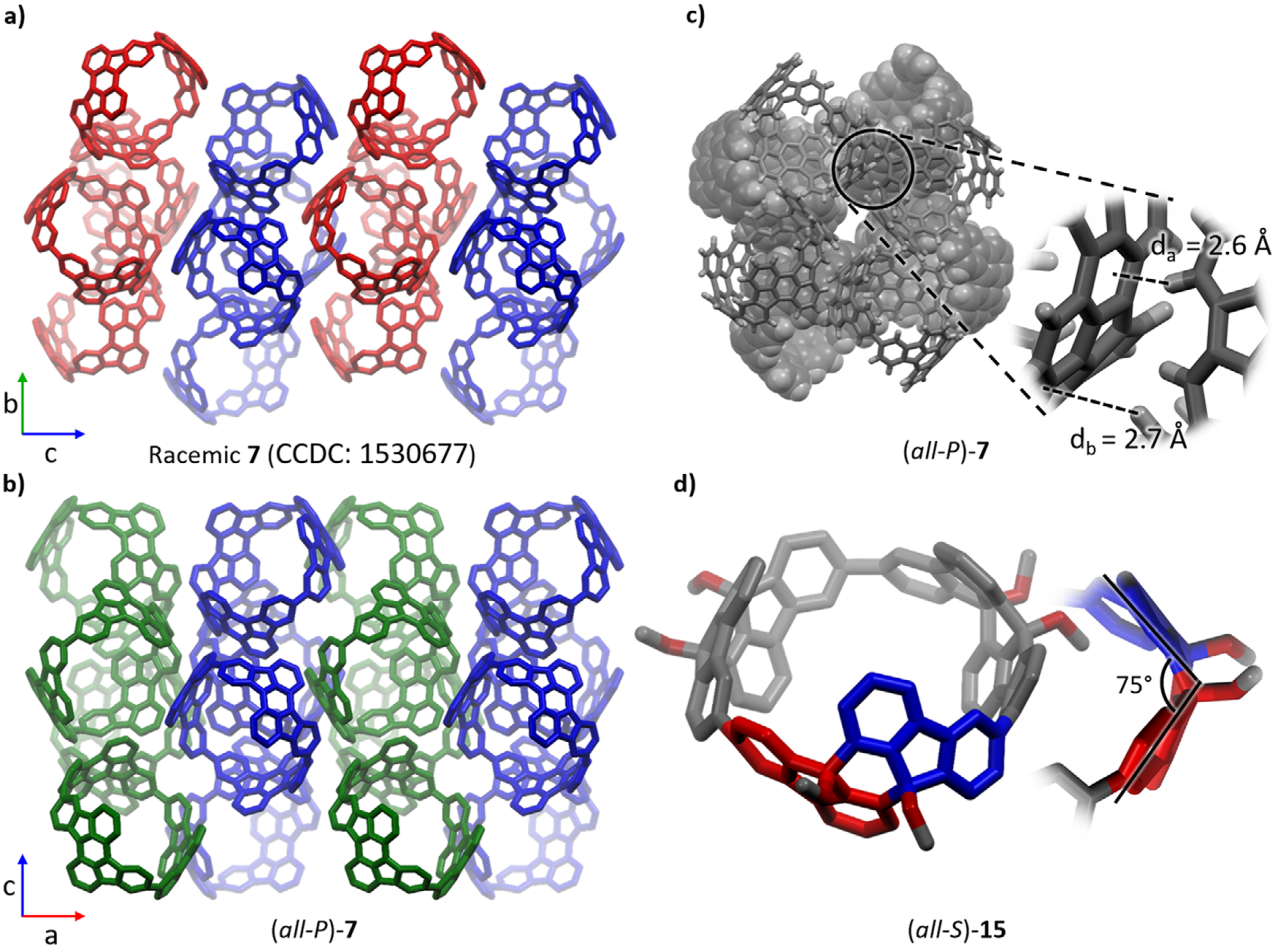
a) Packing of racemic **7** (CCDC: 1530677)^[^ ^33^ ^]^ viewed along the crystallographic *a* axis. The different enantiomers were coloured (*all‐P*: blue, *all‐M*: red) to highlight the enantiopure sheets in the *ab* plane. b) Packing of (*all‐P*)‐**7** viewed along the crystallographic *b* axis. The translated sheets in the *bc* plane are highlighted in blue and green. c) Unit cell of (*all‐P*)‐**7** viewed along the crystallographic *a* axis. *d*_a_ and *d*_b_ are the distances between the hydrogens and the π‐planes of the neighbouring molecule. d) Single crystal X‐ray structure of (*all‐S*)‐**15** with side‐view of a rubicene unit. Two fluoranthene subunits are highlighted in red in blue to show the bend of the dihydrorubicene unit toward the center of the nanohoop. Carbon: grey, hydrogen: white, oxygen: red.

The five‐membered rings in the cyclodihydrorubicene **15** were then closed by a palladium‐catalyzed CH‐activation reaction of the methoxy protected **13** using the conditions reported by Kato et al.^[^ ^45^ ^]^ (*all‐S*)‐**15** was obtained in 65% yield and its enantiomer (*all‐R*)‐**15** in 61% yield. ^1^H NMR spectroscopy shows a characteristic doublet without *ortho*‐coupling for H^d^ (at 6.95 ppm, see Figure 3c). Crystals suitable for single crystal X‐ray diffraction were obtained by layering a solution of (*all‐S*)‐**15** in chloroform with methanol. In each unit cell four nanohoops crystallize in the *P*2_1_2_1_2_1_ space group with 28 chloroform molecules of which 8 are enclathrated inside the cavities. The fluorene subunits of each dihydrorubicene (see Figure 4d, highlighted in red and blue) form an angle of 75°. This angle causes the subunits to be bent more toward the center of the hoop.

In the final step, the cyclodihydrorubicene **15** was rearomatized by reduction with triethylsilane and triflic acid,^[^ ^45^ ^]^ giving the (*all‐M*)‐enantiomer of **7** in 68% yield, and the (*all‐P*)‐enantiomer in 64% yield. The ^1^H and ^13^C NMR spectra of both enantiomers of **7** are identical to the ones previously reported for the racemic mixture of **7**.^[^ ^33^ ^]^ While the low yields reported previously, an assignment of the NMR signals via 2D spectroscopy was made unfeasible, all proton and carbon signals could now be clearly assigned (see Figure 3a for ^1^H NMR and Supporting Information for ^13^C and 2D NMR spectra). After the re‐aromatization MALDI‐TOF mass spectrometry (see Figure 3e) shows a distinct signal for the molecular ion at *m/z* 972.2814 (calculated for [M]^•+^: *m/z* 972.2812) and the ^1^H NMR signals of all aromatic protons are shifted, e. g. protons H^c^ from 7.54 in **15** to 8.36 ppm in **7** and H^d^ from 6.95 in **15** to 7.82 ppm in **7**.

By slow diffusion of ethanol into a chloroform solution of (*all‐P*)‐**7** single crystals suitable for single crystal X‐ray diffraction were obtained. While the published racemic mixture of **7** crystallizes in the *P*2_1_/n space group with *Z* = 8,^[^ ^33^ ^]^ the enantiopure compound crystallizes in the *I*222 space group with *Z* = 8. The unit cell contained disordered solvate molecules, which had to be removed by the SQUEEZE routine function.^[^ ^47^ ^]^ In the racemic crystals, both enantiomers form layers in the crystallographic *ab*‐plane (see Figure 4a, marked blue for the (*all‐P*)‐ and red for the (*all‐M*)‐enantiomer). In the enantiopure crystals, sheets with very similar packing are found along the *bc* plane (see Figure 4b, marked in blue). The sheets where the other enantiomer is found in the racemic crystals, are replaced by another sheet of the (*all‐P*)‐enantiomer (marked in green), which has been shifted by half a unit cell in each direction. In the enantiopure crystals, the dominant interactions leading to this packing are identified as perpendicular T‐shaped π–stacking, with distances of *d_a_* = 2.6 Å and *d_b_* = 2.7 Å between the nanohoops (see Figure 4c). In the racemic crystals, these distances are slightly altered to *d_a_* = 2.5 Å, *d_b_* = 2.9 Å.^[^ ^33^ ^]^ This strong similarity in packing between the racemic and enantiopure structures is likely due to the close shape match between the bottom edge of the nanohoop and its outside surface that enables this π‐stacking motif to dominate the packing.

**Figure 5:**
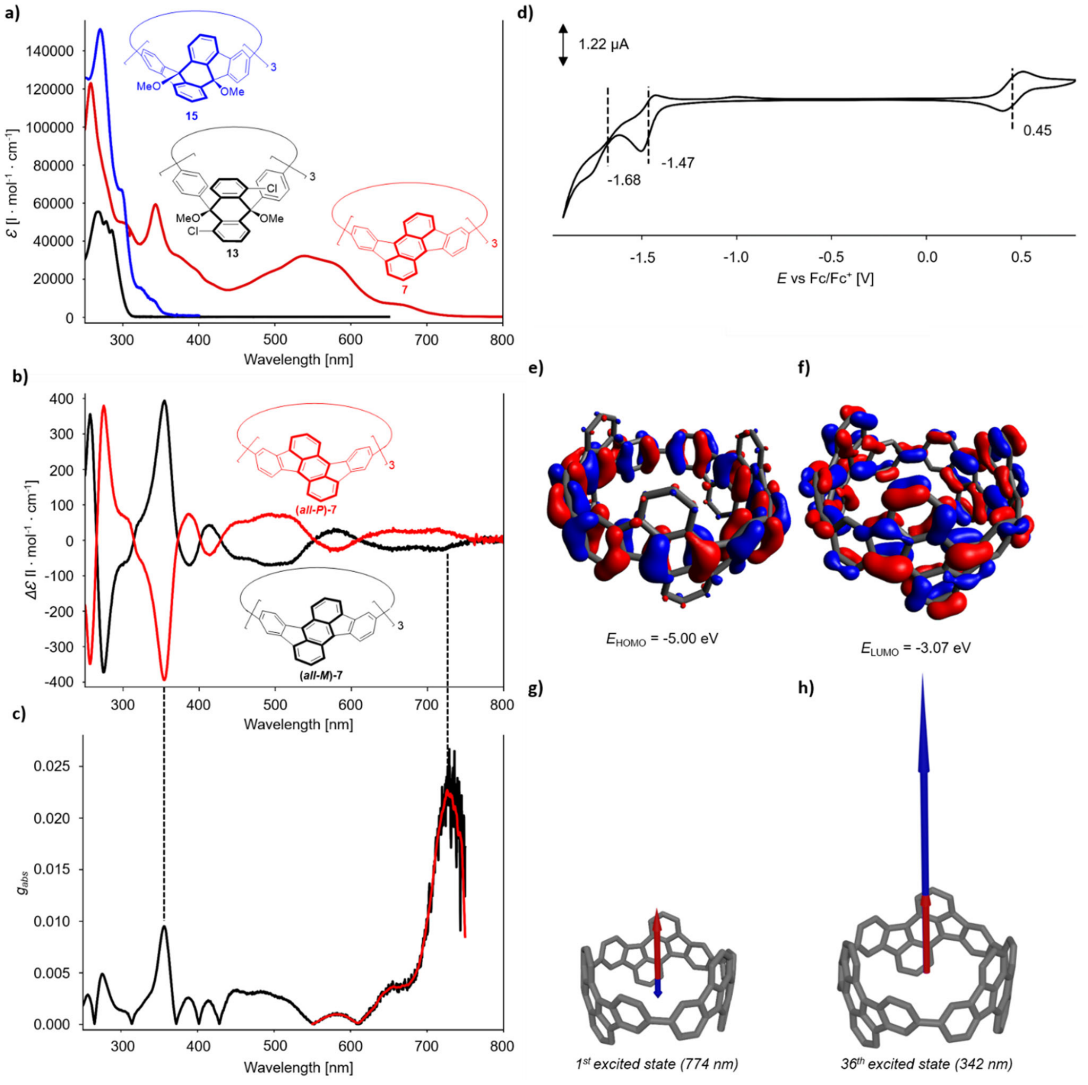
a) UV/vis absorption spectra of **7** (red), **13** (black), and **15** (blue) in dichloromethane. b) CD absorption spectrum of *(all‐M)‐* (black) and *(all‐P)‐* **7** (red) in dichloromethane. c) *g*_abs_‐profile of **7** up to 760 nm (black) and moving average smoothing from 550 nm (red). d) Cyclic voltammogram of **7** (dichloromethane, [Bu_4_N][PF_6_] (0.1 M), 100 mVs^−1^). e) HOMO and f) LUMO of **7** (isovalue: 0.02). Electric (red) and magnetic transition dipole moments (blue) from ground state to g) the first and h) the 36^th^ excited state of **7**. Calculated at PBE1PBE/6–311G(d) level of theory with GD3BJ empirical dispersion correction and dichloromethane solvation. For better visibility the magnetic dipole moments were scaled by a factor of 20.

Although the previously reported Pt‐based synthesis of racemic [3]cyclorubicene **7** enabled the study of its optical properties, the amounts were insufficient to investigate the electrochemical properties and to separate its enantiomers and study their chiroptical properties. Unlike the cyclorubicene, the methoxy‐protected precursor **13** shows only peaks between 267 and 285 nm in the UV region of its absorption spectrum (see Figure 5a). The introduction of the five‐membered rings in the dihydrorubicene derivative **15** strengthens the conjugation in the newly formed fluorene units, which is accompanied by a slight shift of the lowest energy band as a low intensity peak at *λ *= 343 nm. In contrast to these compounds, the cyclorubicene **7** has an expanded π‐system, which is evident from the large bathochromic shift of the lowest energy excitation to *λ *= 670 nm. The UV/vis absorption spectrum is identical to the one reported for the racemic compound.^[^ ^33^ ^]^ As previously described in the literature, the solutions of **7** are non‐fluorescent, which is likely due to the optically forbidden nature of the HOMO to LUMO transition (with a TD‐DFT calculated oscillator strength of *f* = 0.0004) and the lowest energy absorption maximum in this system extends further than in monomeric rubicene, where it is at 530 nm.^[^ ^33^ ^]^ The onset of the absorption is at 714 nm, which corresponds to an optical band gap of *E_gap,opt_* = 1.7 eV. Cyclic voltammetry showed a quasi‐reversible oxidation at *E_1/2_* = 0.45 V versus Fc/Fc^+^ and a first quasi‐reversible reduction at −1.47 V (see Figure 5d). This corresponds to an ionization potential of *E_IP_* = −5.25 eV and an electron affinity of *E_EA_* = −3.33 eV, making the nanohoop an electron acceptor material similar to fullerenes. The band gap as determined by cyclic voltammetry *E*_gap,CV _= 1.92 eV matches the DFT‐calculated one at *E*_gap,DFT_ = 1.93 eV very closely, but differs from the optical bandgap by about 0.2 eV, likely due to exciton binding.^[^ ^48^ ^]^ The experimental ionization potential and electron affinity are slightly lower than the calculated HOMO and LUMO energies (*E_HOMO_* = −5.00 eV, *E_LUMO_* = −3.07 eV), but still in good agreement with differences of 0.25 and 0.26 eV to the experimental values. Both HOMO and LUMO (see Figure 5e,f) extend over the three rubicene units. Within these units, the HOMO is more strongly localized in the central rings of the anthracene units and benzene units of the macrocycle, while the LUMO is delocalized over the full extent of the molecule, showing almost a quinoidal character. The LUMO shows small orbital coefficients along the single bonds connecting the rubicene units, which is again indicative of conjugation between these units.

The chiral compounds **7** and **11**–**15** show mirror image Cotton effects in circular dichroism UV/vis spectroscopy (for **11**–**15** see Supporting Information, for **7** see Figure 5b). Nanohoop **7** has very high dissymmetry factors of *g*_abs_ = 2.3 × 10^−2^ at a wavelength of 726 nm (see Figure 5c), which is comparable to dibenzopentalene containing CPPs (at 2.7 × 10^−2^),^[^ ^31^ ^]^ but lower than the exceptionally high values of chrysene containing nanohoops (1.1 to 1.7 × 10^−1^).^[^ ^49^ ^]^ This dissymmetry factor corresponds only to a small CD response of Δ*ϵ *= 18 M^−1^cm^−1^.^[^ ^50^, ^51^ ^]^ The underlying transition was assigned by TD‐DFT calculations at PBE1PBE/6–311G(d) level of theory with GD3BJ dispersion correction and dichloromethane solvation to the first excitation, which is a HOMO→LUMO transition calculated to be at 774 nm. The magnetic and electric transition dipole moments of this transition (see Figure 5g) are near anti‐parallel with an angle of 179.8°, which is optimal for high *g*_abs_‐values^[^ ^52^ ^]^ and explains the very high calculated *g*_abs_‐value of 8.9 × 10^−1^. Another local maximum of *g*_abs_ can be found at 356 nm (*g*_abs_ = 1.0 × 10^−2^), which is comparable to the *g*_abs_ values reported for other chiral CPPs (e.g., 4 × 10^−3^ for a lemniscular CPP derivative, 8.0 × 10^−3^ for a rubicene containing CPP or 9.5 × 10^−3^ for a helical CPP derivative).^[^ ^25^, ^34^, ^35^ ^]^ This corresponds well with a maximum in CD response at 354 nm (Δ*ϵ* = 394 M^−1^cm^−1^). In this case the underlying transition can be assigned to the 36^th^ excited state (calculated to be at 342 nm), which has near parallel transition dipole moments (see Figure 5h) with an angle of just 0.01°. For this transition a very high *g*_abs_‐value of 1.0 × 10^−1^ was calculated. Generally, the calculations overestimate the dissymmetry factors, which is likely due to an overlap of excitations due to line broadening in the experimental spectra.

**Figure 6:**
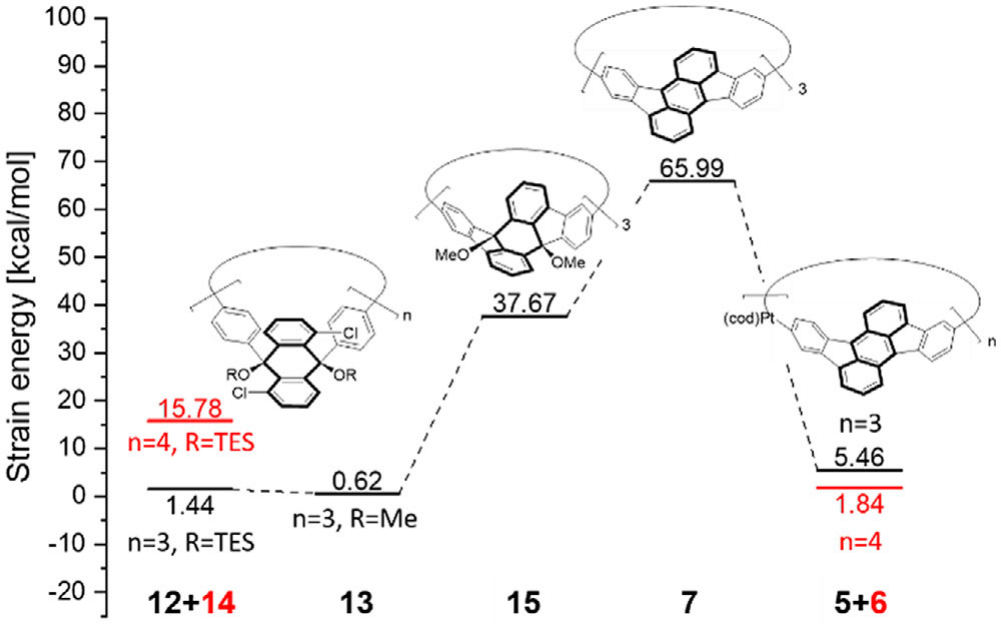
a) UV/vis strain energies and distributions of macrocycles **7** and **12–15** calculated by the StrainViz method^[^ ^53^ ^]^ at B3LYP/6–311G(d) level of theory with GD3BJ dispersion correction. The literature reported platinum cycles **5** and **6** were calculated for comparison using the 6–311G(d)/LANL2DZ basis set. The trimeric compounds are shown in black and the tetrameric compounds in red.

A crucial factor in the synthesis of cyclic aromatic compounds is the change in strain energy, which was calculated for the previously reported platinum based synthesis strategy as well as the one presented herein (see Figure 6). During the synthesis of **7** a strain energy of 66.0 kcal mol^−1^ is built up, which is comparable to the similarly sized [9]CPP (64.2 kcal mol^−1^).^[^ ^53^ ^]^ One of the main causes of this strain are the differences of the internal angles in the building blocks and macrocyclic structures (with optimal angles of 60° for the trimer and 90° for the planar tetramer). The previously used macrocyclic platinum complexes have very small strain energies of 5.46 kcal mol^−1^ for the trimer **5** and 1.84 kcal mol^−1^ for the tetramer **6,** which is formed in higher yields by this method. In contrast to this, the synthetic strategy described herein shows a lower strain energy for the cyclotrimer **12** (of 1.44 kcal mol^−1^) and higher one for the cyclotetramer **14** (of 15.78 kcal mol^−1^), explaining the observed inverse selectivity. This high difference between cyclotrimer and cyclotetramer combined with the overall low energy of the cyclotrimer is presumably one of the main reasons behind the comparably high yield (36%) for the trimer formation.

Starting from the trimeric macrocycle **12** the strain is slightly reduced by the change in protecting group to the methyl ether **13**, as this group has a lower steric demand. When the five membered rings are formed, the strain increases to 37.7 kcal mol^−1^ in **15**, before it is re‐aromatized to **7**, where the remaining 28.3 kcal mol^−1^ of strain energy are created. In contrast, the previously reported platinum based strategy requires more than 60 kcal mol^−1^ in only one step (see Figure 6, right side), which is likely a reason for the lower yields reported for the formation of racemic **7** by this strategy (0.049% over six steps),^[^ ^33^ ^]^ whereas with our two‐step method overall yields of 10% for (*all‐M*)‐**7** and 9.3% for (*all‐P*)‐**7** were achieved over the same number of steps.

## Conclusion

In summary, the two enantiomers of [3]cyclorubicene **7**, a nanohoop with π‐extended subunits have been synthesized under stereo retention in high overall yields, allowing a full characterization, especially of chiroptical properties. Enatiopure cyclorubicenes showed strong CD responses (up to Δ*ϵ* = 394 M^−1^cm^−1^) and high *g*_abs_‐values (up to 2.4 × 10^−2^) which is among the highest *g*_abs_‐values obtained for CPPs so far,^[^ ^22^, ^24^, ^25^, ^31^, ^34^, ^35^ ^]^ yet not as high as the exceptionally high values reported for chrysene based CPPs.^[^ ^49^ ^]^ The strategy presented herein, can be utilized as a more general approach toward the synthesis of pentagon‐containing enantiopure nanohoops and –belts. With its improved yields, it facilitates the use of cyclorubicenes as potential precursors for Haeckelite type nanotubes by postfunctionalization, which is currently being investigated in our laboratories.

## Supporting Information

Experimental section with synthetic procedures and the full characterization (^1^H, ^13^C, and 2D NMR spectra of all new compounds), computational details (coordinates, TD‐DFT, AICD, NICS, and StrainViz) and X‐ray crystal structure data of molecules **7** and **10–15**. Further references were cited in the Supporting Information.^[^ ^53^, ^54^, ^55^, ^56^, ^57^, ^58^, ^59^, ^60^, ^61^, ^62^, ^63^, ^64^, ^65^, ^66^, ^67^, ^68^, ^69^, ^70^, ^71^, ^72^, ^73^, ^74^, ^75^, ^76^, ^77^, ^78^, ^79^, ^80^, ^81^, ^82^, ^83^, ^84^, ^85^, ^86^, ^87^, ^88^, ^89^, ^90^, ^91^, ^92^, ^93^, ^94^, ^95^, ^96^, ^97^, ^98^, ^99^, ^100^, ^101^, ^102^, ^103^, ^104^, ^105^, ^106^, ^107^, ^108^, ^109^, ^110^, ^111^, ^112^, ^113^, ^114^, ^115^, ^116^, ^117^, ^118^, ^119^, ^120^, ^121^, ^122^, ^123^, ^124^, ^125^, ^126^, ^127^, ^128^, ^129^, ^130^, ^131^, ^132^, ^133^, ^134^, ^135^, ^136^ ^]^ The supplementary crystallographic data for this paper are provided free of charge by the joint Cambridge Crystallographic Data Centre and Fachinformationszentrum Karlsruhe.^[^ ^137^ ^]^

## Conflict of Interests

The authors declare no conflict of interest.

## Supporting information



Supporting Information

Supporting Information

## Data Availability

The data that support the findings of this study are available from the corresponding author upon reasonable request.
