# Supplementary material for: Synthesis of Enantiopure [3]Cyclorubicenes
Source: Angew Chem Int Ed Engl. 2025 Nov 10;65(1):e20880. doi: 10.1002/anie.202520880 (PMC12759246; doi:10.1002/anie.202520880)
Supplement: Supplementary file 1 — Supporting Information [file ANIE-65-e20880-s002.docx]

Synthesis of Enantiopure [3]Cyclorubicenes

Moritz P. Schuldt, Frank Rominger, Michael Mastalerz*

Organisch-Chemisches Institut

Ruprecht-Karls-Universität Heidelberg

Im Neuenheimer Feld 272, 69120 Heidelberg, Germany

E-mail: [michael.mastalerz@oci.uni-heidelberg.de](mailto:michael.mastalerz@oci.uni-heidelberg.de)

**-Supporting Information-**

Table of Contents

[2 General Remarks S3](#_Toc208319291)

[3 Experimental procedures S6](#_Toc208319292)

[3.1 Synthetic Procedures S6](#_Toc208319293)

[4 Spectra S17](#_Toc208319294)

[4.1 ^1^H NMR and ^13^C NMR spectra S17](#_Toc208319295)

[4.2 2D NMR spectra S29](#_Toc208319296)

[4.3 IR spectra S42](#_Toc208319297)

[4.4 Mass spectra S48](#_Toc208319298)

[4.5 UV/Vis and fluorescence spectra S55](#_Toc208319299)

[4.6 CD spectra S59](#_Toc208319300)

[5 Cyclic voltammograms S65](#_Toc208319301)

[6 Crystallographic data S66](#_Toc208319302)

[7 GPC and HPLC Chromatograms S74](#_Toc208319303)

[8 Computational details S77](#_Toc208319304)

[8.1 XYZ-Coordinates s77](#_Toc208319305)

[8.2 TDDFT Calculations S106](#_Toc208319306)

[8.3 AICD and NICS Calculations S107](#_Toc208319307)

[8.4 StrainViz Calculations S109](#_Toc208319308)

[9 References S113](#_Toc208319309)

# General Remarks

**Materials:** All used reagents and solvents were purchased from Fisher Scientific, Sigma Aldrich, BLDPharm, Fisher Scientific/Thermo Fisher, Honeywell, Grüssing, Merck, TCI or VWR Chemicals and used without further purification, if not mentioned otherwise. The reactions were performed under standard conditions (25°C, 1013 mbar). Complete removal of solvents was achieved by applying high vacuum (1·10^−3^ mbar).

**Thin layer and flash column chromatography:** Analytical thin layer chromatography was performed with POLYGRAM® SIL G/UV254 gel plates sold by Macherey-Nagel. Detection was accomplished using UV-light (254 nm). Flash column chromatography was performed using Silica gel 60 (40–63 μm / 230–400 mesh ASTM) purchased from Macherey-Nagel.

**Nuclear magnetic resonance (NMR):** All reported NMR spectra were recorded on a Bruker Avance III 400 (400 MHz), a Bruker Avance III 600 (600 MHz) or a Bruker Avance Neo 700 (700 MHz) spectrometer. Chemical shifts (*δ*) are given in parts per million (ppm) and coupling constants in Hertz (Hz). All spectra were calibrated relative to traces of less-deuterated solvent (CDCl_3_: 7.26 ppm / 77.16 ppm, THF-d_8_: 3.58 ppm / 67.21 ppm).^[S1]^ The following abbreviations were used for ^1^H NMR spectra to indicate the signal multiplicity: s (singlet), d (doublet), t (triplet), q (quartet), m (multiplet). All ^13^C NMR spectra were measured with ^1^H-decoupling.

**Mass spectrometry (MS):** MS experiments were performed on a JEOL AccuTOF GCx time-of-flight spectrometer (EI-MS) or a Bruker timsTOFfleX spectrometer (MALDI-MS and APCI-MS). For MALDI-MS experiments DCTB (trans-2-[3-(4-tert-Butylphenyl)-2-methylpropenylidene)malononitrile) was used as matrix. All measurements were performed by the mass spectrometry division of the University of Heidelberg under the supervision of Dr. Jürgen H. Gross.

**Infrared spectroscopy (IR):** IR spectra were recorded on a ZnSe ATR crystal using a Bruker Tensor 27 or Thermo Scientific Nicolet iS5 spectrometer with iD7 ATR module. The following abbreviations were used to indicate the absorption intensity: vw (very weak), w (weak), m (medium), s (strong), vs (very strong).

**Elemental analysis:** Elemental analysis was performed in the Microanalytical laboratory of the University of Heidelberg with an Elementar vario Micro Cube Element Analyzer.

**UV/vis and fluorescence spectroscopy:** UV/vis spectra were recorded with a Jasco V-730 spectrometer and fluorescence spectra with a Jasco FP-8300 spectrometer.

**CD spectroscopy:** Circular dichroism measurements were performed using a Jasco J-1500 spectral polarimeter equipped with a Julabo F250 circulation cooler and FLMS-526 N2 gas flow meter at 20 °C

**Cyclovoltammetry (CV)**: Cyclic voltammograms were recorded on a Metrohm Autolab PGSTAT101 potentiostat using a Pt working electrode (0.78 mm^2^), a Pt counter electrode and a Ag/Ag^+^ pseudo reference electrode in degassed HPLC-grade CH_2_Cl_2_ with NBu_4_PF_6_ as conductiong salt with a scan rate of 100 mV ⋅ s^−1^and referenced against Fc/Fc^+^ as internal standard.

**Melting points**: The non-corrected melting points were determined with a Büchi Melting Point B-565.

**X-ray crystal structure analysis:** X-ray crystal diffractograms were recorded with a Bruker APEX-II Quazar diffractometer using Mo-K_α_ radiation (*λ* = 0.71073 Å) or a STOE Stadivari diffractometer using Cu-K_α_ radiation (*λ* = 1.54178 Å). Intensities were corrected for Lorentz and polarization effects, an empirical scaling and absorption correction was applied using SADABS^[S2]^ or X-Area LANA 2.8.4 (STOE, 2024) based on the Laue symmetry of the reciprocal space (μ, Tmin, Tmax). The structures were solved with SHELXT-2014 or SHELXT-2018/2 ^[S3]^ and refined against F2 with a full-matrix least-squares algorithm using the SHELXL-2018/3 or the SHELXL-2019/2 (Sheldrick, 2019) software.^[S4]^

**Chiral HPLC:** Preparative chiral resolution was performed with a pre-packed Chiralpak® IB HPLC column by Daicel Corporation on a Shimadzu Nexera HPLC with a LC-20AP pump, FCV-20AL quaternary valve, SIL.-10AP autosampler, CTO-40C column oven CBM-4a system controller and FRC-10A fraction collector Analytical HPLC-chromatograms were recorded on a Shimadzu Nexera LC-40 analytical HPLC equipped with a LC-40D solvent delivery pump, a CTO-40C column oven, SPD-M40 photo diode array detector and an analytical Chiralpak® IB column by Daicel Corporation.

**Gel permeation chromatography (GPC):** Preparative gel permeation chromatography was performed in dichloromethane with four PSS SDV 100 Å columns and one PSS SDV 500 Å column on a Nexera LC-40 recycling GPC consisting of a LC-40D pump, a SIL-40C autosampler, a DGU-403 degassing unit, a CTO-40C column oven and a FRC-10A fraction collector. Substances were detected by a SPD-M40A photo diode array detector.

**Computational Details:** All quantum chemical calculations were performed by employing the Gaussian16 program package.^[S5]^ The theoretical approach is based on Kohn-Sham density functional methodologies^[S6-9]^ using the B3LYP^[S10-13]^ functional and GD3BJ dispersion correction.^[S14]^ As basis set the triple-ζ-basis (6-311G(d))^[S15-20]^ was used. The geometries were pre-optimized using the semi-empirical PM6 method.^[S21]^ Ground states and transition states were confirmed by using frequency calculations to exhibit no or exactly one imaginary frequency. Prediction of excited state properties were performed using time-depended DFT^[S22-33]^ using the PBE1PBE^[S34]^ functional and PCM^[S35-59]^ solvation and convoluted with GaussSum.^[S60]^ For the calculation of g_abs_ values of the excited states the electric and magnetic transition dipole vectors were converted from atomic units to cgs units using the factors 1 a.u. = 2.54175·10^-18^ esu·cm (*µ_e_*) and 1 a.u. = 9.27401·10-21 erg·G^-1^ (*µ_m_*). NICS(0) and NICS(1) values were calculated from the optimized geometries by adding a ghost atom in the center (0) or 1 Å inside (-1) or outside (+1) of the corresponding ring and performing a single-point calculation based on Hartree-Fock methods^[S61-68]^ using the triple-ζ-basis (6-311G(d))^[S15-20]^ with the GIAO method.^[S69-73]^ ICS surfaces^[S74]^ were calculated using the same level of theory and Multiwfn^[S75]^. For the NICS contour plots, the aromatic subunits were cut out of the optimized structure of [3]cyclorubicene and saturated with hydrogens. The resulting geometries were reoptimized with all atoms except the new hydrogen atoms frozen. The contour plots were then calculated using the same methodology as used for the NICS values. Ring-current analysis was accomplished by performing a single-point calculation with the CSGT method^[S73, 76-77]^ on the HF/6-311G(d) ^[S15-20, 61-68]^ level, the π-orbitals were extracted using Multiwfn^[S75, 78]^ and using the AICD program package of the Herges group.^[S79-80]^ Strain Calculations were performed using the B3LYP functional^[S10-13]^ and the triple-ζ-basis (6-311G(d))^[S15-20]^ using the StrainViz program^[S81]^ For the structures containing platinum atoms, the LANL2DZ double-ζ-basis and pseudopotentials were used.^[S82-83]^

**Assignment of the Stereodescriptors for nanohoop** **7:** To determine the stereodescriptors, a helix formed by the highest priority atom outside of the anthracene plane (10b) and the first three (highest priority) atoms in this plane (14a, 14b and 14e) was determined (see Figure S1).^[S84]^ For this helix the corresponding stereodescriptor was assigned to the nanohoop. Due to the high symmetry of nanohoop **7** (*D*_3_) all stereogenic moieties are symmetry equivalent and are therefore assigned the same stereodescriptor.


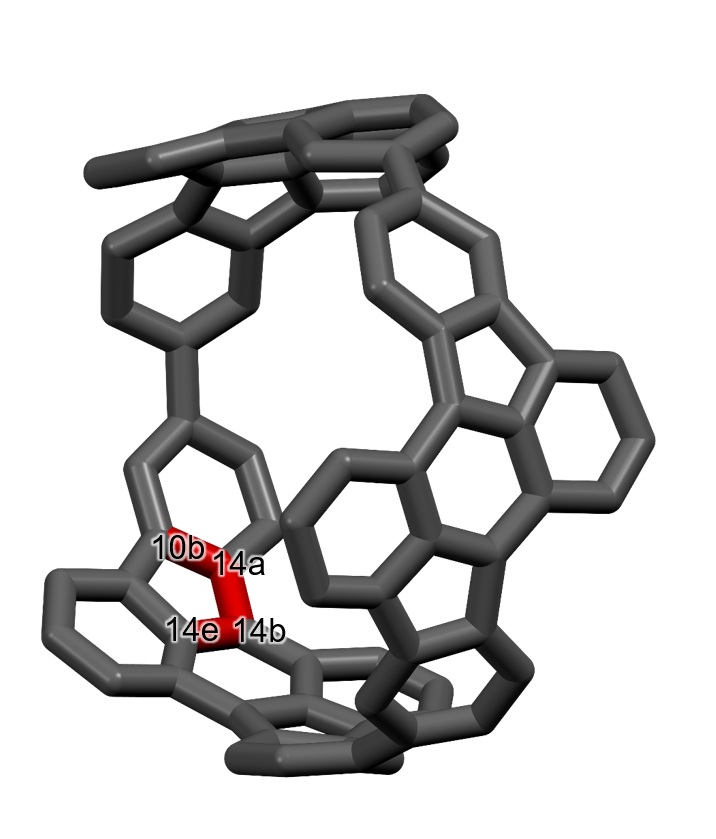


**Figure S1:** Structure of nanohoop (all-P)-**7**. The atoms 10b, 14a, 14b and 14e of one rubicene^[S84]^ unit are highlighted and the helix formed from them is highlighted in red.

# Experimental procedures

## Synthetic Procedures

**Synthesis of syn-9,10-bis(4-bromophenyl)-1,5-dichloro-9,10-dihydroanthracene-9,10-diol (****10):**

1,4-Dibromobenzene (8.51 g, 36.1 mmol) was dissolved in dry tetrahydrofuran (30 mL) under an argon atmosphere and cooled to -84°C. Over the course of 5-10 min, *n*-butyllithium (2.5 M solution in n-hexane, 14.4 mL, 36.1 mmol) was added and the solution was stirred for 30 min. A suspension of 1,5-dichloro-9,10-anthraquinone (4.00 g, 14.4 mmol) in dry tetrahydrofuran (30 mL) was added and the reaction was allowed to warm up to r.t. overnight. Water (50 mL) was added and the reaction mixture was extracted with tetrahydrofuran (2x 100 mL) and the organic layers combined. The solvents were removed *in vacuo* and the crude product was suspended in dichloromethane (50 mL), sonicated, filtered and washed with dichloromethane (50 mL) to obtain the diol **10** as a colorless solid (6.82 g, 80%). **M.p.:** >283 °C decomp.**^1^H NMR (600 MHz, THF-*d*_8_):** *δ* = 7.78 (d, *J* = 7.8 Hz, 2H, H‑6), 7.42 (d, *J* = 8.6 Hz, 4H, H‑10), 7.39 (d, *J* = 8.7 Hz, 4H, H‑11), 7.28-7.23 (m, 4H, H1+H2), 5.70 (s, 2H, -OH).**^13^C NMR (151 MHz, THF-*d*_8_):** *δ* = 148.5 (C‑8), 145.0 (C‑3/5), 135.5 (C‑4), 134.7 (C‑3/5), 131.9 (C‑10), 131.7(C‑2), 130.0(C‑1), 129.4(C‑6), 129.3(C‑11), 121.4 (C‑9), 74.1 (C‑7). **IR (ATR):** 3522 (w), 3411 (m), 1588 (w), 1562 (w), 1483 (m), 1452 (m), 1430 (m), 1398 (m), 1366 (w), 1340 (w), 1296 (w), 1242 (w), 1206 (w), 1182 (w), 1151 (m), 1110 (w), 1074 (m), 1038 (m), 1007 (vs), 968 (m), 927 (w), 918 (w), 890 (m), 823 (m), 811 (s), 789 (s), 762 (m), 741 (vs), 723 (s), 698 (s), 663 (m). **UV-Vis (dichloromethane):** *λ*_max_ (lg *ε*) = 280 (3.41), 272 (3.20) nm. **MS (HR-EI):** *m/z* calculated for [M]^+^: 587.8889, found 587.8878. **Elemental Analysis:** calculated for C_26_H_16_Br_2_Cl_2_O_2_·0.33CH_2_Cl_2_: C 51.06%, H 2.71%; found: C 50.80%, H 2.87%.

**Synthesis of syn-9,10-bis(4-bromophenyl)-1,5-dichloro-9,10-dihydroanthracene-9,10-diyl)bis(oxy))bis(triethylsilane) (****11):**

Diol **10** (3.00 g, 5.08 mmol) was suspended in dry 1,4-dioxane (60 mL) in a Schlenk flask with an overpressure valve. Sodium hydride (60 wt.% in mineral oil, 812 mg, 20.3 mmol) was added and the solution was heated to 50 °C for 30 min. Triethylsilylchloride (5.2 mL, 30.5 mmol) was added and the reaction was stirred at 115°C overnight. The reaction mixture was cooled down to r.t., NaHCO_3_ solution (conc., 10 mL) and methanol (50 mL) were added and the resulting precipitate was collected by filtration and washed with methanol (100 mL) and dried *in vaccuo* to obtain silyl ether **11** as colorless solid (4.65 g, 92%). **M.p.:** 234°C **^1^H NMR (400 MHz, CDCl_3_):** *δ* = 7.75 (dd, *J* = 7.8, 1.7 Hz, 2H, H‑3), 7.31 (d, *J* = 8.9 Hz, 4H, H‑9), 7.25-7.12 (m, 8H, H‑arom.), 0.91 (t, *J* = 8.0 Hz, 18H, H‑13), 0.56-0.34 (m, 12H, H‑12). **^13^C NMR (101 MHz, CDCl_3_):** *δ* = 148.8 (Cq.), 143.1 (Cq.), 134.9 (Cq.), 133.9 (Cq.), 131.5 (C‑1/2), 131.0 (C‑9), 129.2 (C‑1/2), 128.7 (C‑3), 127.9 (C‑10), 120.8 (Cq.), 75.8 (C‑7), 7.5 (C‑13), 6.5 (C‑12). **IR (ATR):** 2951 (m), 2923 (m), 2873 (m), 2853 (w), 1592 (w), 1561 (w), 1483 (m), 1449 (m), 1427 (m), 1411 (w), 1391 (m), 1377 (w), 1237 (m), 1206 (m), 1156 (s), 1129 (m), 1113 (m), 1092 (s), 1071 (s), 1008 (vs), 980 (m), 941 (w), 929 (m), 916 (m), 896 (m), 869 (m), 844 (m), 823 (s), 815 (s), 793 (m), 766 (w), 746 (vs), 732 (vs), 716 (vs), 698 (s), 679 (s), 668 (m), 667 (m), 627 (w), 587 (m). **UV-Vis (dichloromethane):** *λ*_max_ (lg *ε*) = 263 (3.34), 272 (3.36), 285 (3.11) nm. **MS (HR-APCI):** *m/z* calculated for [M-OTES]^+^: 684.9726, found 684.9738. **Elemental Analysis:** calculated for C_38_H_44_Br_2_Cl_2_O_2_Si_2_: C 55.68%, H 5.41%; found: C 55.63%, H 5.37%.

**Chiral resolution of syn-9,10-bis(4-bromophenyl)-1,5-dichloro-9,10-dihydroanthracene-9,10-diyl)bis(oxy))bis(triethylsilane) (****11):**

TES ether **11** (4.00 g, 4.88 mmol) was dissolved in *n*-heptane (400 mL). The enantiomers were separated by chiral HPLC (CHIRALPAK® IB, 9 mL/min, *n*-heptane, 5 mL injection volume, retention times: 9.27 min and 12.39 min) to obtain (*S,S*)-**11** (1.74 g) and (*R,R*)-**11** (1.75 g).

The absolute configuration of the first enantiomer was determined via SCXRD.

(*S,S*)-**11** (retention time: 9.27 min)**: M.p.:** 253 °C**;** ${[\boldsymbol{\alpha}]}_{\boldsymbol{D}}^{\boldsymbol{20}}$ (c = 0.921): -147 °⋅mL⋅dm^-1^⋅g^-1^

(*R,R*)-**11**  (retention time: 12.39 min)**: M.p.:** 253 °C**;** ${[\boldsymbol{\alpha}]}_{\boldsymbol{D}}^{\boldsymbol{20}}$ (c = 0.837): 133°⋅mL⋅dm^-1^⋅g^-1^

**Synthesis of chiral macrocycles** (*all-S*)*-***12** and (*all-S*)*-***14:**

Ni(COD)_2_ (210 mg, 0.76 mmol) was dissolved in dry tetrahydrofuran (60 mL) under an argon atmosphere. A solution of 2,2'-bipyridine (119 mg, 0.76 mmol) in tetrahydrofuran (30 mL) was added slowly. A solution of (*S,S*)-**11** (250 mg, 0.31 mmol) in tetrahydrofuran (30 mL) was added and the mixture was heated to 65°C overnight. The reaction mixture was cooled to r.t., the solvents were removed *in vacuo,* the remaining solid was filtered through a silica plug, which was washed with dichloromethane (150 mL). The solvents were removed in vacuo and the remaining solid was purified by column chromatography (SiO_2_, 10% to 15% dichloromethane/petrol ether, *R*_f_ (15% DCM) = 0.46 (**12**), 0.21 (**14**)) to give after washing with *n*-pentane (2 mL) and drying *in vacuo* (*all-S*)-**12** (80.6 mg, 30%) and (*all-S*)-**14** (25.1 mg, 9.5%) as colorless solids.

Alternative purification of (*all-S*)*-***12:** On a larger scale, (*all-S*)*-***12** was prepared from (*S,S*)-**11** (500 mg, 0.61 mmol) according to the procedure above and purified by gel permeation chromatography. After washing with *n*-pentane (2 mL), drying *in vacuo* a slightly higher yield (145 mg, 36%) was obtained.

(*all-S*)*-***12:**

**M.p.:** >327 °C decomp. **^1^H NMR (600 MHz, CDCl_3_):** *δ* = 7.73 (dd, *J* = 7.9, 1.7, 6H, H‑3), 7.54-7.41 (m, 12H, H‑8), 7.37 (d, *J* = 8.0, 12H, H‑7), 7.19-7.11 (m, 12H, H‑1+H‑2), 0.95 (t, *J* = 7.9, 54H, H‑13), 0.49 (ddt, *J* = 34.2, 15.3, 7.7, 36H, H‑12). **^13^C NMR (151 MHz, CDCl_3_):** *δ* = 148.9 (C‑9), 143.6 (C‑11), 139.3 (C‑6), 134.9 (C‑4), 133.9 (C‑10), 131.0 (C‑1), 129.0 (C‑2/3), 129.0 (C‑2/3), 126.7 (C‑7), 126.2 (C‑8), 76.1 (C‑5), 7.6 (C‑13), 6.6 (C‑12). **IR (ATR):** 2951 (w), 2936 (w), 2909 (w), 2874 (w), 1562 (w), 1492 (w), 1450 (m), 1427 (w), 1413 (w), 1238 (m), 1209 (w), 1165 (m), 1155 (m), 1131 (m), 1093 (s), 1005 (s), 981 (m), 933 (w), 917 (m), 898 (m), 876 (m), 854 (m), 842 (m), 816 (s), 794 (m), 751 (s), 739 (vs), 725 (vs), 707 (s), 683 (m), 653 (m), 587 (m). **UV-Vis (dichloromethane):** *λ*_max_ (lg *ε*) = 269 (4.88), 280 (4.84), 287 (4.79) nm. **Fluorescence (dichloromethane, *λ*_exc_ = 260 nm):** *λ*_max_ = 316, 326 nm. **MS (HR-MALDI-TOF, DCTB):** *m/z* calculated for [M-OTES]^+^: 1843.5873, found 1843.5866. **Elemental Analysis:** calculated for C_114_H_132_Cl_6_O_6_Si_6_: C 69.17%, H 6.72%; found: C 68.84%, H 6.54%. ${[\boldsymbol{\alpha}]}_{\boldsymbol{D}}^{\boldsymbol{20}}$ (c = 0.188): -178°⋅mL⋅dm^-1^⋅g^-1^

*all-S*)*-***14:**

**M.p.:** >318 °C decomp. **^1^H NMR (700 MHz, CDCl_3_):** *δ* = 7.95 (dd, *J* = 8.3, 1.3, 4H, H‑3), 7.83 (dd, *J* = 8.3, 1.5, 4H, H‑3), 7.26 (t, *J* = 7.8, 4H, H‑2), 7.22 (dd, *J* = 7.6, 1.5, 4H, H‑1), 7.18 (t, *J* = 8.0, 4H, H‑2), 7.14 (d, *J* = 8.0, 8H, H‑7), 7.07 (td, *J* = 6.7, 6.1, 3.3, 12H, H‑1+7), 0.91 (dt, *J* = 20.8, 8.0, 72H, H‑13), 0.52-0.38 (m, 48H, H‑12). **^13^C NMR (176 MHz, CDCl_3_):** *δ* = 148.3 (C‑6), 147.7 (C‑6), 143.6 (C‑11), 143.4 (C‑11), 140.3 (C‑9), 138.1 (C‑9), 135.0 (C‑4/10), 134.8 (C‑4/10), 134.7 (C‑4/10), 134.3 (C‑4/10), 131.2 (C‑1), 130.9 (C‑1), 128.9 (C‑2/3), 128.8 (C‑2/3), 128.7 (C‑2/3), 128.5 (C‑2/3), 126.9 (C‑7), 126.7 (C‑8), 126.4 (C‑7), 125.8 (C‑8), 76.0 (C‑5), 75.8 (C‑5), 7.6 (C‑13), 6.6 (C‑12), 6.5 (C‑12). **IR (ATR):** 2952 (m), 2933 (w), 2875 (m), 1494 (w), 1449 (m), 1427 (m), 1415 (w), 1238 (m), 1209 (w), 1166 (m), 1155 (m), 1131 (w), 1118 (w), 1090 (s), 1070 (m), 1004 (s), 979 (m), 917 (m), 897 (m), 876 (w), 840 (s), 817 (m), 792 (m), 738 (vs), 721 (vs), 704 (s), 684 (s), 658 (m), 587 (m). **UV-Vis (dichloromethane):** λ_max_ (lg ε) = 268 (5.04), 279 (5.00), 286 (4.95) nm. **Fluorescence (dichloromethane, λ_exc_ = 270 nm):** λ_max_ = 314, 326 nm. **MS (HR-MALDI-TOF, DCTB):** *m/z* calculated for [M-OTES]^+^: 2506.8149, found 2506.8221. ${[\boldsymbol{\alpha}]}_{\boldsymbol{D}}^{\boldsymbol{20}}$ (c = 0.376): -336°⋅mL⋅dm^-1^⋅g^-1^

**Synthesis of chiral macrocycles** (*all-R*)-**12** and (*all-R*)*-***14:**

(*all-R*)-**12** and (*all-R*)-**14** were prepared from (*R,R*)-**11** (250 mg, 0.31 mmol) according to the procedure for the preparation of (*all-S*)-**12** and (*all-S*)-14 as decribed above, giving (*all-R*)-**12** (76 mg, 29%) and (*all-R*)-**14** (24.2 mg, 9.1%) were obtained as colorless solid.

On a larger scale, (*all-R*)-**12** was prepared from (*R,R*)-**11** (500 mg, 0.61 mmol) and purified by gel permeation chromatography according to the procedure for the preparation of (*all-S*)-**12.** (*all-R*)-**12** was obtained as colorless solid (144 mg, 36%).

(*all-R*)-**12**:

**M.p.:** >325 °C decomp. **^1^H NMR (600 MHz, CDCl_3_):** *δ* = 7.73 (dd, *J* = 8.0, 1.7, 6H, H‑3), 7.51-7.41 (m, 12H, H‑8), 7.38 (d, *J* = 7.9, 12H, H‑7), 7.19-7.11 (m, 12H, H‑1+H‑2), 0.95 (t, *J* = 7.9, 54H, H‑13), 0.57-0.41 (m, 36H, H‑12). **^13^C NMR (151 MHz, CDCl_3_):** *δ* = 148.9(C‑9), 143.6 (C‑11), 139.3(C‑6), 134.9(C‑4), 133.9(C‑10), 131.0(C‑1), 129.0 (C‑2/3), 128.9 (C‑2/3), 126.7 (C‑7), 126.2 (C‑8), 76.1 (C‑13), 7.6 (C‑13), 6.6 (C‑12). **IR (ATR):** 2952 (w), 2936 (w), 2909 (w), 2874 (w), 1562 (w), 1492 (w), 1449 (m), 1427 (m), 1415 (w), 1238 (m), 1209 (w), 1165 (m), 1155 (m), 1131 (w), 1093 (s), 1004 (s), 981 (m), 933 (w), 917 (w), 898 (m), 876 (w), 854 (m), 842 (m), 816 (s), 794 (m), 751 (s), 739 (vs), 726 (vs), 707 (s), 683 (m), 653 (m), 587 (m). **MS (HR-MALDI-TOF, DCTB):** *m/z* calculated for [M-OTES]^+^: 1843.5873, found 1843.5886. ${[\boldsymbol{\alpha}]}_{\boldsymbol{D}}^{\boldsymbol{20}}$ (c = 1.339): 161°⋅mL⋅dm^-1^⋅g^-1^

(*all-R*)-**14**:

**M.p.:** >317 °C decomp. **^1^H NMR (600 MHz, CDCl_3_):** *δ* = 7.95 (dd, *J* = 8.4, 1.4, 4H, H‑3), 7.83 (dd, *J* = 8.1, 1.6, 4H, H‑3), 7.27 (t, *J* = 7.9, 4H, H‑2), 7.23 (dd, *J* = 7.7, 1.5, 4H, H‑1), 7.18 (t, *J* = 8.0, 4H, H‑2), 7.14 (d, *J* = 8.0, 8H, H‑7), 7.09-7.05 (m, 12H, H‑1+7), 0.91 (dt, *J* = 17.6, 8.0, 72H, H‑13), 0.53 – 0.37 (m, 48H, H‑12). **^13^C NMR (151 MHz, CDCl_3_):** *δ* = 148.5 (C‑6), 147.8 (C‑6), 143.8 (C‑11), 143.5 (C‑11), 140.4 (C‑9), 138.2 (C‑9), 135.2 (C‑4/10), 134.9 (C‑4/10), 134.8 (C‑4/10), 134.5 (C‑4/10), 131.3 (C‑1), 131.0 (C‑1), 129.0 (C‑2/3), 128.9 (C‑2/3), 128.8 (C‑2/3), 128.6 (C‑2/3), 127.1 (C‑7), 126.8 (C‑8), 126.6 (C‑7), 126.0 (C‑8), 76.2 (C‑5), 75.9 (C‑5), 7.7 (C‑13), 6.8 (C‑12), 6.7 (C‑12). **IR (ATR):** 2952 (m), 2933 (m), 2874 (m), 1558 (w), 1494 (w), 1449 (m), 1427 (m), 1237 (m), 1209 (w), 1166 (m), 1155 (m), 1130 (w), 1091 (s), 1068 (m), 1004 (s), 979 (m), 934 (w), 917 (m), 897 (m), 876 (w), 841 (s), 817 (m), 792 (m), 740 (vs), 722 (vs), 705 (s), 684 (m), 658 (m), 588 (w). **MS (HR-MALDI-TOF, DCTB):** *m/z* calculated for [M-OTES]^+^: 2506.8149, found 2506.8196. ${[\boldsymbol{\alpha}]}_{\boldsymbol{D}}^{\boldsymbol{20}}$ (c = 0.865): 346°⋅mL⋅dm^-1^⋅g^-1^

**Synthesis of chiral macrocycle** (*all-S*)*-***13:**

Macrocycle (*all-S*)*-***12** (343 mg, 173 μmol) was dissolved in tetrahydrofuran (14 mL), NBu_4_F · 3H_2_O (492 mg, 1.56 mmol) was added and the reaction was stirred for 2 hours at room temperature. Water was added (20 mL) and the mixture was extracted with dichloromethane (3 x 50 mL), the extracts combined and dried over magnesium sulfate. Solvents were removed *in vacuo*. The solid was dissolved in dry tetrahydrofuran (14 mL), sodium hydride (60% wt in mineral oil, 83 mg, 2.08 mmol) and methyl iodide (0.19 mL, 3.12 mmol) were added and the resulting mixture stirred at room temperature overnight. Triethylamine (1 mL) was added and the mixture stirred for 30 min, before water (10 mL) and methanol (2 mL) were added. The precipitate was removed by filtration and dissolved in dichloromethane (5 mL). Solvents were removed *in vacuo.* The resulting solid were washed with *n*-pentane (2 mL) and dried *in vacuo* to obtain the methyl ether (*all-S*)-**13** as colorless solid (220 mg, 92%). **M.p.:** >373 °C decomp. **^1^H NMR (600 MHz, CDCl_3_):** *δ* = 7.76 (dd, *J* = 7.7, 1.9, 6H, H‑2), 7.52 (d, *J* = 8.1, 12H, H‑7), 7.44 (d, *J* = 8.5, 12H, H‑8), 7.25-7.20 (m, 12H, H‑1+H‑2), 3.02 (s, 18H, H‑12). **^13^C NMR (151 MHz, CDCl_3_):** *δ* = 146.2 (C‑6), 143.1 (C‑4), 139.6 (C‑9), 134.2 (C‑11), 132.8 (C‑10), 131.3 (C‑1), 129.8 (C‑2), 127.4 (C‑3), 127.0 (C‑8), 126.5 (C‑7), 79.3 (C‑5), 51.3 (C‑12). **IR (ATR):** 2935 (w), 2896 (vw), 2822 (w), 1592 (vw), 1562 (w), 1495 (w), 1451 (w), 1426 (m), 1395 (vw), 1241 (w), 1210 (w), 1176 (w), 1166 (w), 1147 (m), 1117 (w), 1088 (s), 1006 (m), 993 (vw), 958 (w), 938 (w), 927 (w), 899 (vw), 852 (w), 833 (w), 816 (vs), 795 (s), 777 (w), 759 (s), 742 (s), 708 (s), 659 (w), 646 (w), 592 (w), 583 (w). **UV-Vis (dichloromethane):** *λ*_max_ (lg *ε*) = 267 (4.76), 277 (4.71), 285 (4.67) nm. **Fluorescence (dichloromethane, λ_exc_ = 276 nm):** λ_max_ = 314, 325 nm_._ **MS (HR-MALDI-TOF, DCTB):** *m/z* calculated for [M]^+^: 1374.2516, found 1374.2539. **Elemental Analysis:** calculated for C_84_H_60_Cl_6_O_6_: C 73.21%, H 4.39%; found: C 73.12%, H 4.79%. ${[\boldsymbol{\alpha}]}_{\boldsymbol{D}}^{\boldsymbol{20}}$ (c = 0.390): -211°⋅mL⋅dm^-1^⋅g^-1^

**Synthesis of chiral macrocycle** (*all-R*)*-***13:**

(*all-R*)-**13** was prepared from (*all-R*)-**12** (321 mg, 162 μmol) according to the procedure for the preparation of (*all-S*)-**13.** (*all-R*)-**13** was obtained as colorless solid (191 mg, 85%). **M.p.:** >373 °C decomp. **^1^H NMR (600 MHz, CDCl_3_):** *δ* = 7.76 (dd, *J* = 7.7, 1.9, 6H, H‑2), 7.52 (d, *J* = 8.1, 12H, H‑7), 7.44 (d, *J* = 8.4, 12H, H‑8), 7.25-7.21 (m, 12H, H‑1+H‑2), 3.02 (s, 18H, H‑12). **^13^C NMR (151 MHz, CDCl_3_):** *δ* = 146.2 (C‑6), 143.1 (C‑4), 139.6 (C‑9), 134.2 (C‑11), 132.8 (C‑10), 131.3 (C‑1), 129.8 (C‑2), 127.4 (C‑3), 127.0 (C‑8), 126.5 (C‑7), 79.3 (C‑5), 51.3 (C‑12). **IR (ATR):** 2925 (m), 2869 (w), 2854 (w), 2822 (w), 1592 (w), 1562 (w), 1495 (m), 1450 (m), 1425 (m), 1393 (w), 1377 (w), 1242 (w), 1208 (m), 1176 (w), 1166 (w), 1147 (m), 1117 (w), 1088 (s), 1006 (m), 958 (m), 938 (m), 928 (w), 832 (w), 815 (vs), 795 (s), 776 (w), 758 (s), 742 (s), 738 (s), 707 (vs), 659 (m), 646 (w), 595 (w), 582 (w). **MS (HR-MALDI-TOF, DCTB):** *m/z* calculated for [M]^+^: 1374.2516, found 1374.2538. ${[\boldsymbol{\alpha}]}_{\boldsymbol{D}}^{\boldsymbol{20}}$ (c = 0.393): 227°⋅mL⋅dm^-1^⋅g^-1^

**Synthesis of chiral macrocycle** (*all-S*)*-***15:**

Macrocycle (*all-S*)*-***13** (50.0 mg, 36.3 μmol), Pd(PCy)_3_Cl_2_ (16.1 mg, 21.8 μmol) and potassium carbonate (150 mg, 1.09 mmol) were suspended in dry dimethylacetamide (3 mL) under an argon atmosphere. The suspension was heated to 135°C and stirred overnight. The reaction mixture was cooled to r.t. and poured into water (50 mL) and the resulting precipitate collected by filtration and washed with water (10 mL) and methanol (10 mL). The resulting solid was purified by column chromatography (SiO_2_, dichloromethane to 10% ethyl acetate/dichloromethane, *R*_f_ = 0.23) followed by precipitation from chloroform (5 mL) with methanol (15 mL). The product was washed with *n*-pentane (2 mL) and dried *in vacuo* to obtain (*all-S*)-**15** as off-white solid (26.8 mg, 64%). **M.p.:** >240 °C decomp. **^1^H NMR (600 MHz, CDCl_3_):** *δ* = 8.04 (d, *J* = 8.0, 6H, H‑7), 7.64 (dd, *J* = 8.1, 1.7, 6H, H‑8), 7.54 (dd, *J* = 7.8, 0.9, 6H, H‑3), 7.21 (dd, *J* = 7.7, 0.8, 6H, H‑1), 7.10 (t, *J* = 7.6, 6H, H‑2), 6.96 (d, *J* = 1.7, 6H, H‑12), 3.33 (s, 3H, H‑14). **^13^C NMR (151 MHz, CDCl_3_):** *δ* = 148.1 (C‑10), 144.8 (C‑9), 141.8 (C‑13), 140.2 (C‑6+C‑11), 138.1 (C‑4), 129.2 (C‑2), 128.2 (C‑7), 125.8 (C‑3), 124.6 (C‑12), 123.9 (C‑8), 118.6 (C‑1), 83.2 (C‑5), 53.3 (C‑14). **IR (ATR):** 2978 (w), 2929 (w), 2896 (w), 2818 (w), 1604 (w), 1574 (w), 1557 (w), 1484 (w), 1454 (m), 1414 (w), 1389 (w), 1277 (w), 1263 (w), 1246 (w), 1195 (w), 1179 (w), 1158 (m), 1106 (w), 1078 (vs), 1063 (s), 998 (m), 972 (m), 946 (m), 918 (m), 894 (m), 880 (m), 863 (m), 839 (m), 821 (s), 805 (m), 796 (s), 745 (vs), 725 (m), 683 (m), 659 (w), 641 (m), 626 (s). **UV-Vis (dichloromethane):** λ_max_ (lg ε) = 271 (5.18), 299 (4.82), 325 (4.19), 343 (3.91) nm. **MS (HR-MALDI-TOF, DCTB):** *m/z* calculated for [M]^+^: 1158.3915, found 1158.3926. **Elemental Analysis:** calculated for C_84_H_54_O_6_ · 1.5 C_4_H_8_O_2_: C 83.70%, H 5.15%, found: C 83.39%, H 5.02%. ${[\boldsymbol{\alpha}]}_{\boldsymbol{D}}^{\boldsymbol{20}}$ (c = 0.034): -1758°⋅mL⋅dm^-1^⋅g^-1^

**Synthesis of chiral macrocycle** (*all-R*)*-***15:**

(*all-R*)-**15** was prepared from (*all-R*)-**13** (50.0 mg, 36.3 μmol) according to the procedure for the preparation of (*all-S*)-**15.** (*all-R*)-**15** was obtained as colorless solid (25.6 mg, 61%).

**M.p.:** >240 °C decomp. **^1^H NMR (600 MHz, CDCl_3_):** *δ* = 8.04 (d, *J* = 8.1, 6H, H‑7), 7.63 (dd, *J* = 8.0, 1.7, 6H, H‑8), 7.54 (d, *J* = 7.7, 6H, H‑3), 7.20 (d, *J* = 7.6, 6H, H‑1), 7.09 (t, *J* = 7.6, 6H, H‑2), 6.95 (d, *J* = 1.7, 6H, H‑12), 3.33 (s, 16H, H‑14). **^13^C NMR (151 MHz, CDCl_3_):** *δ* = 148.1 (C‑10), 144.8 (C‑9), 141.8 (C‑13), 140.2 (C‑6+C‑11), 138.1 (C‑4), 129.2 (C‑2), 128.2 (C‑7), 125.8 (C‑3), 124.6 (C‑12), 123.9 (C‑8), 118.6 (C‑1), 83.2 (C‑5), 53.3 (C‑14). **IR (ATR):** 2981 (w), 2928 (w), 2897 (w), 2818 (w), 1604 (w), 1574 (w), 1557 (w), 1484 (w), 1453 (m), 1435 (w), 1414 (w), 1389 (w), 1276 (w), 1263 (w), 1246 (w), 1214 (w), 1195 (w), 1179 (w), 1157 (w), 1119 (w), 1107 (w), 1078 (s), 1063 (s), 998 (w), 972 (m), 945 (m), 918 (w), 894 (w), 880 (w), 864 (w), 839 (w), 821 (s), 805 (m), 797 (s), 783 (w), 745 (vs), 725 (w), 683 (w), 659 (w), 641 (m), 626 (s), 585 (w). **MS (HR-MALDI-TOF, DCTB):** *m/z* calculated for [M]^+^: 1158.3915, found 1158.3917. ${[\boldsymbol{\alpha}]}_{\boldsymbol{D}}^{\boldsymbol{20}}$ (c = 0.124): 1837°⋅mL⋅dm^-1^⋅g^-1^

**Synthesis of** (*all-M*)*-***[3]cyclorubicene (****7):**

Macrocycle (*all-S*)*-***15** (24.7 mg, 21.3 μmol) and triethylsilane (0.51 mL, 3.20 mmol) were dissolved in dichloromethane (6 mL). Triflic acid (23 μL, 0.26 mmol) was added, and the mixture was stirred for 10 min. The reaction was poured into sodium bicarbonate solution (30 mL) and stirred for an additional 10 mins. The phases were separated, and the aqueous phase was extracted with dichloromethane (3 × 30 mL) and layers combined. Solvent was removed *in vacuo* and the crude product was filtered through a silica pad with dichloromethane (150 mL) and the solvent removed *in vacuo*. The crude product was purified by HPLC (SiO_2_, 65% PhMe/*n*-heptane) to obtain (*all-M*)-**7** as purple solid (13.3 mg, 64%). **M.p.:** >400 °C. **^1^H NMR (600 MHz, CDCl_3_):** *δ* = 8.36 (d, *J* = 8.6, 6H, H‑3), 8.07 (d, *J* = 8.6, 6H, H‑7), 7.92 (d, *J* = 8.7, 6H, H‑8), 7.84-7.80 (m, 12H, H‑1+H‑12), 7.60 (dd, *J* = 8.6, 6.9, 6H, H‑2). **^13^C NMR (151 MHz, CDCl_3_):** *δ* = 141.2 (C‑13), 139.7 (C‑11), 137.5 (C‑6/9), 137.4 (C‑6/9), 134.4 (C‑10), 133.9 (C‑5), 129.2 (C‑2), 127.5 (C‑4), 125.0 (C‑3), 124.7 (C‑7), 124.1 (C‑12), 123.8 (C‑8), 120.4 (C‑1). **IR (ATR):** 3063 (w), 3047 (w), 2957 (w), 2922 (w), 2851 (w), 1598 (w), 1568 (w), 1558 (w), 1519 (w), 1472 (w), 1444 (s), 1435 (m), 1403 (m), 1377 (m), 1320 (m), 1298 (w), 1281 (w), 1259 (w), 1234 (m), 1176 (m), 1158 (m), 1117 (m), 1065 (w), 1037 (m), 903 (m), 886 (w), 873 (m), 856 (m), 818 (s), 802 (m), 761 (m), 731 (vs), 702 (m), 678 (w), 667 (m), 662 (s), 628 (w), 611 (m). **UV-Vis (dichloromethane):** *λ*_max_ (lg ε) = 258 (5.09), 304 (4.69), 344 (4.77), 378 (4.49), 400 (4.38), 488 (4.36), 537 (4.51), 582 (4.44), 670 (3.81) nm.**MS (HR-MALDI-TOF):** *m/z* calculated for [M]^+^: 972.2812, found 972.2815.

**Synthesis of** (*all-P*)*-***[3]cyclorubicene (****7):**

(*all-P*)-**7** was prepared from (*all-R*)-**15** (24.8 mg, 21.4 μmol) according to the procedure for the preparation of (*all-M*)-**7.** (*all-P*)-**7** was obtained as colorless solid (14.1 mg, 68%).

**M.p.:** >400 °C. **^1^H NMR (600 MHz, CDCl_3_):** *δ* = 8.36 (d, *J* = 8.6, 6H, H‑3), 8.07 (d, *J* = 8.6, 6H, H‑7), 7.92 (d, *J* = 8.7, 6H, H‑8), 7.84-7.80 (m, 12H, H‑1+H‑12), 7.60 (dd, *J* = 8.6, 6.9, 6H, H‑2). **^13^C NMR (151 MHz, CDCl_3_):** *δ* = 141.3 (C‑13), 139.9 (C‑11), 137.6 (C‑6/9), 137.6 (C‑6/9), 134.5 (C‑10), 134.1 (C‑5), 129.2 (C‑2), 127.7 (C‑4), 125.2 (C‑3), 124.8 (C‑7), 124.3 (C‑12), 124.0 (C‑8), 120.5 (C‑1). **IR (ATR):** 3063 (w), 3046 (w), 2920 (w), 2849 (vw), 1597 (w), 1560 (w), 1518 (w), 1444 (m), 1400 (w), 1377 (m), 1320 (w), 1298 (w), 1261 (w), 1234 (w), 1175 (w), 1157 (w), 1116 (w), 1036 (w), 914 (w), 883 (w), 872 (m), 856 (m), 818 (s), 801 (m), 760 (s), 732 (vs), 702 (m), 677 (m), 662 (s), 628 (m), 611 (m), 585 (w). **UV-Vis (dichloromethane):** *λ*_max_ (lg ε) = 258 (5.09), 304 (4.69), 344 (4.77), 378 (4.49), 400 (4.38), 488 (4.36), 537 (4.51), 582 (4.44), 670 (3.81) nm. **MS (HR-MALDI-TOF):** *m/z* calculated for [M]^+^: 972.2812, found 972.2814.

# Spectra

## ^1^H NMR and ^13^C NMR spectra


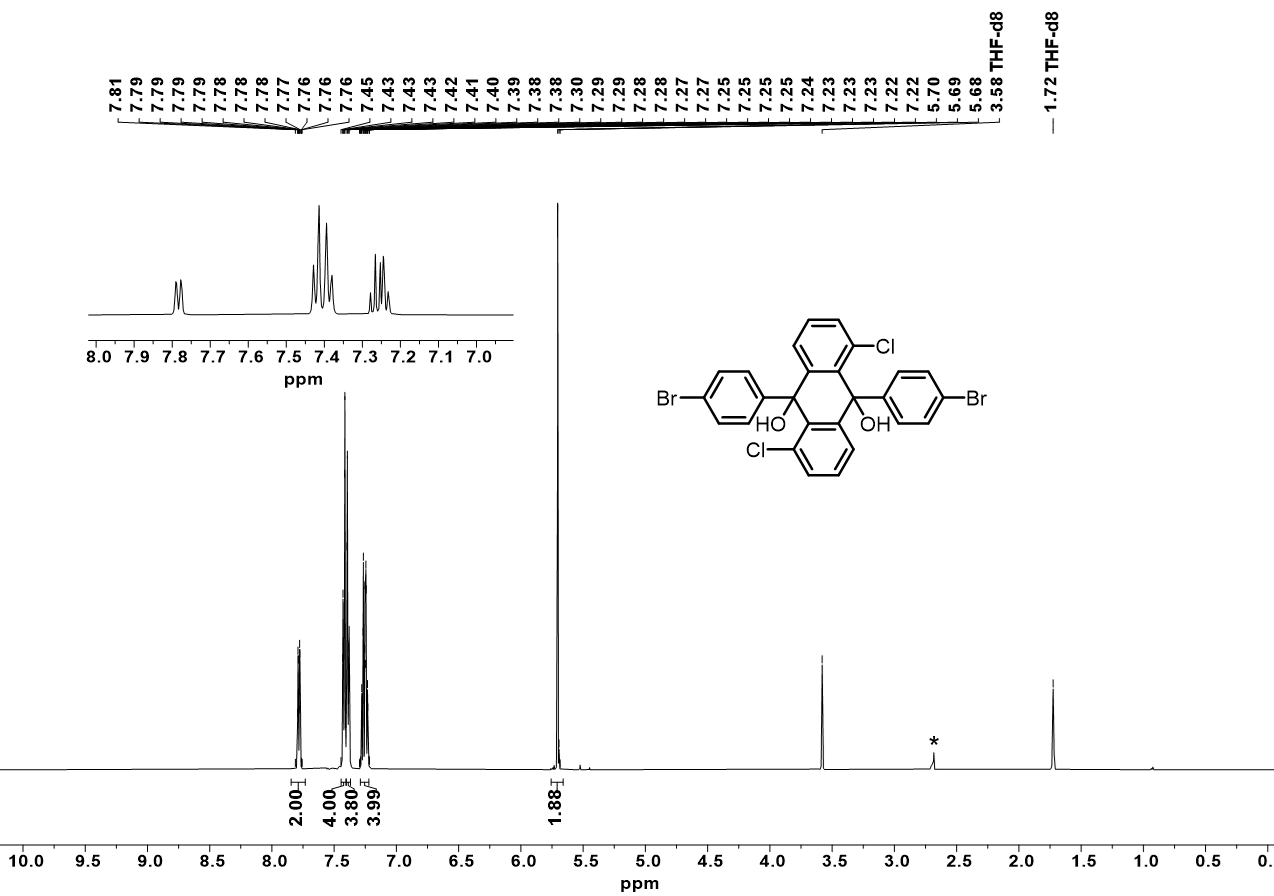


**Figure S2:** ^1^H NMR spectrum of **10** (THF-d_8_, 600 MHz, 300K). *water


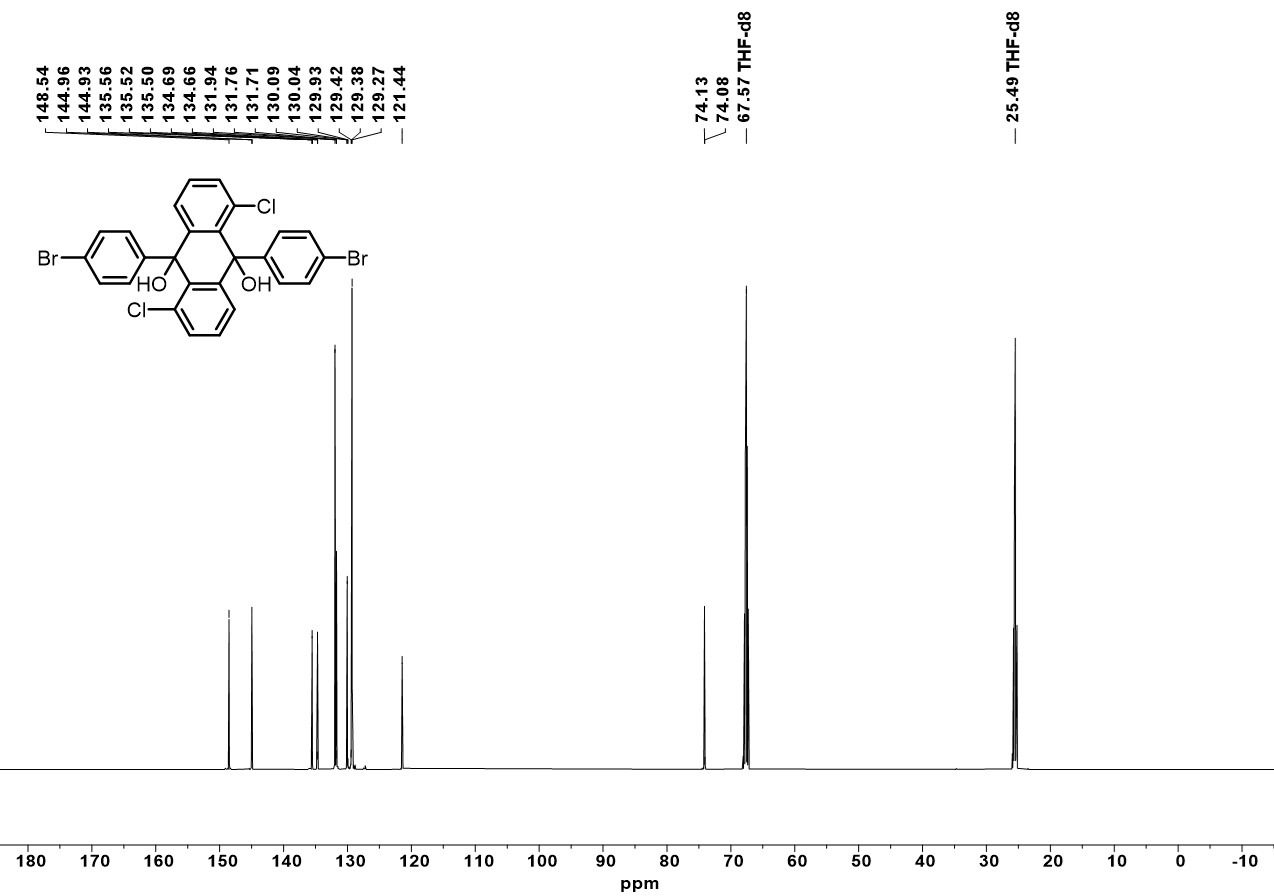


**Figure S3:** ^13^C NMR spectrum of **10** (THF-d_8_, 151 MHz, 300K).


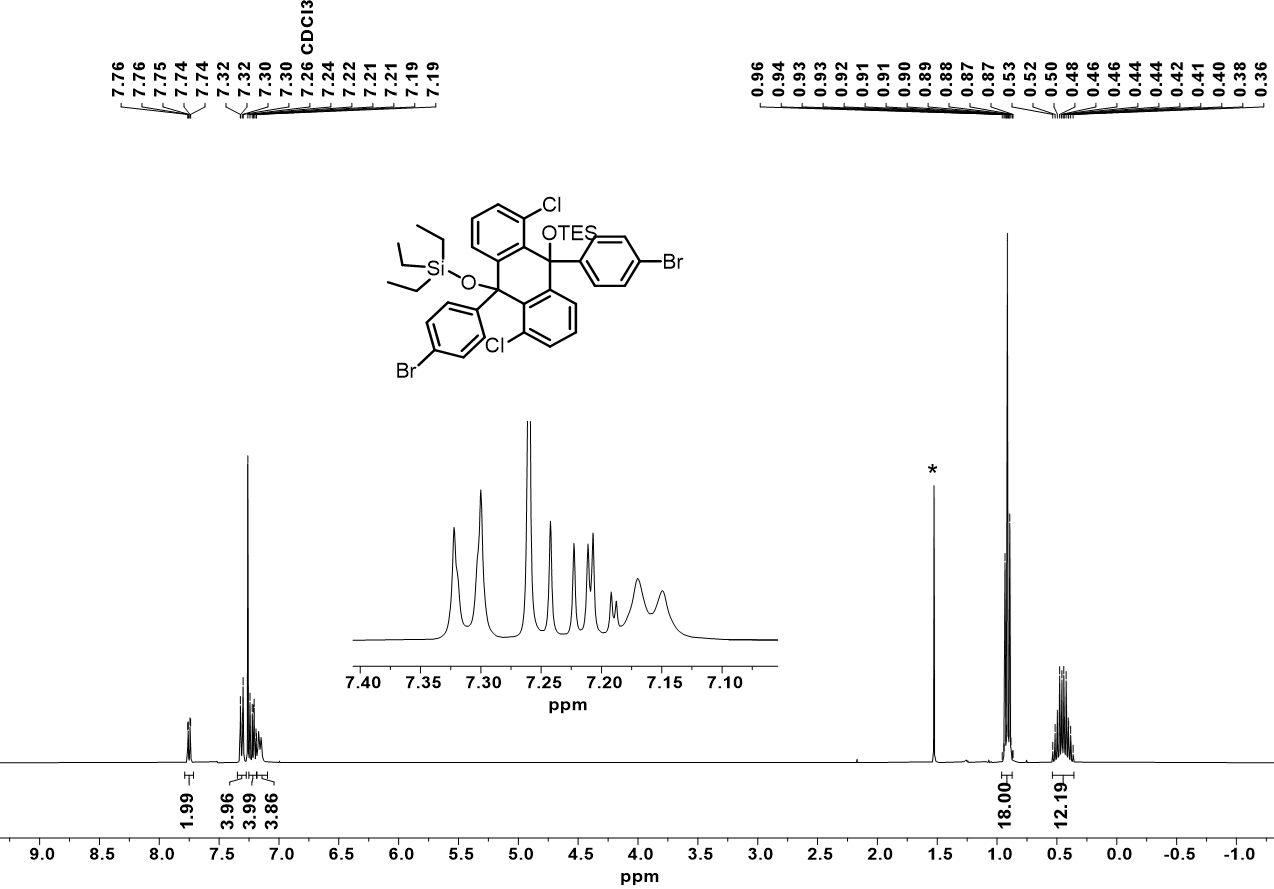


**Figure S4:** ^1^H NMR spectrum of **11** (CDCl_3_, 400 MHz, 300K). *water


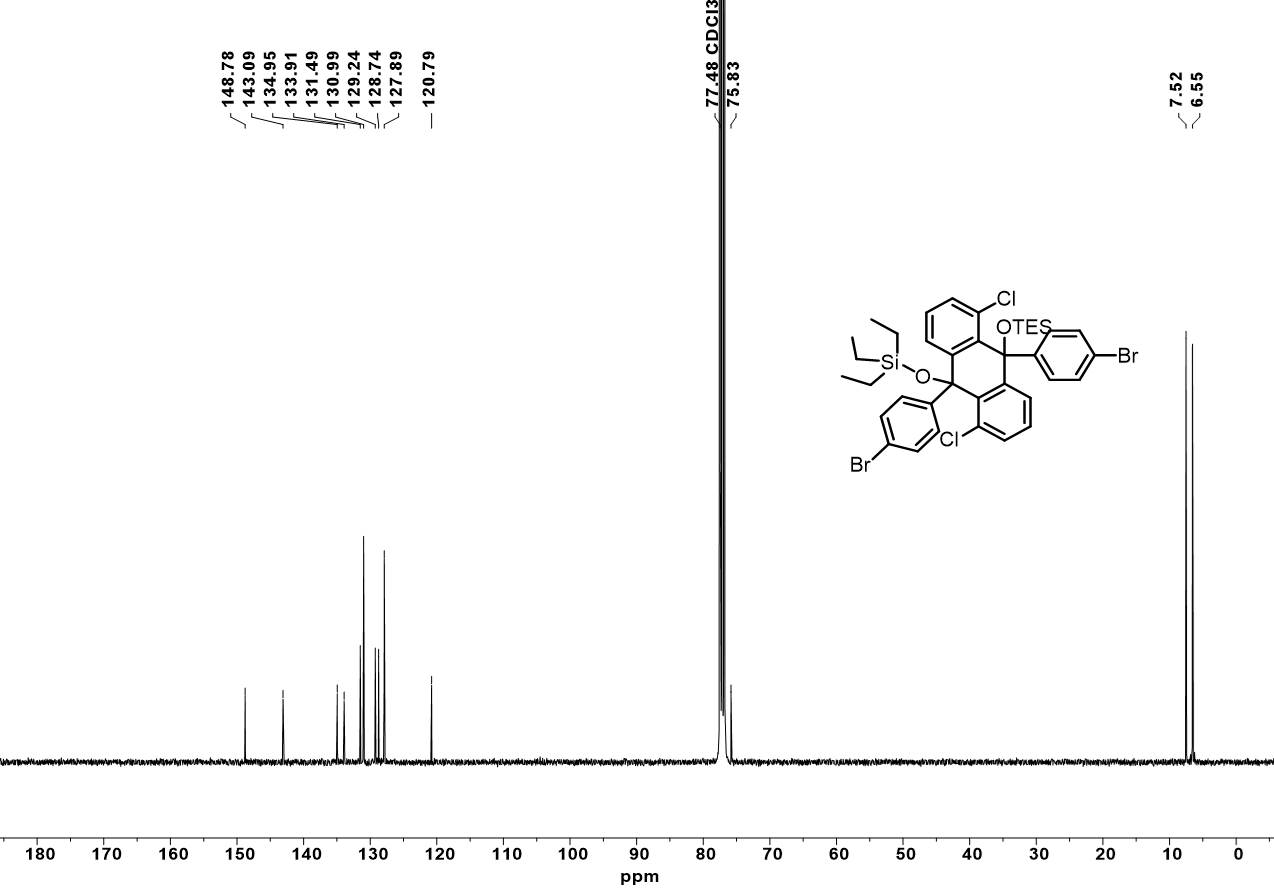


**Figure S5:** ^13^C NMR spectrum of **11** (CDCl_3_, 101 MHz, 300K).


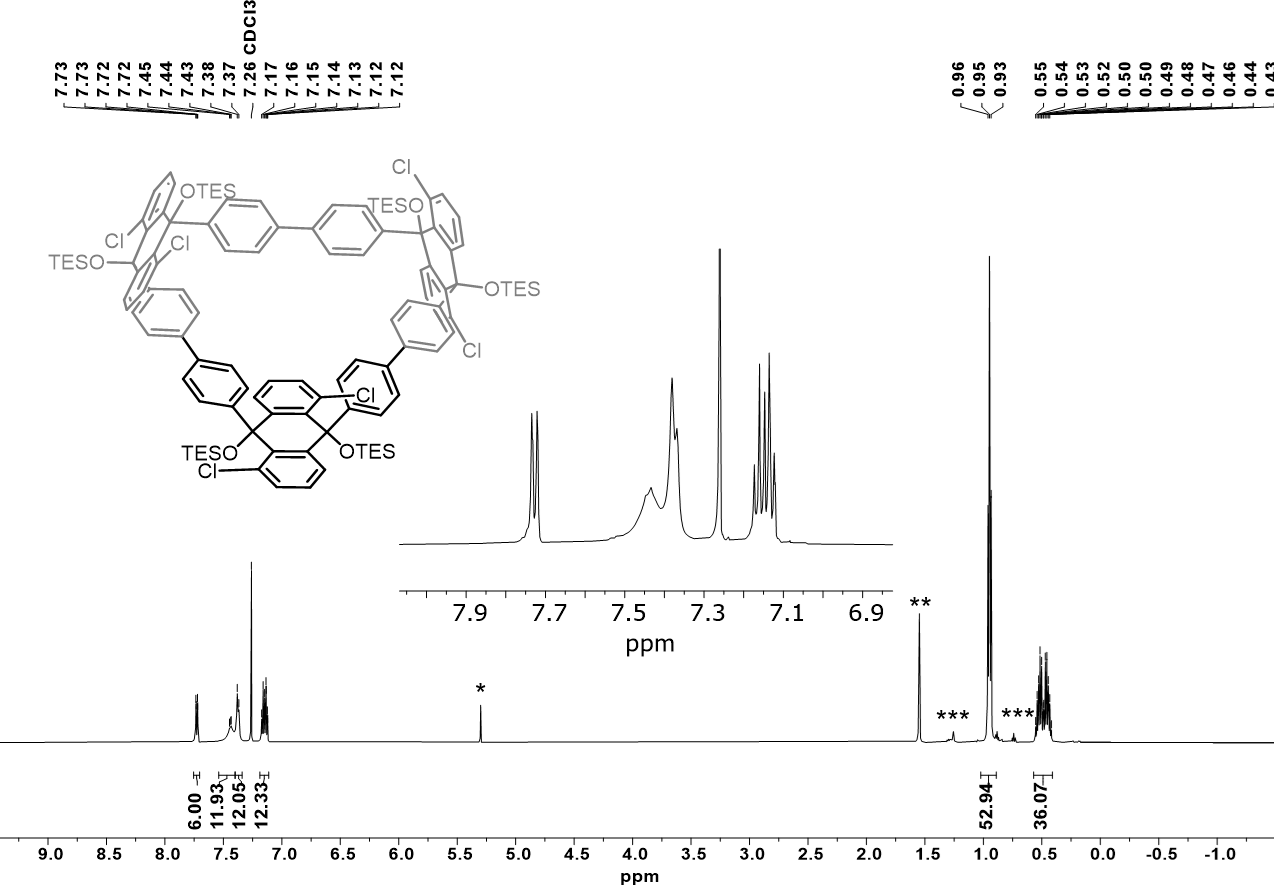


**Figure S6:** ^1^H NMR spectrum of (all-S)-**12** (CDCl_3_, 600 MHz, 300K). *DCM **water ***n-pentane


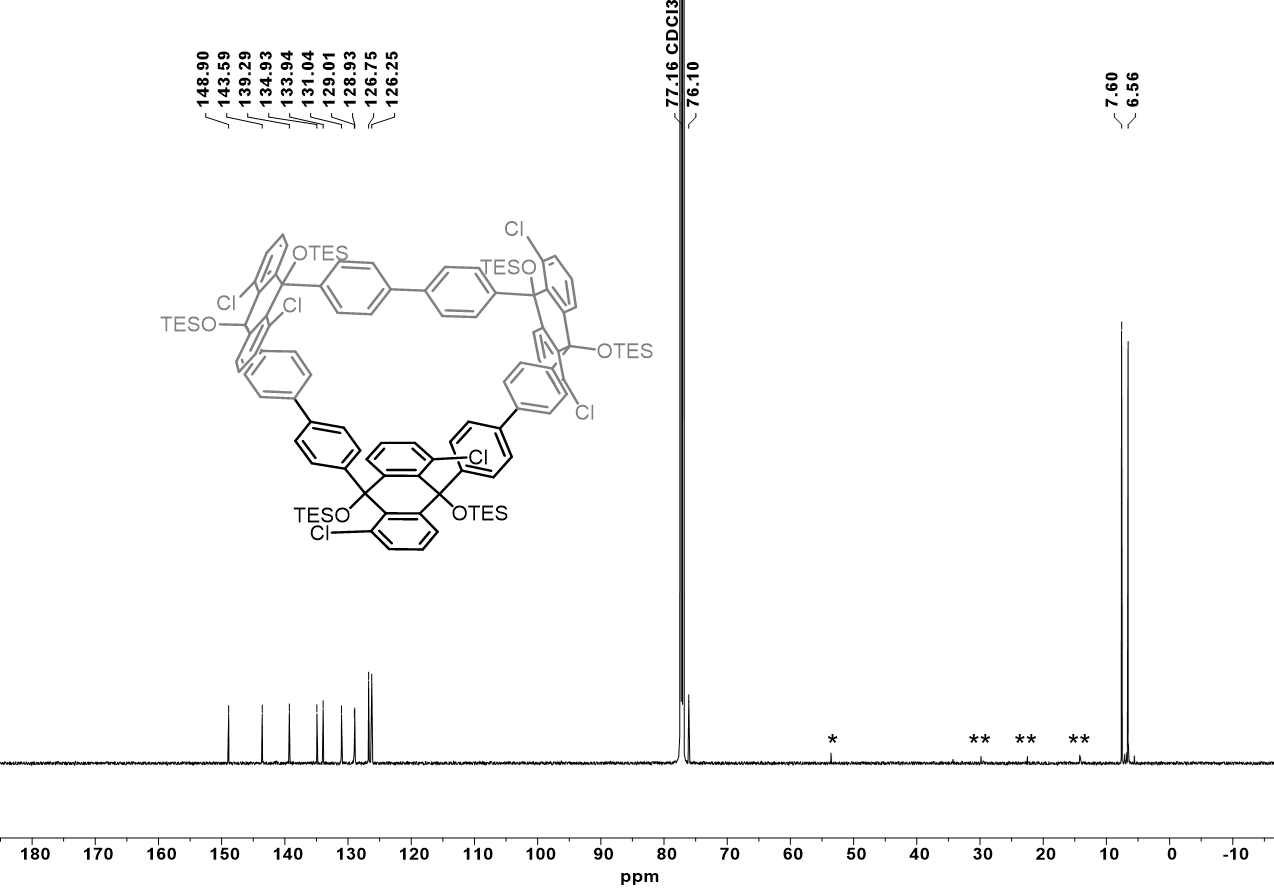


**Figure S7:** ^13^C NMR spectrum of (all-S)-**12** (CDCl_3_, 151 MHz, 300K). *DCM ** n-pentane


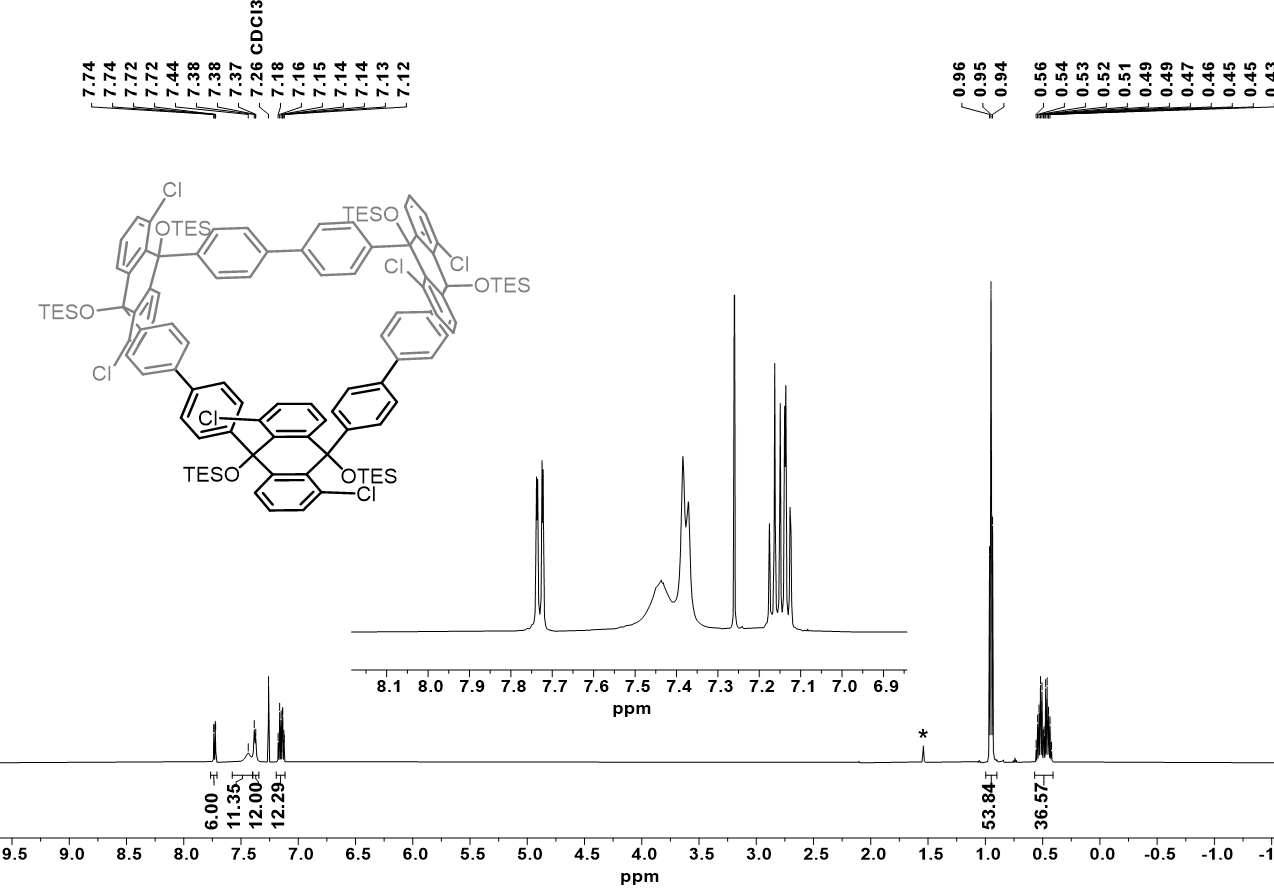


**Figure S8:** ^1^H NMR spectrum of (all-R)-**12** (CDCl_3_, 600 MHz, 300K). *water


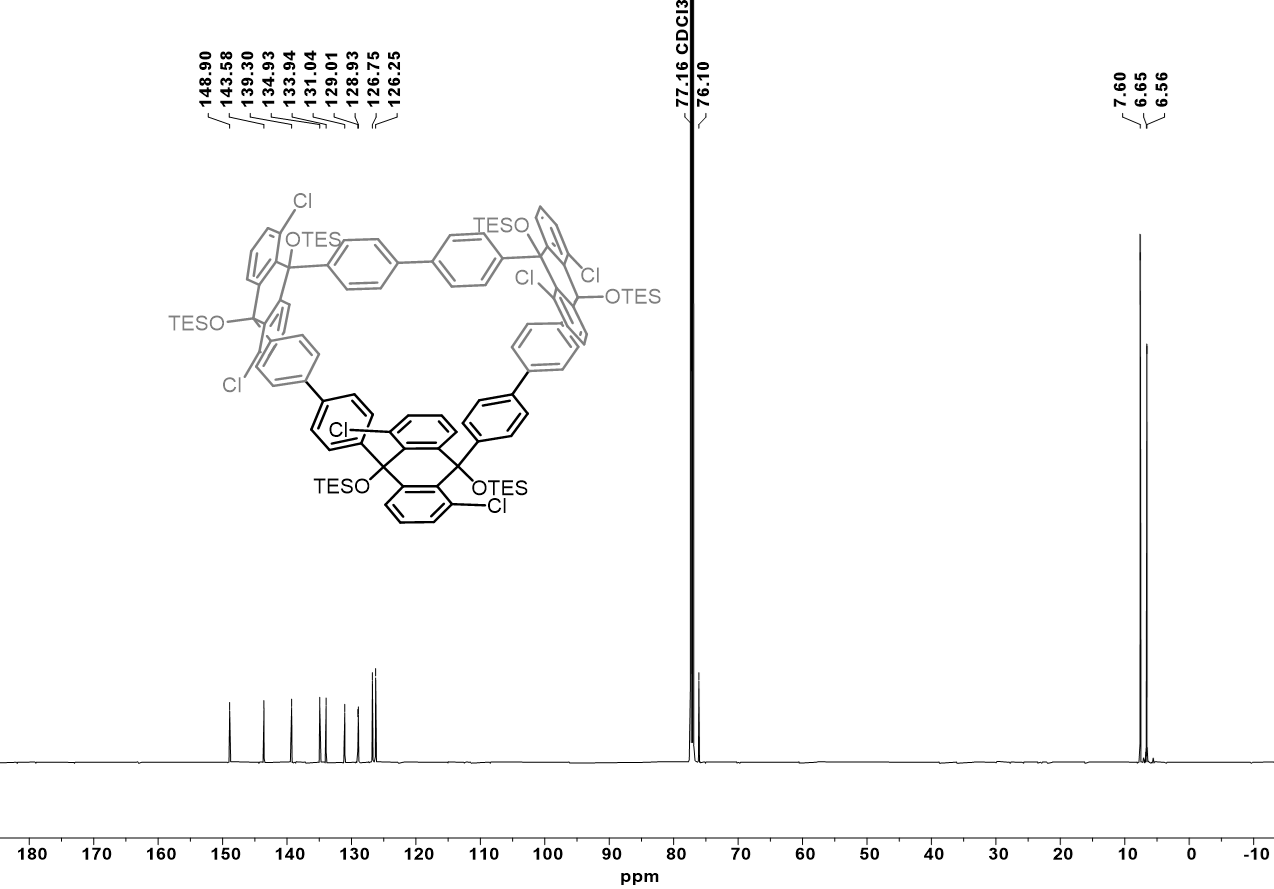


**Figure S9:** ^13^C NMR spectrum of (all-R)-**12** (CDCl_3_, 151 MHz, 300K).


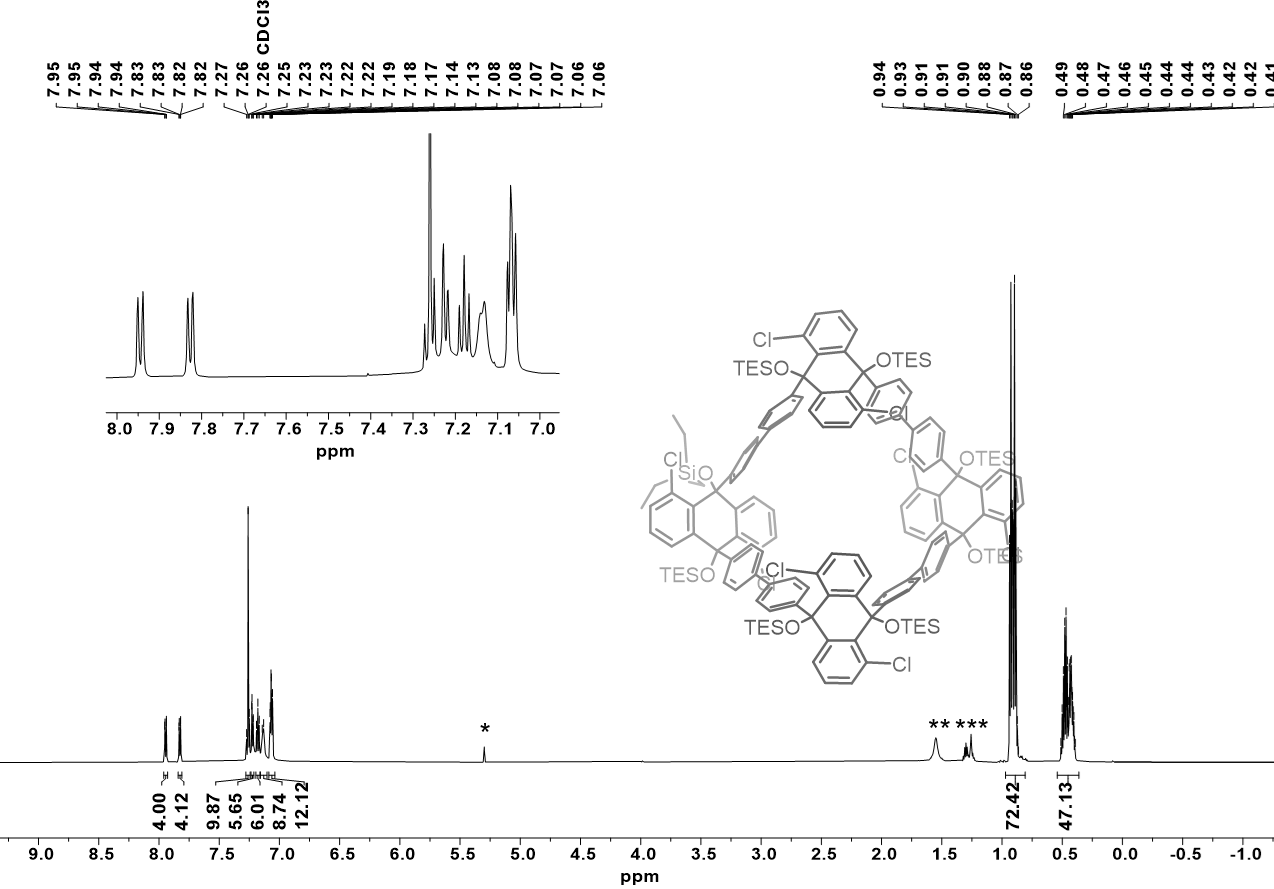


**Figure S10:** ^1^H NMR spectrum of (all-S)-**14** (CDCl_3_, 700 MHz, 300K). *DCM **water *** n-pentane


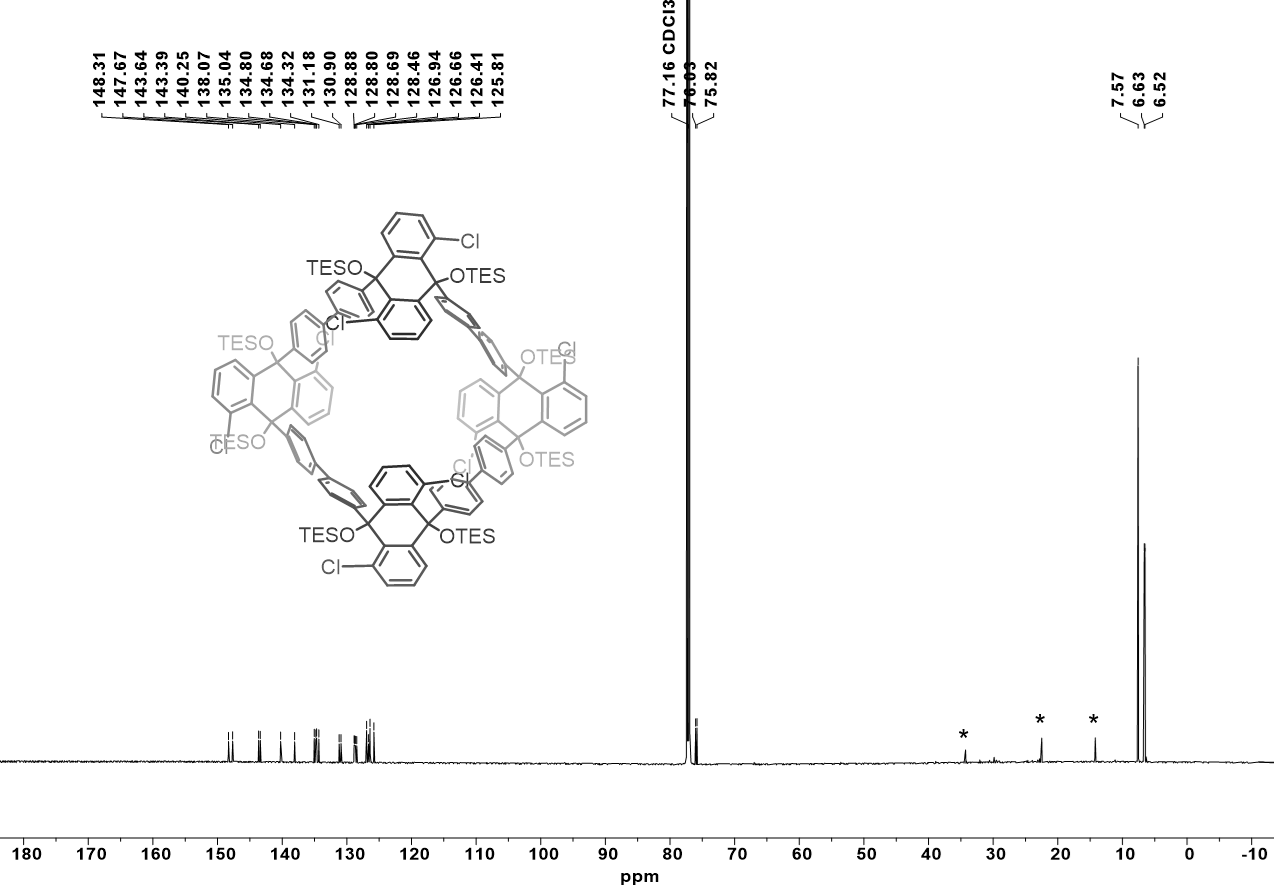


**Figure S11:** ^13^C NMR spectrum of (all-S)-**14** (CDCl_3_, 176 MHz, 300K). * n-pentane


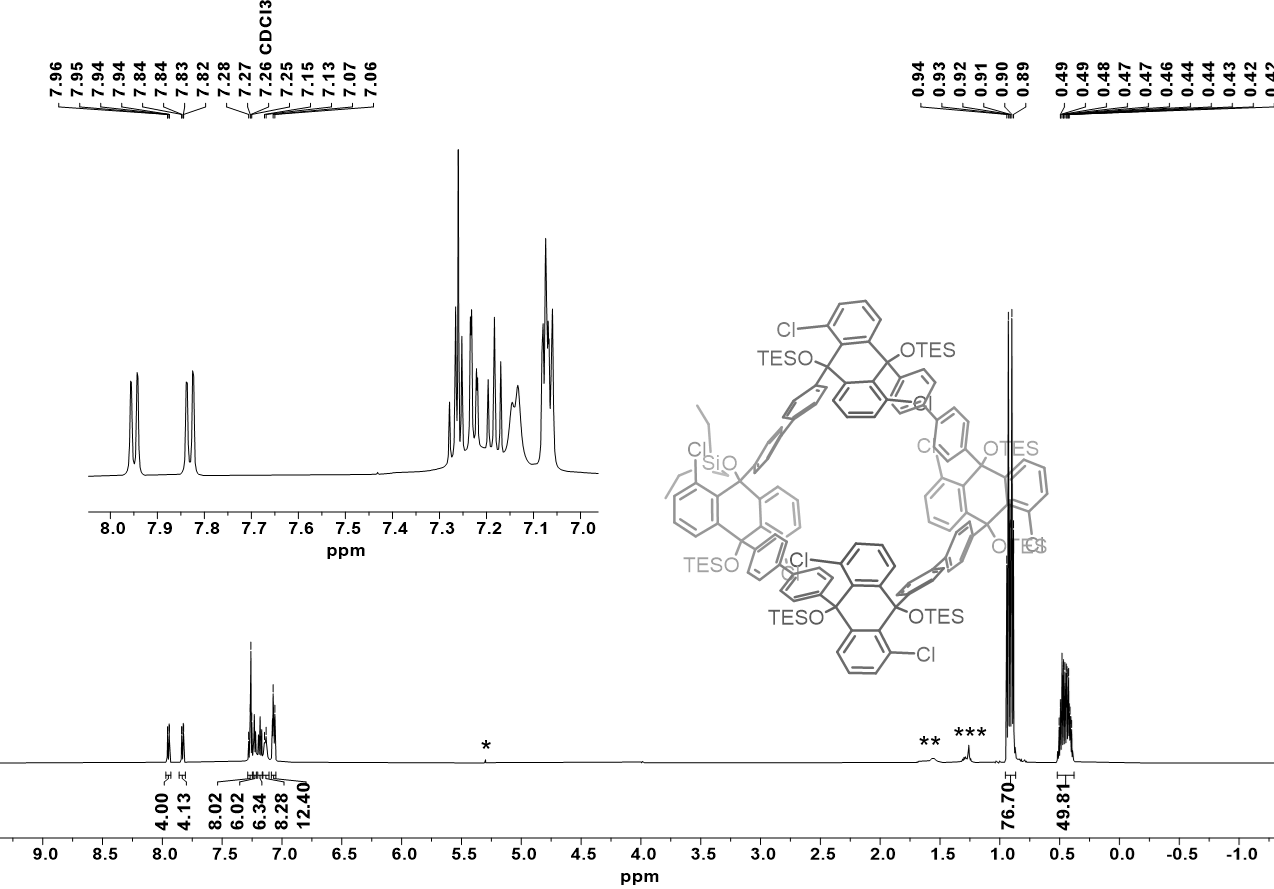


**Figure S12:** ^1^H NMR spectrum of (all-R)-**14** (CDCl_3_, 600 MHz, 300K). *DCM **water *** n-pentane


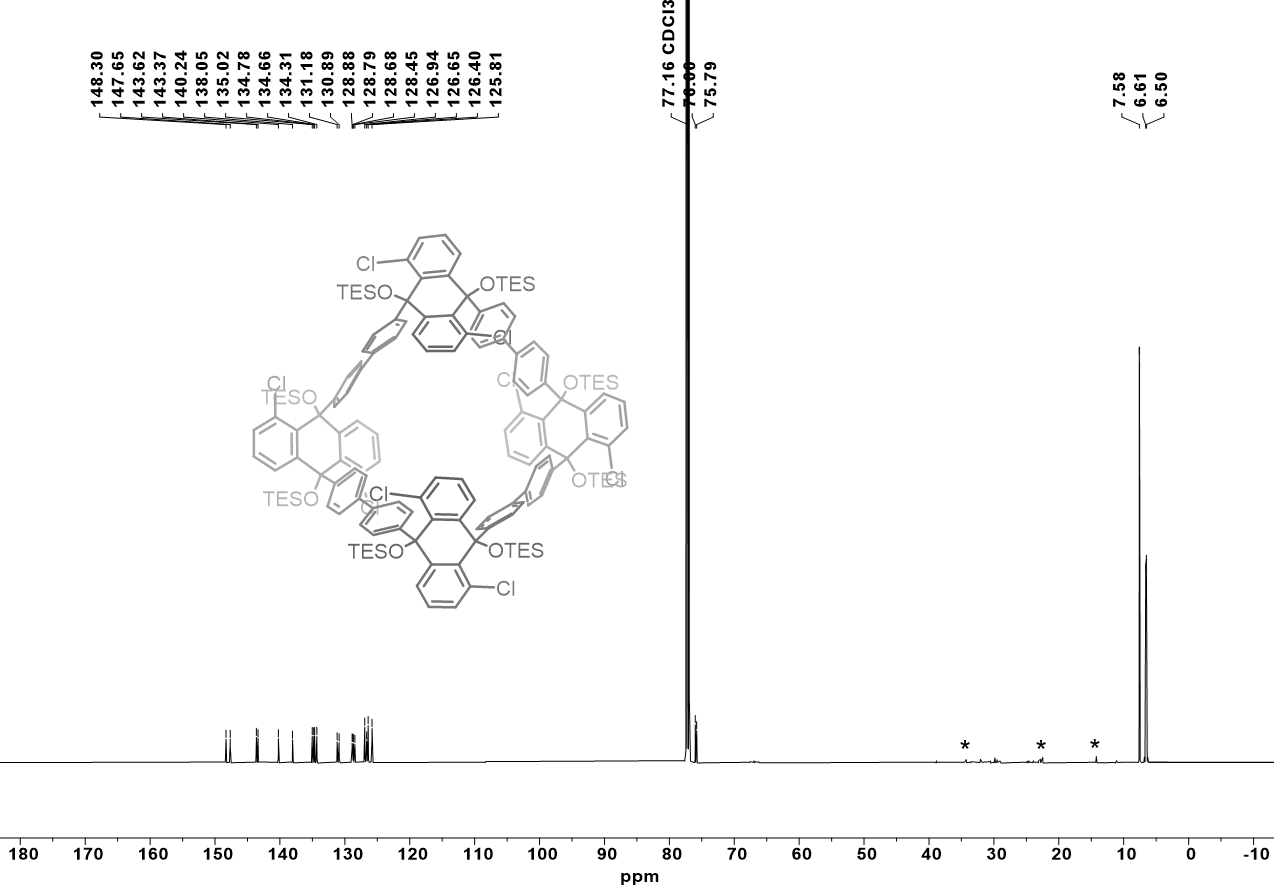


**Figure S13:** ^13^C NMR spectrum of (all-R)-**14** (CDCl_3_, 151 MHz, 300K). * n-pentane


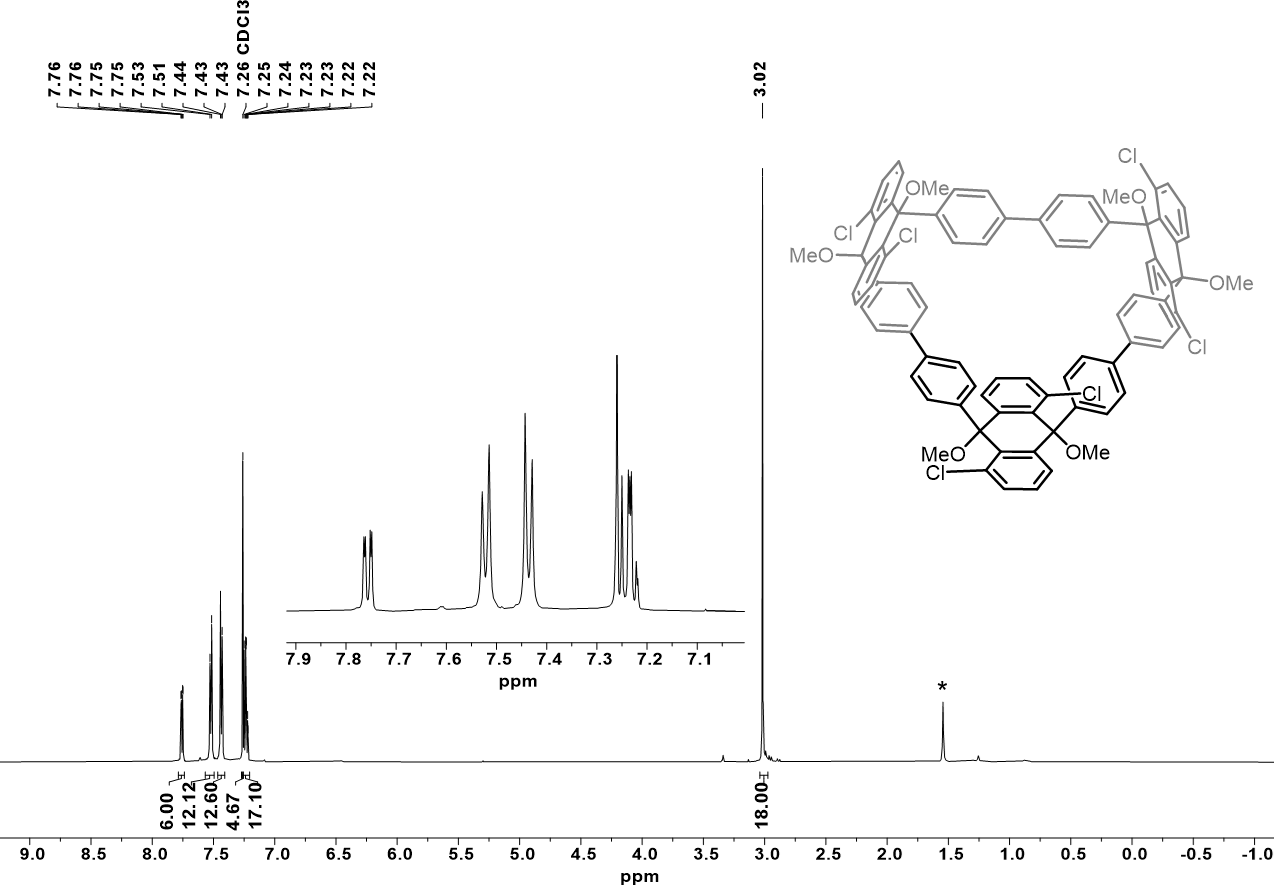


**Figure S14:** ^1^H NMR spectrum of (all-S)-**13** (CDCl_3_, 600 MHz, 300K). *water


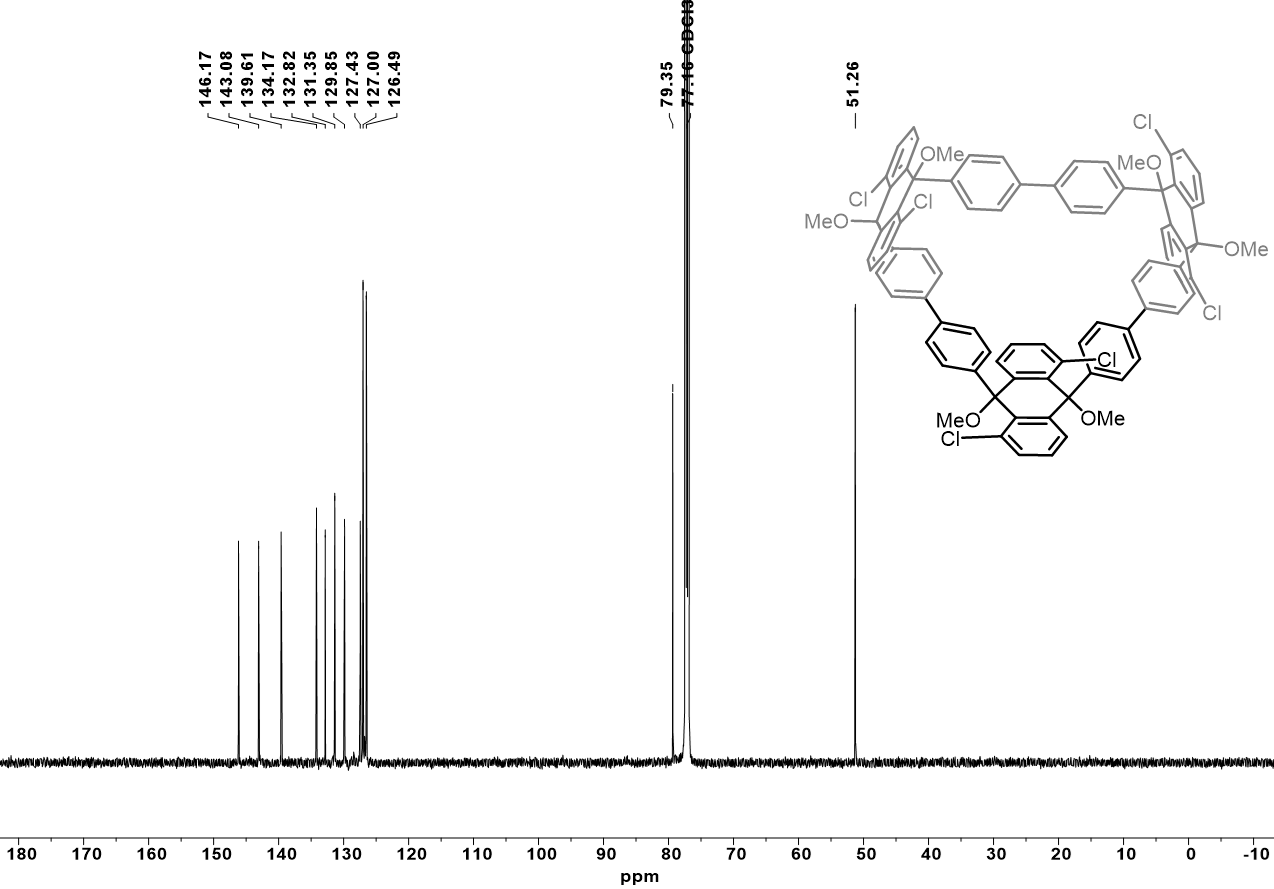


**Figure S15:** ^13^C NMR spectrum of (all-S)-**13** (CDCl_3_, 151 MHz, 300K).


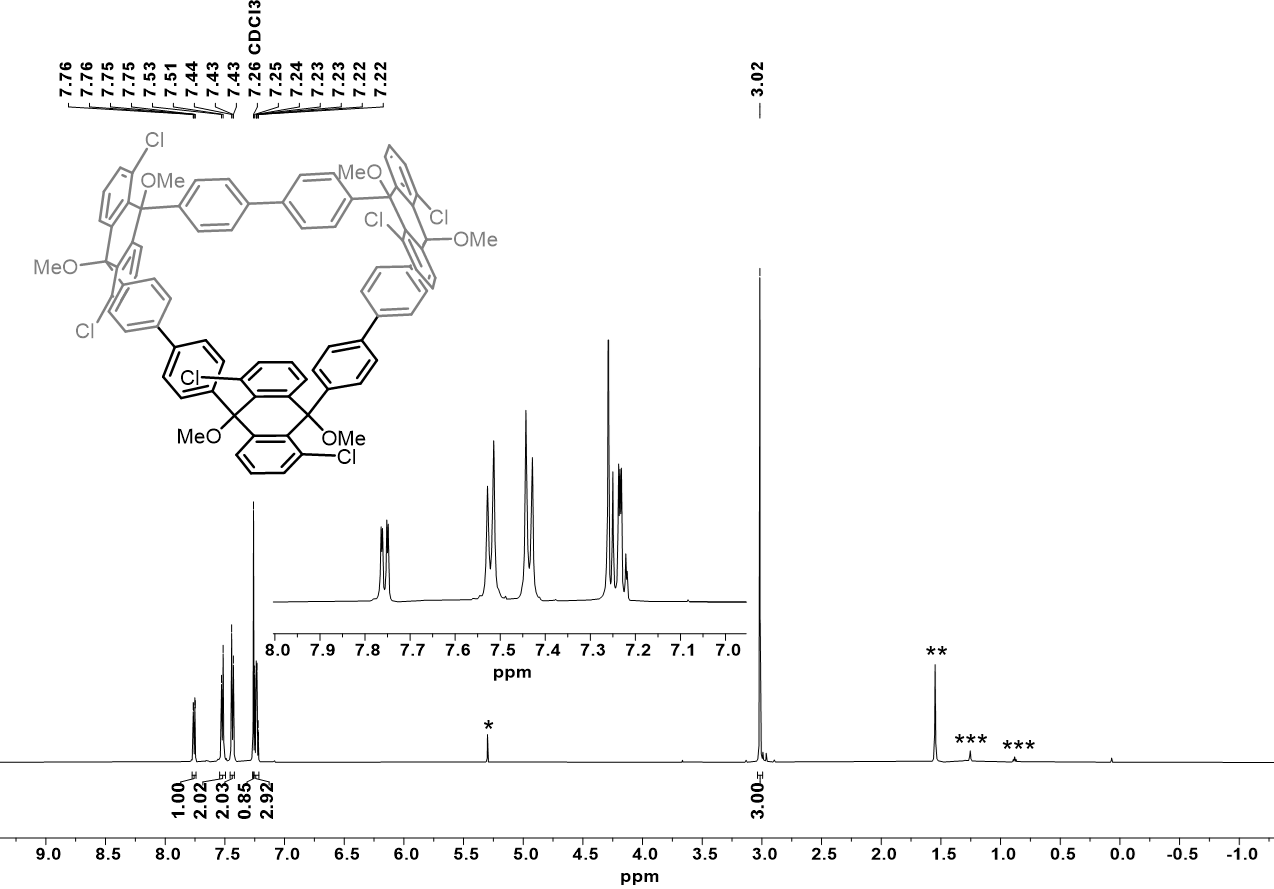


**Figure S16:** ^1^H NMR spectrum of (all-R)-**13** (CDCl_3_, 600 MHz, 300K). *DCM **water *** n-pentane


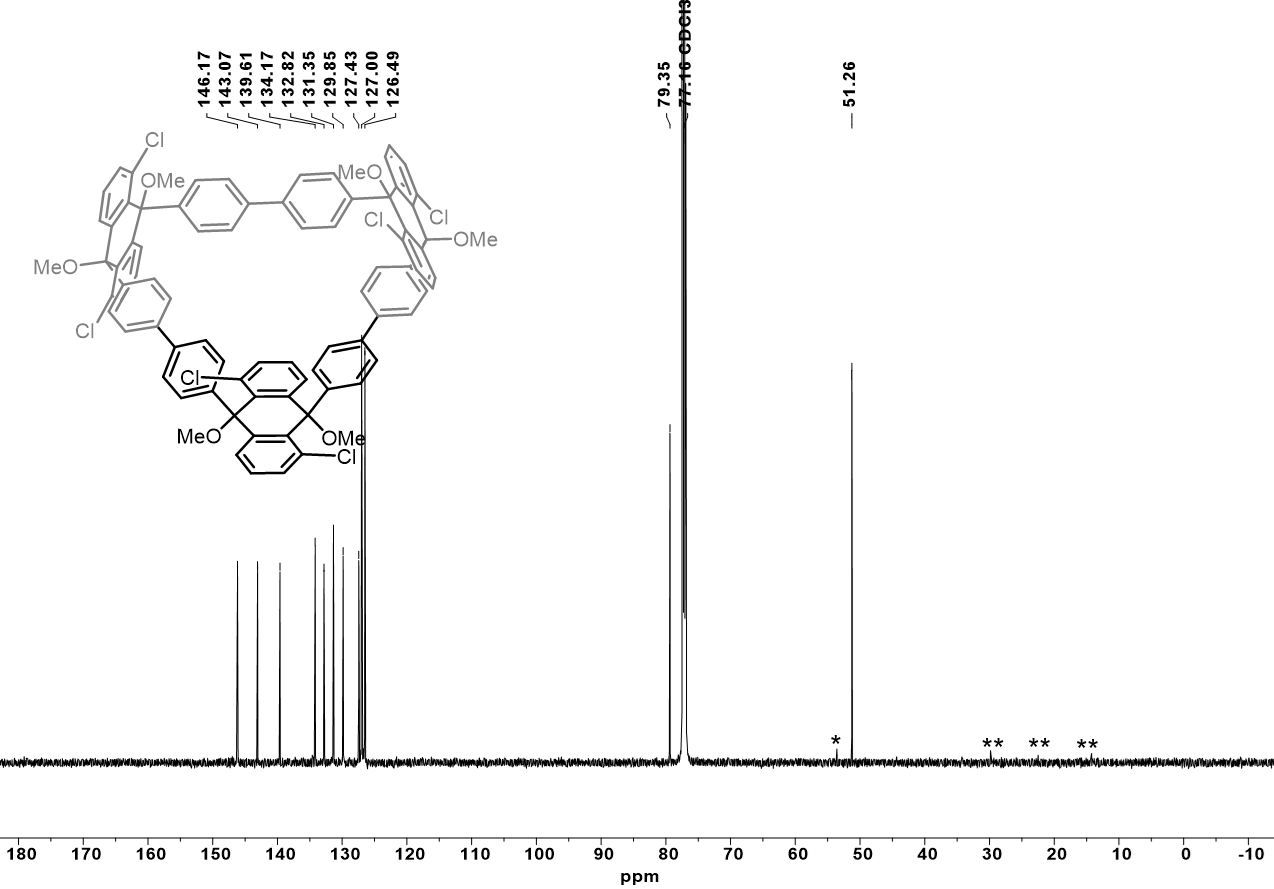


**Figure S17:** ^13^C NMR spectrum of (all-R)-**13** (CDCl_3_, 151 MHz, 300K). *DCM ** n-pentane


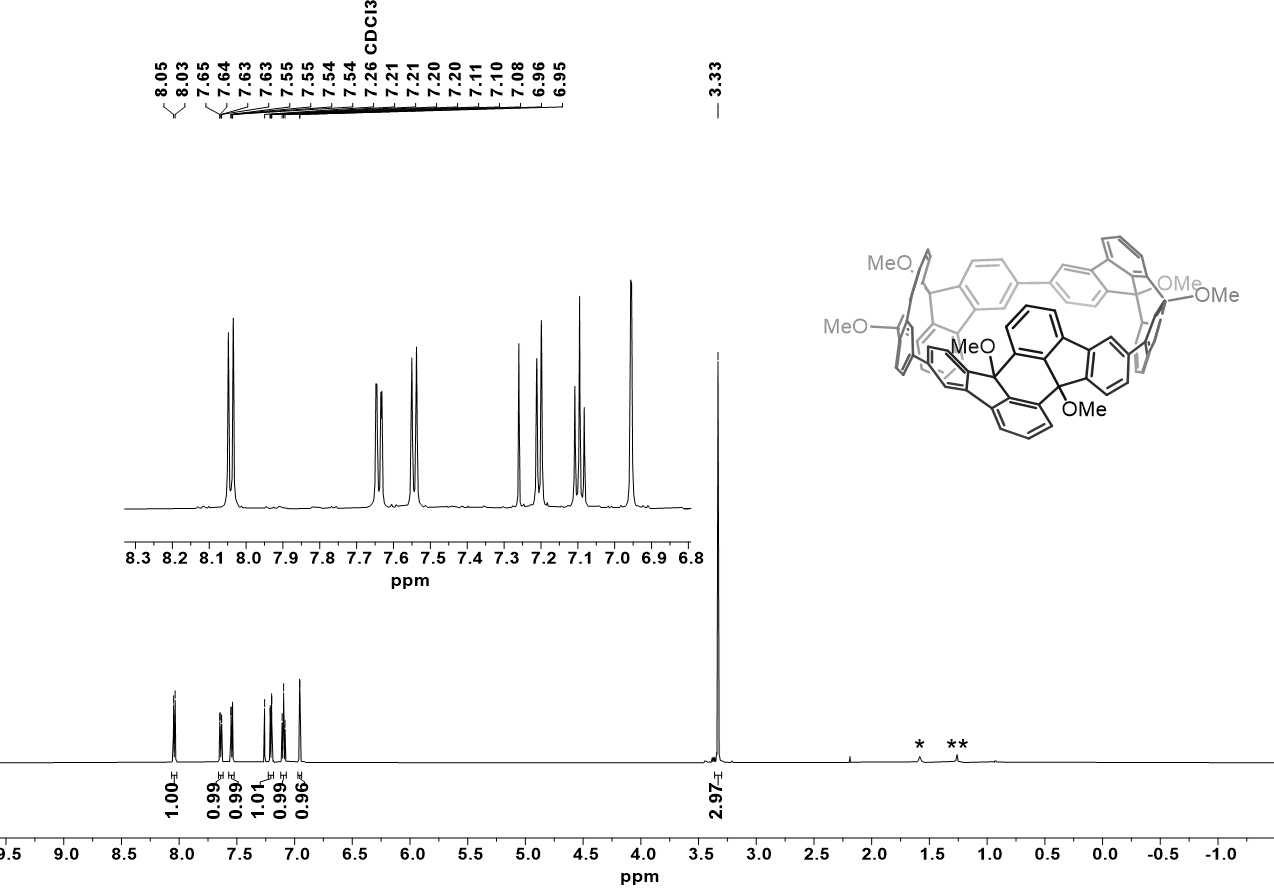


**Figure S18:** ^1^H NMR spectrum of (all-S)-**15** (CDCl_3_, 600 MHz, 300K). *water ** n-pentane


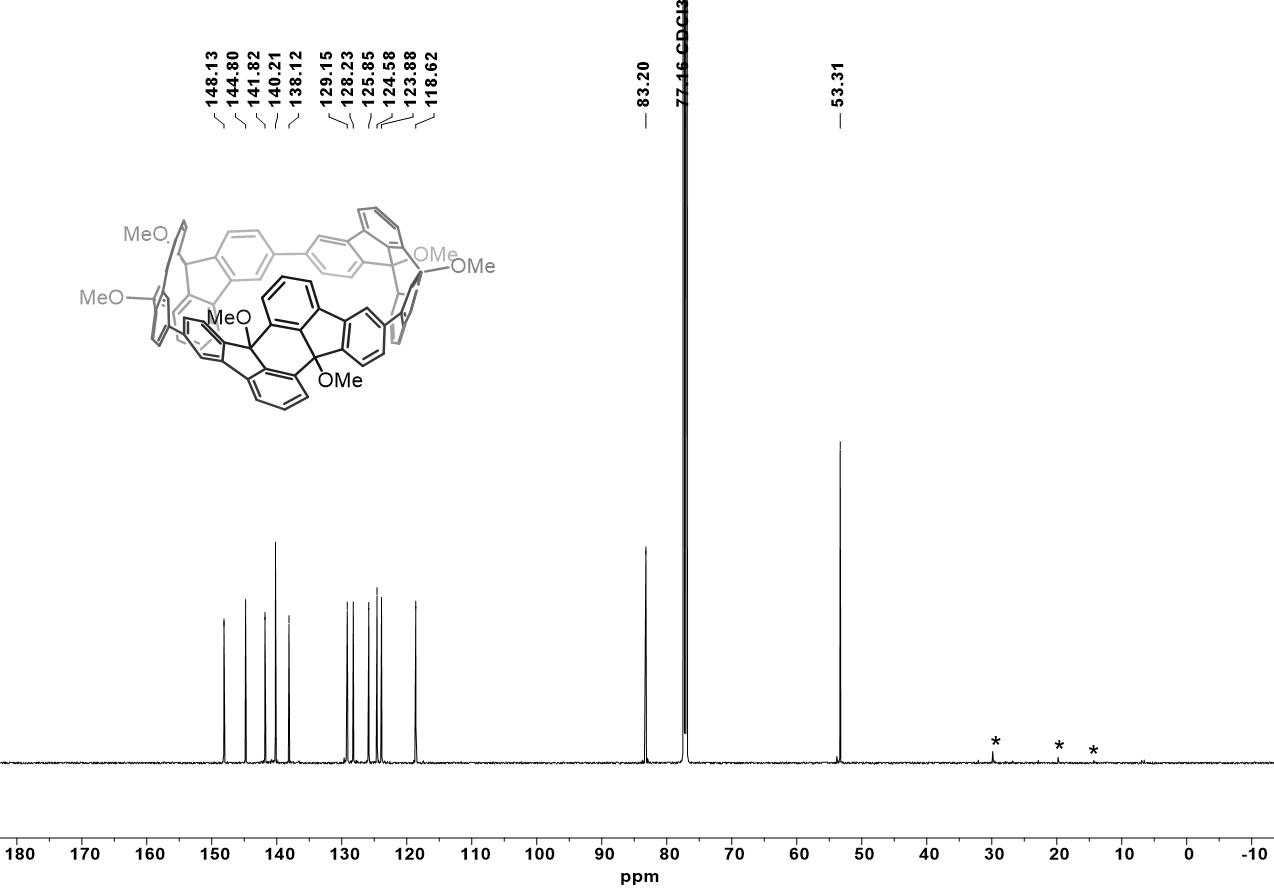


**Figure S19:** ^13^C NMR spectrum of (all-S)-**15** (CDCl_3_, 151 MHz, 300K). *n-pentane


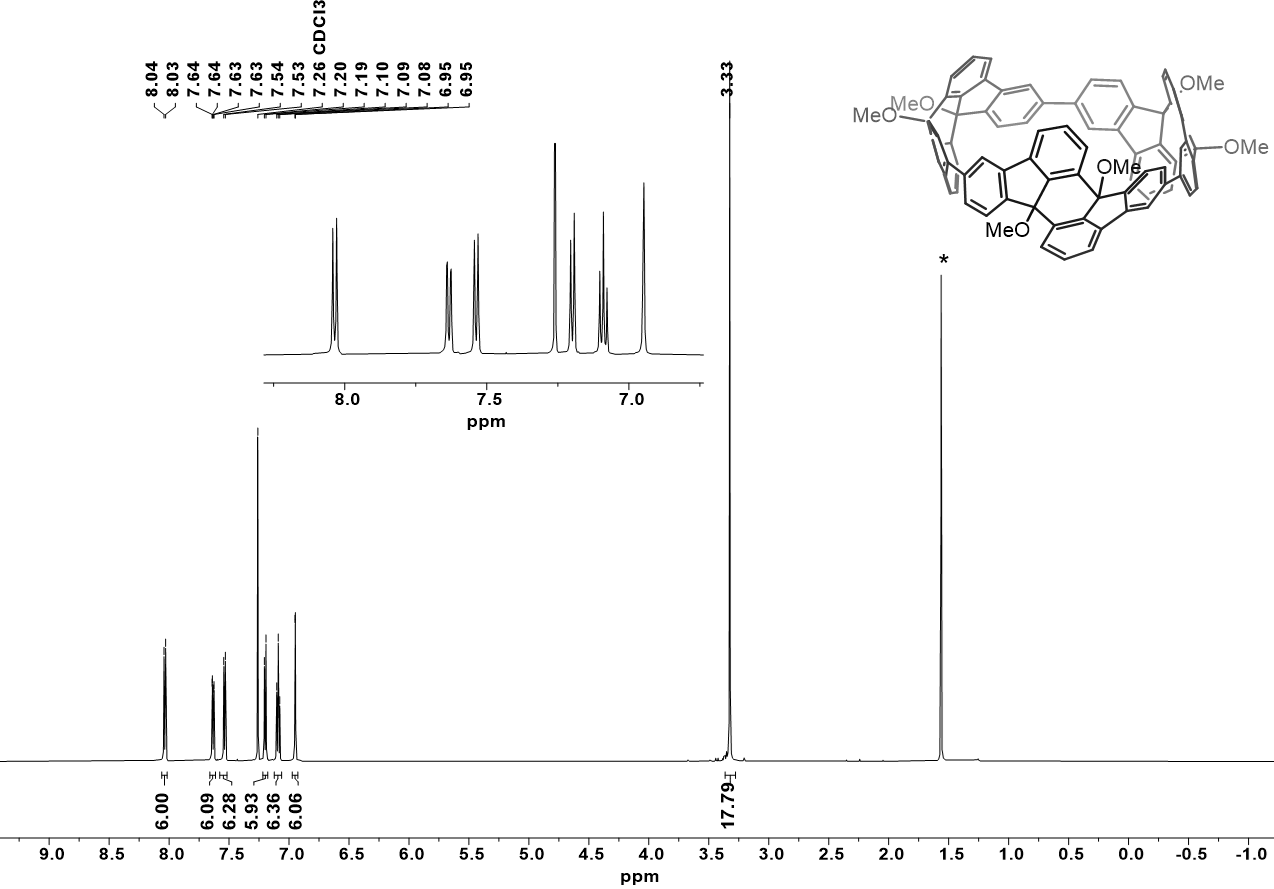


**Figure S20:** ^1^H NMR spectrum of (all-R)-**15** (CDCl_3_, 600 MHz, 300K). *water


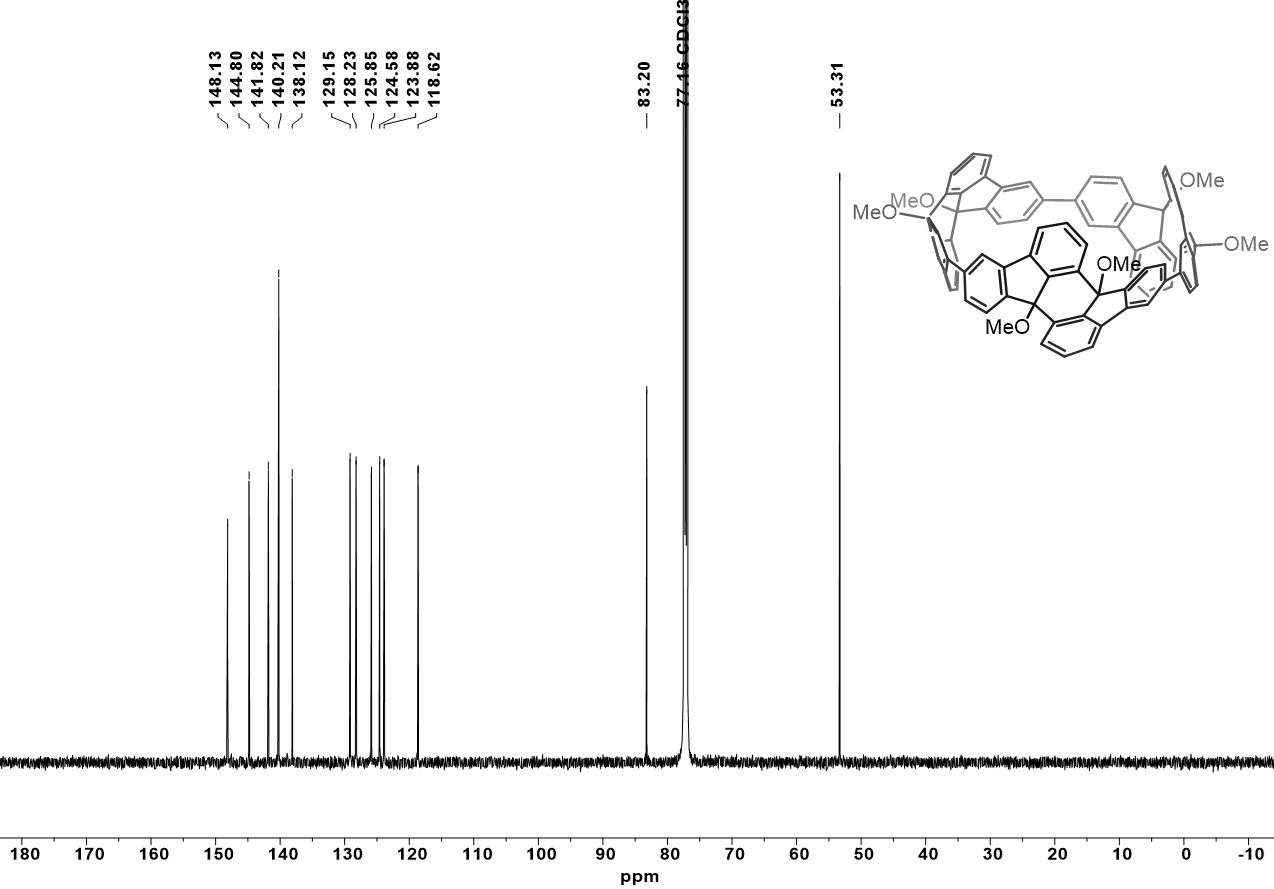


**Figure S21:** ^13^C NMR spectrum of (all-R)-**15** (CDCl_3_, 151 MHz, 300K).


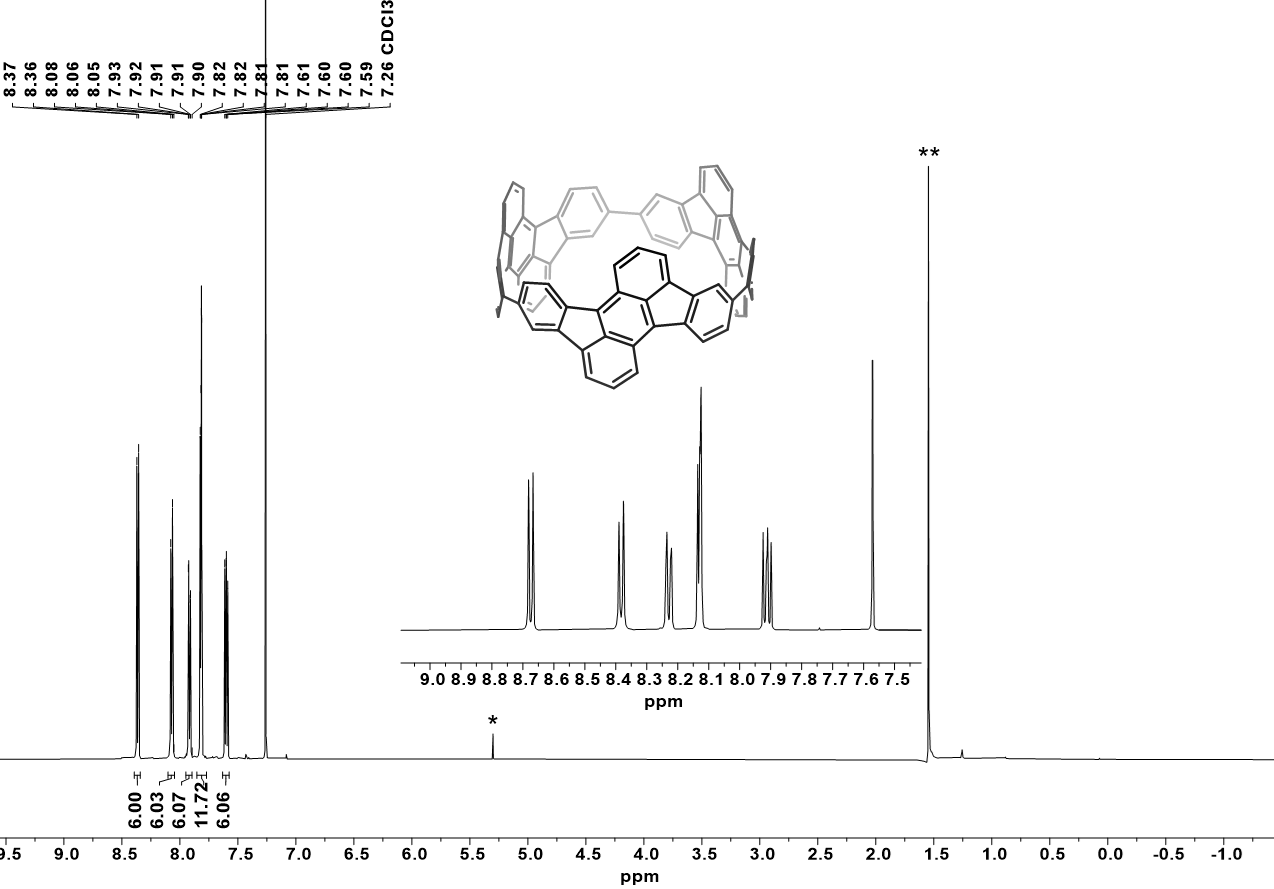


**Figure S22:** ^1^H NMR spectrum of (all-M)-**7** (CDCl_3_, 600 MHz, 300K). *DCM **water


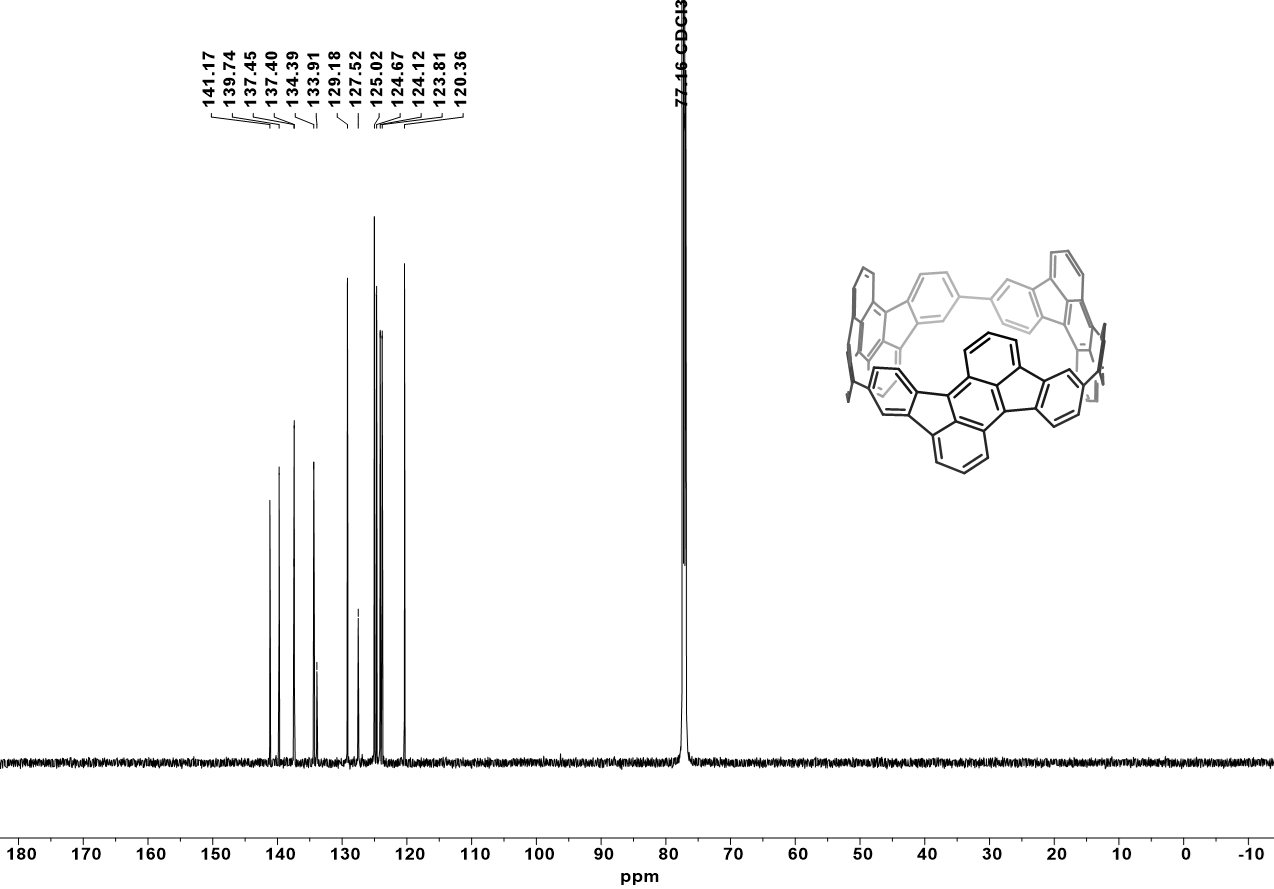


**Figure S23:** ^13^C NMR spectrum of (all-M)-**7** (CDCl_3_, 151 MHz, 300K).


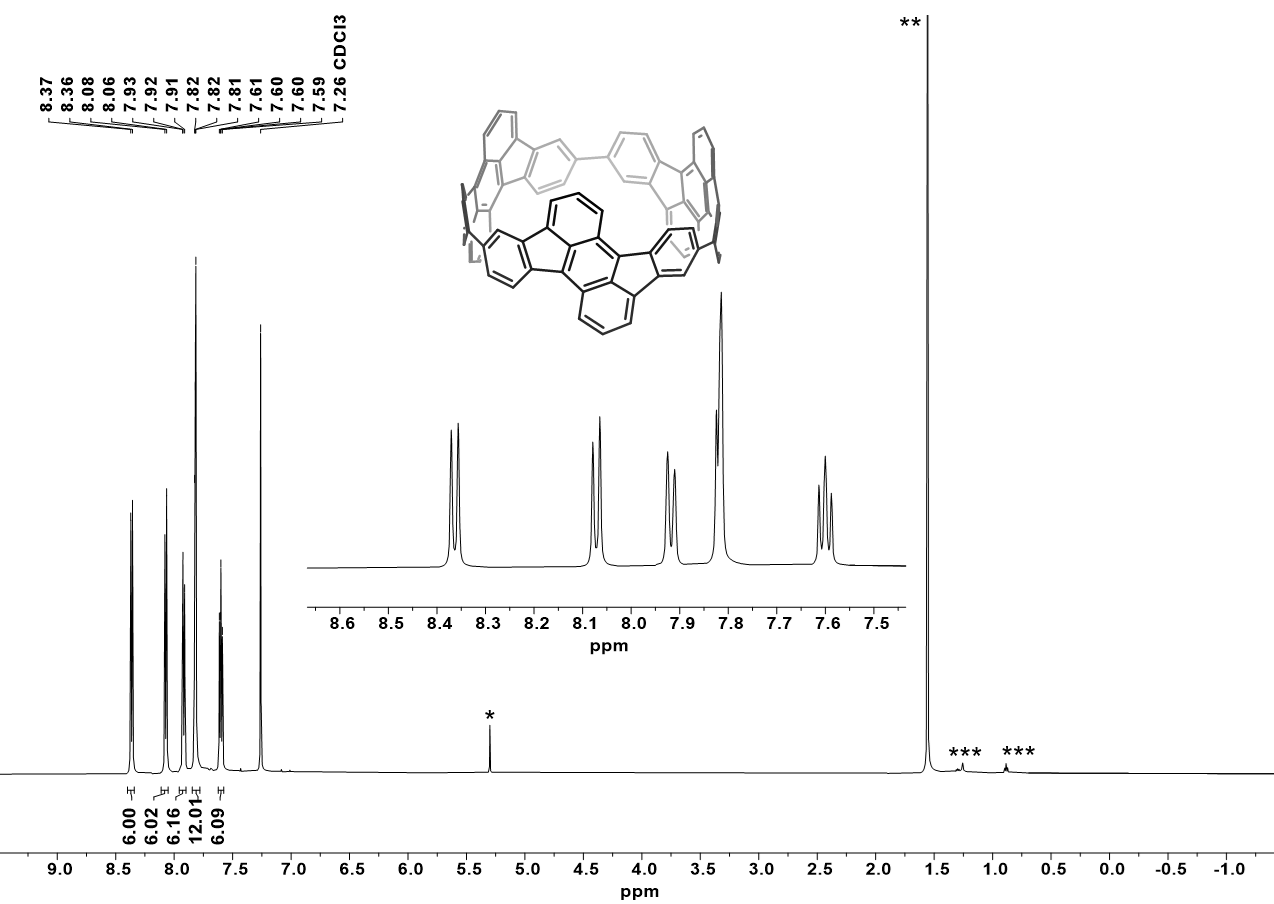


**Figure S24:** ^1^H NMR spectrum of (all-P)-**7** (CDCl_3_, 600 MHz, 300K). *dichloromethane **water *** n-pentane


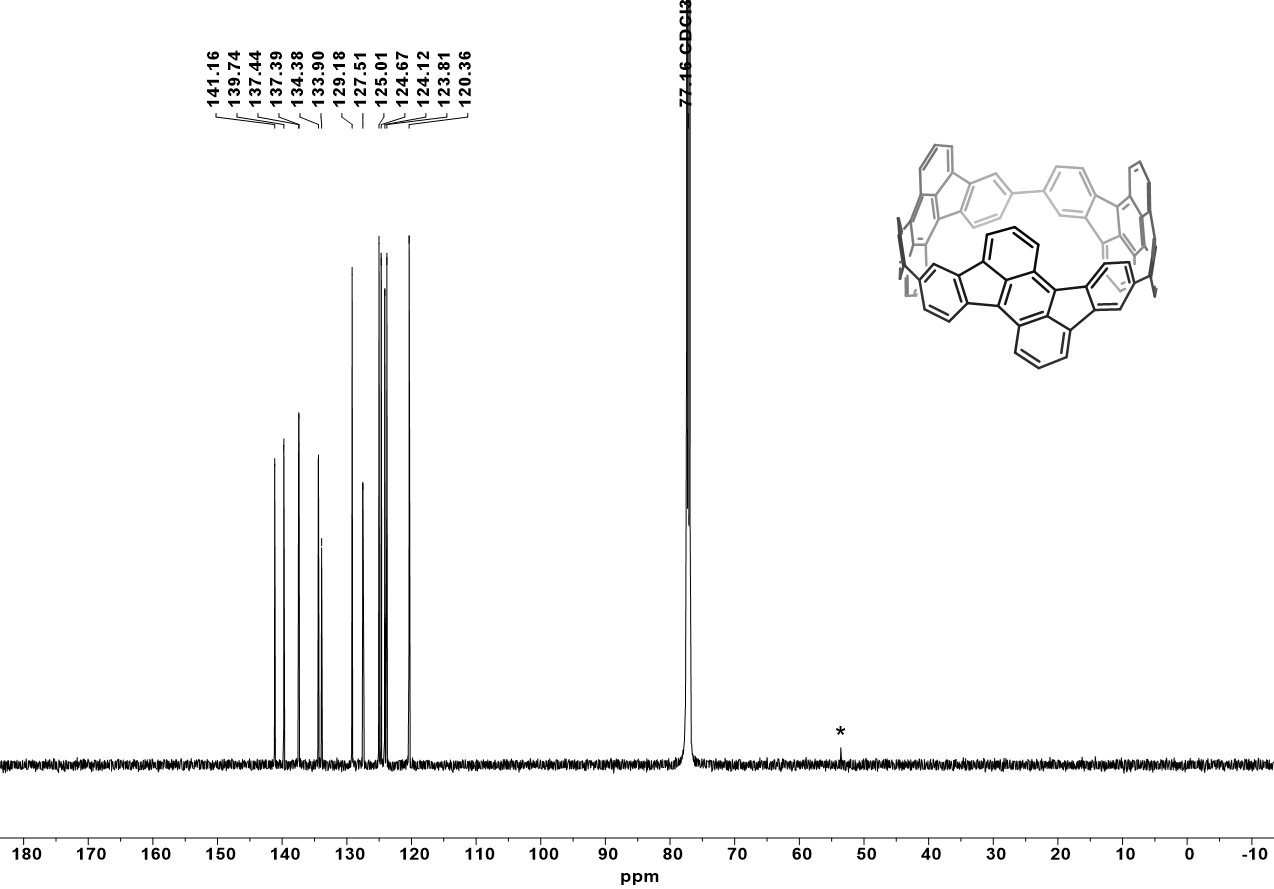


**Figure S25:** ^13^C NMR spectrum of (all-P)-**7** (CDCl_3_, 151 MHz, 300K). *dichloromethane

## 2D NMR spectra


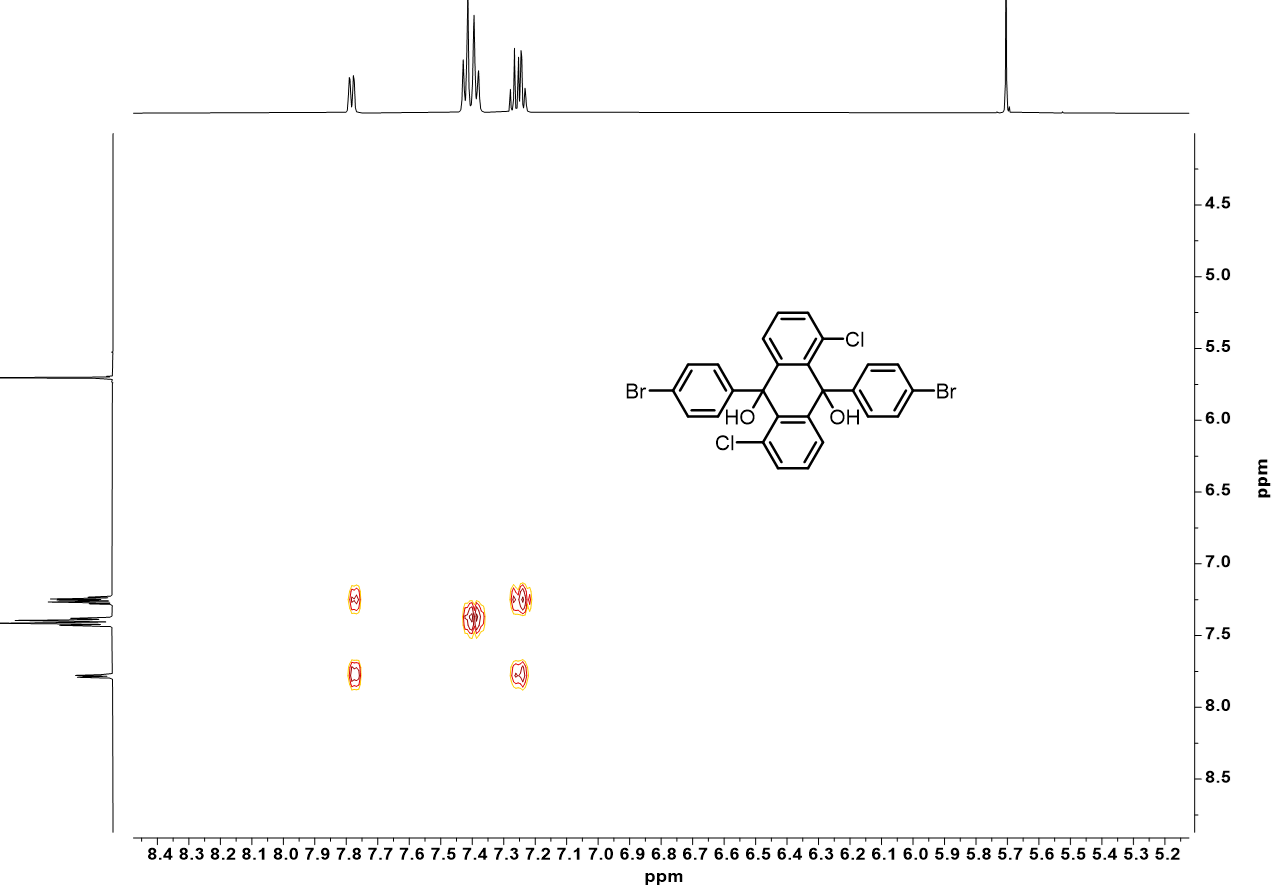


**Figure S26:** ^1^H,^1^H COSY spectrum of **10** (THF-d_8_, 600 MHz, 300 K).


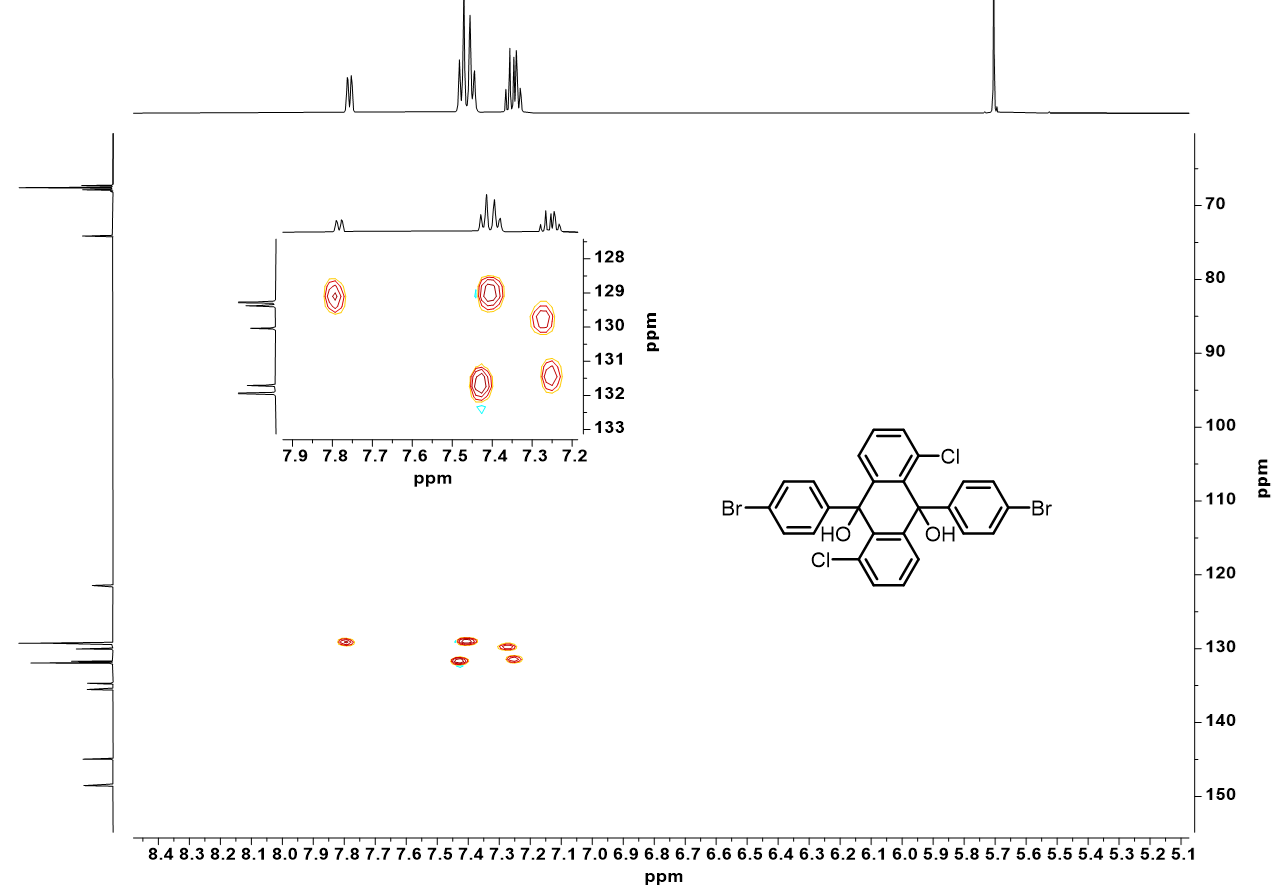


**Figure S27:** ^1^H,^13^C HSQC spectrum of **10** (THF-d_8_, 600 MHz, 151 MHz 300 K).


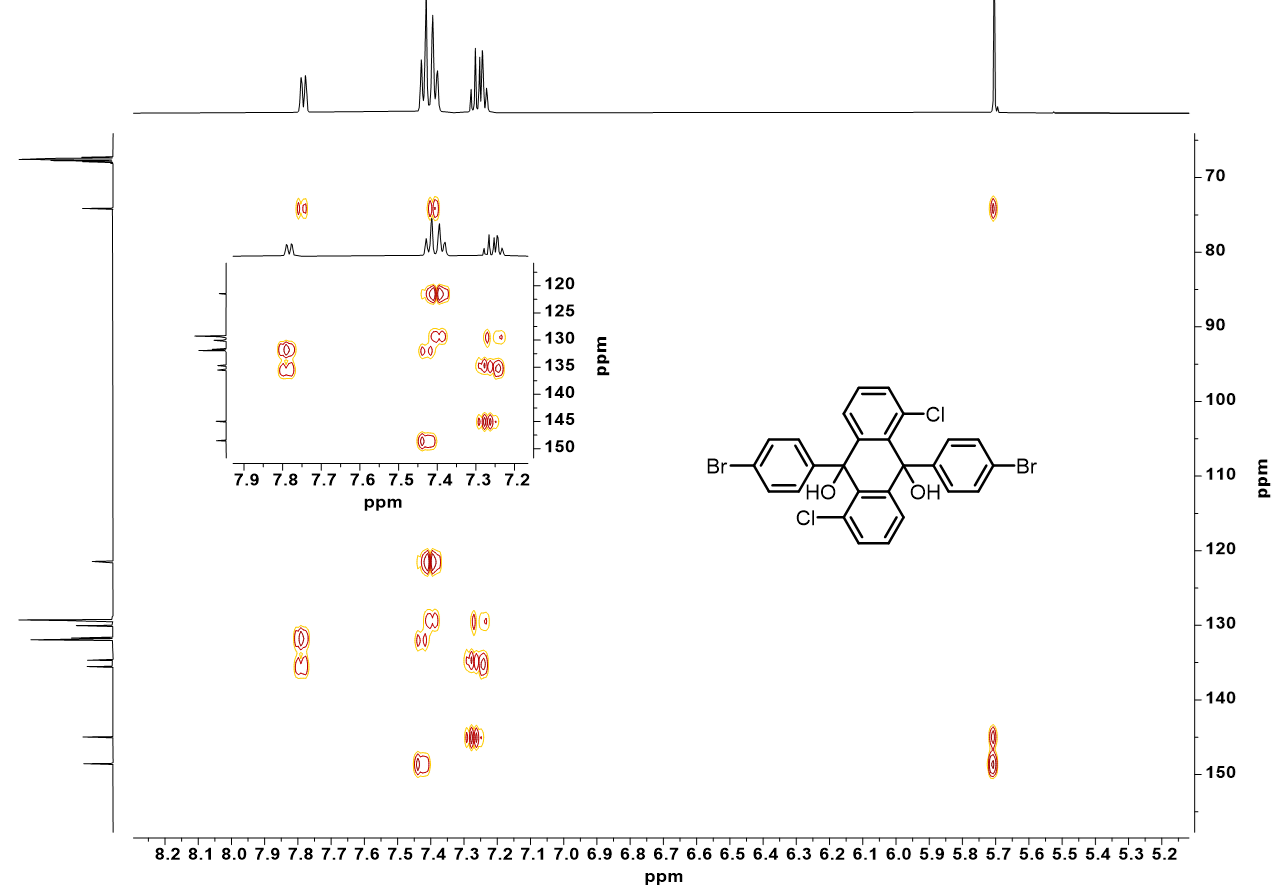


**Figure S28:** ^1^H,^13^C HMBC spectrum of **10** (THF-d_8_, 600 MHz, 151 MHz 300 K).


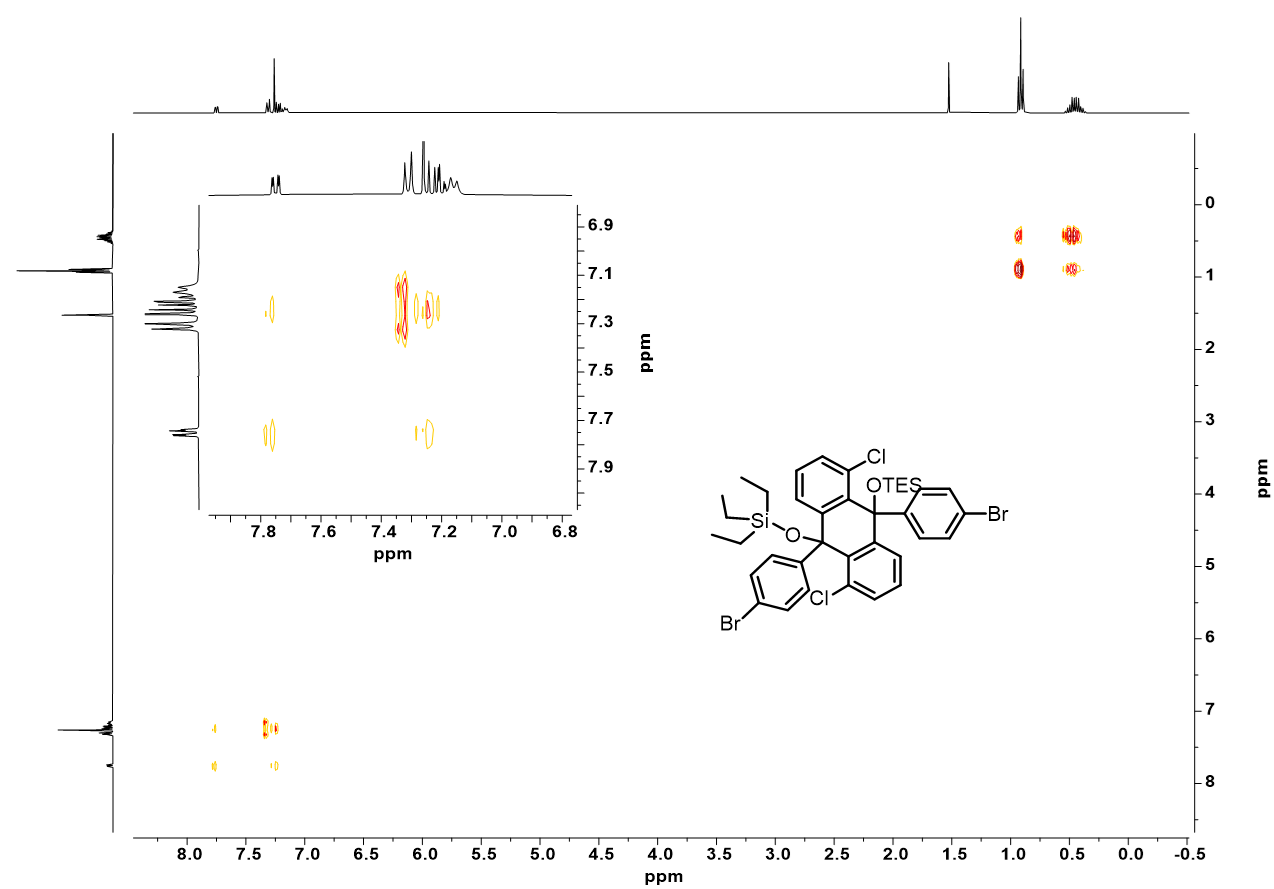


**Figure S29:** ^1^H,^1^H COSY spectrum of **11** (CDCl_3_, 400 MHz, 300 K).


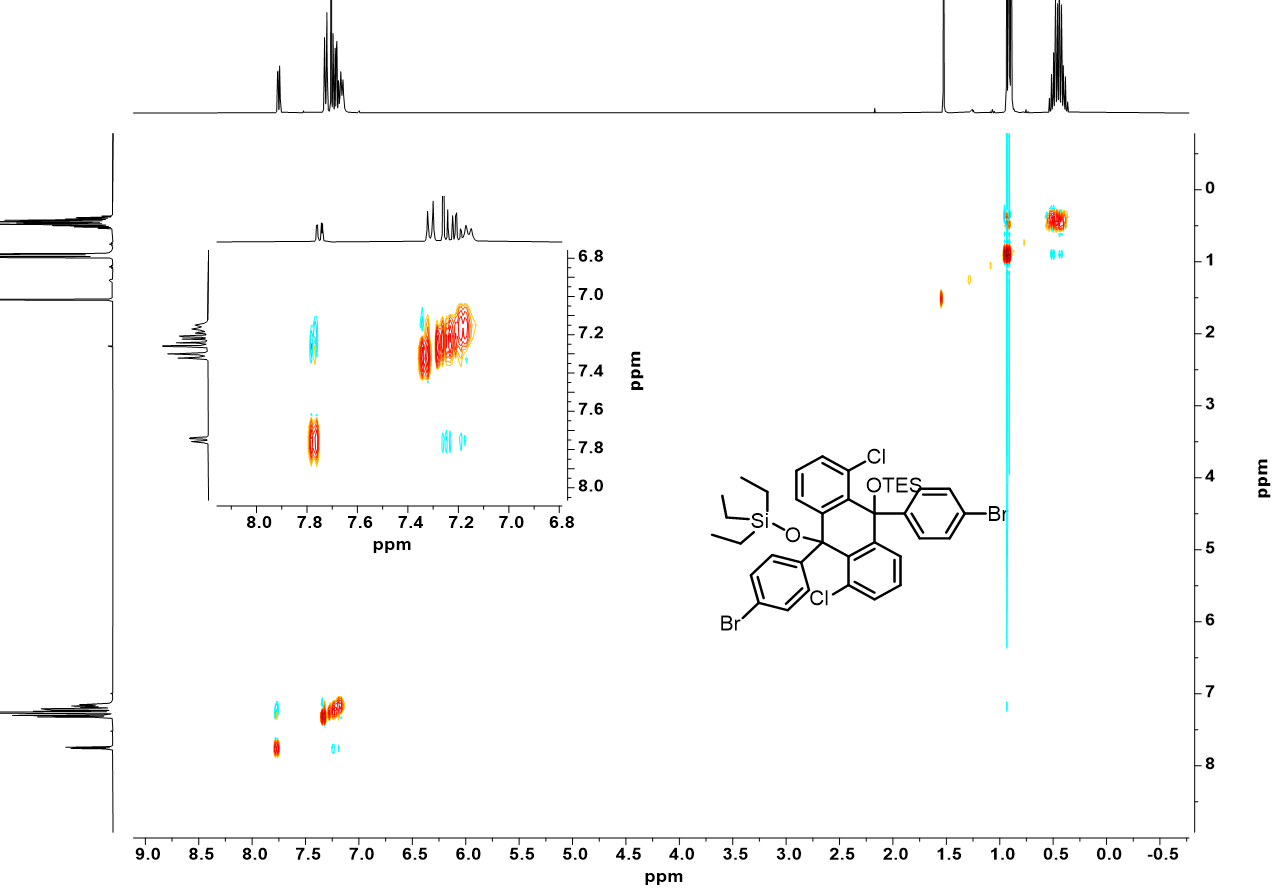


**Figure S30:** ^1^H,^1^H NOESY spectrum of **11** (CDCl_3_, 400 MHz, 300 K).


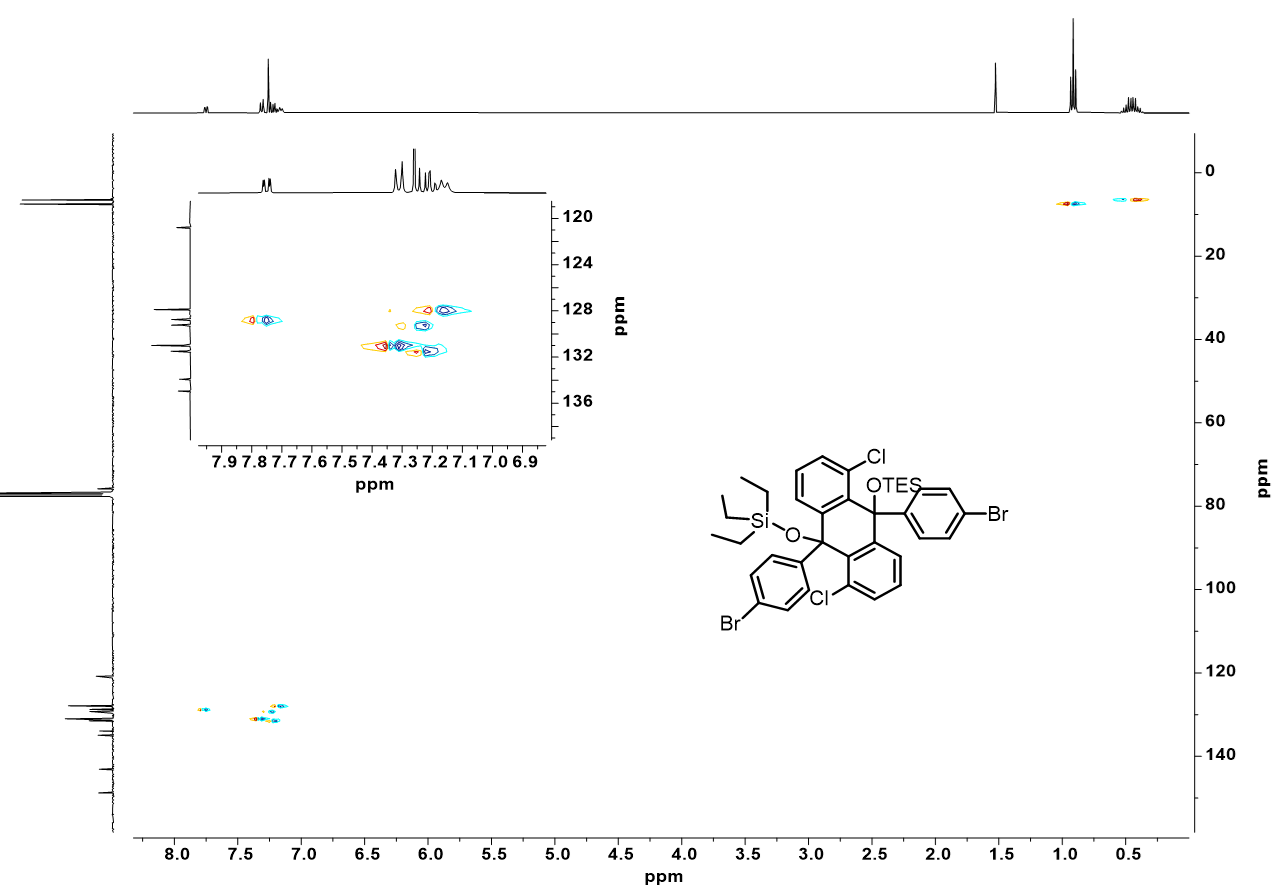


**Figure S31:** ^1^H,^13^C HSQC spectrum of **11** (CDCl_3_, 400 MHz, 101 MHz 300 K).


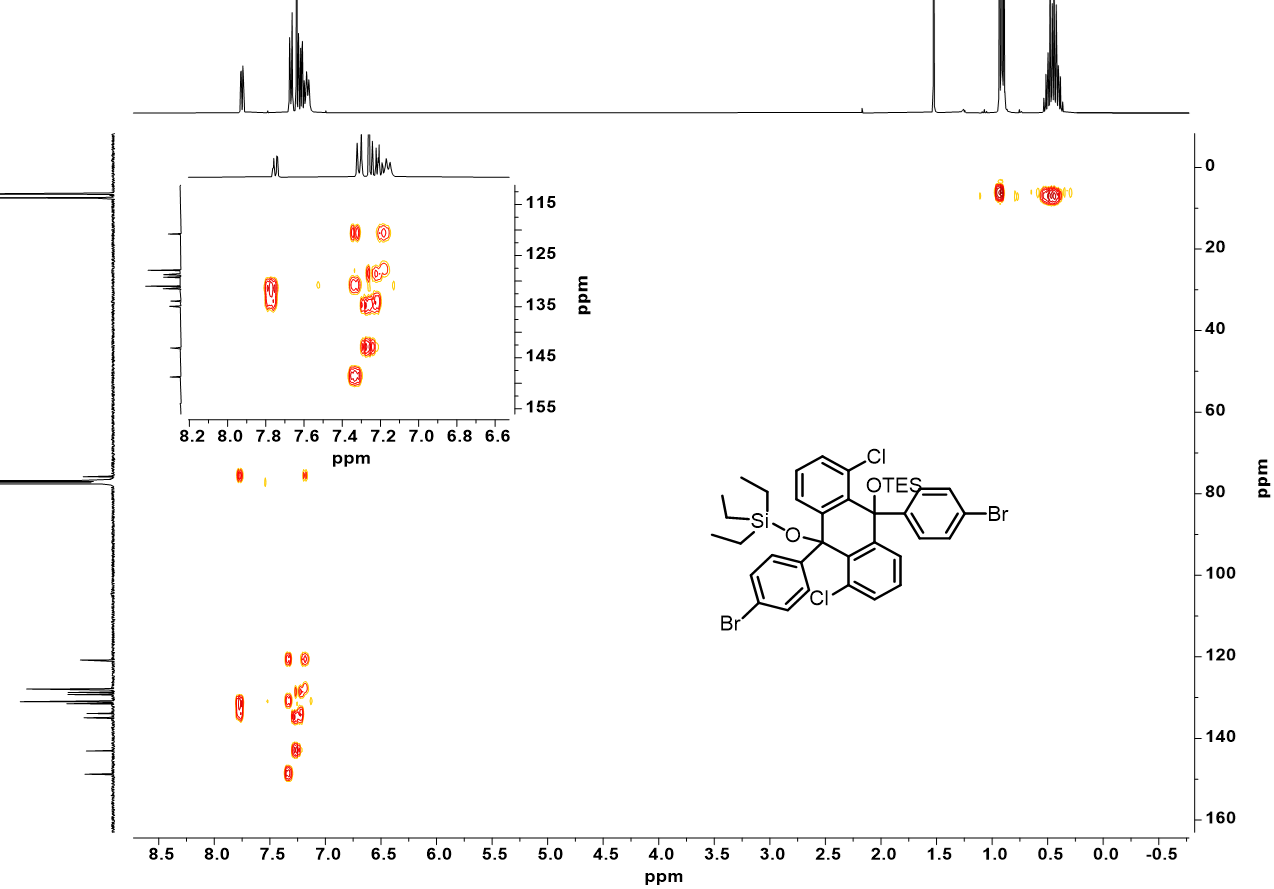


**Figure S32:** ^1^H,^13^C HMBC spectrum of **11** (CDCl_3_, 400 MHz, 101 MHz 300 K).


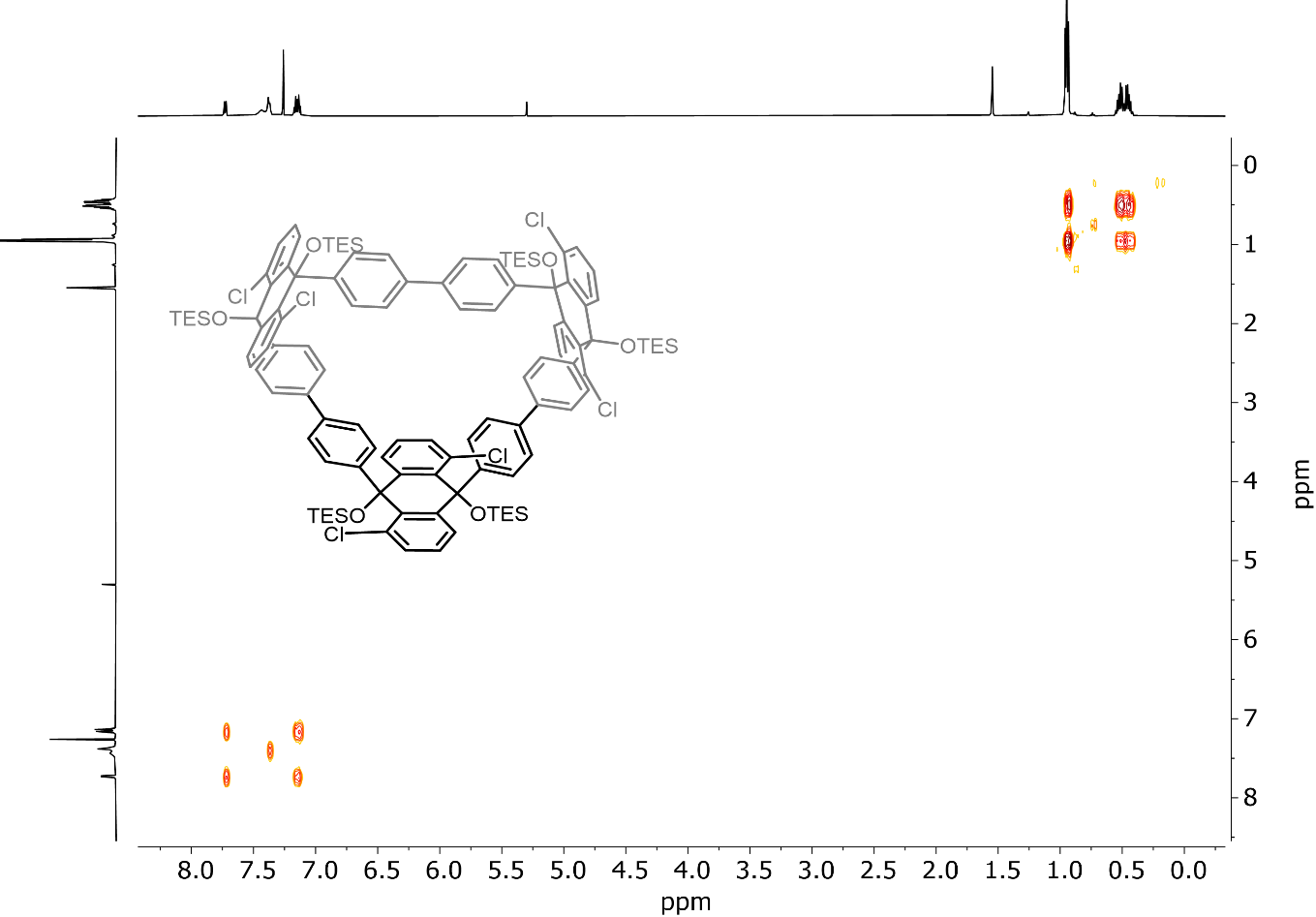


**Figure S33:** ^1^H,^1^H COSY spectrum of **12** (CDCl_3_, 600 MHz, 300 K).


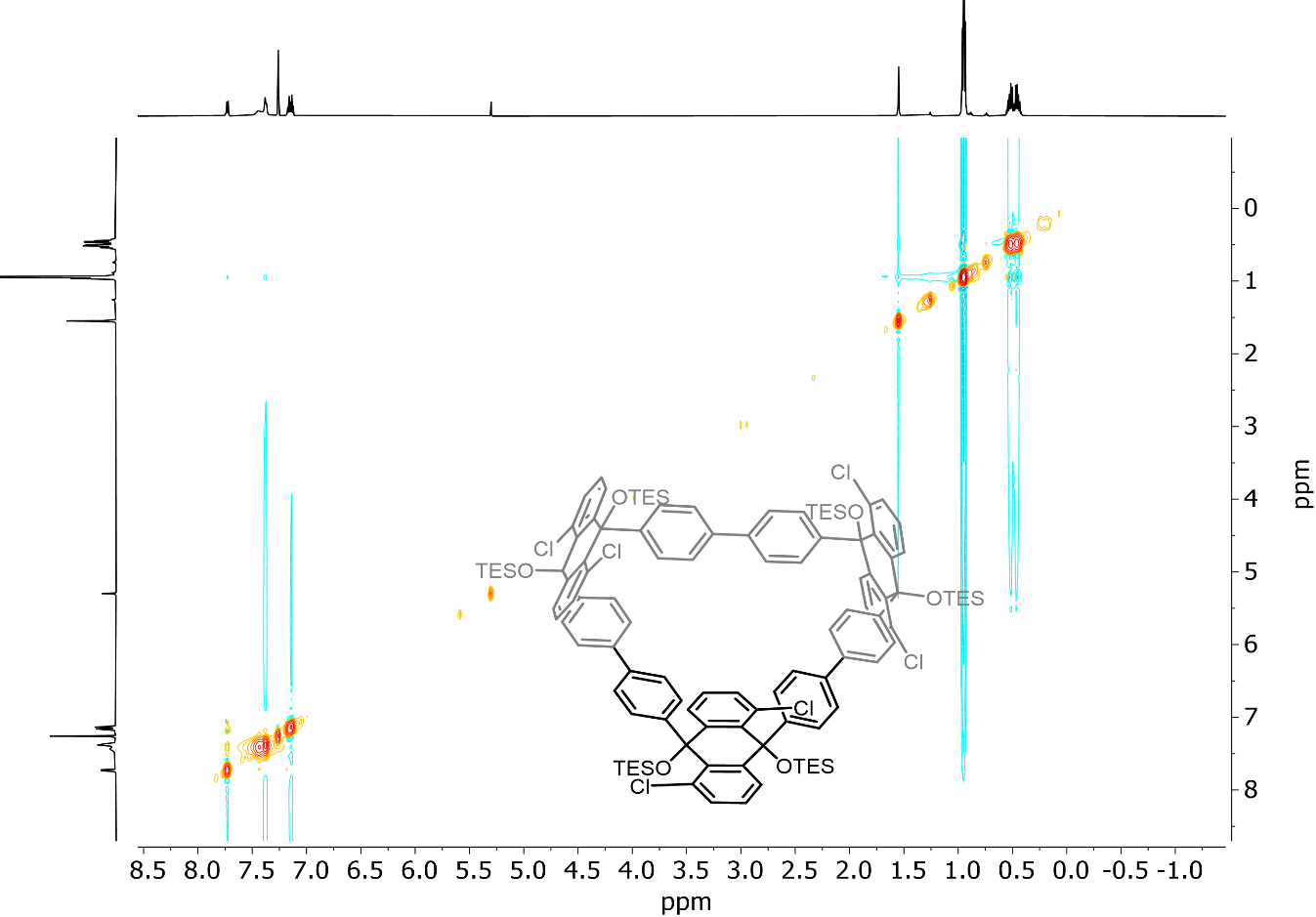


**Figure S34:** ^1^H,^1^H NOESY spectrum of **12** (CDCl_3_, 600 MHz, 300 K).


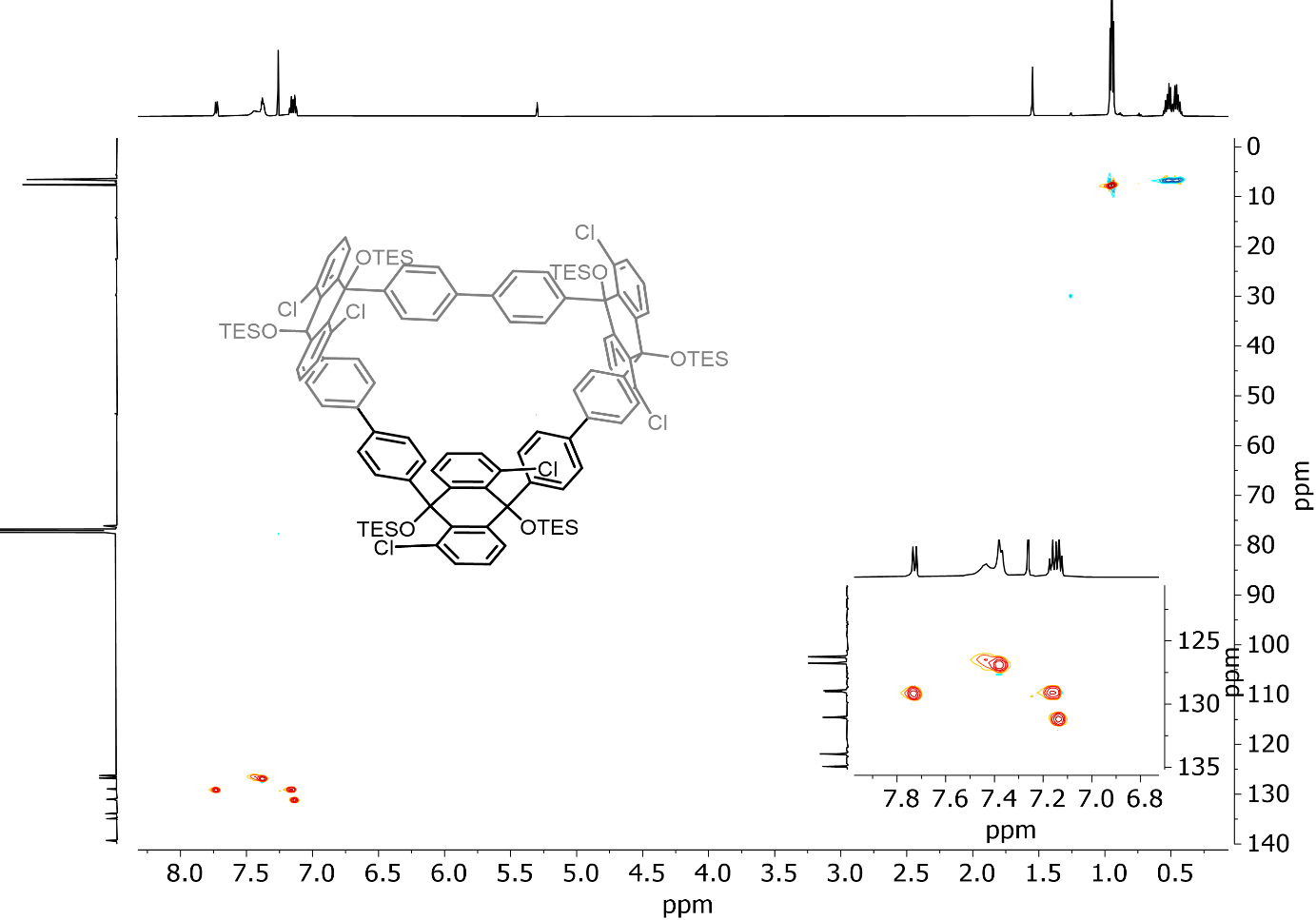


**Figure S35:** ^1^H,^13^C HSQC spectrum of **12** (CDCl_3_, 600 MHz, 151 MHz 300 K).


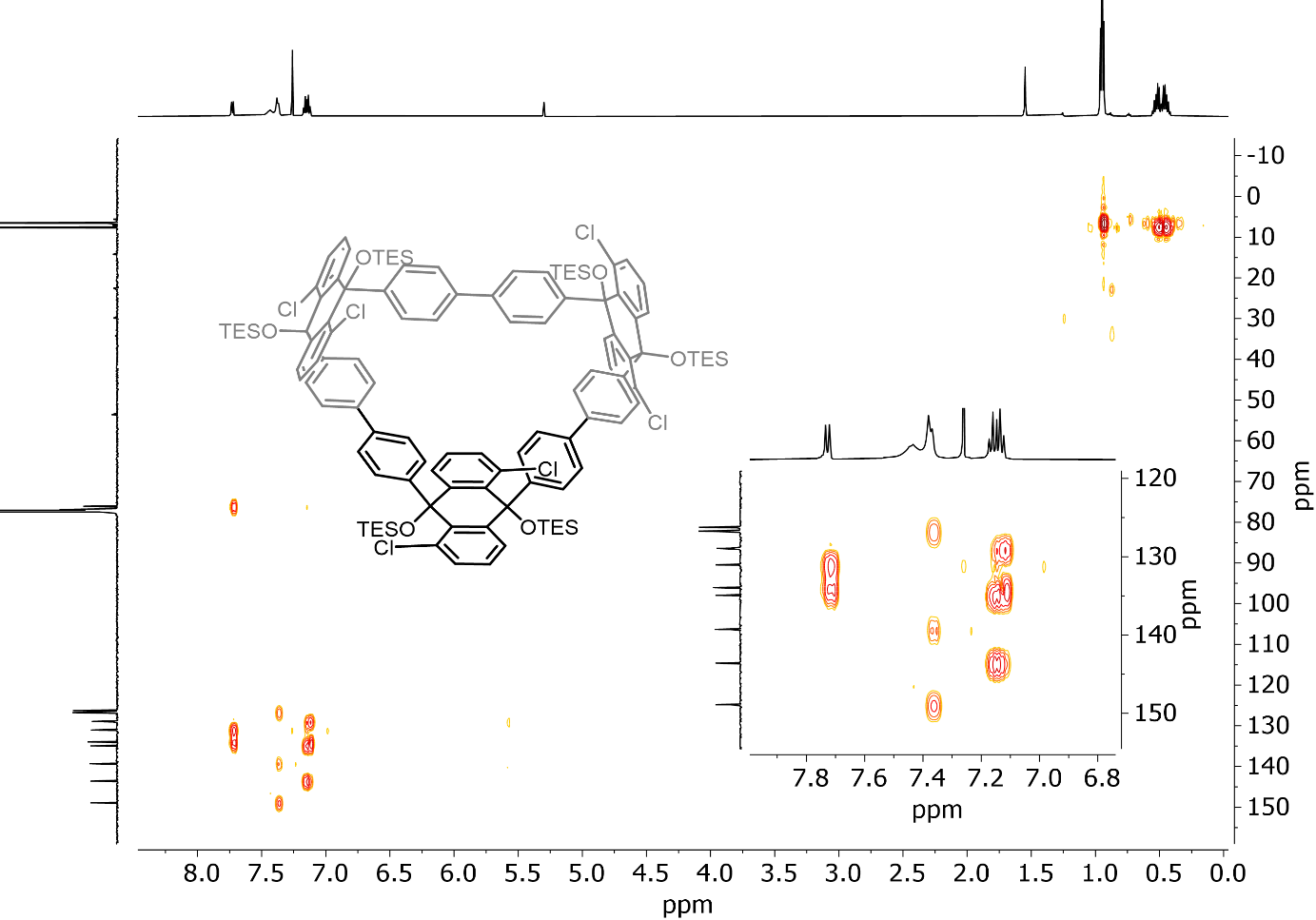


**Figure S36:** ^1^H,^13^C HMBC spectrum of **12** (CDCl_3_, 600 MHz, 151 MHz 300 K).


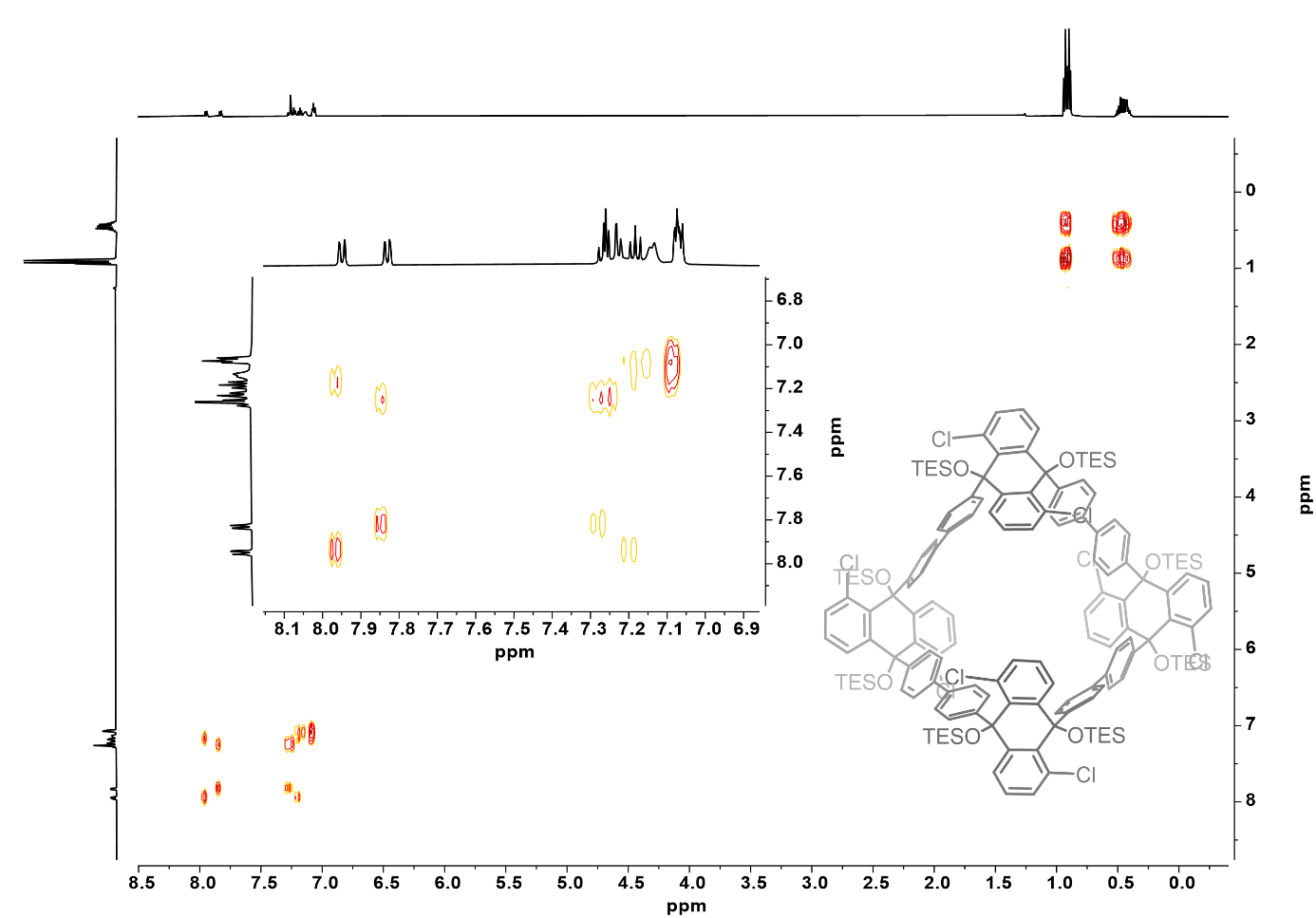


**Figure S37:** ^1^H,^1^H COSY spectrum of **14** (CDCl_3_, 600 MHz, 300 K).


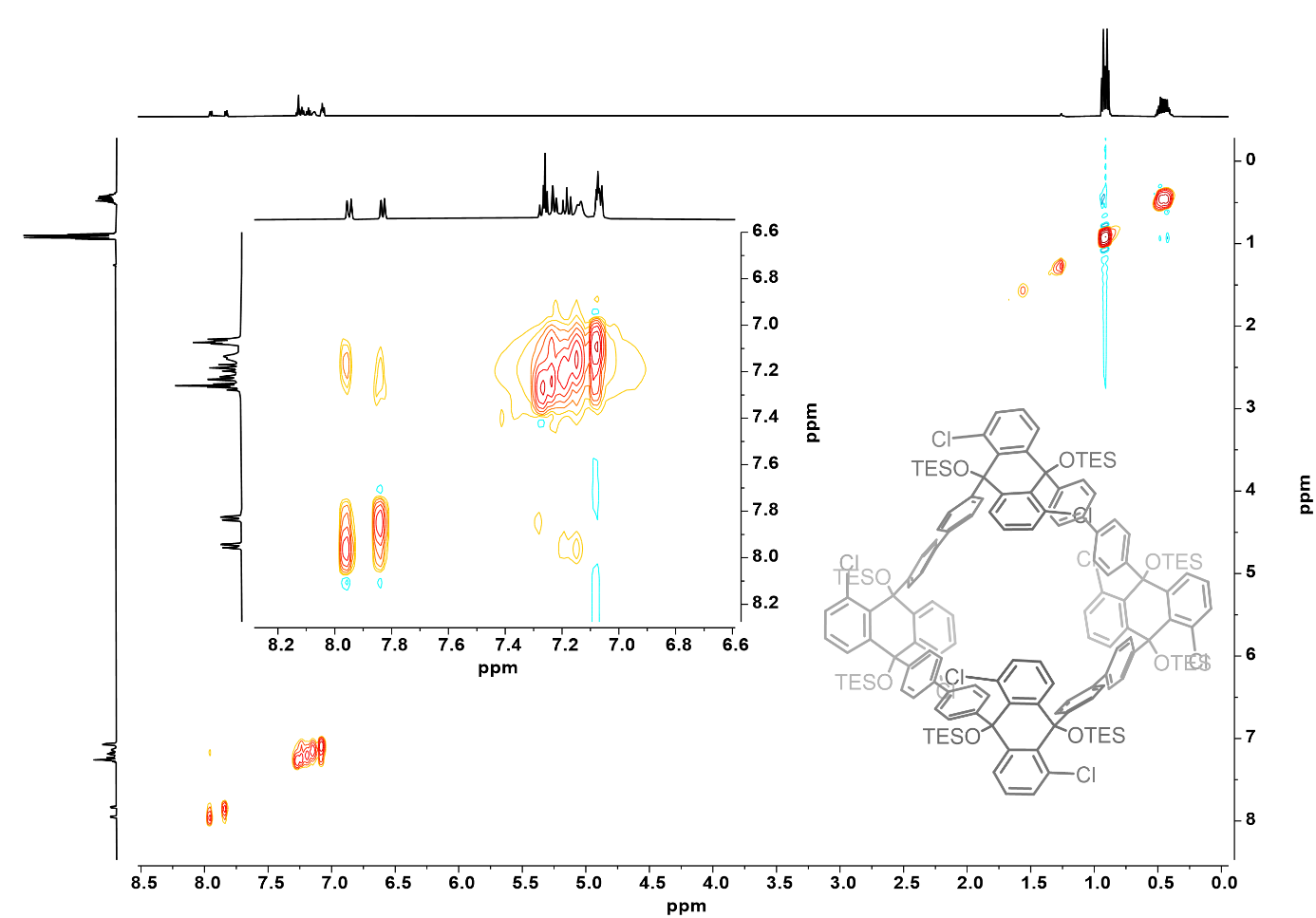


**Figure S38:** ^1^H,^1^H NOESY spectrum of **14** (CDCl_3_, 600 MHz, 300 K).


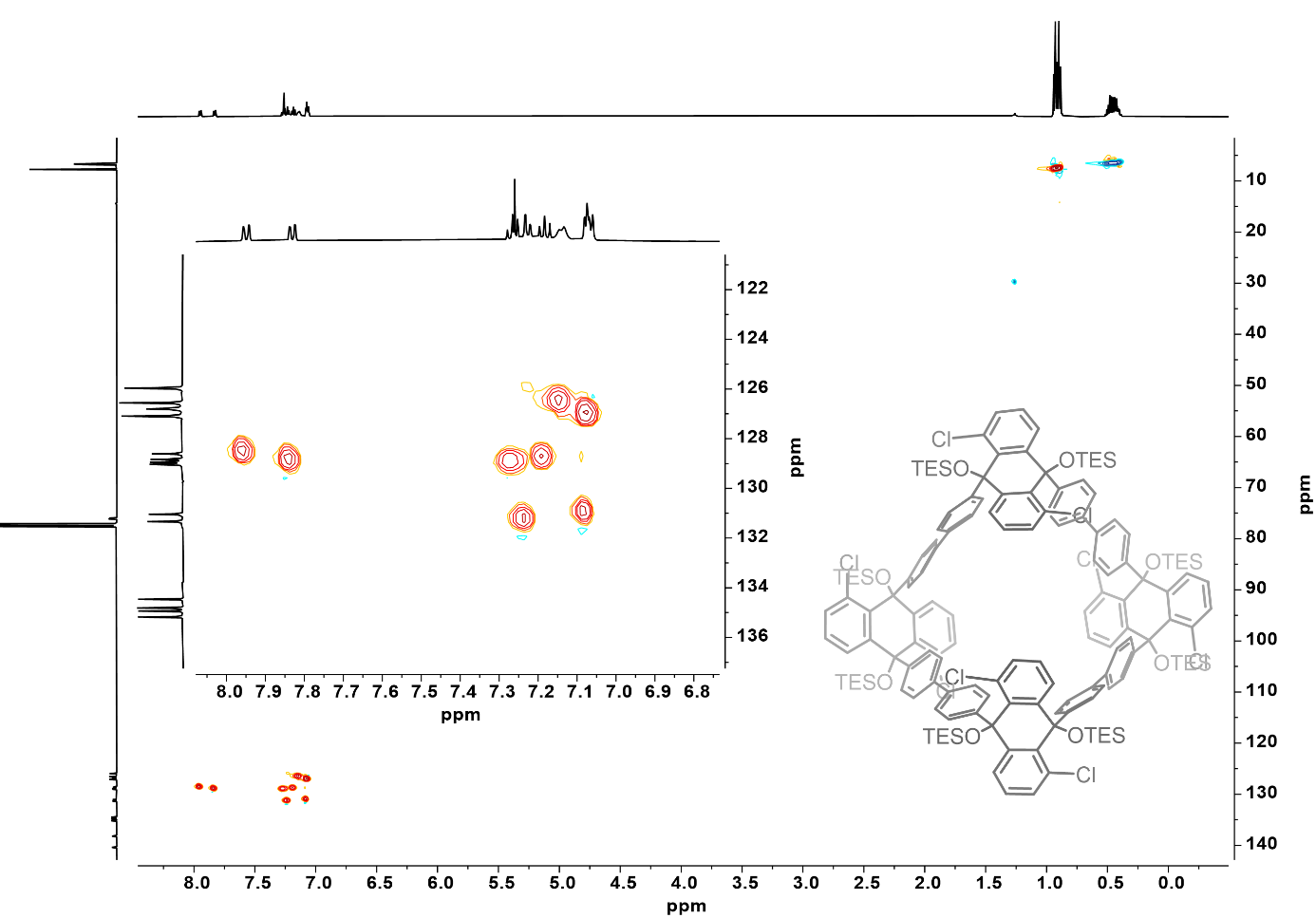


**Figure S39:** ^1^H,^13^C HSQC spectrum of **14** (CDCl_3_, 600 MHz, 151 MHz 300 K).


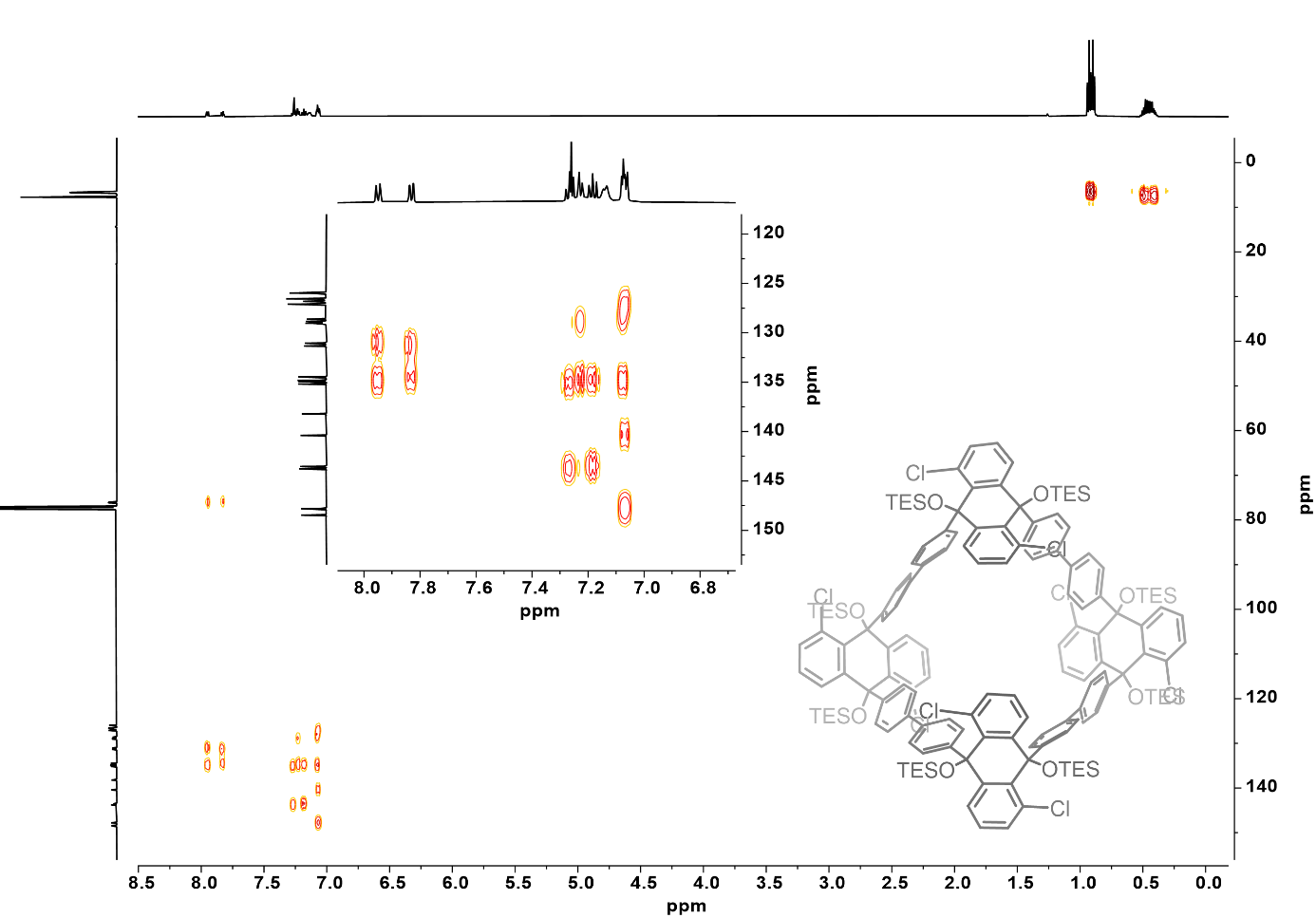


**Figure S40:** ^1^H,^13^C HMBC spectrum of **14** (CDCl_3_, 600 MHz, 151 MHz 300 K).


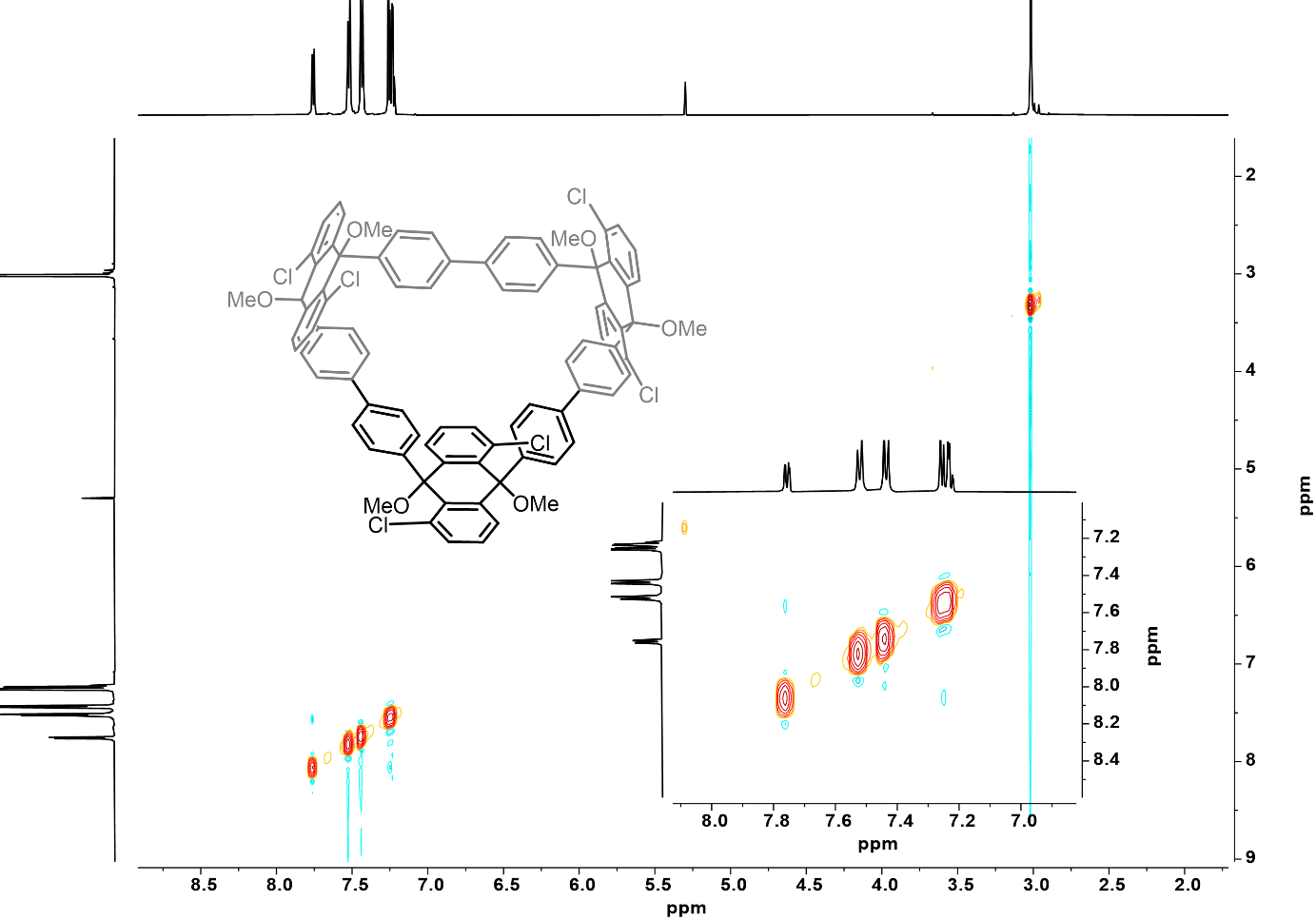


**Figure S41:** ^1^H,^1^H NOESY spectrum of **13** (CDCl_3_, 600 MHz, 300 K).


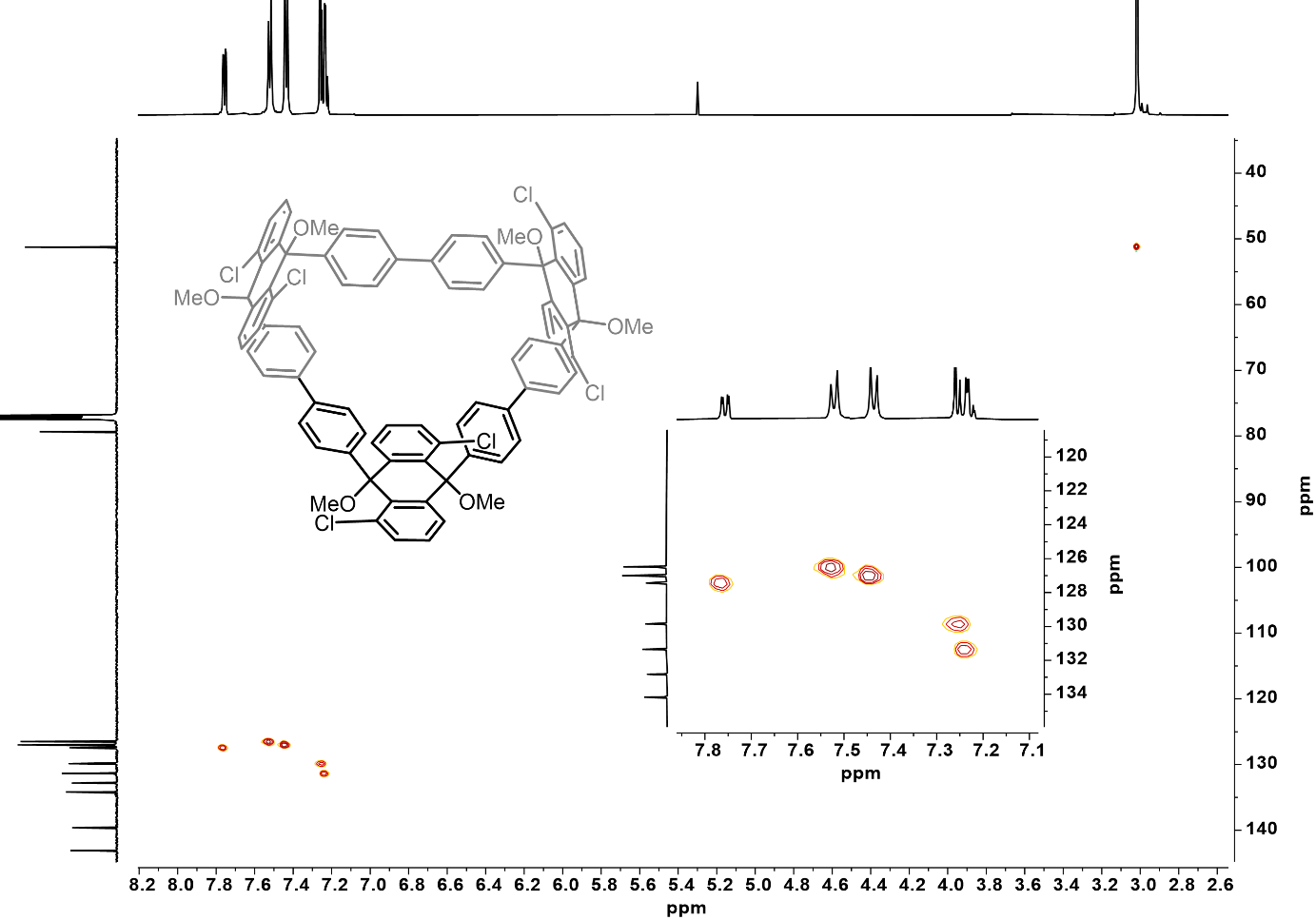


**Figure S42:** ^1^H,^13^C HSQC spectrum of **13** (CDCl_3_, 600 MHz, 151 MHz 300 K).


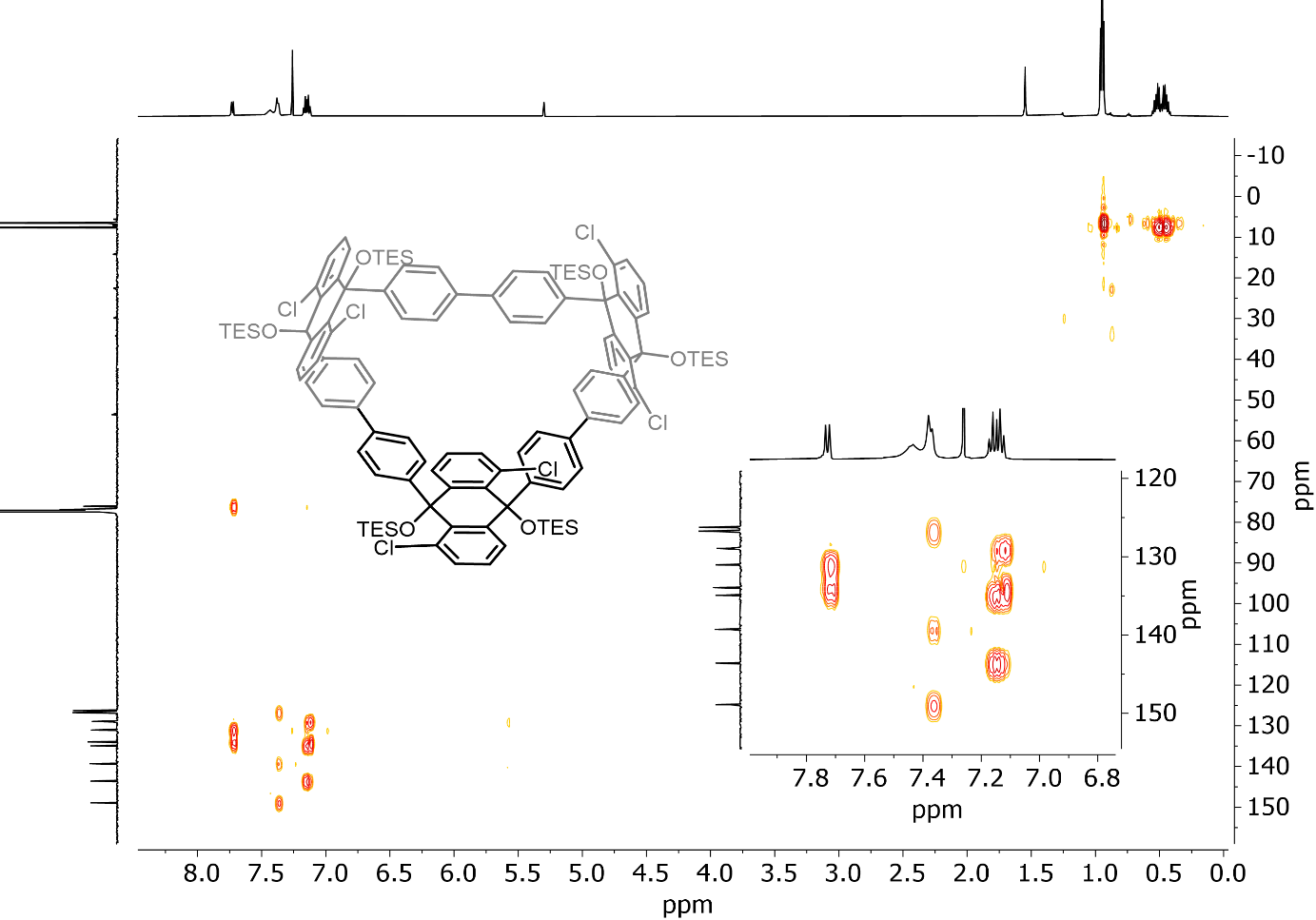


**Figure S43:** ^1^H,^13^C HMBC spectrum of **13** (CDCl_3_, 600 MHz, 151 MHz 300 K).


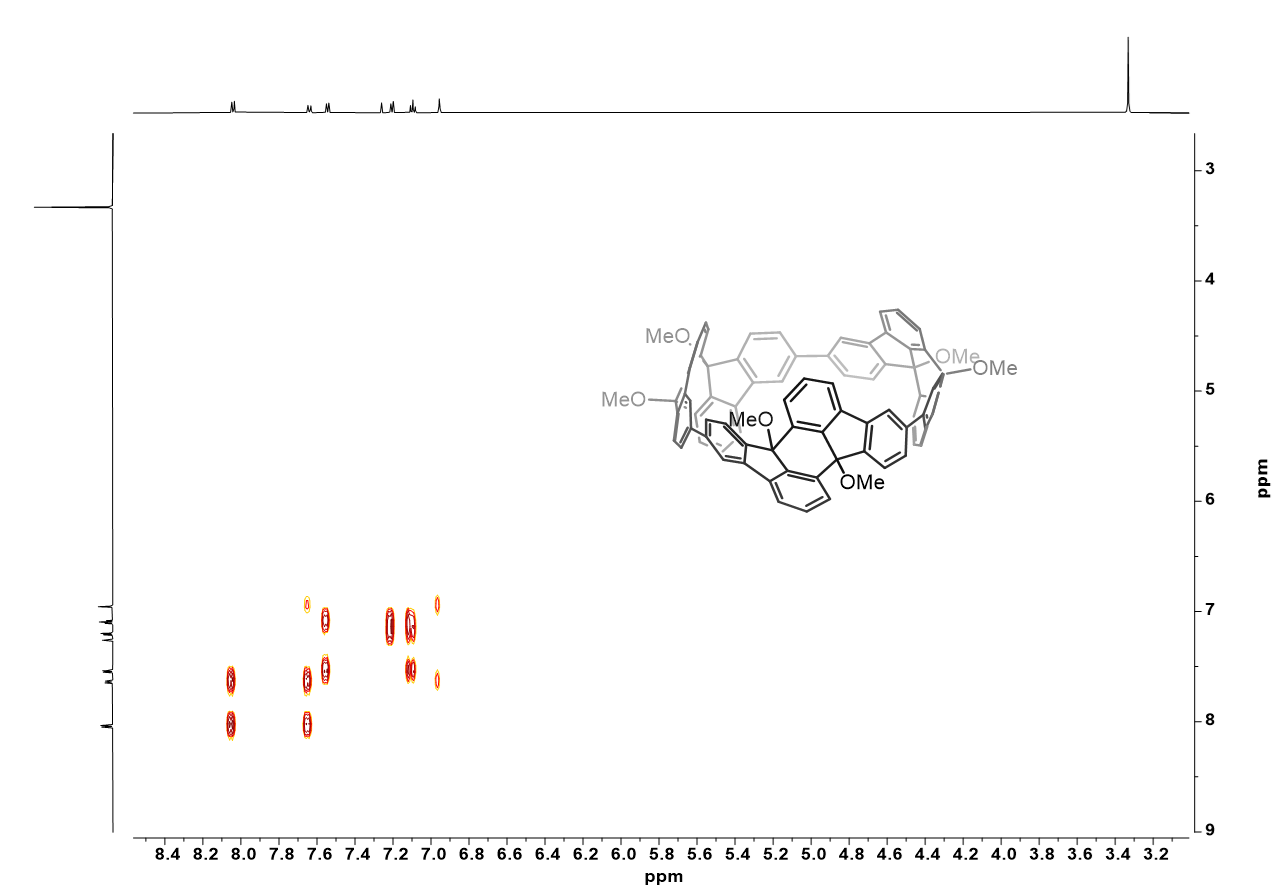


**Figure S44:** ^1^H,^1^H COSY spectrum of **15** (CDCl_3_, 600 MHz, 300 K).


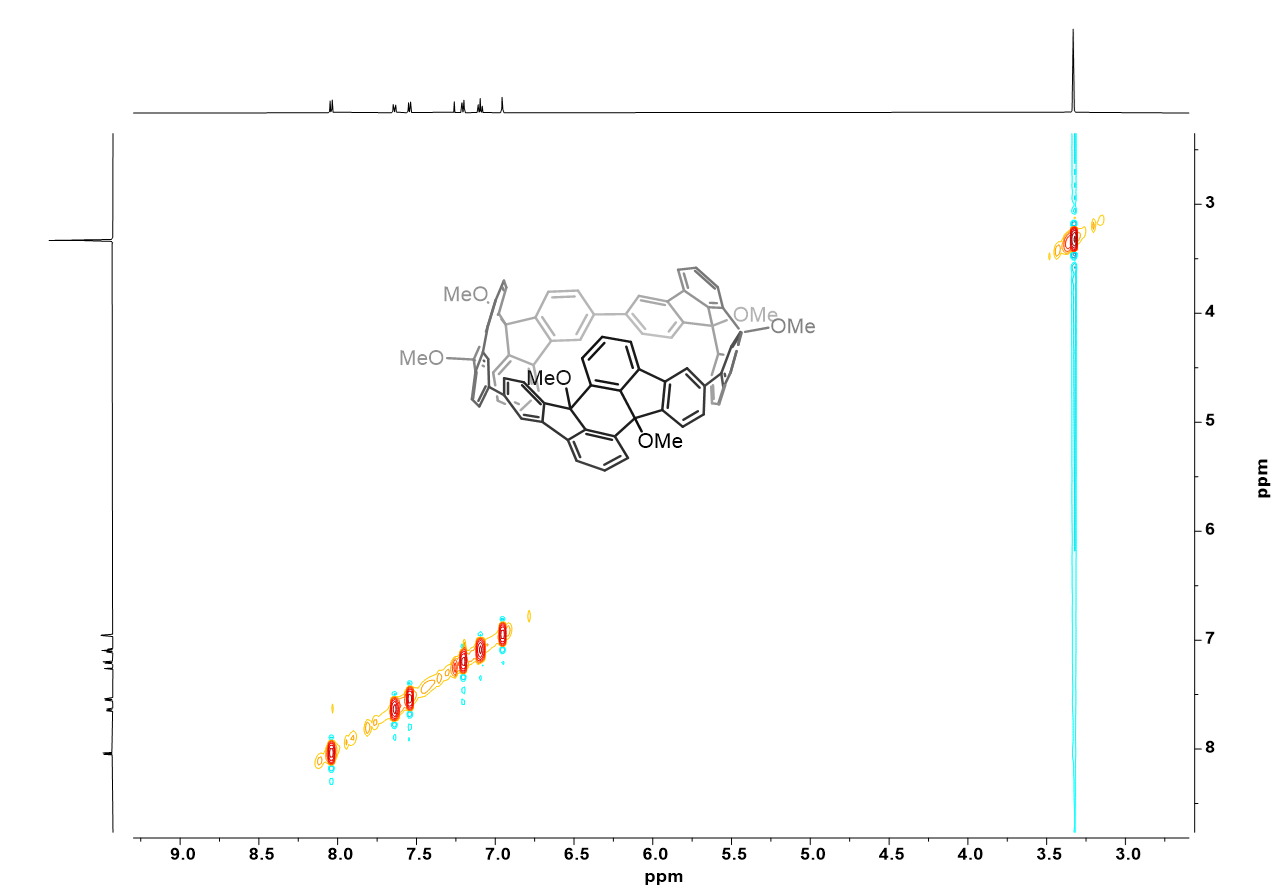


**Figure S45:** ^1^H,^1^H NOESY spectrum of **15** (CDCl_3_, 600 MHz, 300 K).


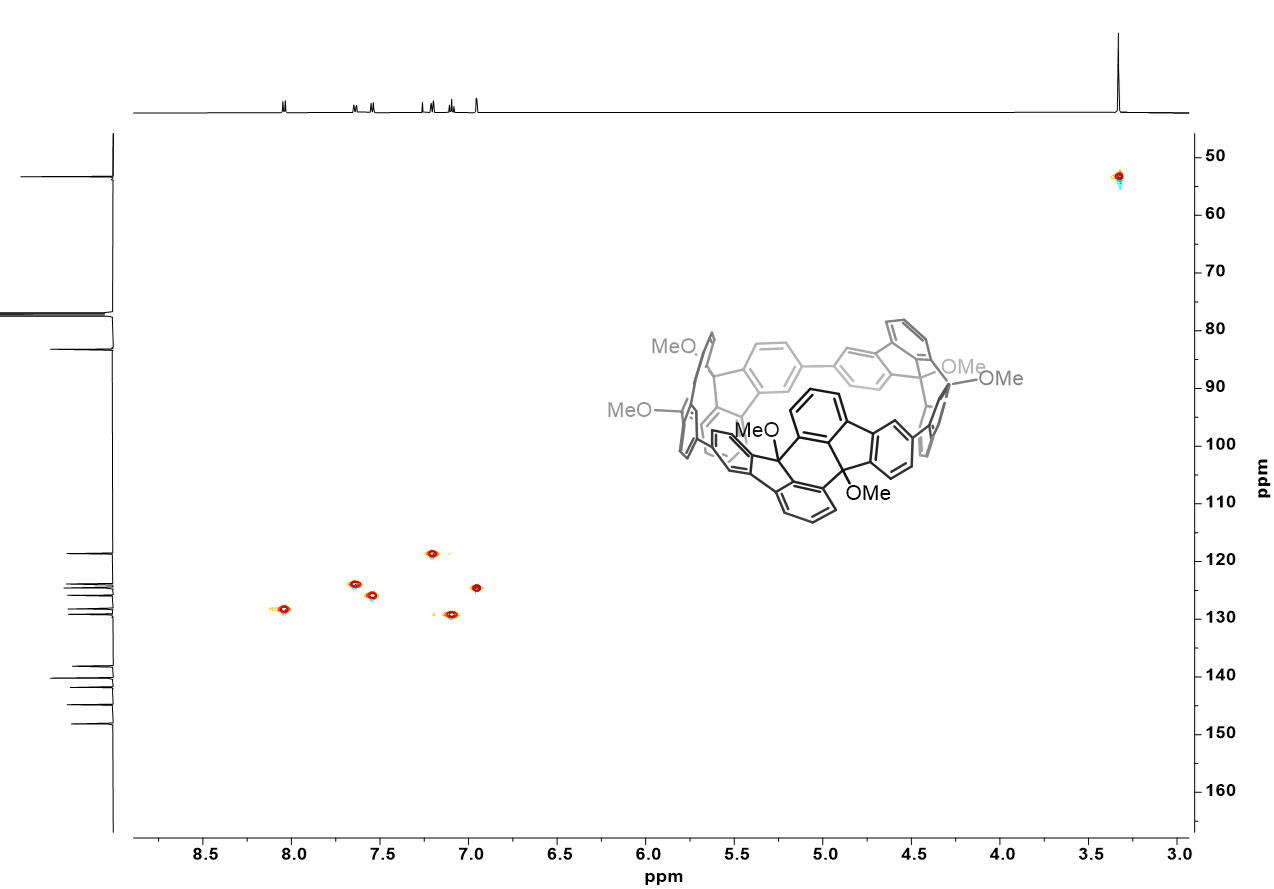


**Figure S46:** ^1^H,^13^C HSQC spectrum of **15** (CDCl_3_, 600 MHz, 151 MHz 300 K).


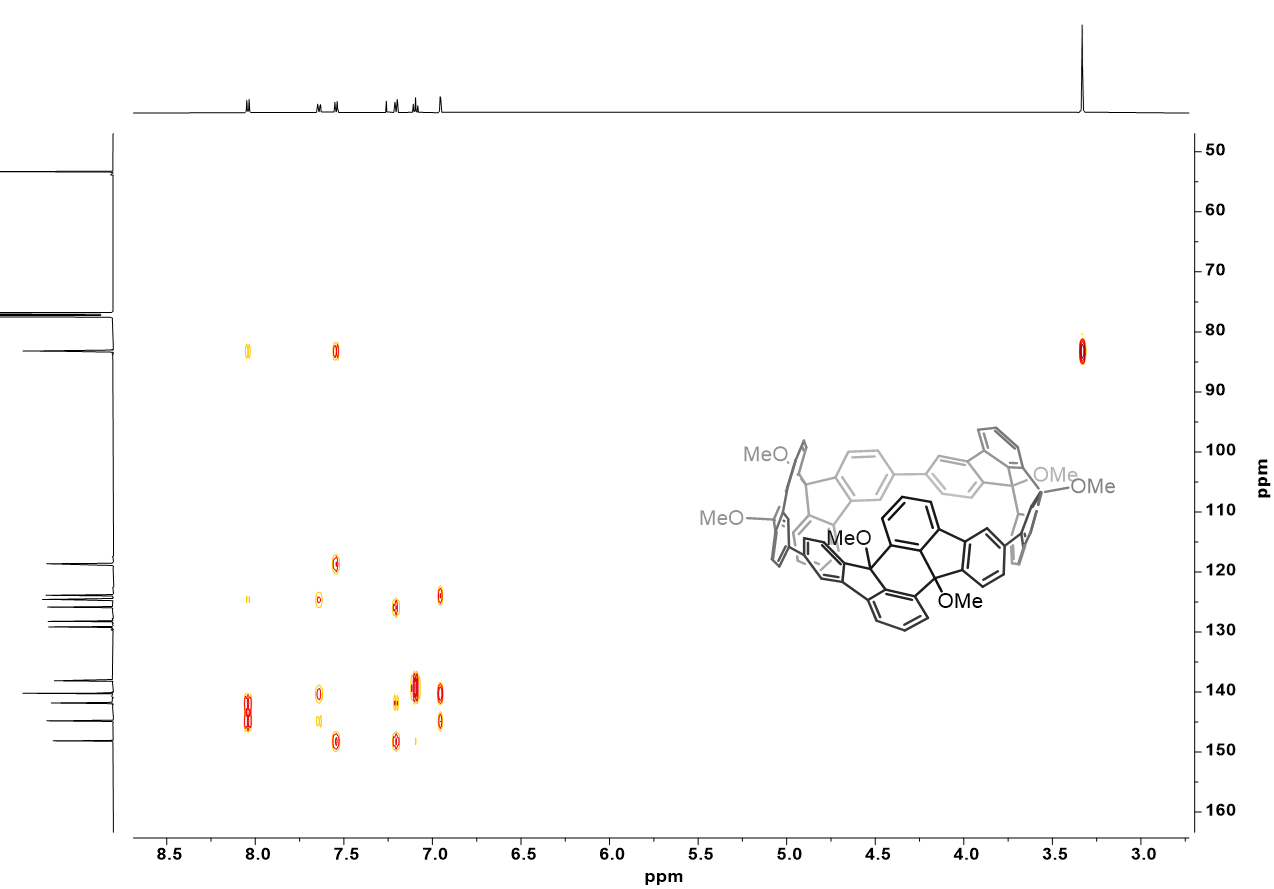


**Figure S47:** ^1^H,^13^C HMBC spectrum of **15** (CDCl_3_, 600 MHz, 151 MHz 300 K).


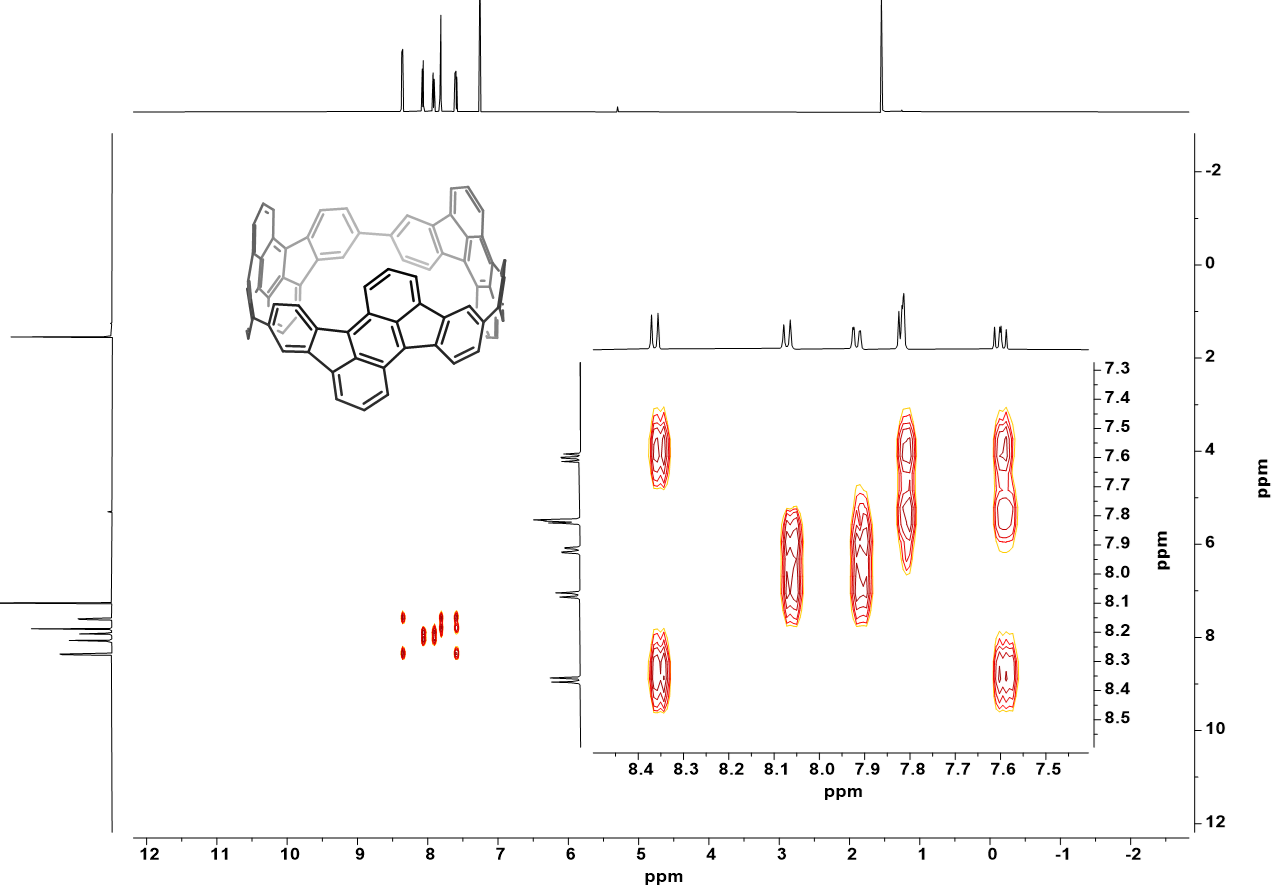


**Figure S48:** ^1^H,^1^H COSY spectrum of **7** (CDCl_3_, 600 MHz, 300 K).


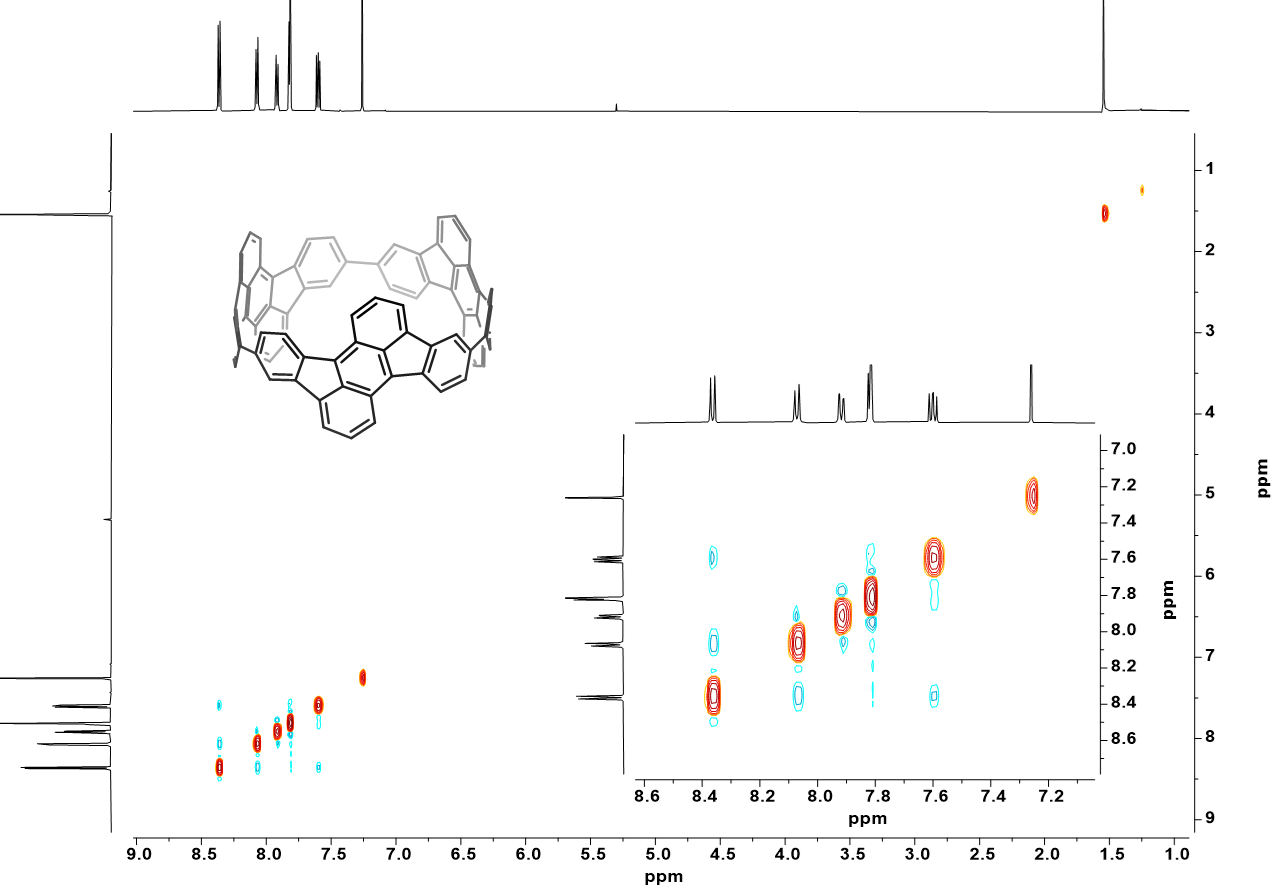


**Figure S49:** ^1^H,^1^H NOESY spectrum of **7** (CDCl_3_, 600 MHz, 300 K).


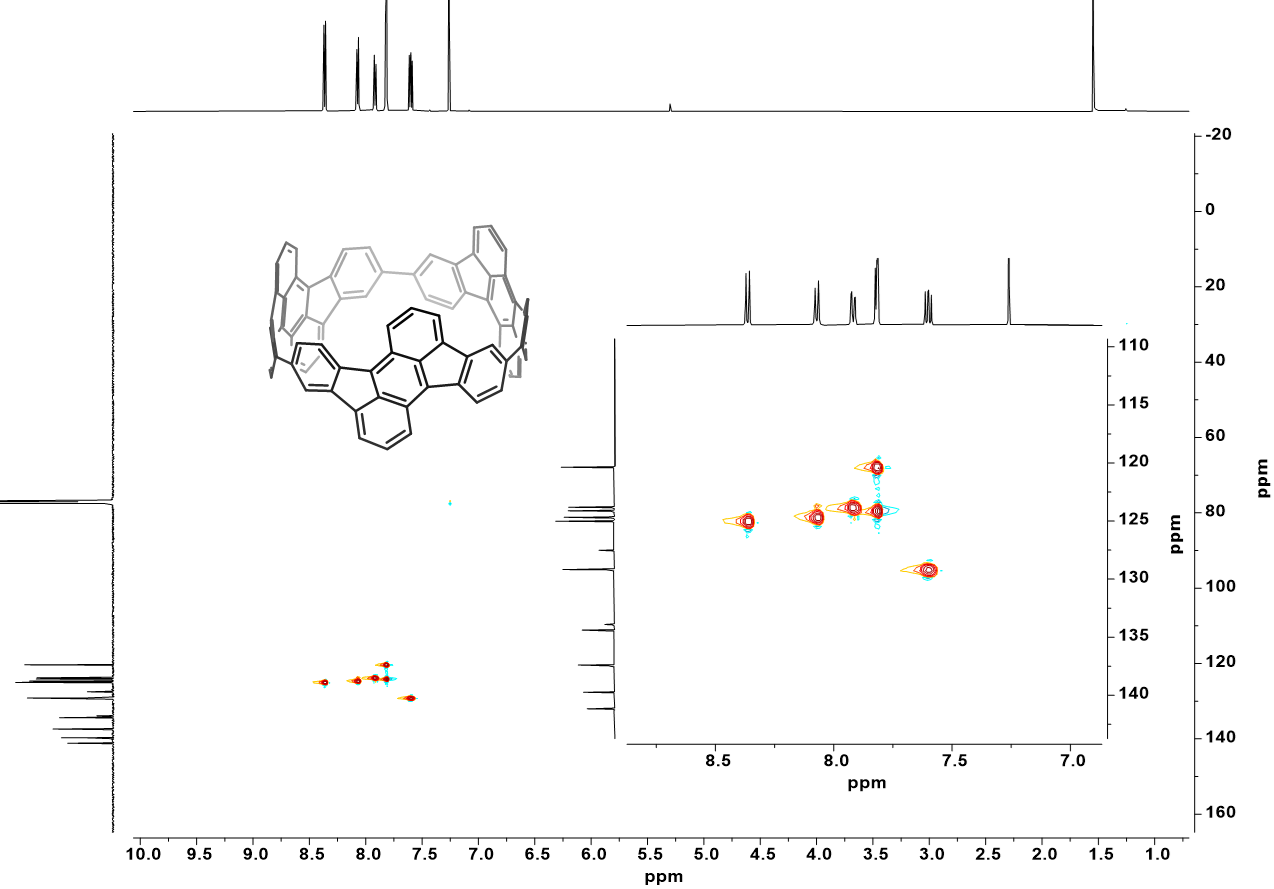


**Figure S50:** ^1^H,^13^C HSQC spectrum of **7** (CDCl_3_, 600 MHz, 151 MHz 300 K).


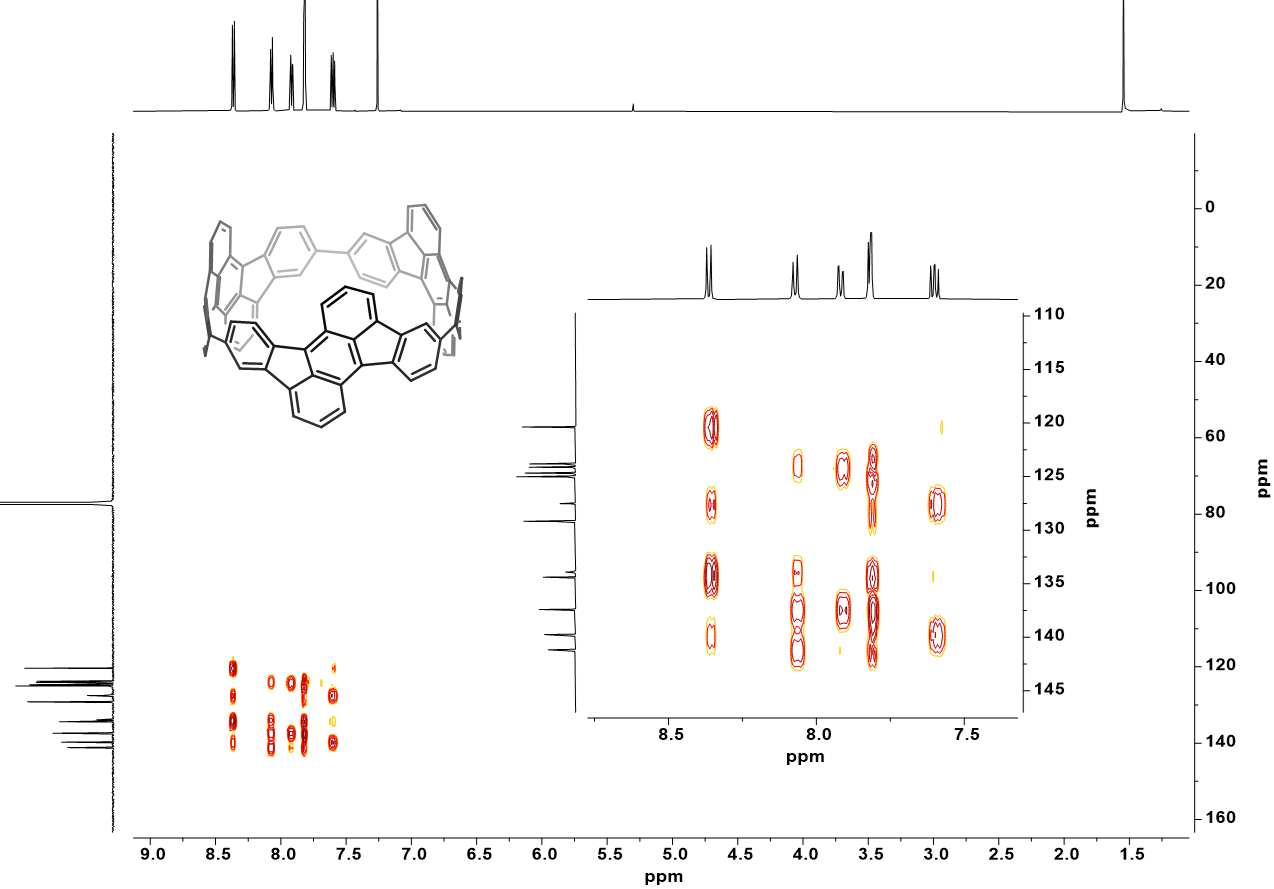


**Figure S51:** ^1^H,^13^C HMBC spectrum of **7** (CDCl_3_, 600 MHz, 151 MHz 300 K).

## IR spectra


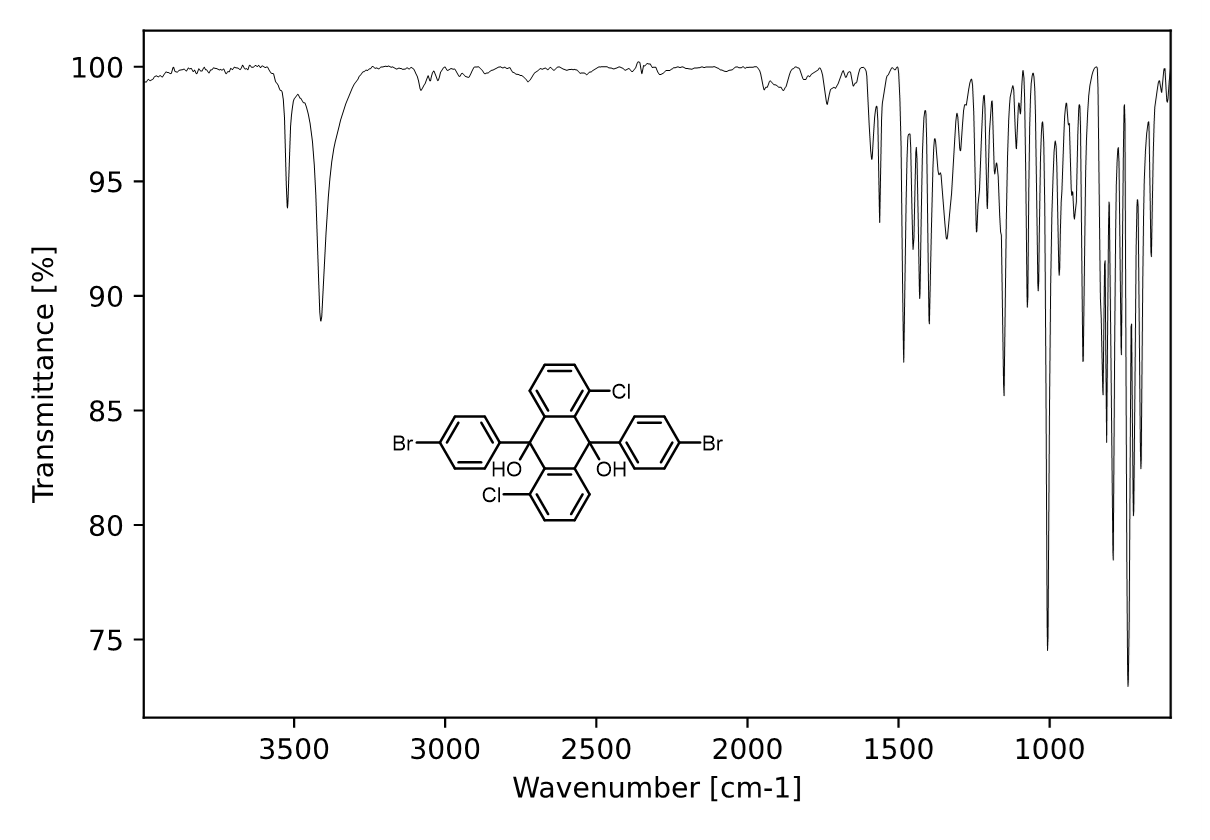


**Figure S52:** IR spectrum of **10** (ATR).


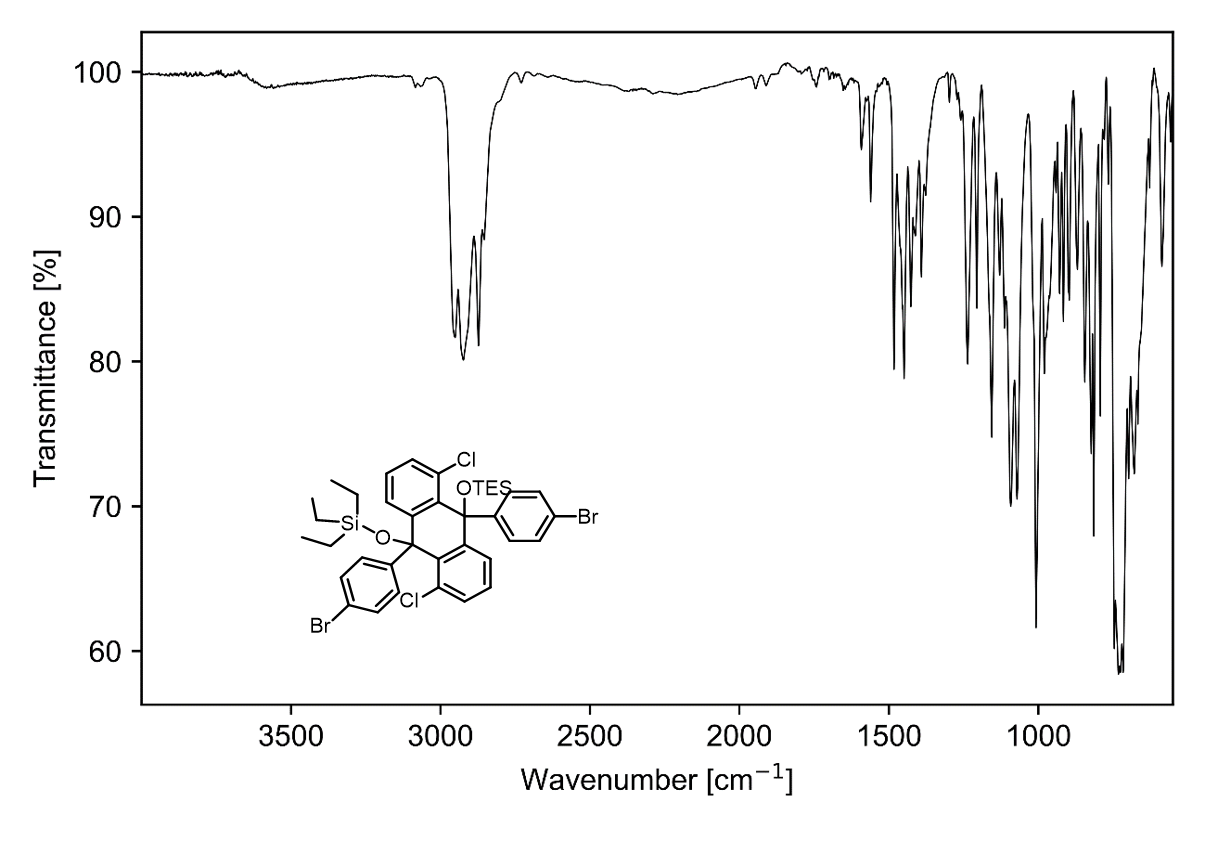


**Figure S53:** IR spectrum of **11** (ATR).


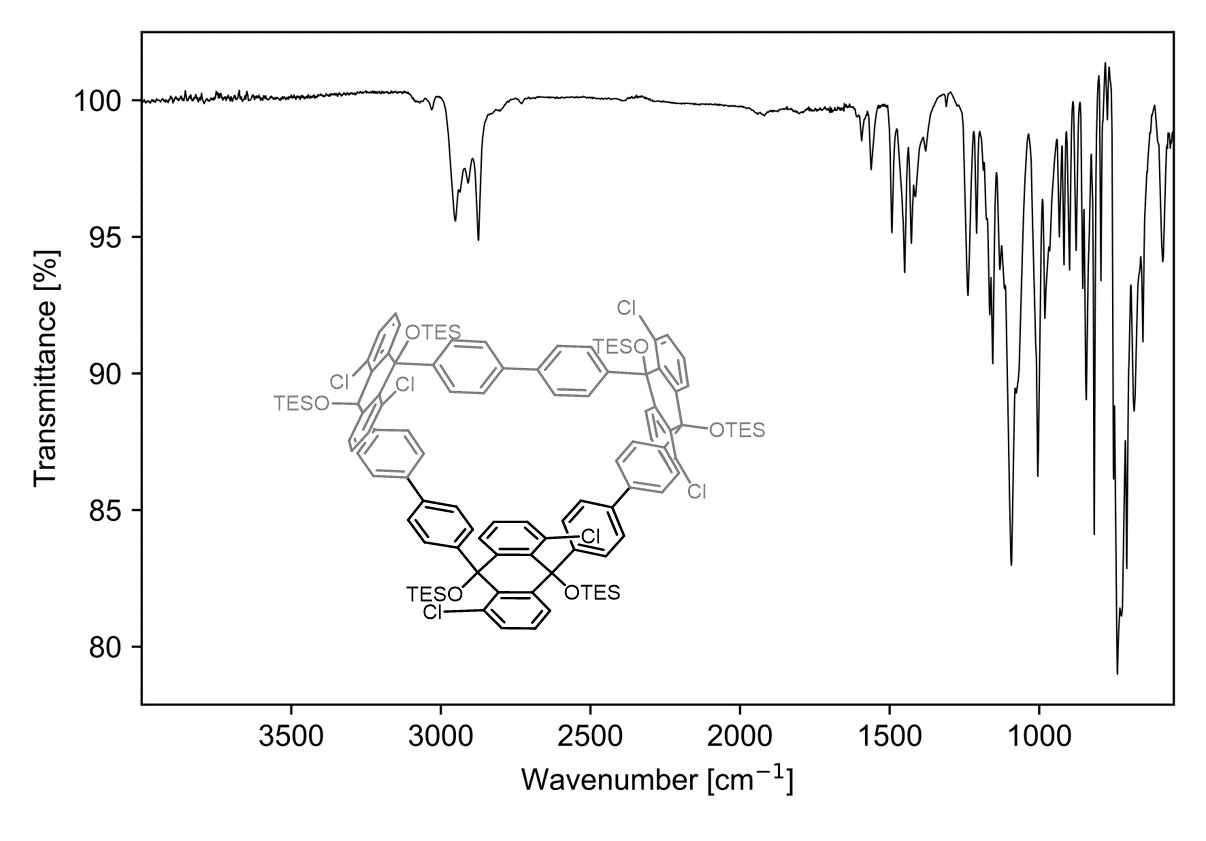


**Figure S54:** IR spectrum of (all-S)-**12** (ATR).


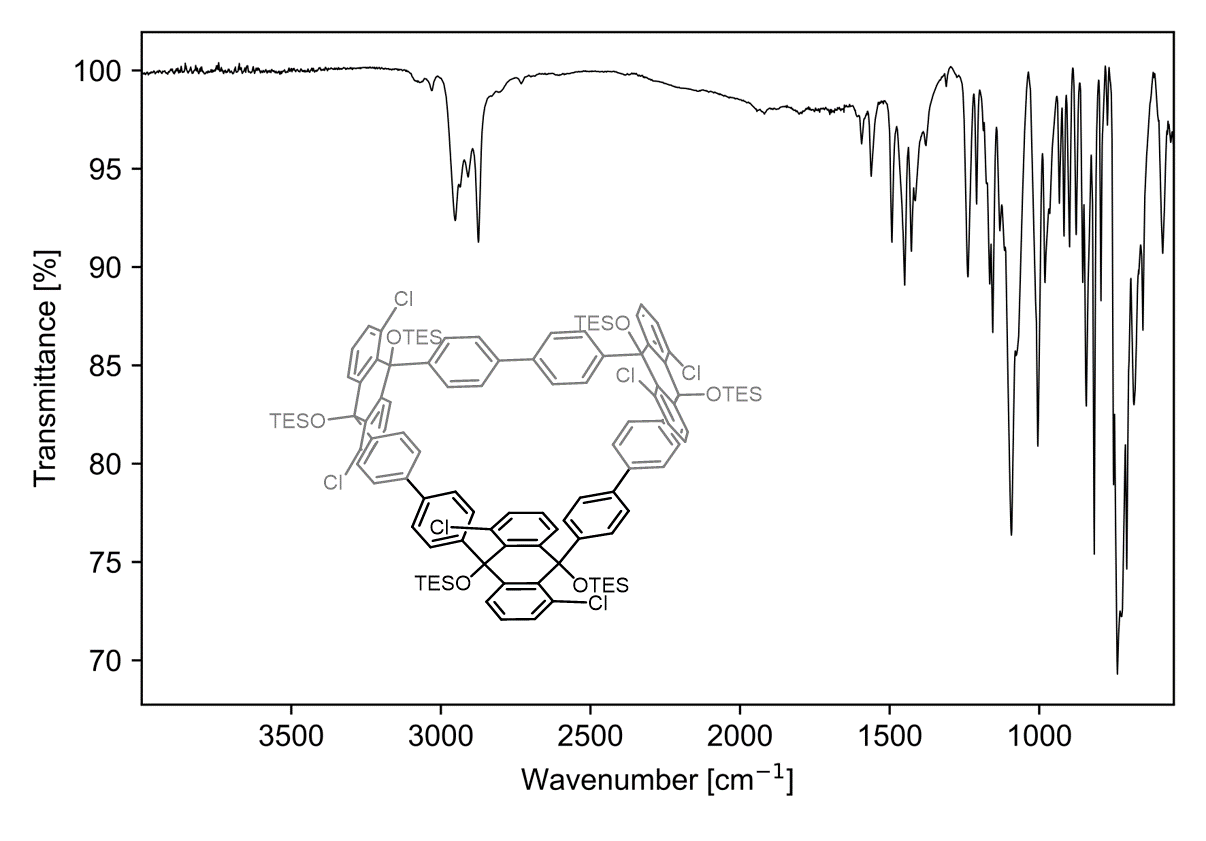


**Figure S55:** IR spectrum of (all-R)-**12** (ATR).


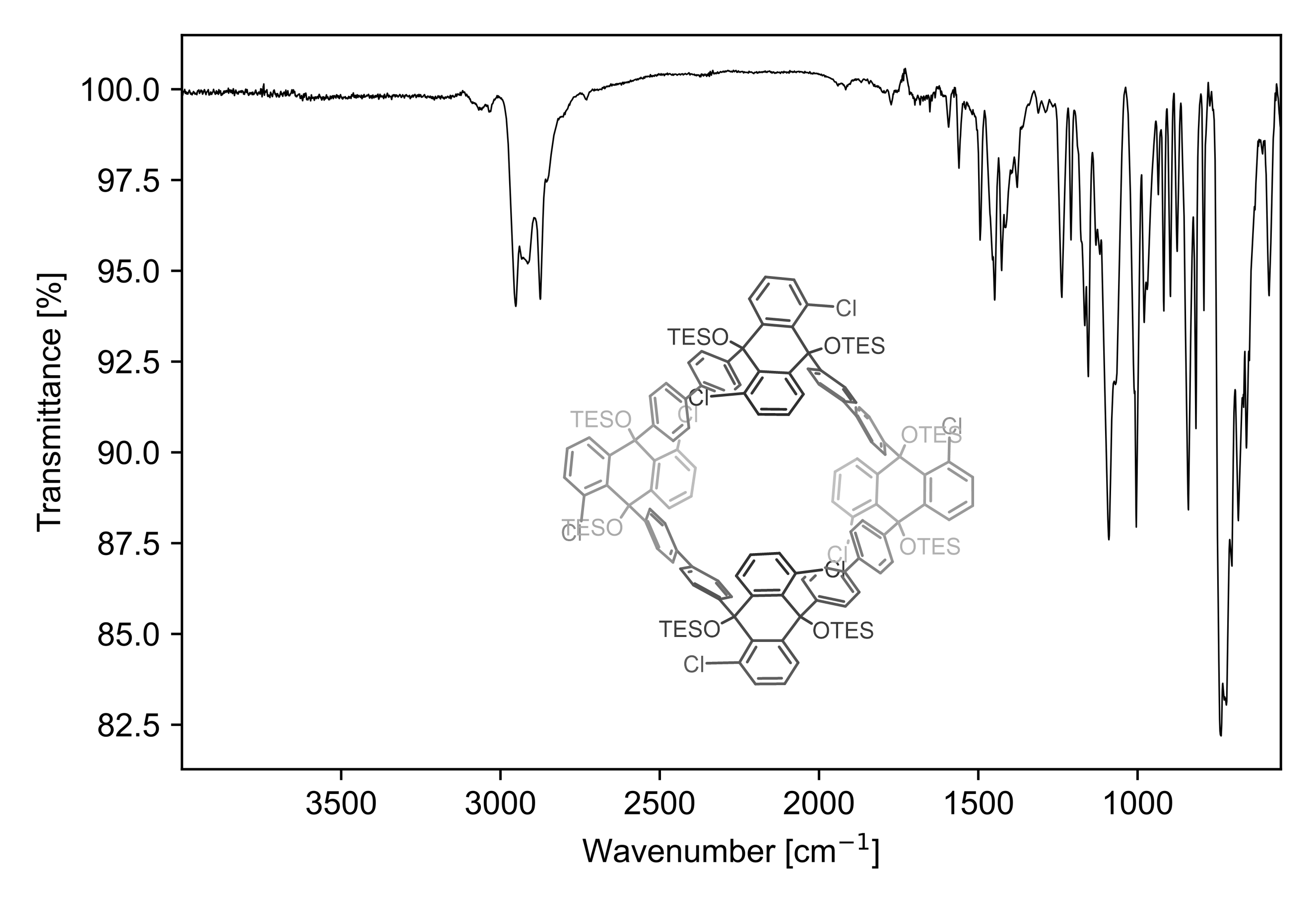


**Figure S56:** IR spectrum of (all-S)-**14** (ATR).


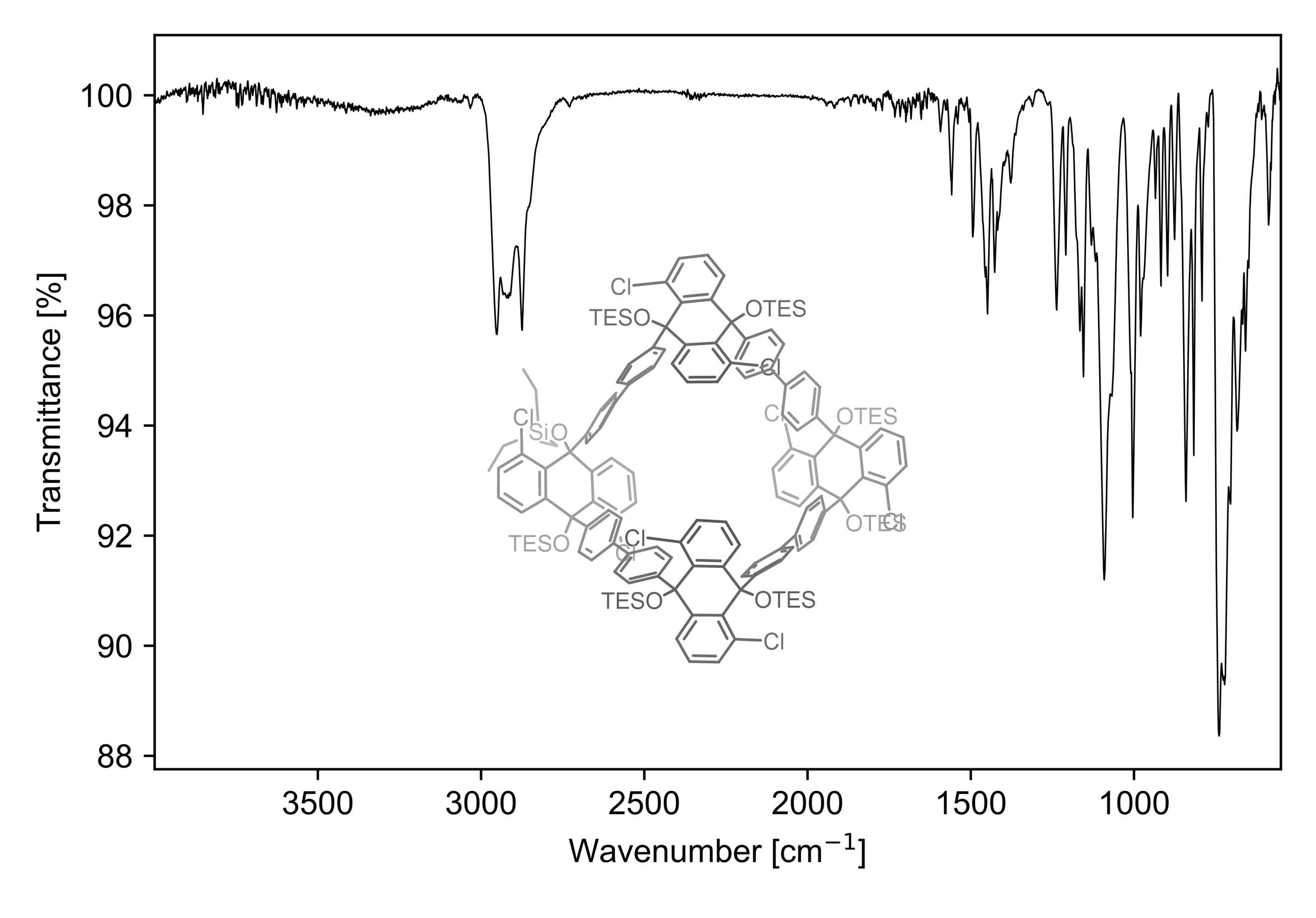


**Figure S57:** IR spectrum of (all-R)-**14** (ATR).


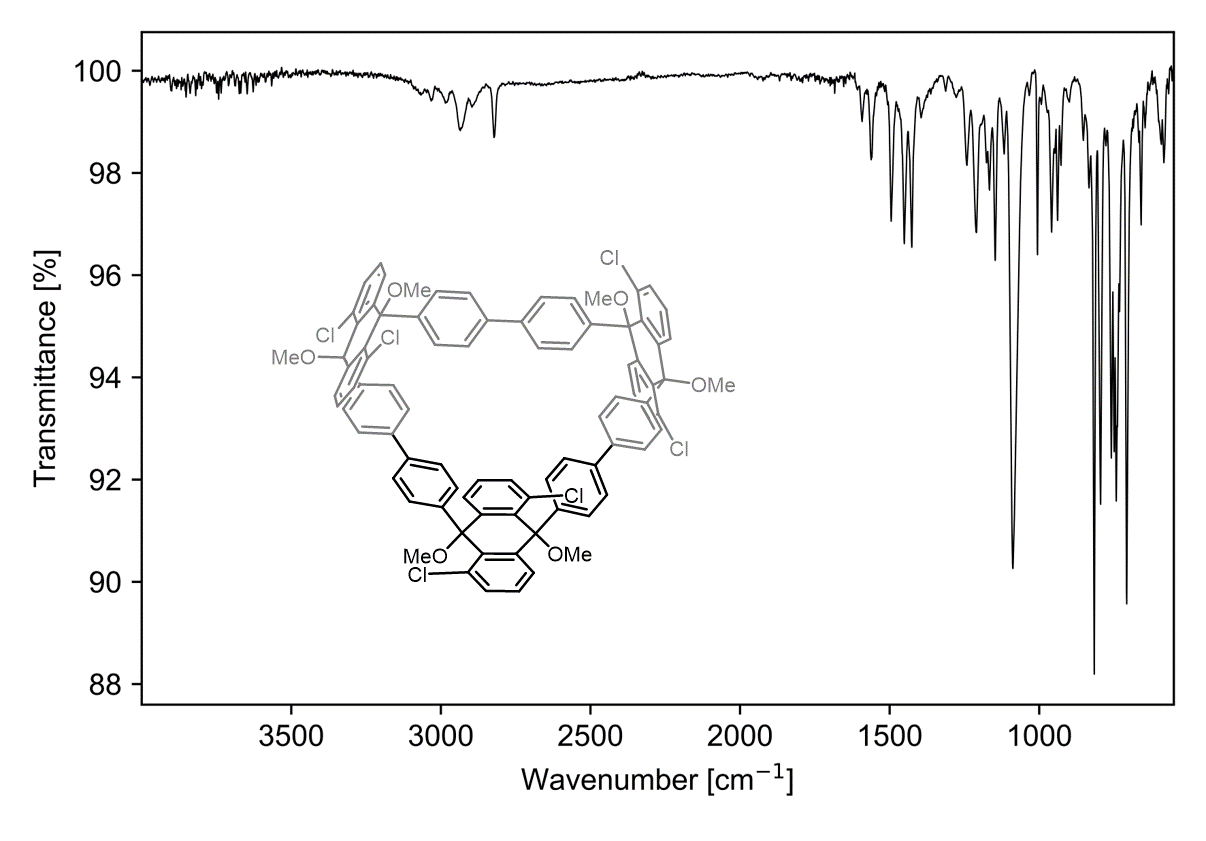


**Figure S58:** IR spectrum of (all-S)-**13** (ATR).


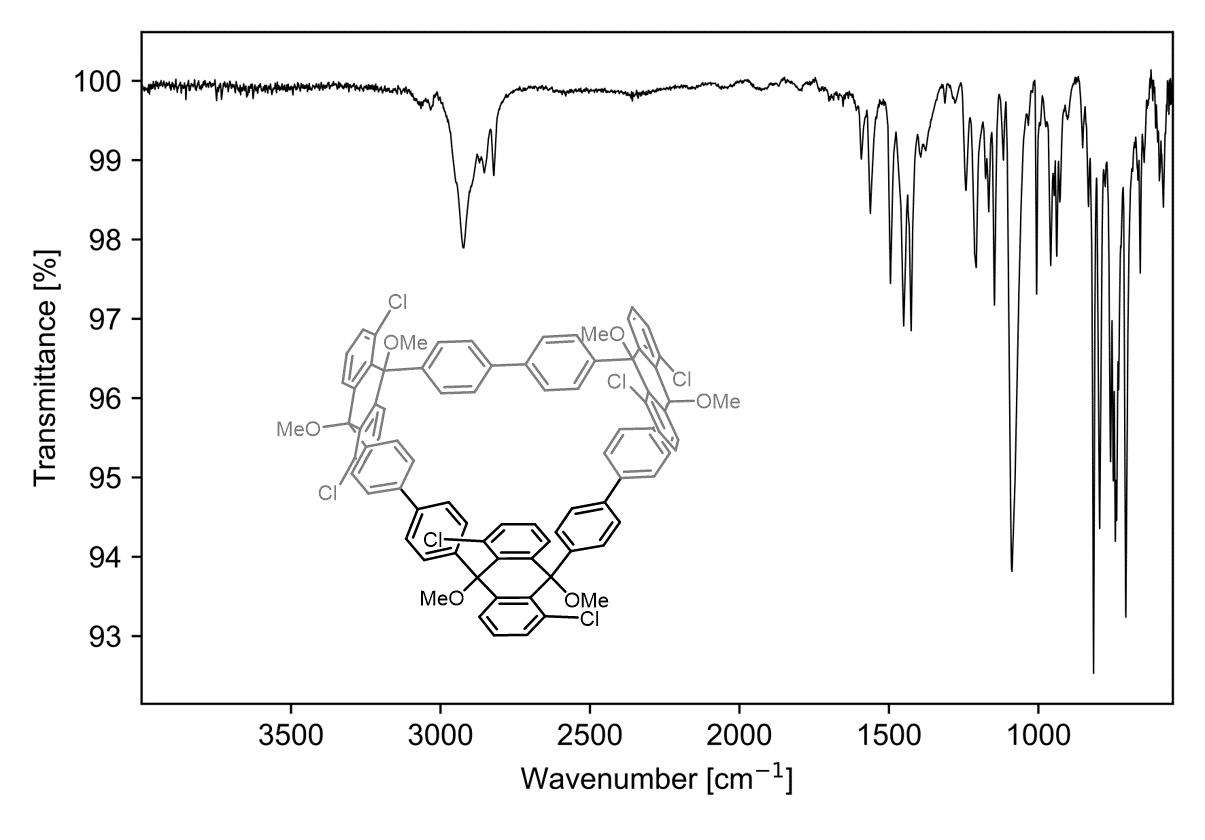


**Figure S59:** IR spectrum of (all-R)-**13** (ATR).


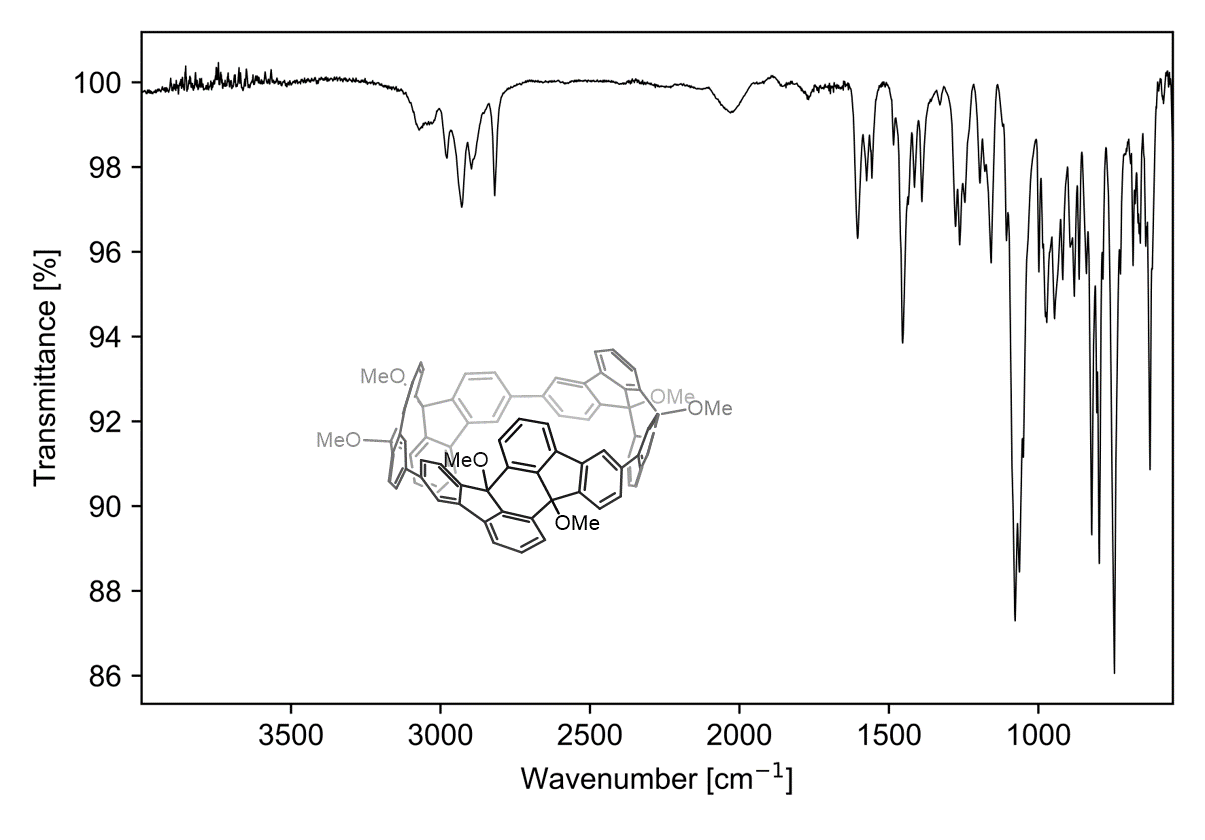


**Figure S60:** IR spectrum of (all-S)-**15** (ATR).


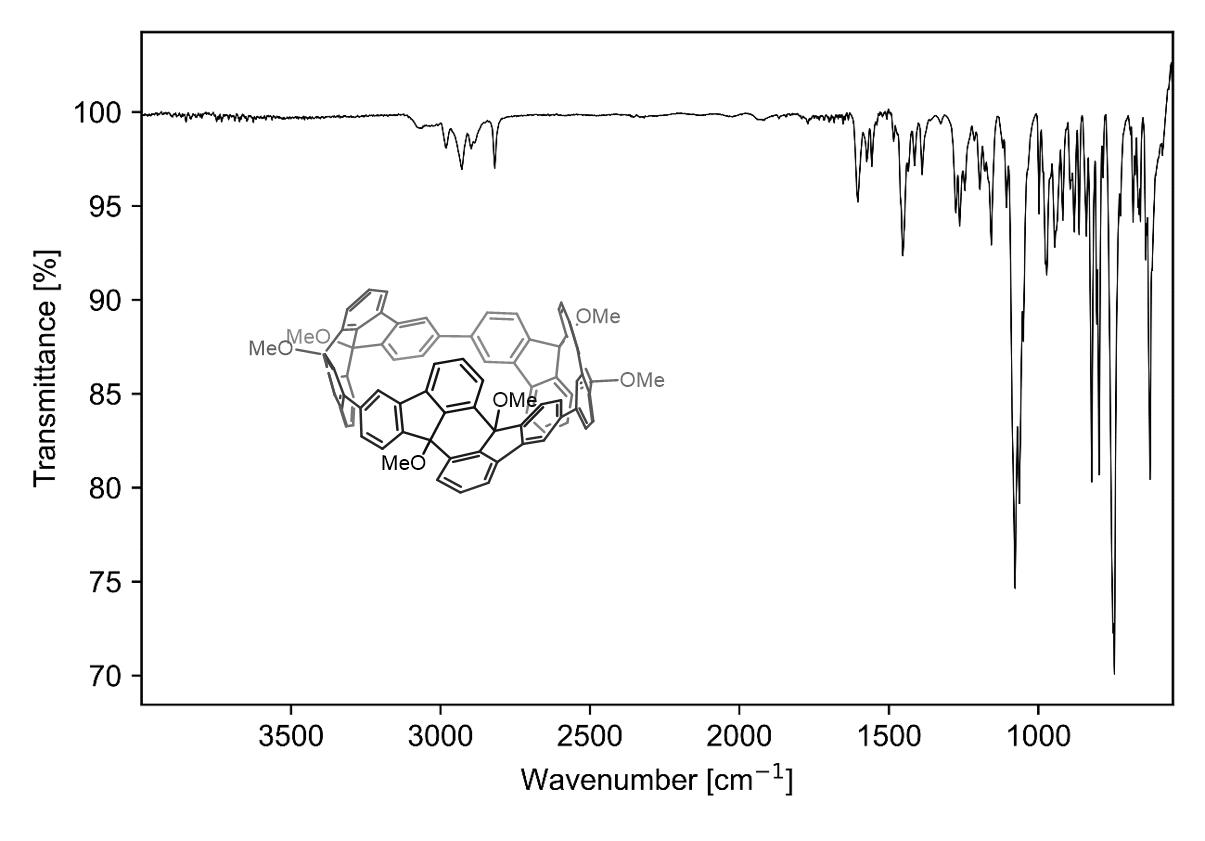


**Figure S61:** IR spectrum of (all-R)-**15** (ATR).


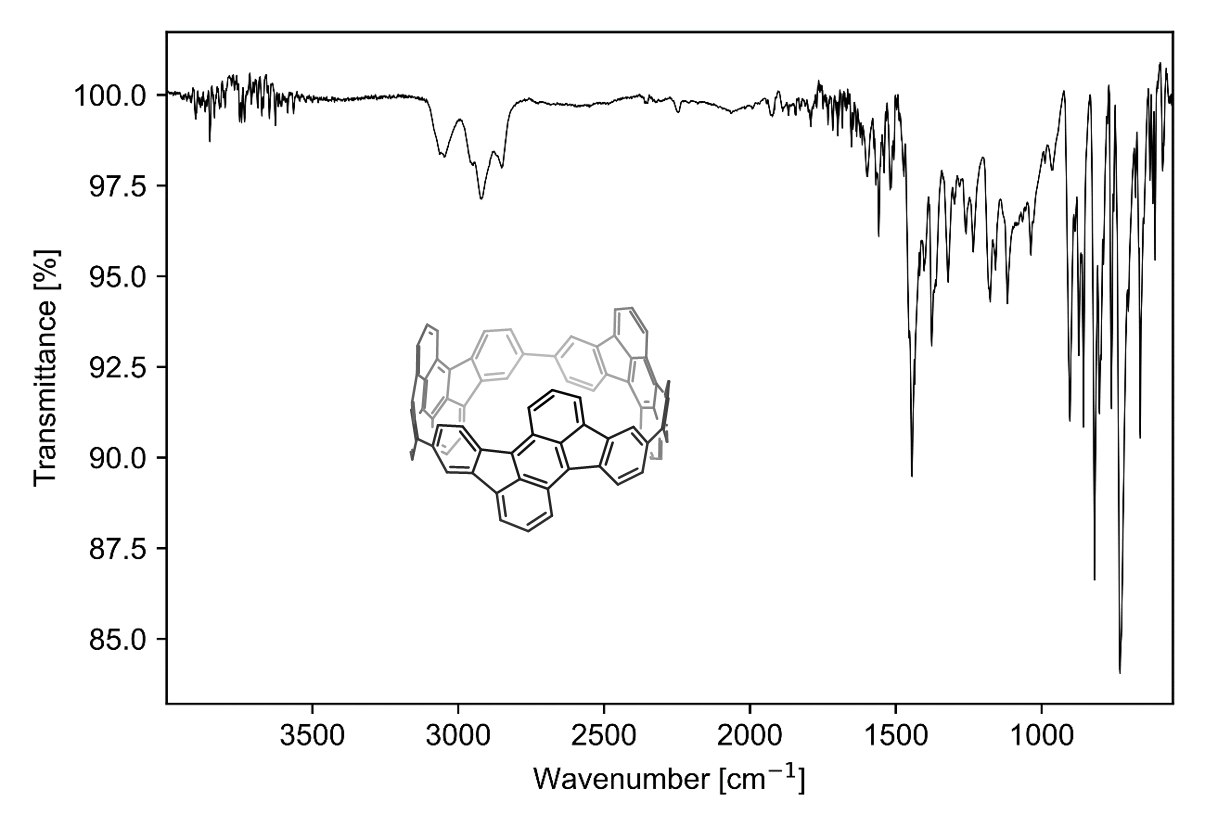


**Figure S62:** IR spectrum of (all-M)-**7** (ATR).


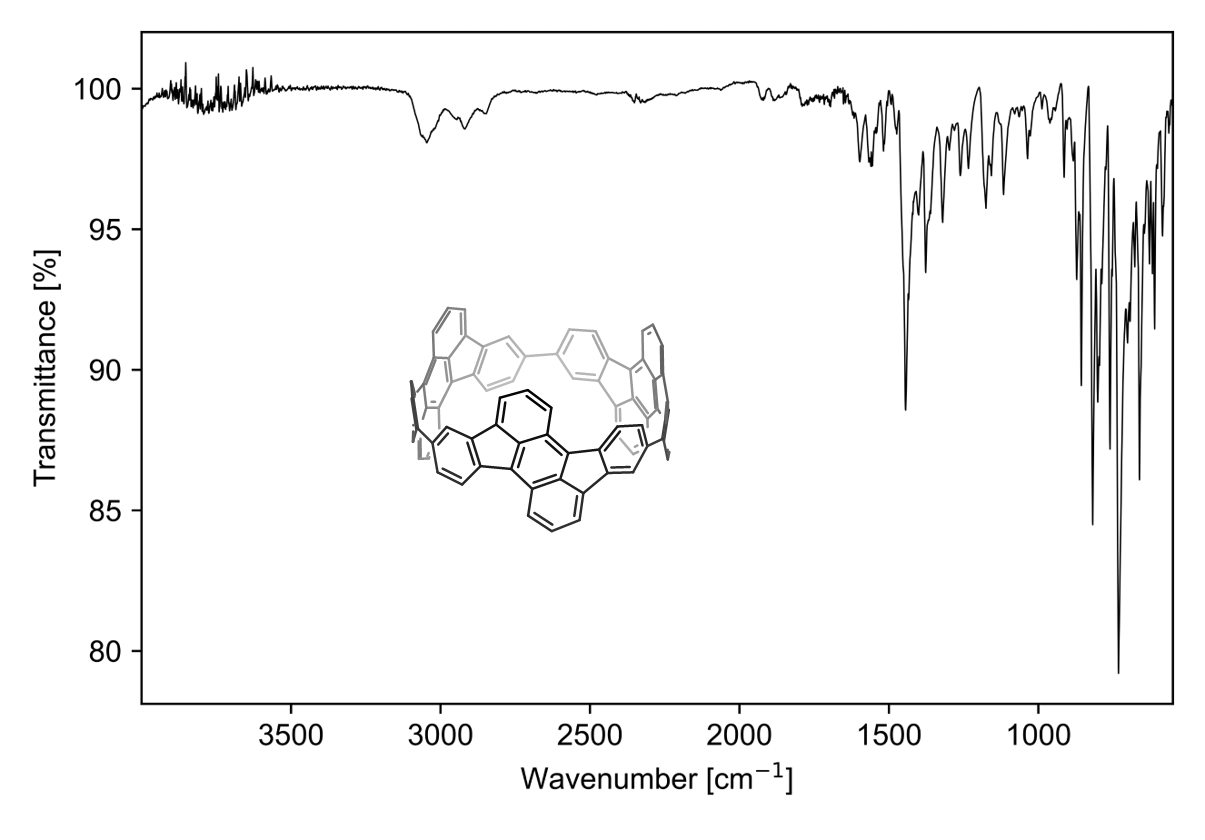


**Figure S63:** IR spectrum of (all-P)-**7** (ATR).

## Mass spectra


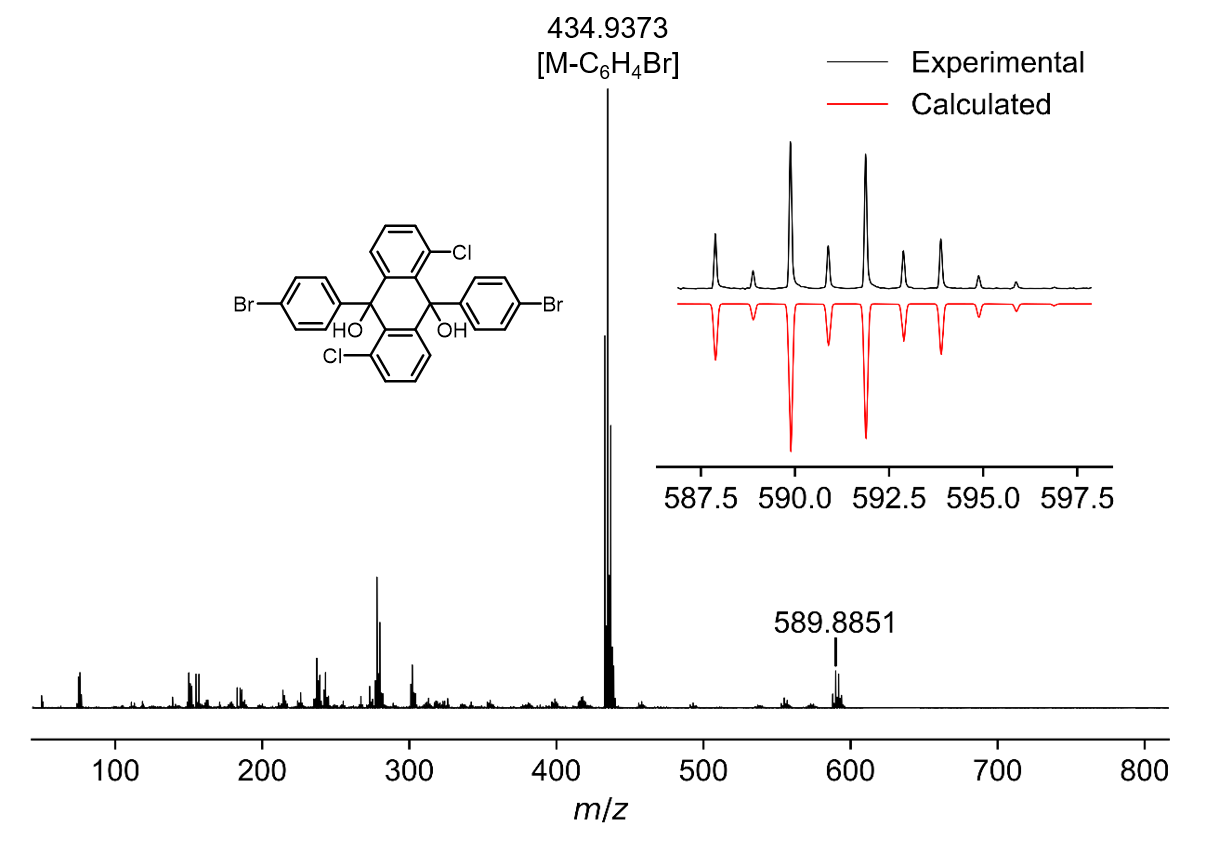


**Figure S64:** HR EI mass spectrum of **10**.


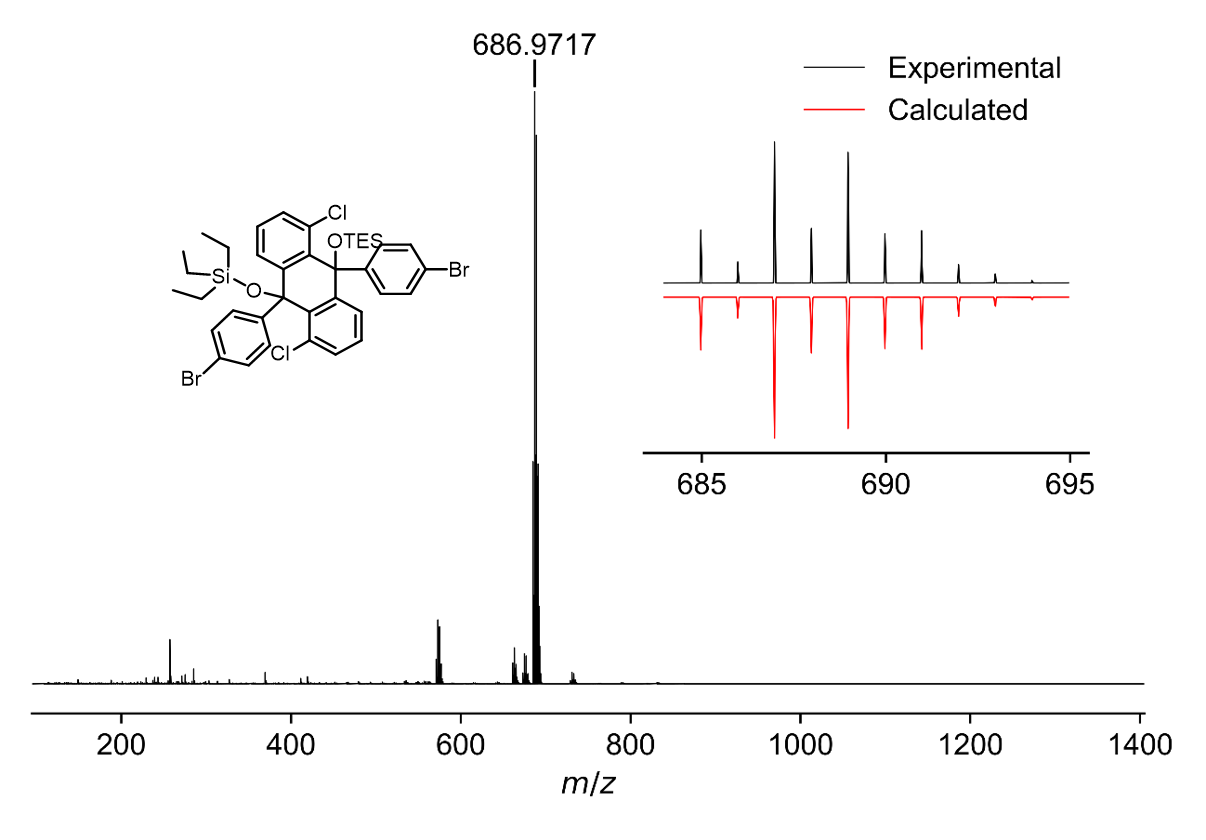


**Figure S65:** HR APCI mass spectrum of **11**.


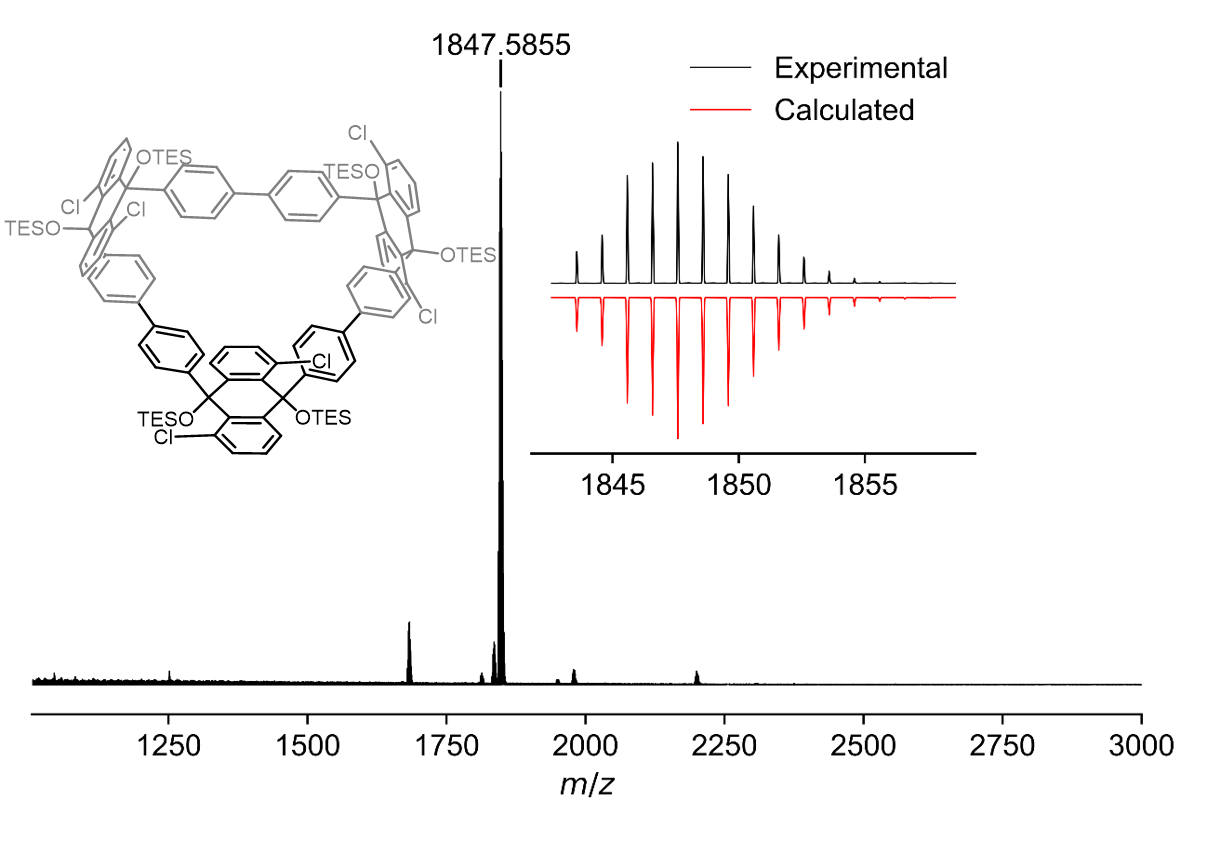


**Figure S66:** HR MALDI-TOF-MS of compound (all-S)-**12**.


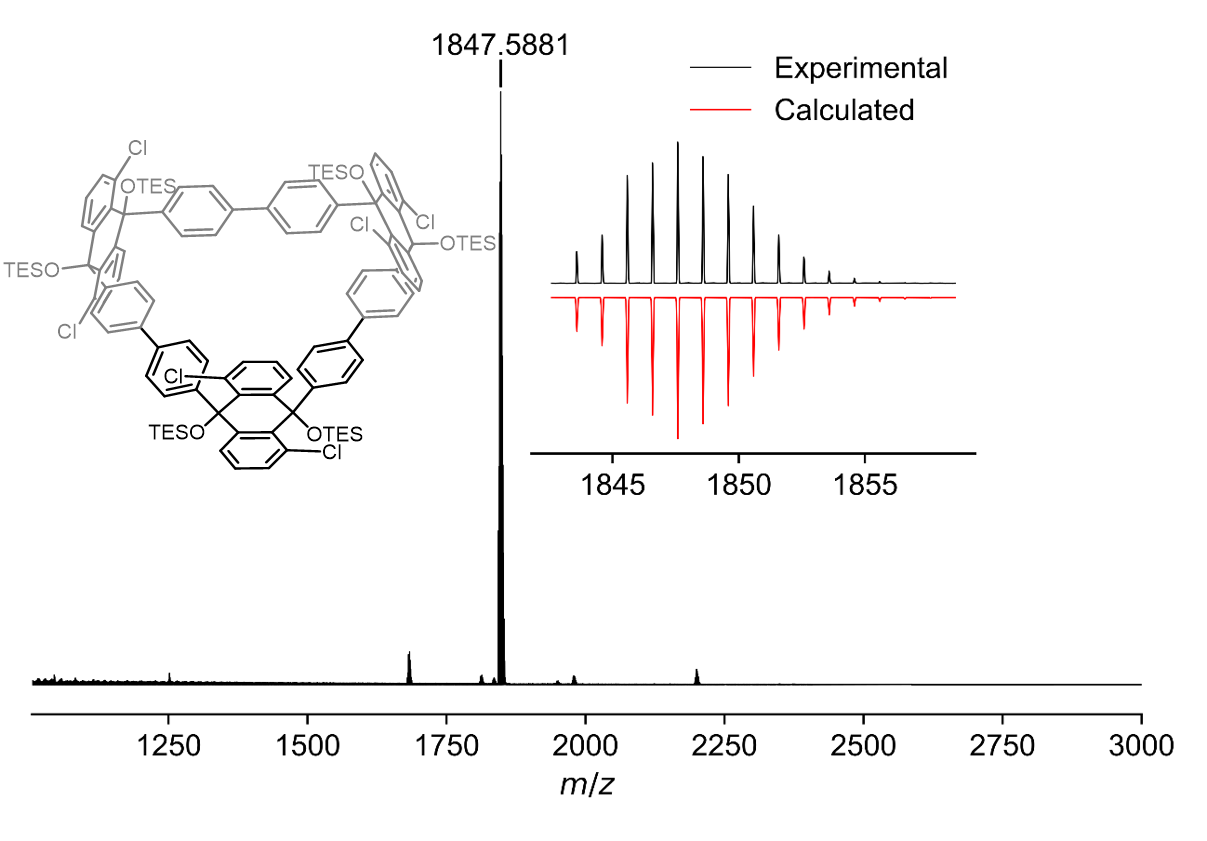


**Figure S67:** HR MALDI-TOF-MS of compound (all-R)-**12**.


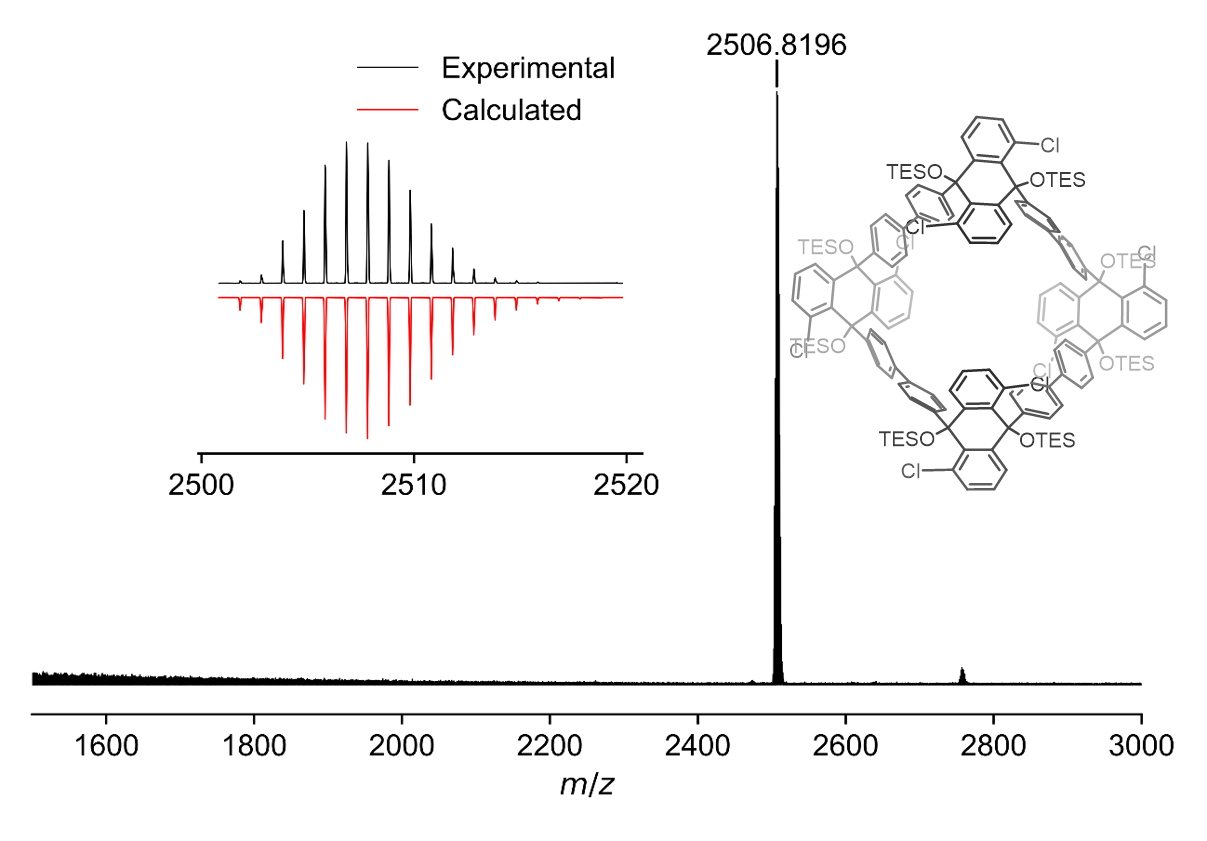


**Figure S68:** HR MALDI-TOF-MS of compound (all-S)-**14**.


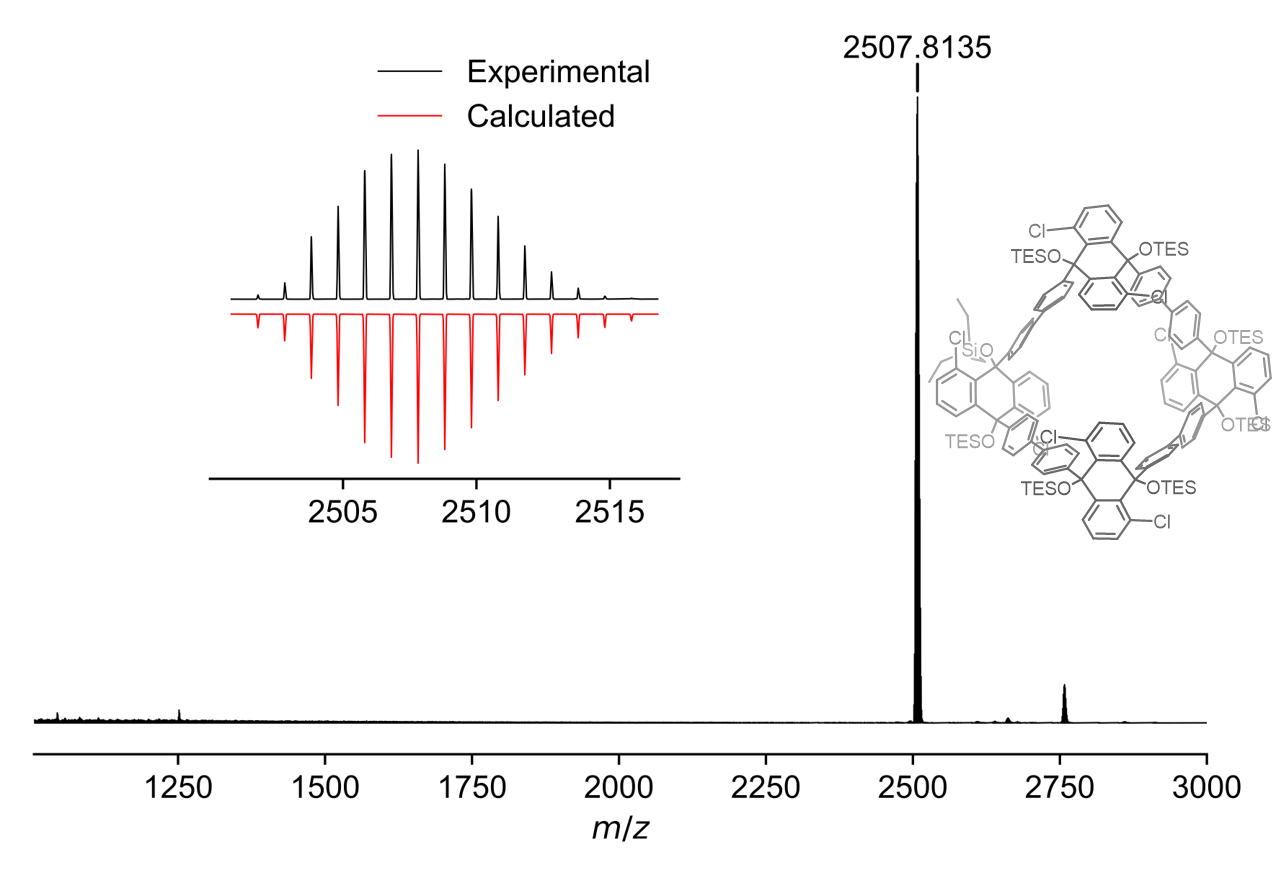


**Figure S69:** HR MALDI-TOF-MS of compound (all-R)-**14**.


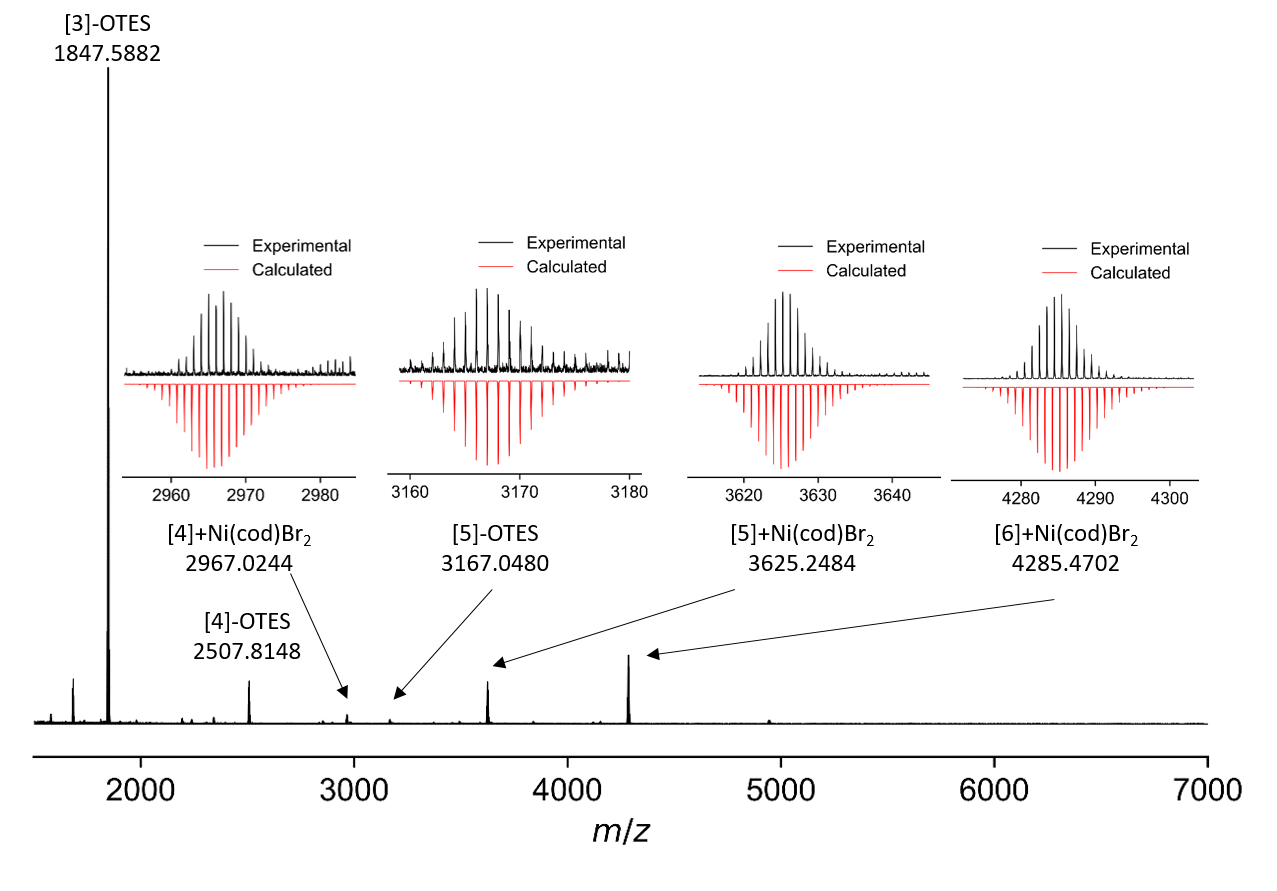


**Figure S70:** HR MALDI-TOF-MS of the macrocyclization reaction.


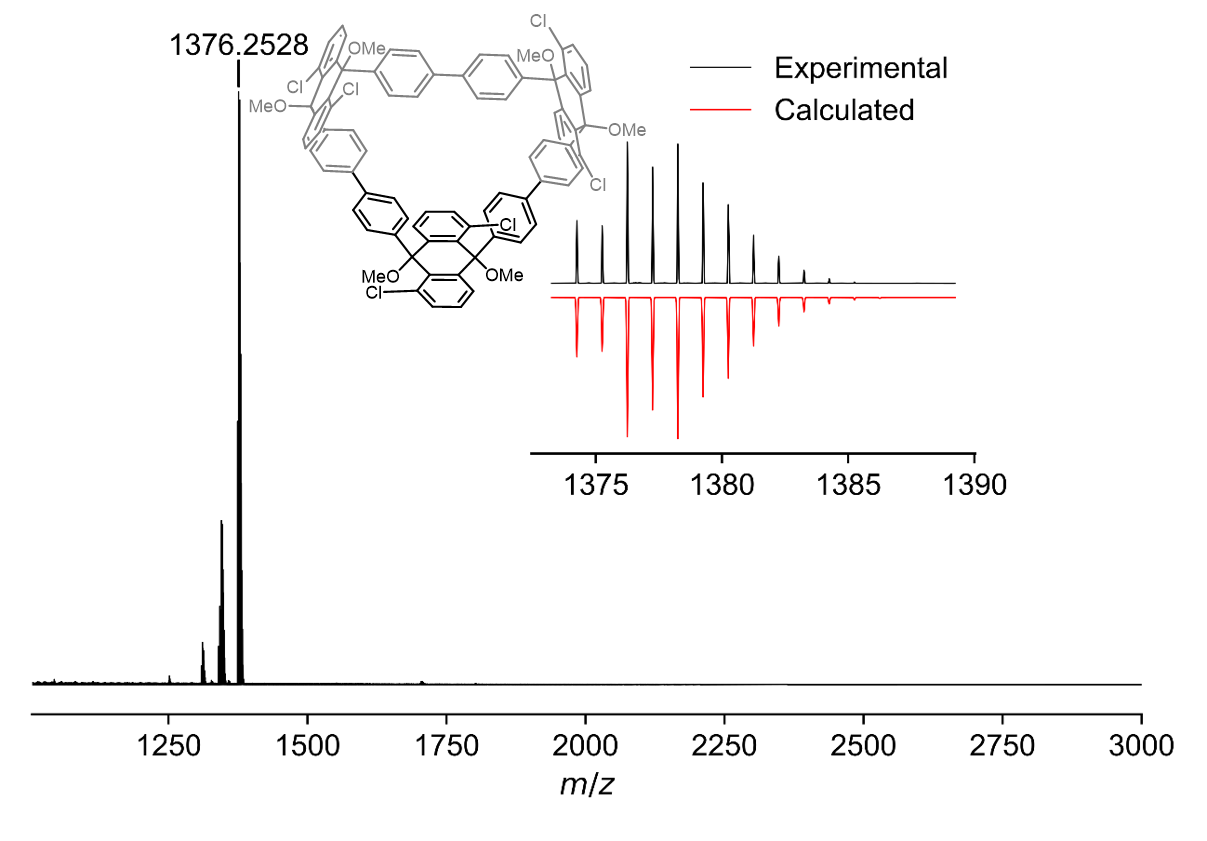


**Figure S71:** HR MALDI-TOF-MS of compound (all-S)-**13**.


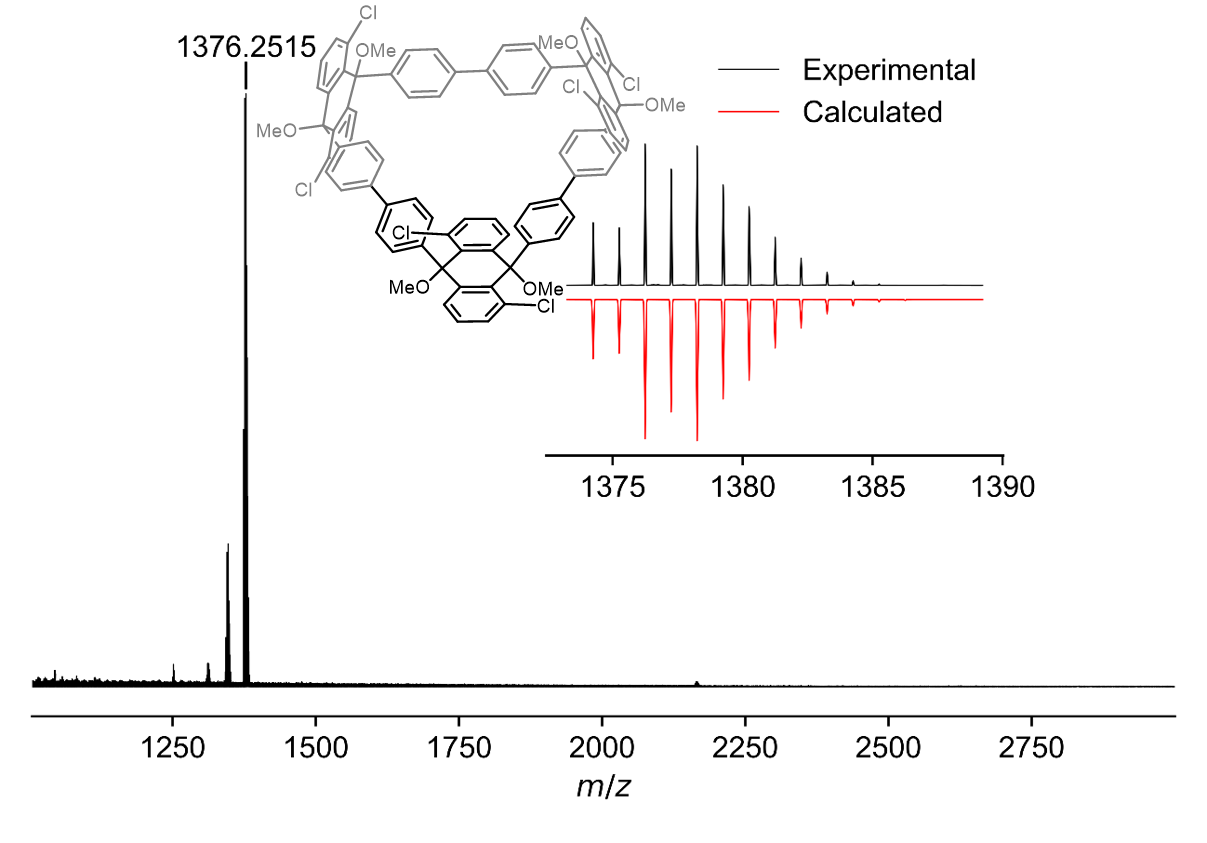


**Figure S72:** HR MALDI-TOF-MS of compound (all-R)-**13**.


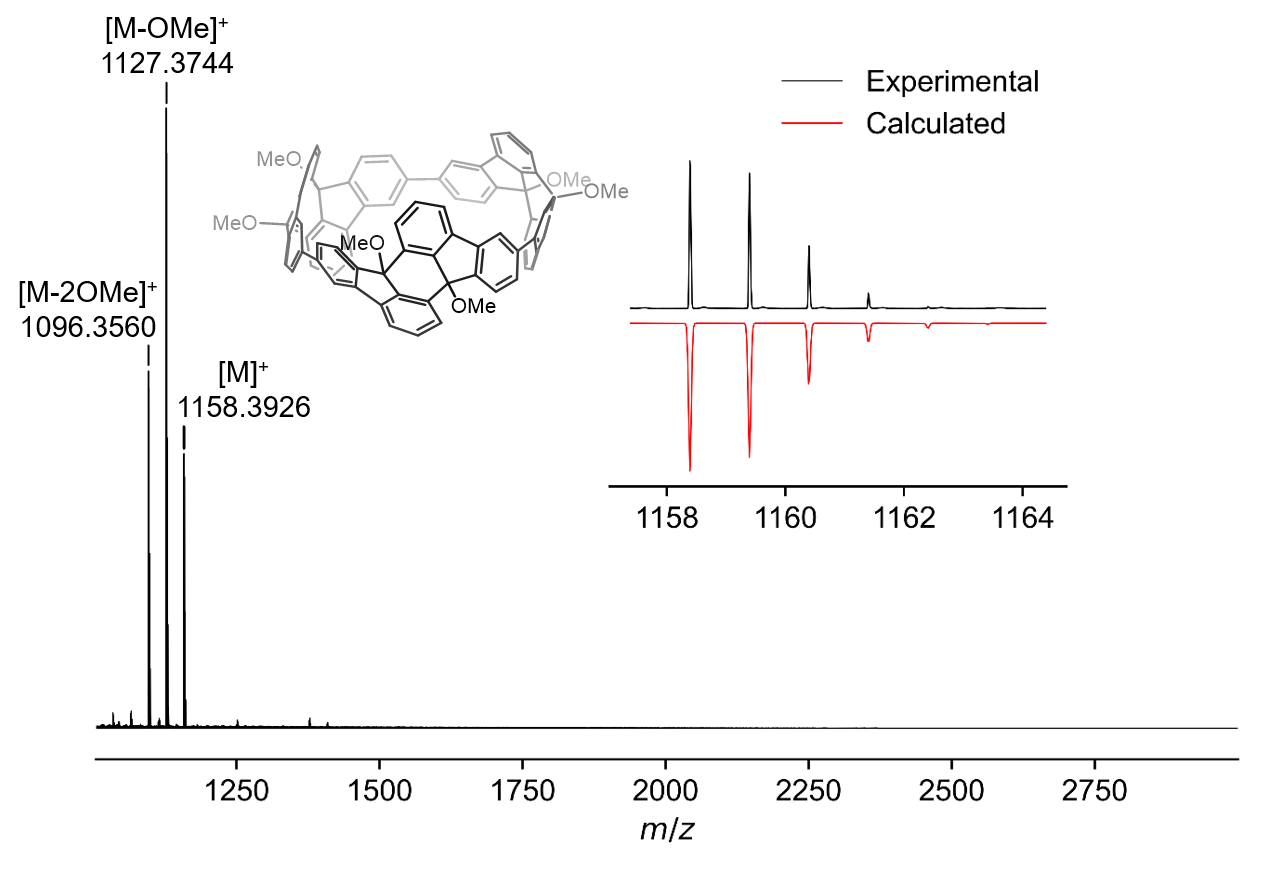


**Figure S73:** HR MALDI-TOF-MS of compound (all-S)-**15**.


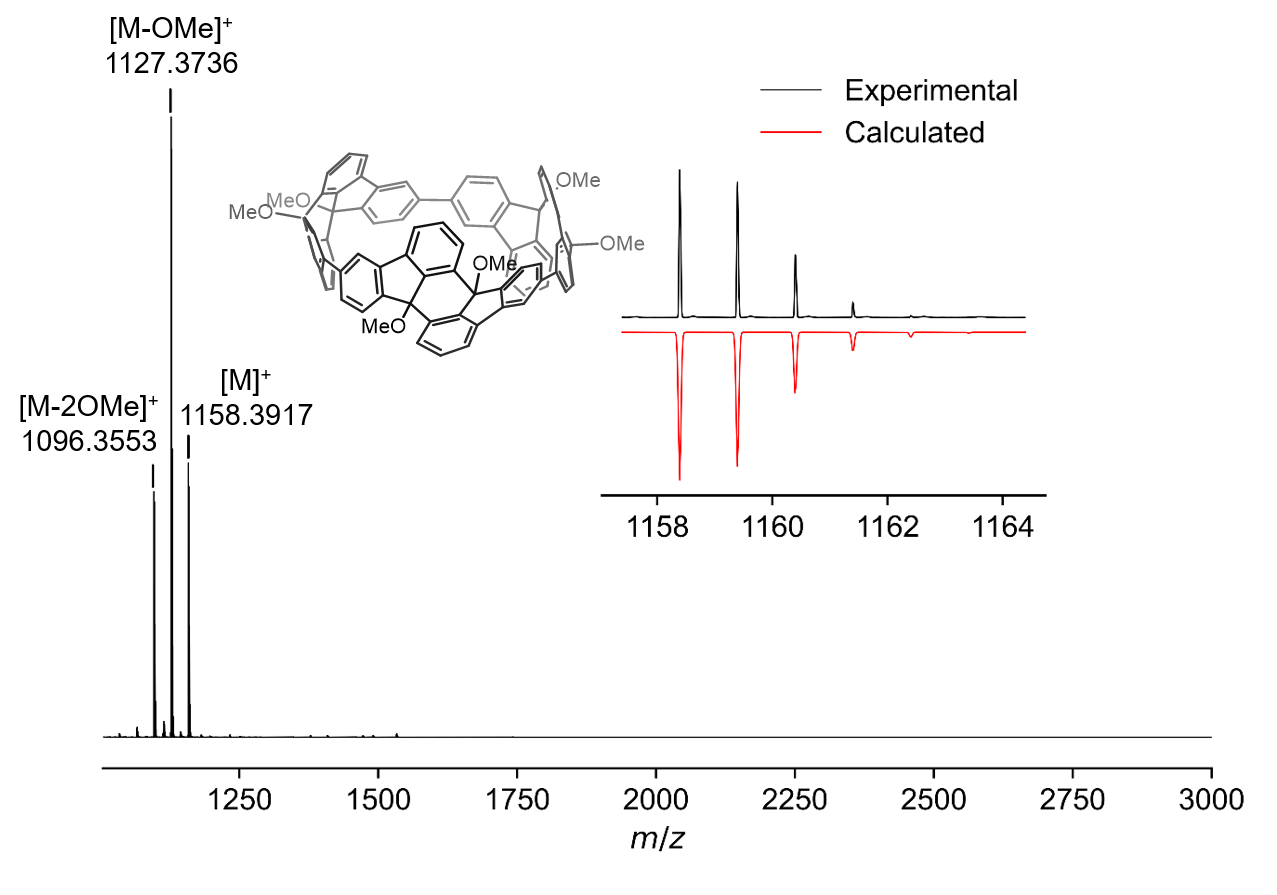


**Figure S74:** HR MALDI-TOF-MS of compound (all-R)-**15**.


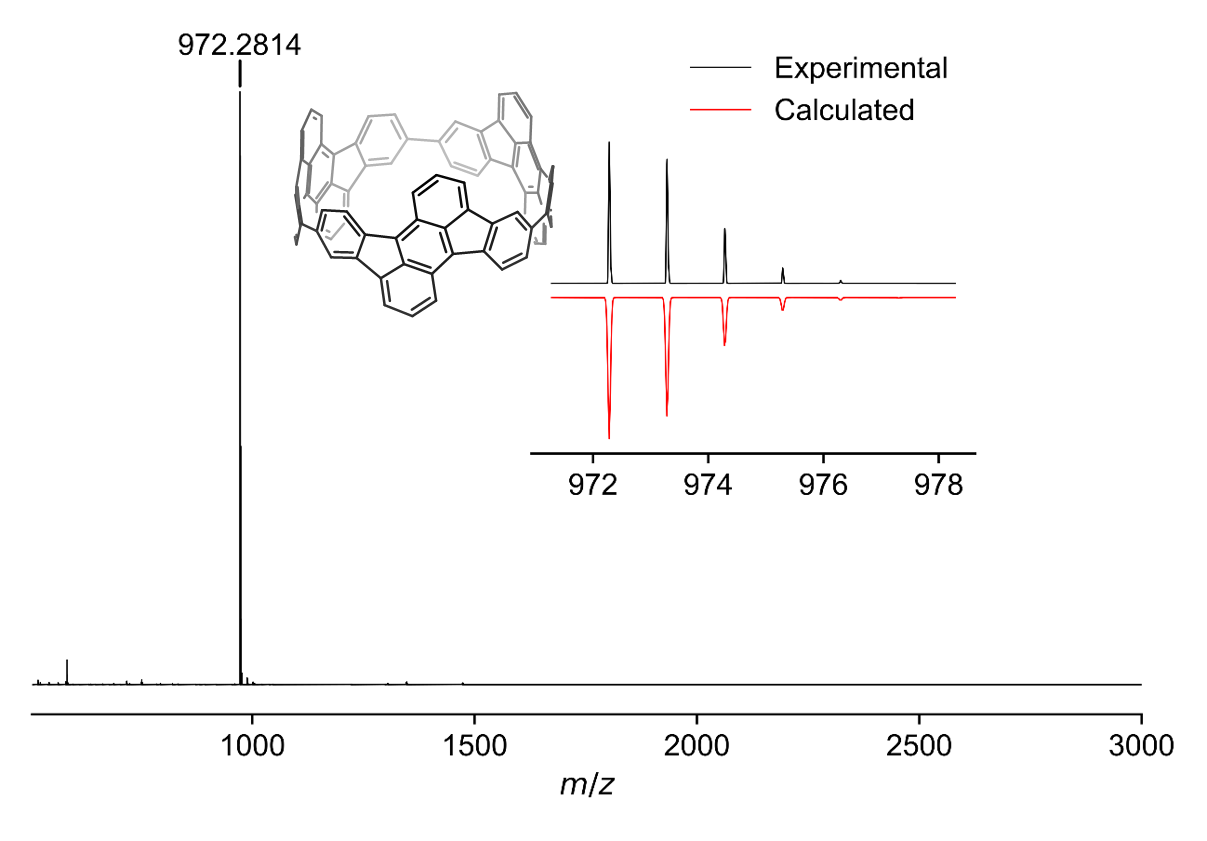


**Figure S75:** HR MALDI-TOF-MS of compound (*all-M*)-***7***.


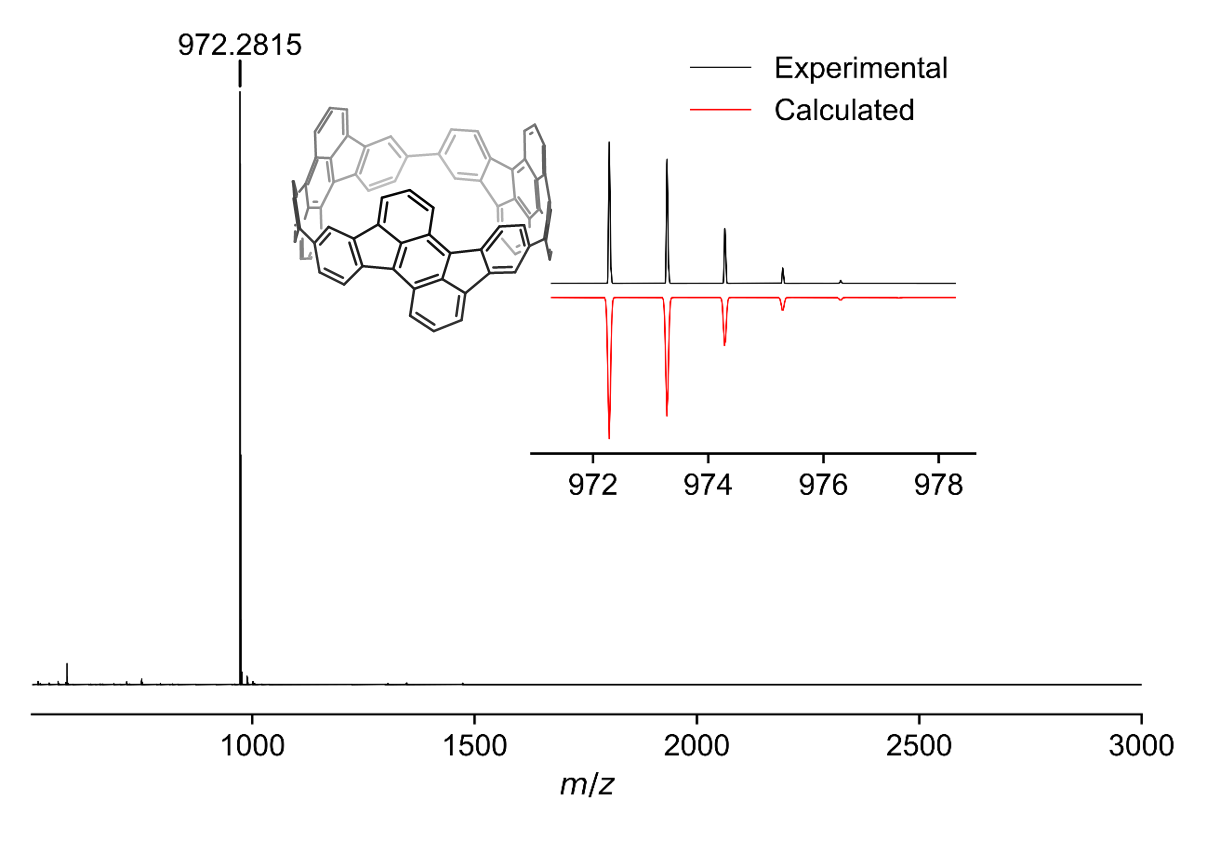


**Figure S76:** HR MALDI-TOF-MS of compound (all-P)-**7**.

## UV/Vis and fluorescence spectra


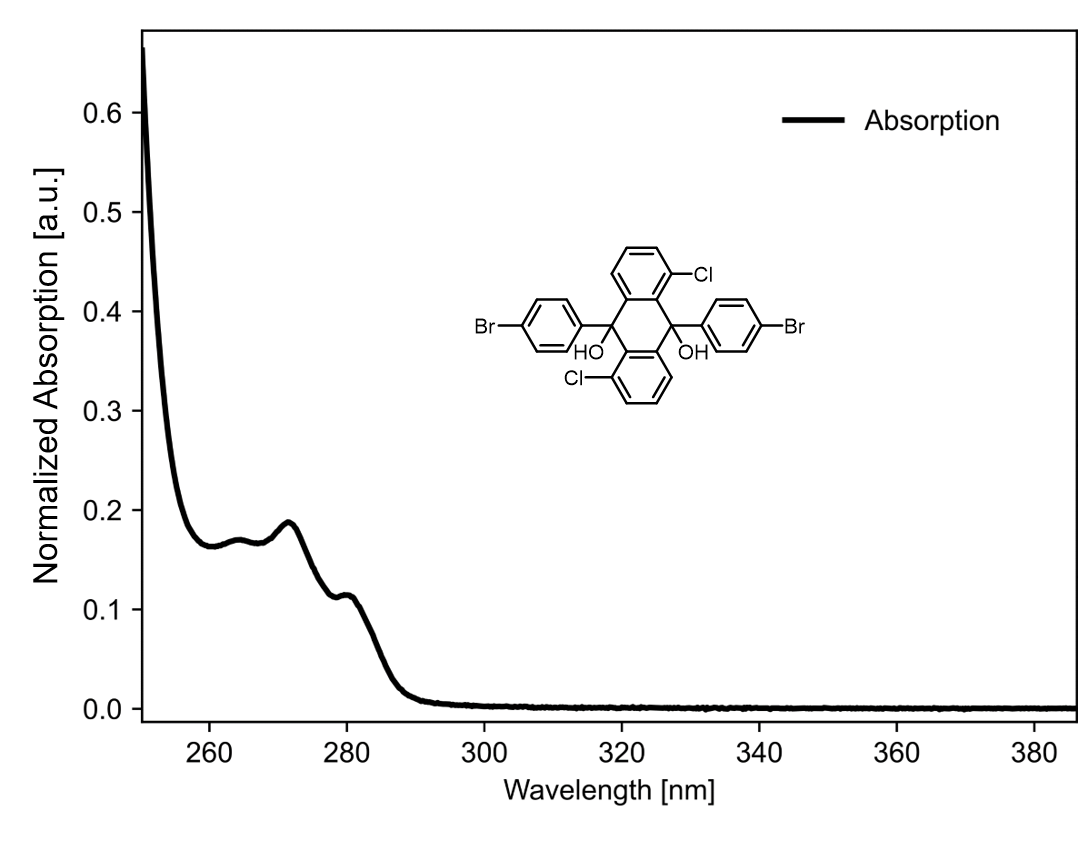


**Figure S77:** UV/Vis spectrum of **10** in dichloromethane.


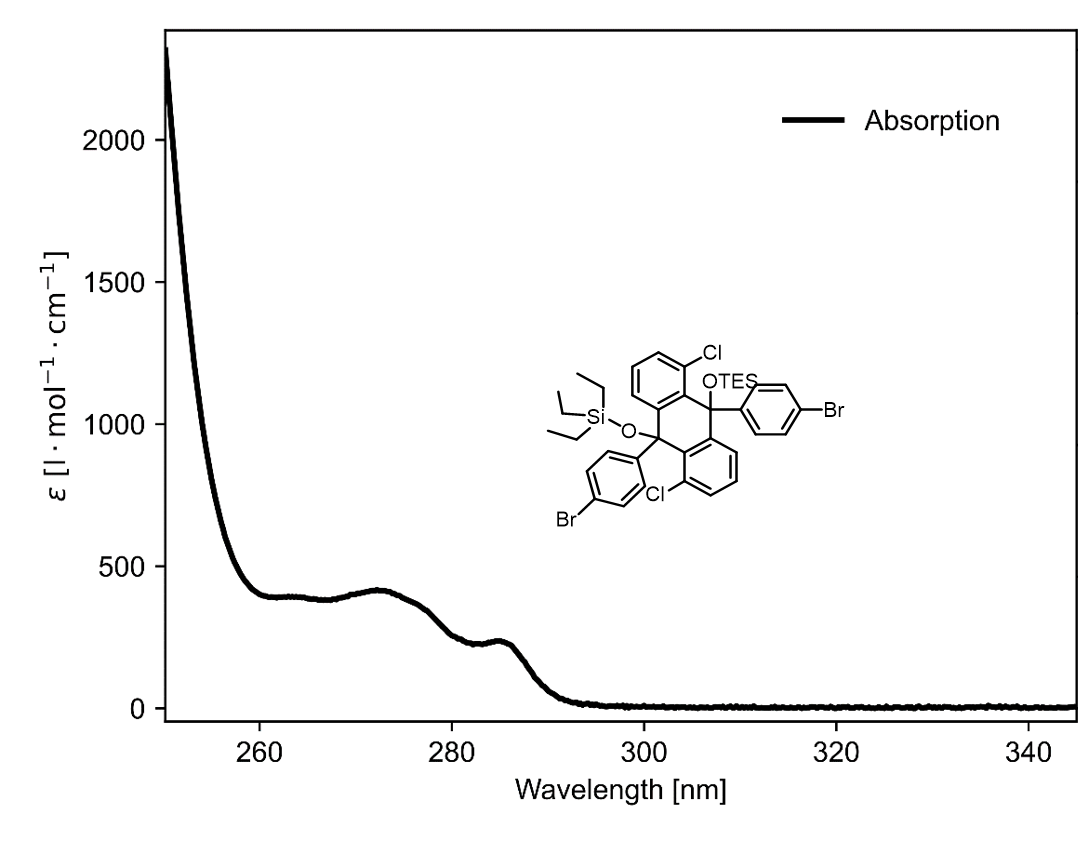


**Figure S78:** UV/Vis spectrum of **11** in dichloromethane.


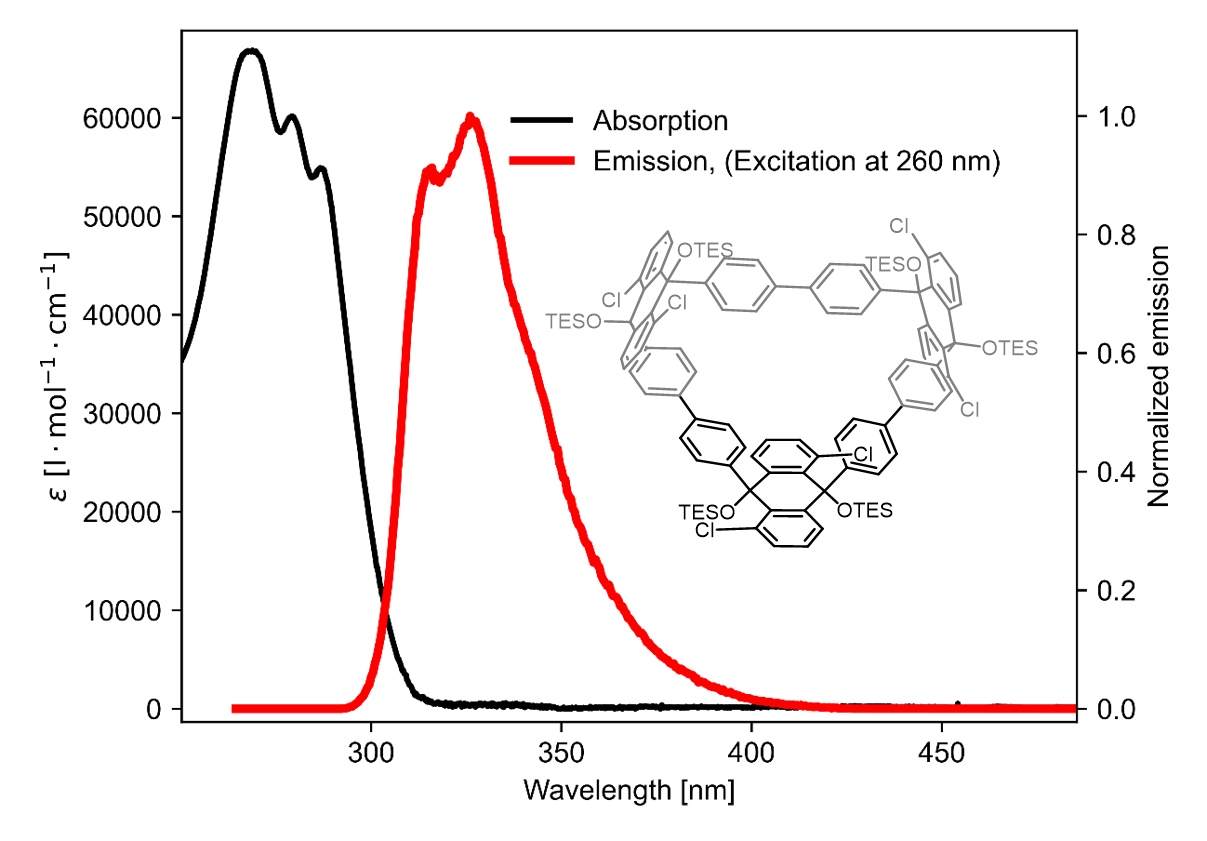


**Figure S79:** UV/Vis and fluorescence spectra of (all-S)-**12** in dichloromethane.


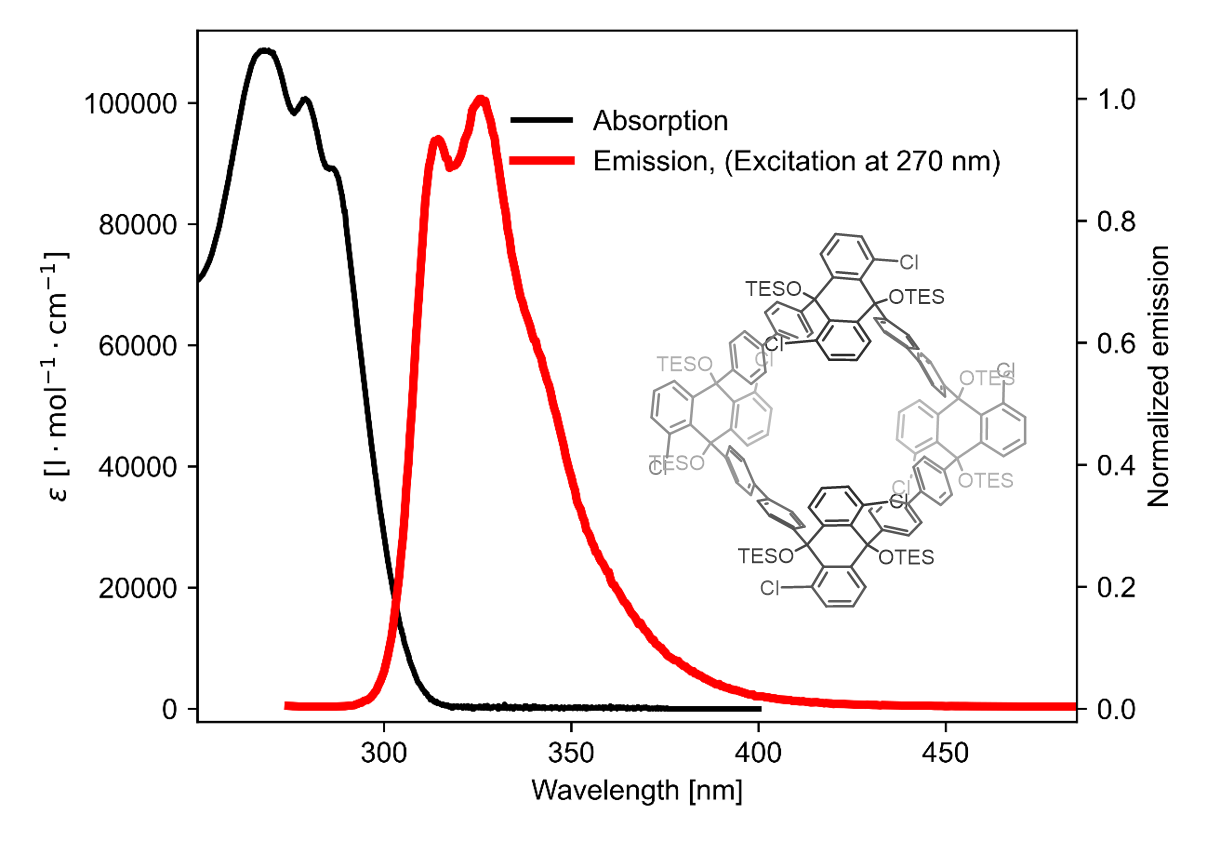


**Figure S80:** UV/Vis and fluorescence spectra of (all-S)-**14** in dichloromethane.


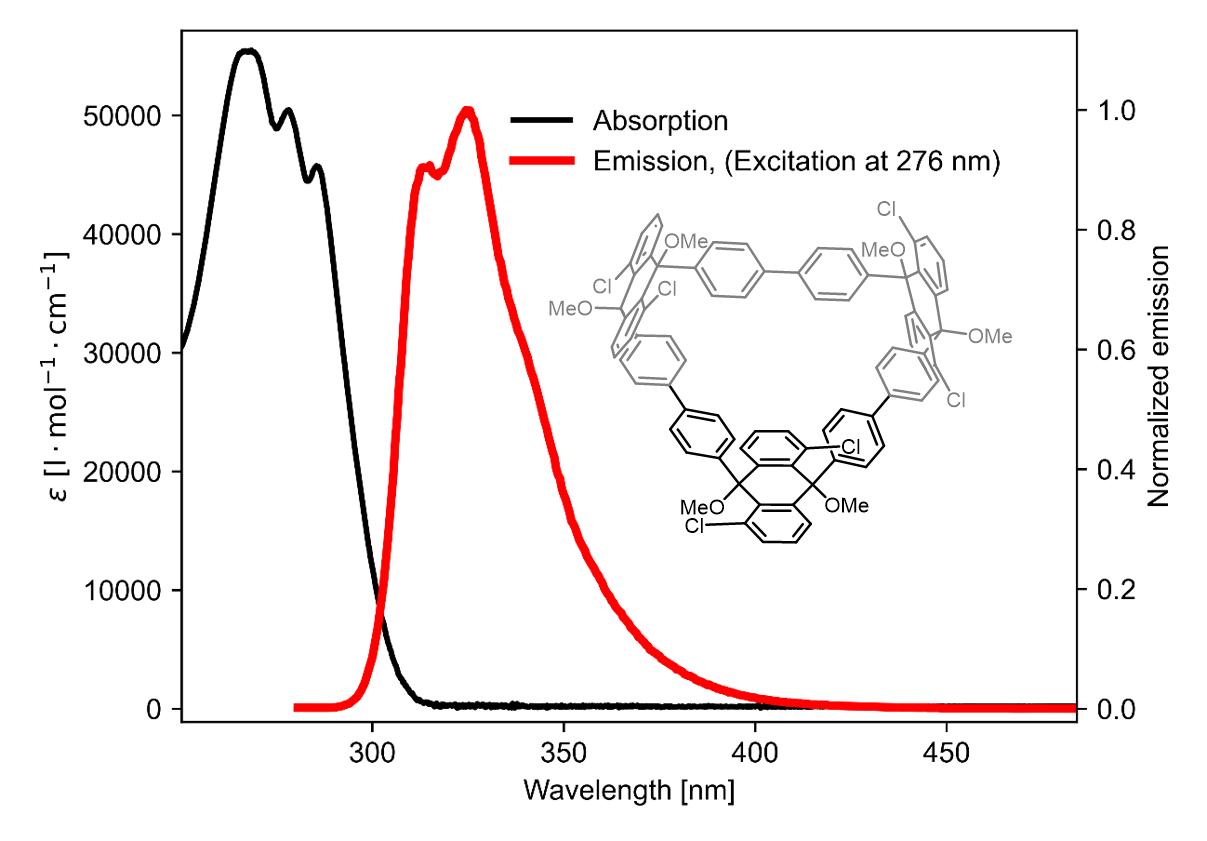


**Figure S81:** UV/Vis and fluorescence spectra of (all-S)-**13** in dichloromethane.


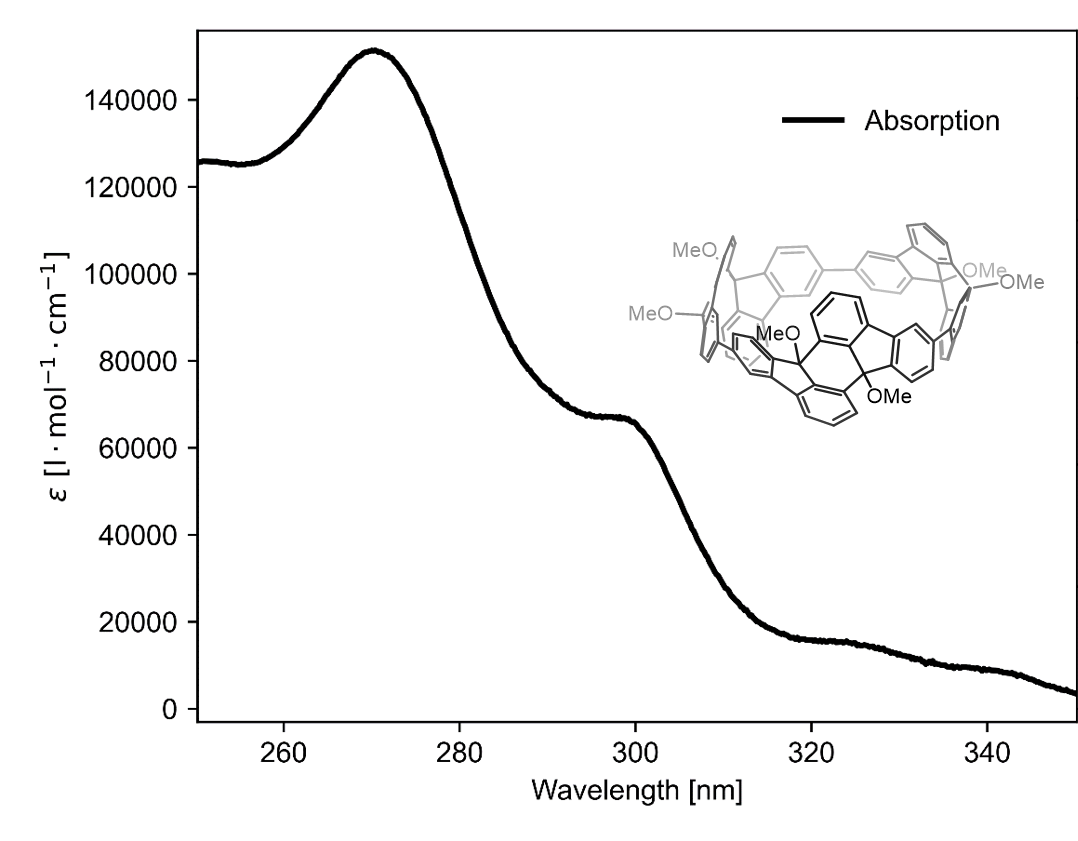


**Figure S82:** UV/Vis spectrum of (all-S)-**15** in dichloromethane.


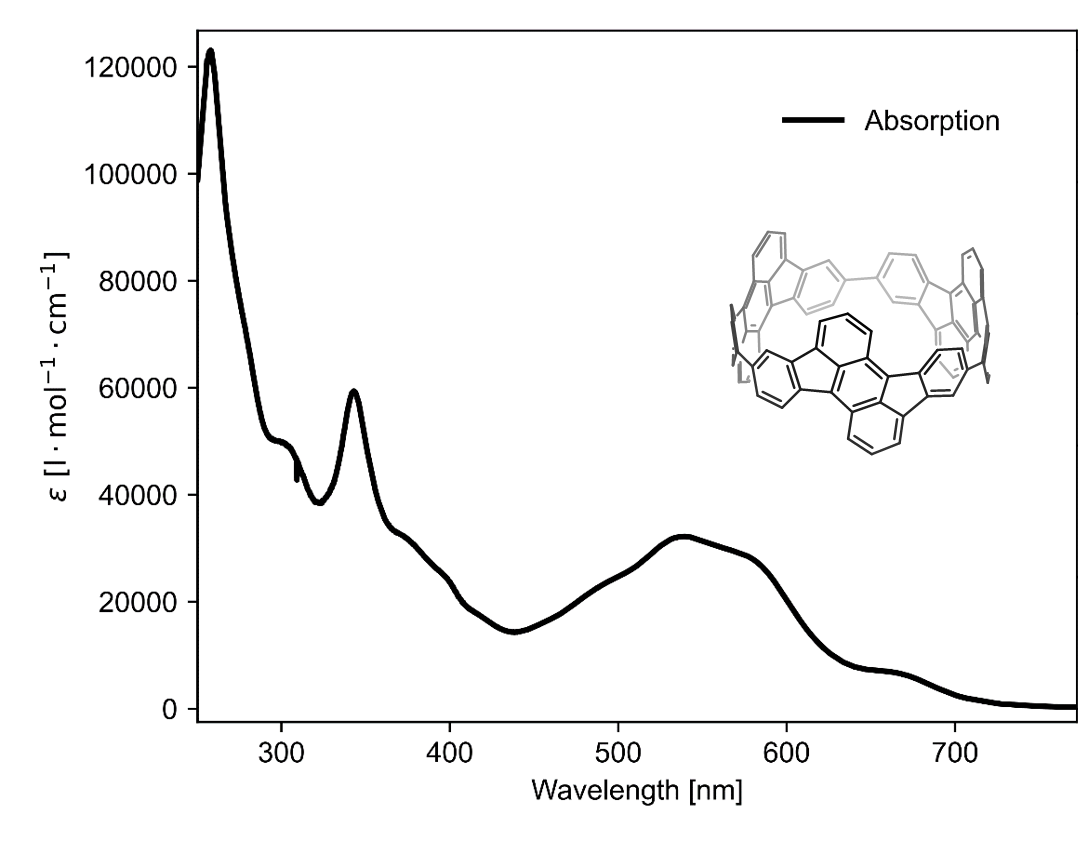


**Figure S83:** UV/Vis spectrum of (all-M)-**7** in dichloromethane.

## CD spectra


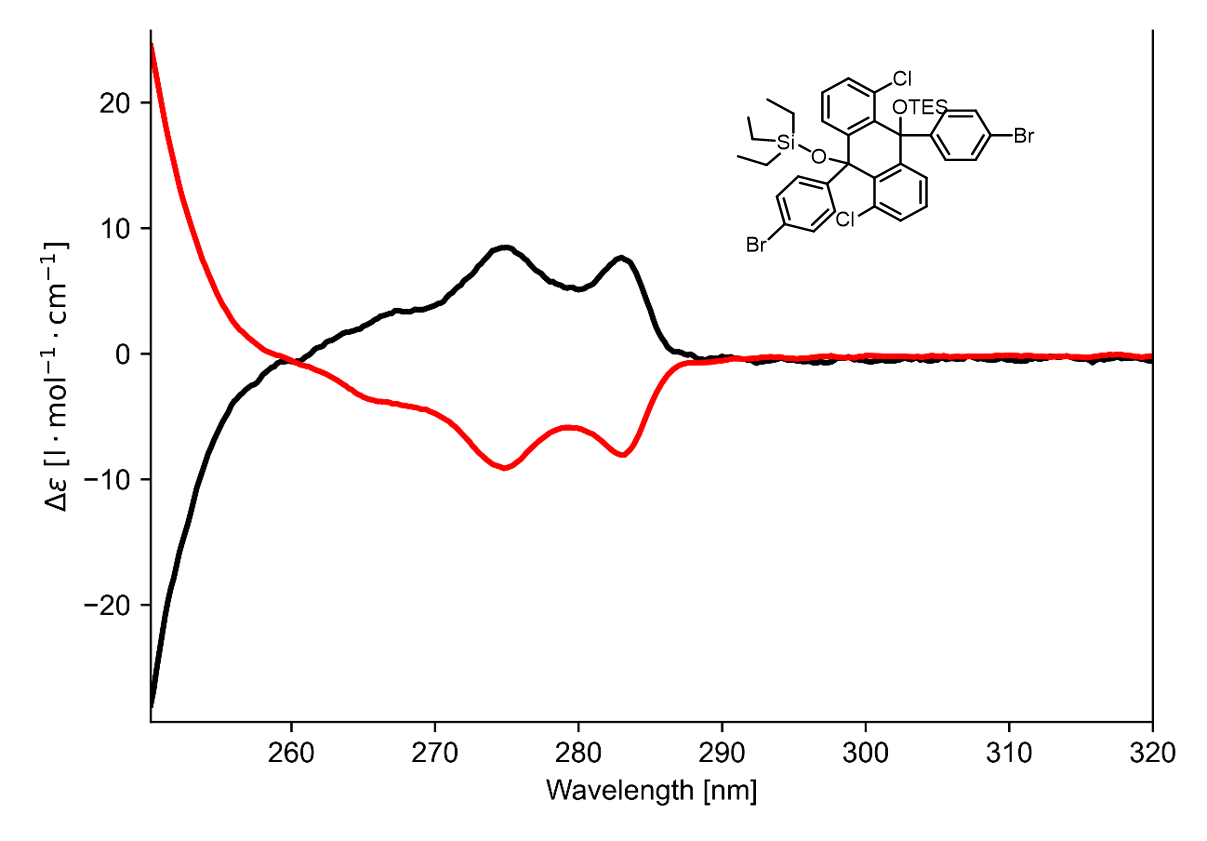


**Figure S84:** CD spectrum of **(*R,R*)-** (red) and **(*S,S*)-****11** (black) in dichloromethane.


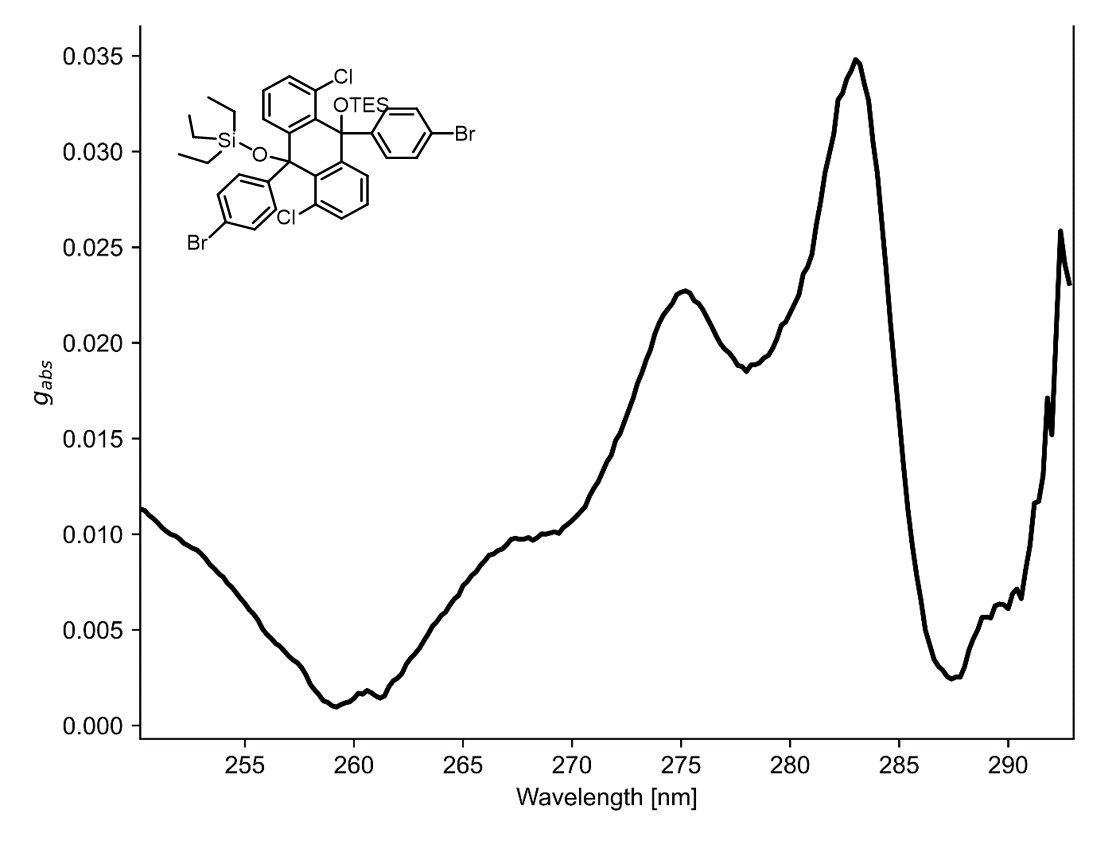


**Figure S85:** g_abs_ profile of **11** in dichloromethane.


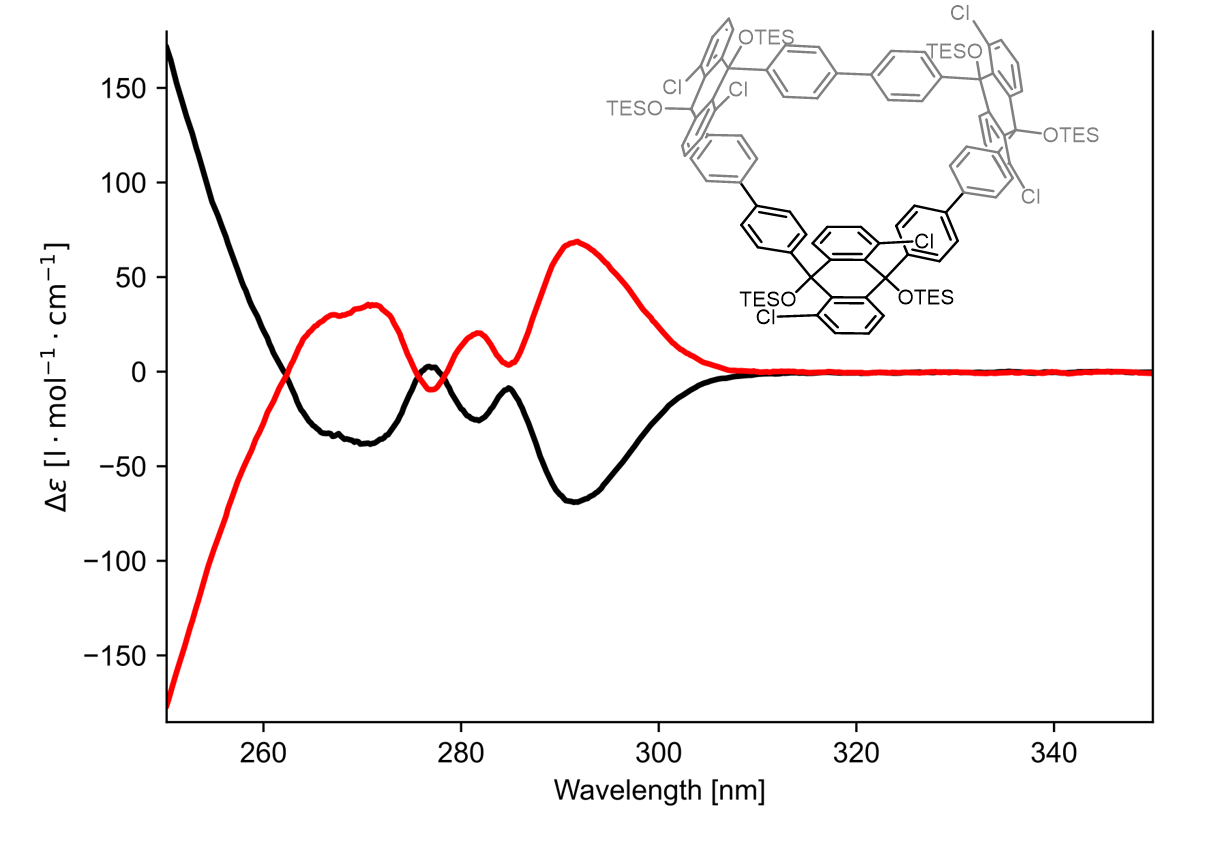


**Figure S86:** CD spectrum of (*all-R*)*-* (red) and (*all-S*)*-***12** (black) in dichloromethane.


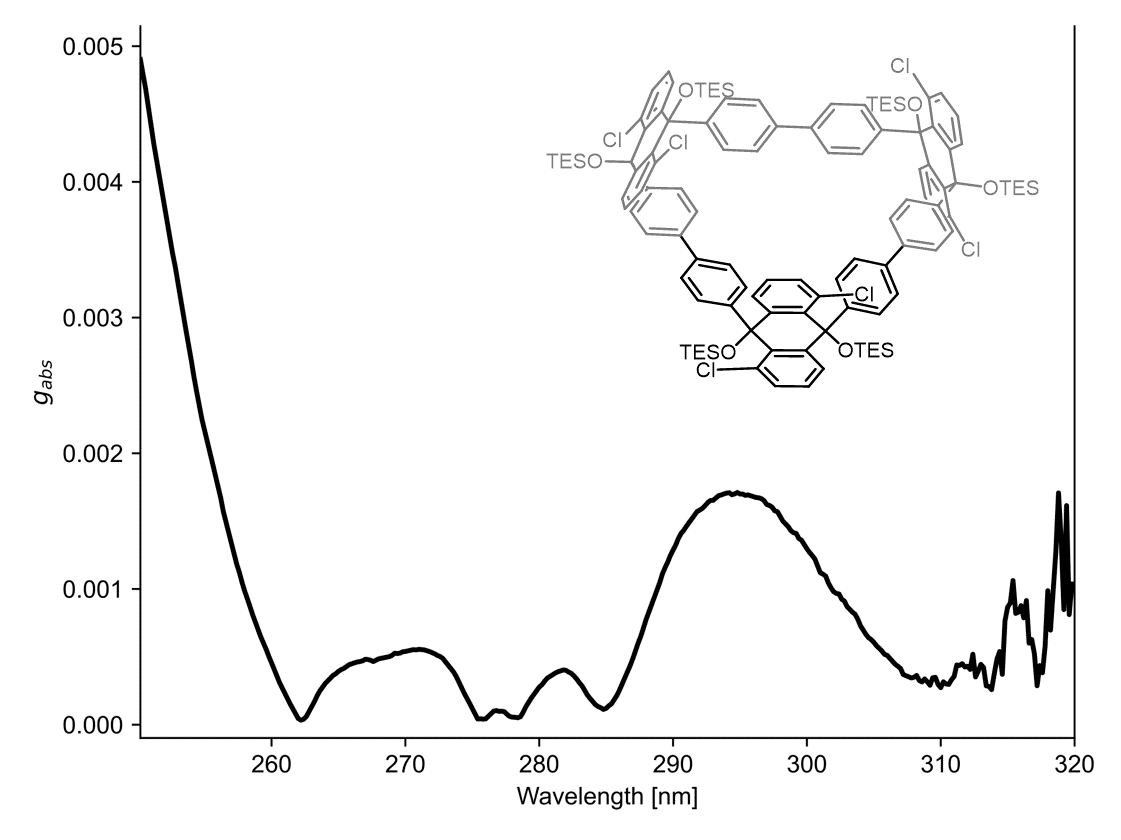


**Figure S87:** g_abs_ profile of **12** in dichloromethane.


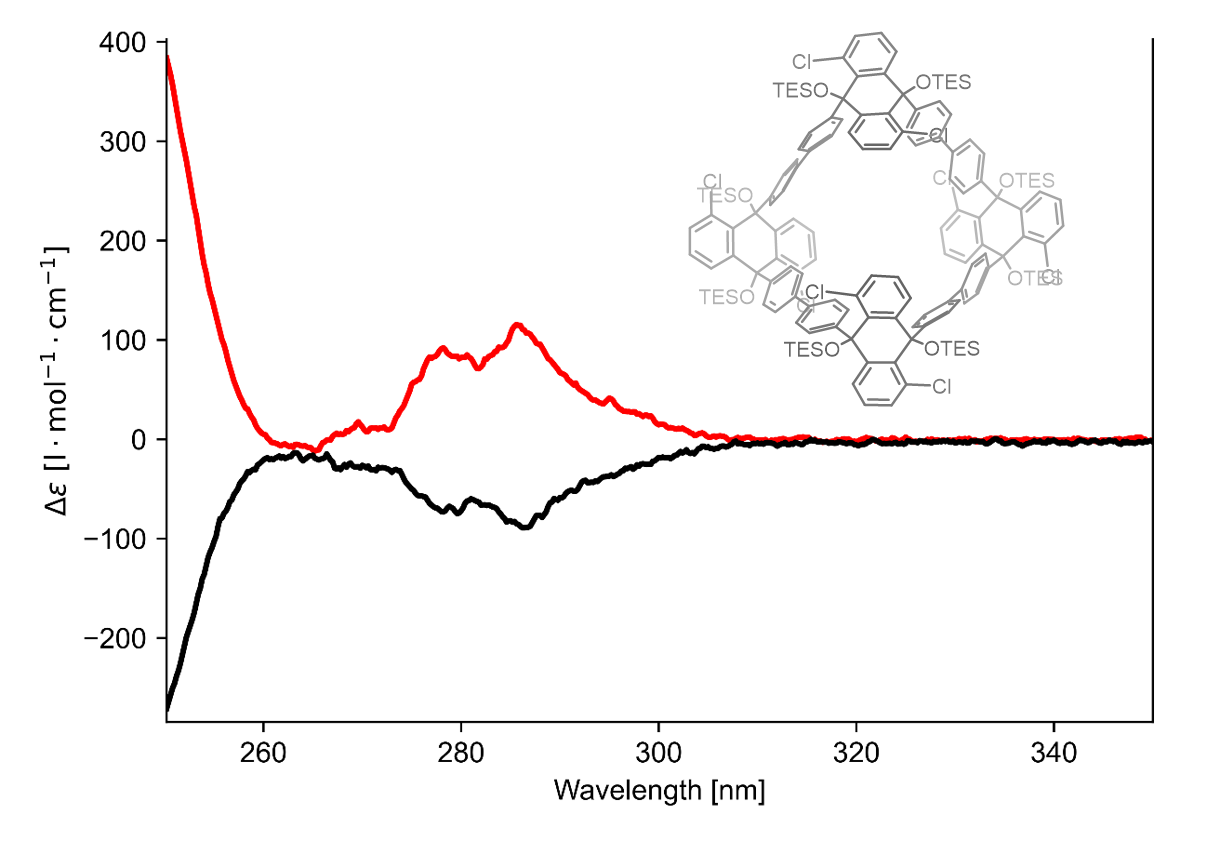


**Figure S88:** CD spectrum of (*all-R*)*-* (red) and (*all-S*)*-***16** (black) in dichloromethane.


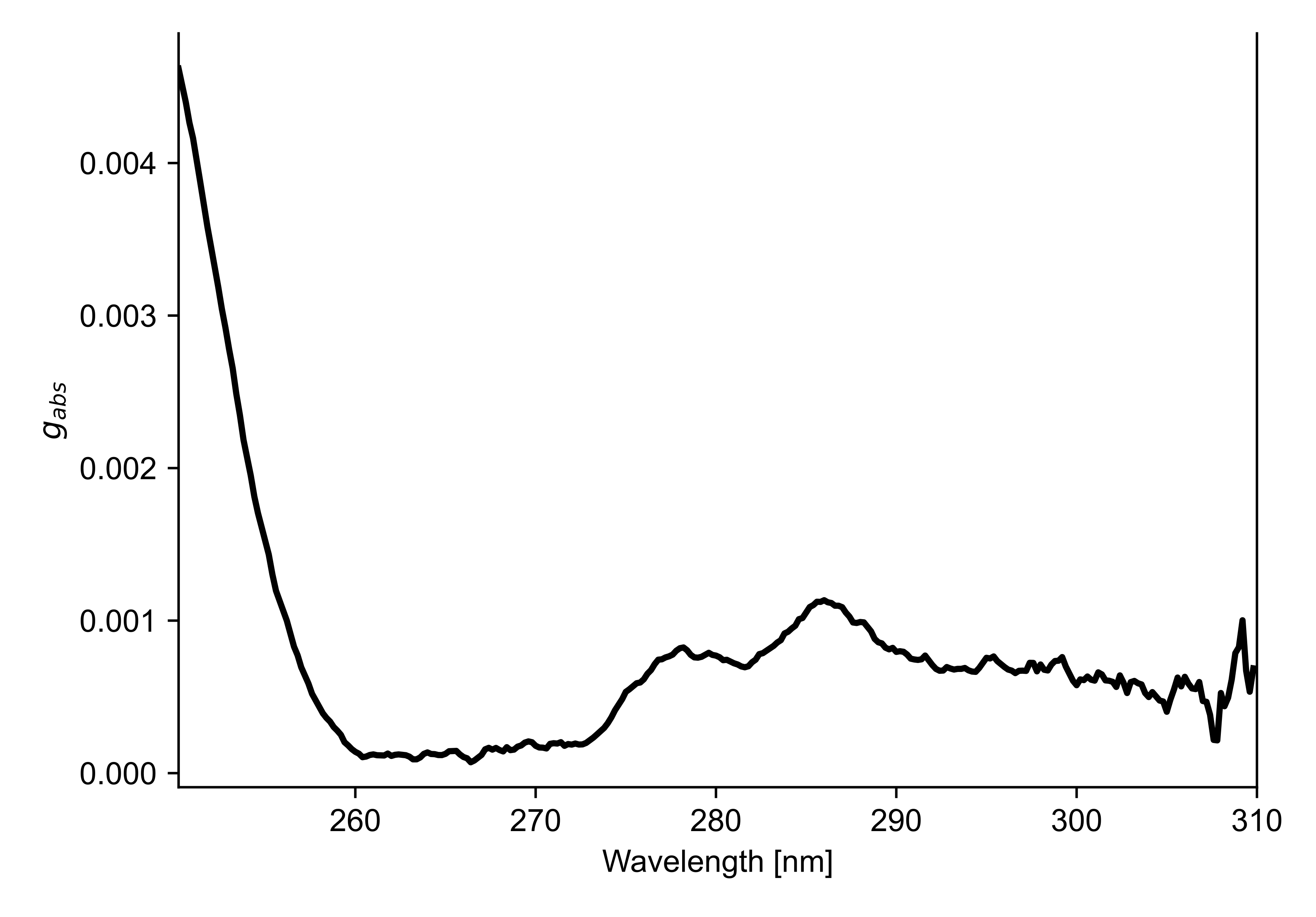


**Figure S89:** g_abs_ profile of **16** in dichloromethane.


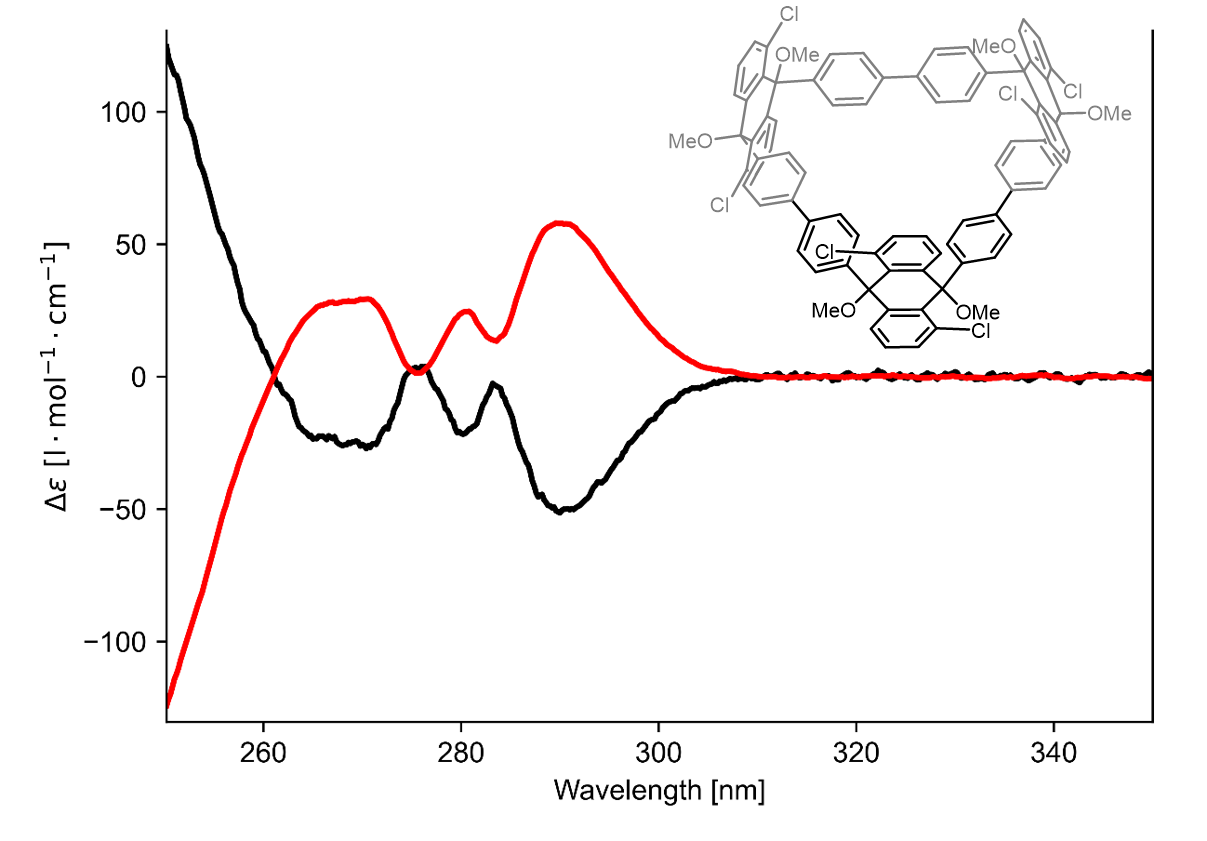


**Figure S90:** CD spectrum of (*all-R*)*-* (red) and (*all-S*)*-***13** (black) in dichloromethane.


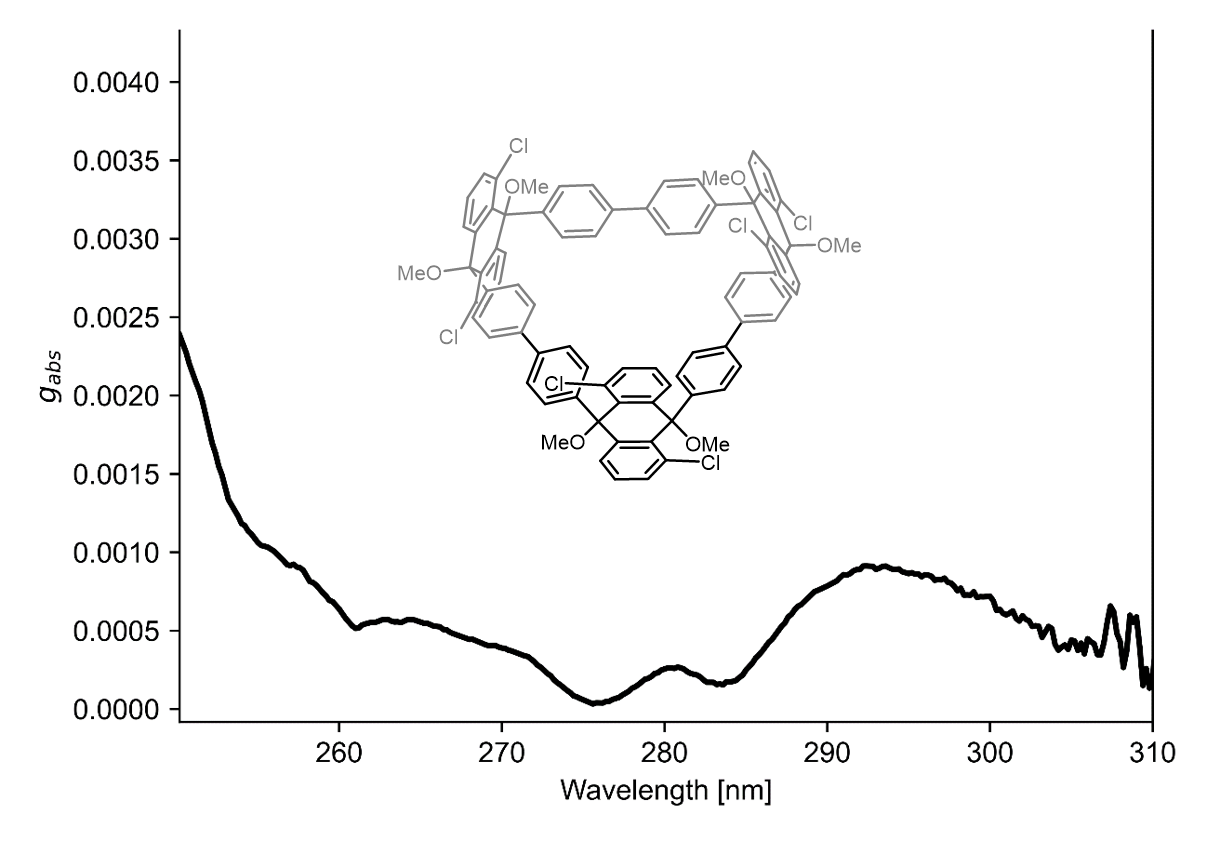


**Figure S91:** g_abs_ profile of **13** in dichloromethane.


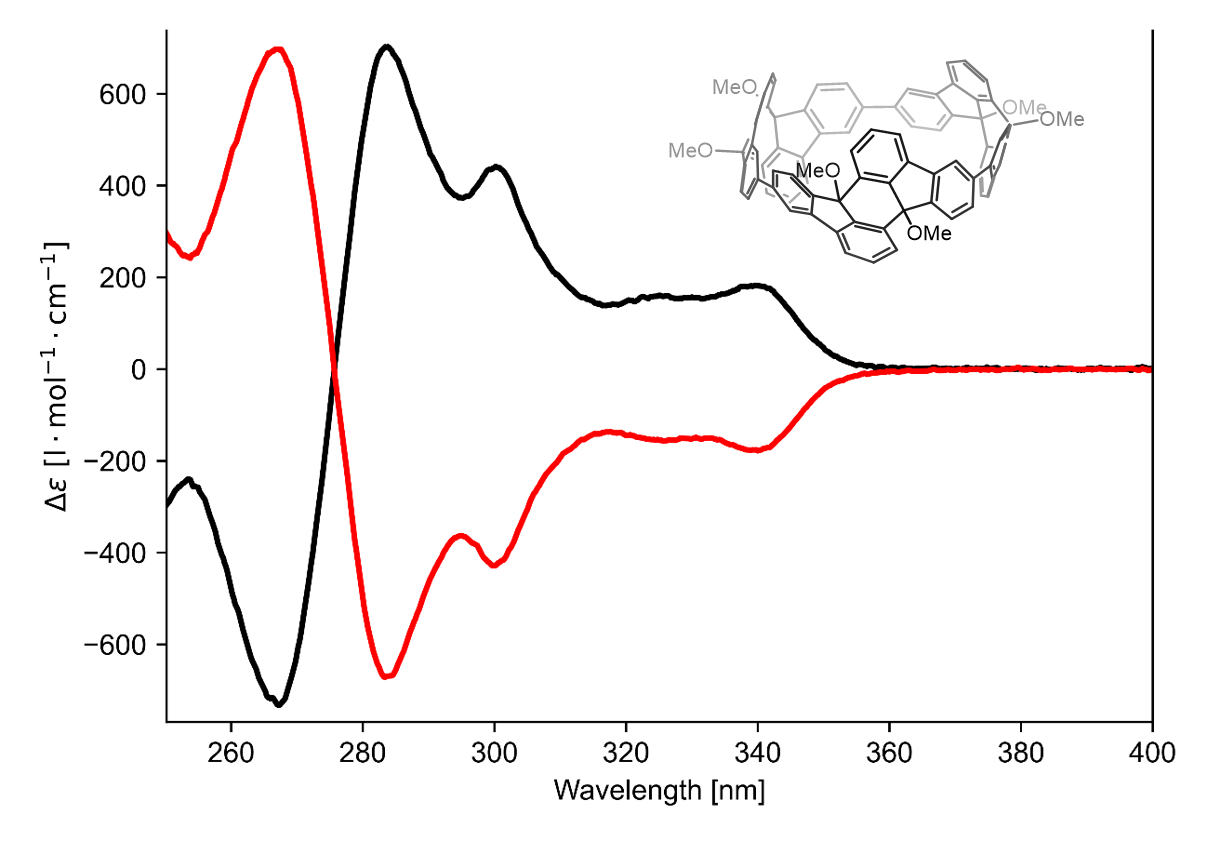


**Figure S92:** CD spectrum of (*all-R*)*-* (red) and (*all-S*)*-***15** (black) in dichloromethane.


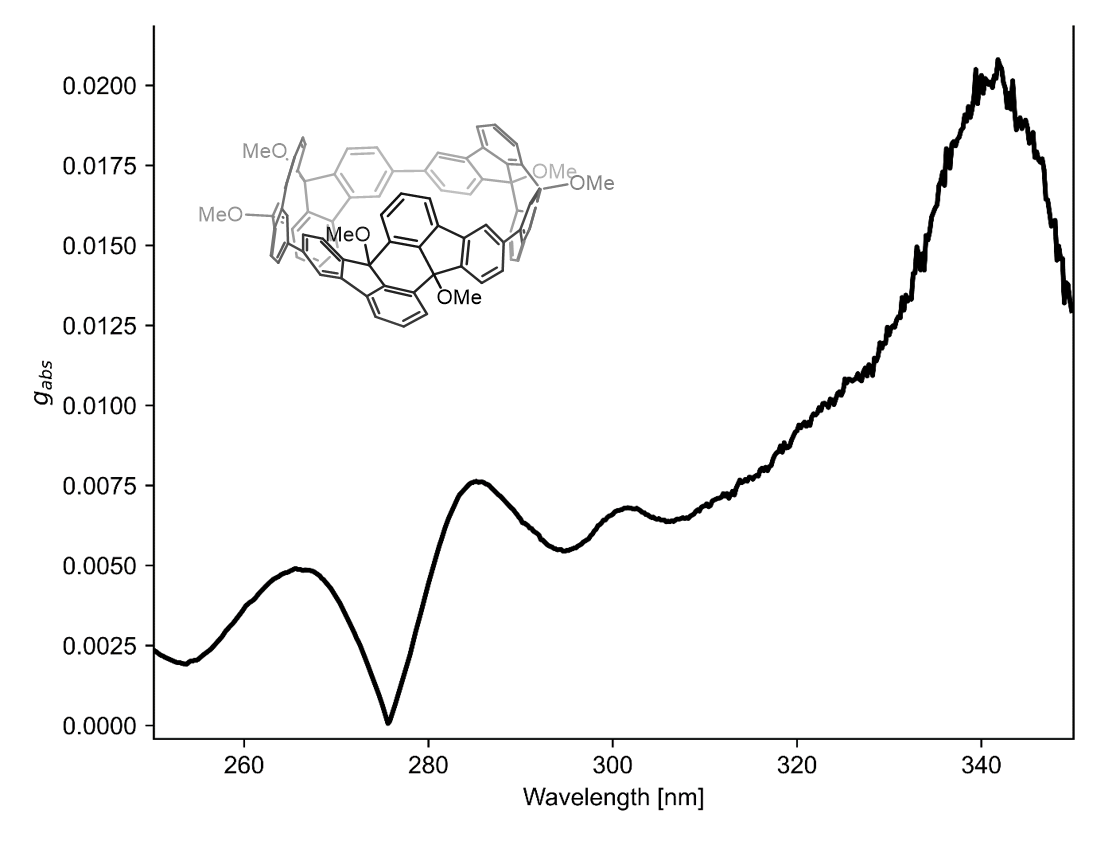


**Figure S93:** g_abs_ profile of **15** in dichloromethane.


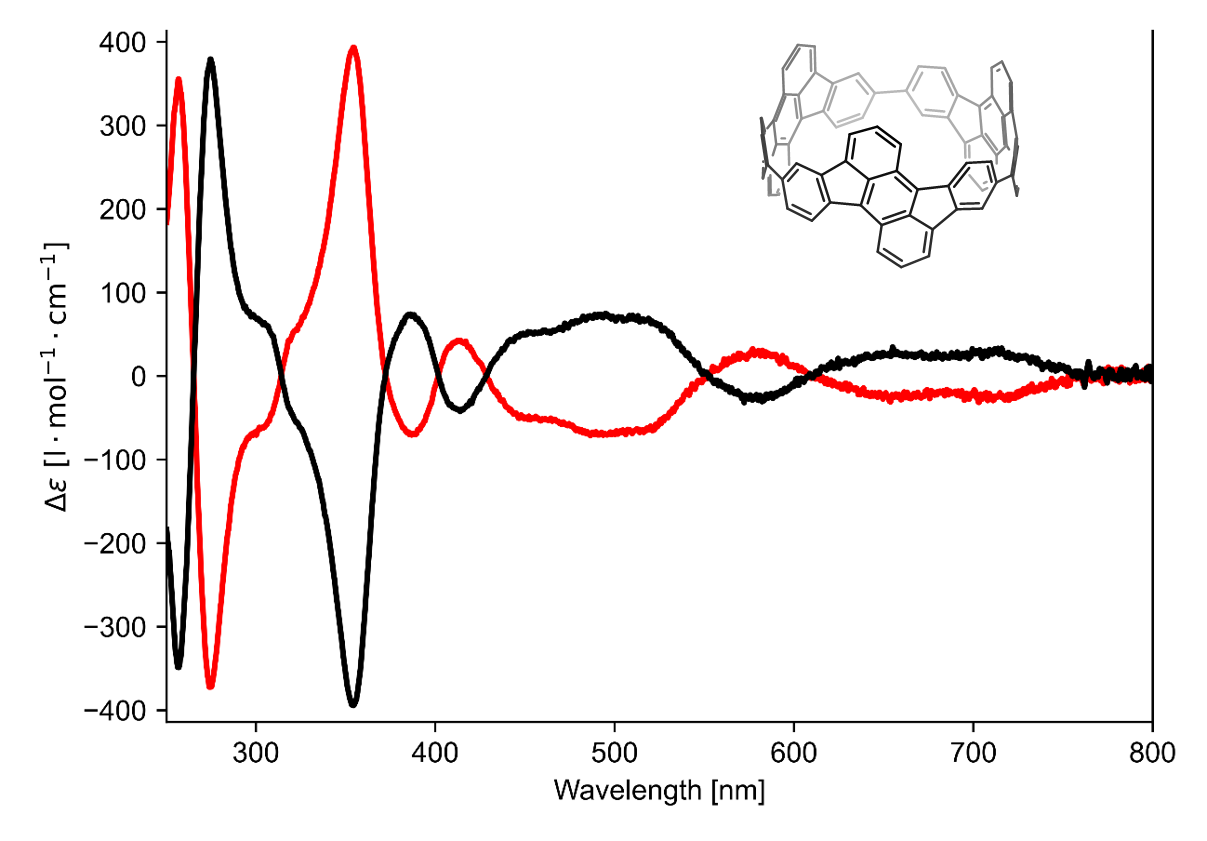


**Figure S94:** CD spectrum of (*all-P*)*-* (red) and (*all-M*)*-***7** (black) in dichloromethane.


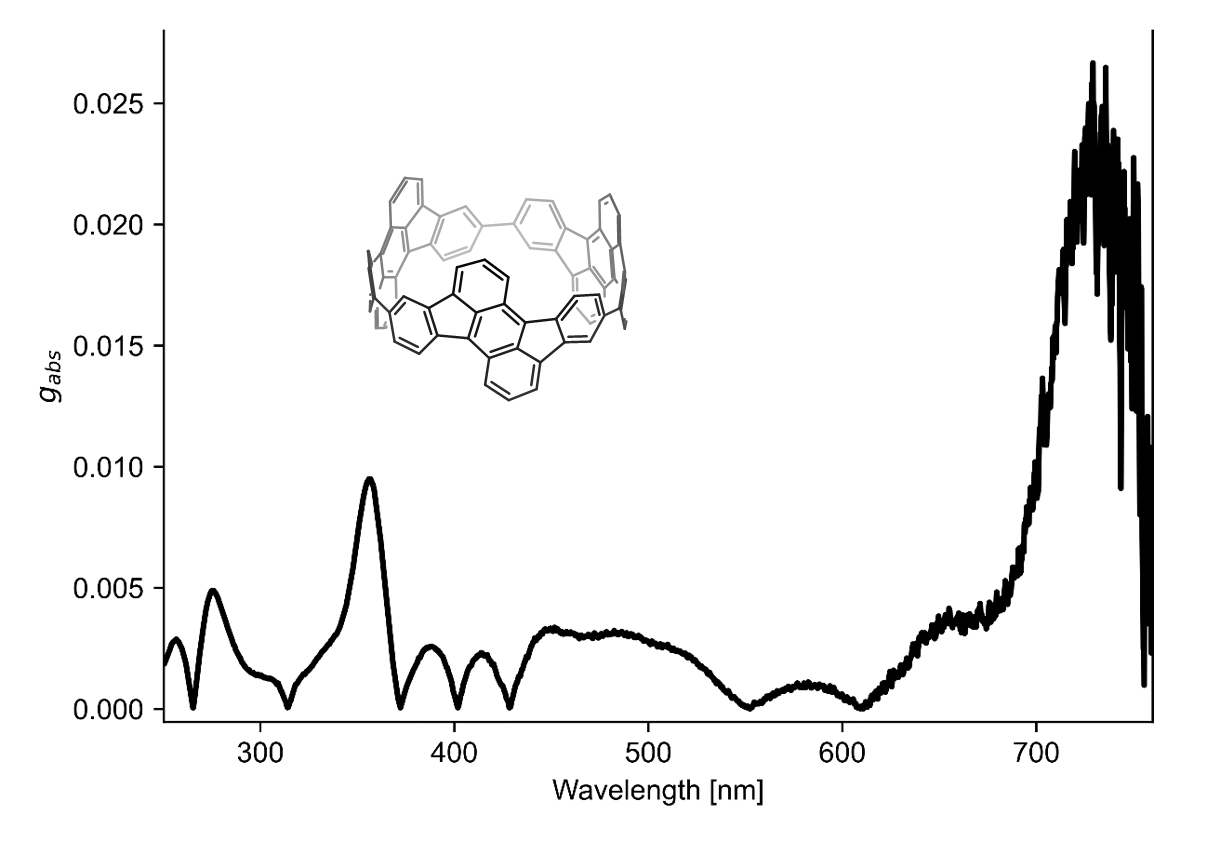


**Figure S95:** g_abs_ profile of **7** in dichloromethane.

# Cyclic voltammograms


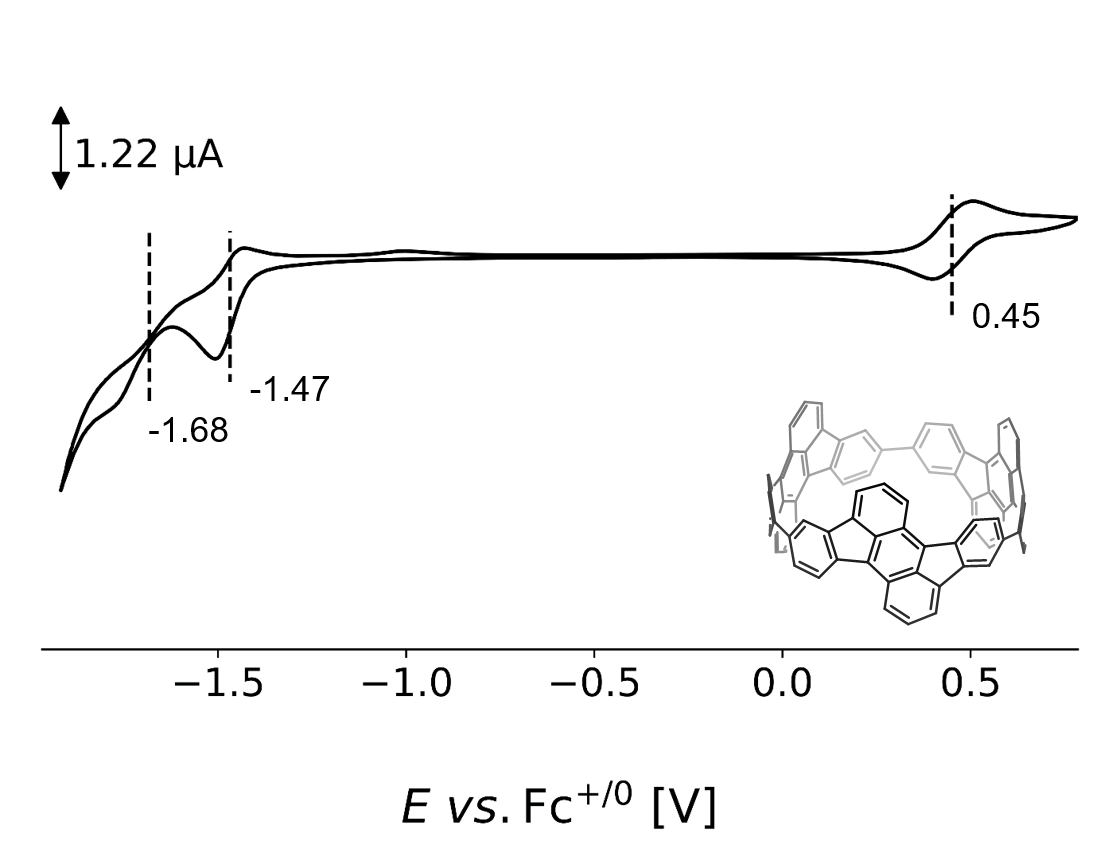


**Figure S96:** Cyclic voltammogram of **7** (dichloromethane, [Bu_4_N][PF_6_] (0.1 M), 100 mVs^–1^).

# Crystallographic data

**Crystallographic data for compound** **10:**

Crystals suitable for X-ray diffraction were obtained by layering a solution of **10** in ethyl acetate with n*-*pentane.

CCDC 2477518

Empirical formula C_26_H_16_Br_2_Cl_2_O_2_

Formula weight 591.11

Temperature 200(2) K

Wavelength 0.71073 Å

Crystal system monoclinic

Space group C2/c

Z 8

Unit cell dimensions a = 22.852(2) Å α = 90 deg.

b = 11.9745(12) Å β = 120.037(2) deg.

c = 18.938(3) Å γ = 90 deg.

Volume 4486.3(10) Å^3^

Density (calculated) 1.75 g/cm^3^

Absorption coefficient 3.88 mm^-1^

Crystal shape brick

Crystal size 0.103 x 0.070 x 0.048 mm^3^

Crystal colour colourless

Theta range for data collection 2.0 to 28.0 deg.

Index ranges -29≤h≤29, -15≤k≤15, -24≤l≤23

Reflections collected 23780

Independent reflections 5069 (R(int) = 0.0705)

Observed reflections 3094 (I > 2σ(I))

Absorption correction Semi-empirical from equivalents

Max. and min. transmission 0.86 and 0.78

Refinement method Full-matrix least-squares on F^2^

Data/restraints/parameters 5069 / 0 / 291

Goodness-of-fit on F^2^ 1.00

Final R indices (I>2sigma(I)) R1 = 0.042, wR2 = 0.069

Largest diff. peak and hole 0.40 and -0.82 eÅ^-3^

**Crystallographic data for compound *rac*-****11:**

Crystals suitable for X-ray diffraction were obtained by diffusion of methanol into a solution of ***rac*-****11** in chloroform.

CCDC 2477519

Empirical formula C_38_H_44_Br_2_Cl_2_O_2_Si_2_

Formula weight 819.63

Temperature 200(2) K

Wavelength 0.71073 Å

Crystal system monoclinic

Space group P2_1_/n

Z 4

Unit cell dimensions a = 10.9498(11) Å α = 90 deg.

b = 15.9920(15) Å β = 96.187(3) deg.

c = 21.661(2) Å γ = 90 deg.

Volume 3770.9(6) Å^3^

Density (calculated) 1.44 g/cm^3^

Absorption coefficient 2.39 mm^-1^

Crystal shape prism

Crystal size 0.224 x 0.141 x 0.117 mm^3^

Crystal colour colourless

Theta range for data collection 1.6 to 25.1 deg.

Index ranges -13≤h≤12, -19≤k≤18, -25≤l≤25

Reflections collected 34735

Independent reflections 6696 (R(int) = 0.0531)

Observed reflections 4794 (I > 2σ(I))

Absorption correction Semi-empirical from equivalents

Max. and min. transmission 0.80 and 0.73

Refinement method Full-matrix least-squares on F^2^

Data/restraints/parameters 6696 / 0 / 421

Goodness-of-fit on F^2^ 1.02

Final R indices (I>2sigma(I)) R1 = 0.042, wR2 = 0.087

Largest diff. peak and hole 0.67 and -0.58 eÅ^-3^

**Crystallographic data for compound (*S,S*)-****11:**

Crystals suitable for X-ray diffraction were obtained by layering a solution of (*S,S*)*-***11** in chloroform with methanol.

CCDC 2477520

Empirical formula C_38_H_44_Br_2_Cl_2_O_2_Si_2_

Formula weight 819.63

Temperature 200(2) K

Wavelength 1.54178 Å

Crystal system monoclinic

Space group C2

Z 8

Unit cell dimensions a = 42.1139(17) Å α = 90 deg.

b = 16.1134(4) Å β = 95.663(3) deg.

c = 11.2544(4) Å γ = 90 deg.

Volume 7599.9(5) Å^3^

Density (calculated) 1.43 g/cm^3^

Absorption coefficient 4.85 mm^-1^

Crystal shape prism

Crystal size 0.226 x 0.093 x 0.060 mm^3^

Crystal colour colourless

Theta range for data collection 2.1 to 68.7 deg.

Index ranges -50≤h≤39, -17≤k≤18, -9≤l≤13

Reflections collected 32172

Independent reflections 11578 (R(int) = 0.0460)

Observed reflections 7653 (I > 2σ(I))

Absorption correction Semi-empirical from equivalents

Max. and min. transmission 0.65 and 0.41

Refinement method Full-matrix least-squares on F^2^

Data/restraints/parameters 11578 / 1494 / 862

Goodness-of-fit on F^2^ 0.86

Final R indices (I>2sigma(I)) R1 = 0.038, wR2 = 0.065

Absolute structure parameter 0.124(16)

Largest diff. peak and hole 0.39 and -0.36 eÅ^-3^

**Crystallographic data for compound (*all-S*)*-*****12:**

Crystals suitable for X-ray diffraction were obtained by layering a solution of (*all-S*)*-***12** in dichloromethane with methanol.

CCDC 2477521

Empirical formula C_115.50_H_135_Cl_9_O_6_Si_6_

Formula weight 2106.82

Temperature 200(2) K

Wavelength 1.54178 Å

Crystal system orthorhombic

Space group P2_1_2_1_2_1_

Z 8

Unit cell dimensions a = 14.6347(3) Å α = 90 deg.

b = 39.2936(8) Å β = 90 deg.

c = 40.4280(10) Å γ = 90 deg.

Volume 23248.1(9) Å^3^

Density (calculated) 1.20 g/cm^3^

Absorption coefficient 2.97 mm^-1^

Crystal shape plate

Crystal size 0.160 x 0.070 x 0.020 mm^3^

Crystal colour colourless

Theta range for data collection 2.2 to 62.3 deg.

Index ranges -9≤h≤16, -43≤k≤44, -44≤l≤45

Reflections collected 115815

Independent reflections 34332 (R(int) = 0.1368)

Observed reflections 20985 (I > 2σ(I))

Absorption correction Semi-empirical from equivalents

Max. and min. transmission 0.97 and 0.74

Refinement method Full-matrix least-squares on F^2^

Data/restraints/parameters 34332 / 9696 / 2458

Goodness-of-fit on F^2^ 1.07

Final R indices (I>2sigma(I)) R1 = 0.095, wR2 = 0.213

Absolute structure parameter 0.266(9)

Largest diff. peak and hole 0.54 and -0.54 eÅ^-3^

**Crystallographic data for compound (*all-S*)*-*14:**

Crystals suitable for X-ray diffraction were obtained by layering a solution of (*all-S*)*-***14** in chloroform with methanol.

Identification code 2477522

Empirical formula C_158_H_182_Cl_26_O_8_Si_8_

Formula weight 3355.45

Temperature 200(2) K

Wavelength 1.54178 Å

Crystal system orthorhombic

Space group P2_1_2_1_2

Z 2

Unit cell dimensions a = 17.1502(2) Å α = 90 deg.

b = 34.2589(4) Å β = 90 deg.

c = 14.5653(2) Å γ = 90 deg.

Volume 8557.80(18) Å^3^

Density (calculated) 1.30 g/cm^3^

Absorption coefficient 4.74 mm^-1^

Crystal shape column

Crystal size 0.232 x 0.135 x 0.100 mm^3^

Crystal colour colourless

Theta range for data collection 2.6 to 68.6 deg.

Index ranges -19≤h≤20, -37≤k≤41, -17≤l≤11

Reflections collected 65440

Independent reflections 15445 (R(int) = 0.0367)

Observed reflections 11601 (I > 2σ(I))

Absorption correction Semi-empirical from equivalents

Max. and min. transmission 0.69 and 0.54

Refinement method Full-matrix least-squares on F^2^

Data/restraints/parameters 15445 / 1355 / 938

Goodness-of-fit on F^2^ 1.01

Final R indices (I>2sigma(I)) R1 = 0.071, wR2 = 0.202

Absolute structure parameter -0.001(5)

Largest diff. peak and hole 0.62 and -0.51 eÅ^-3^

**Crystallographic data for compound (*all-S*)*-*13:**

Crystals suitable for X-ray diffraction were obtained slow evaporation of a solution of (*all-S*)*-***13** in CDCl_3_.

CCDC 2477523

Empirical formula C_87_H_63_Cl_15_O_6_

Formula weight 1736.12

Temperature 200(2) K

Wavelength 1.54178 Å

Crystal system orthorhombic

Space group P2_1_2_1_2_1_

Z 8

Unit cell dimensions a = 14.7856(3) Å α = 90 deg.

b = 21.0667(6) Å β = 90 deg.

c = 55.2321(11) Å γ = 90 deg.

Volume 17203.9(7) Å^3^

Density (calculated) 1.34 g/cm^3^

Absorption coefficient 4.80 mm^-1^

Crystal shape plate

Crystal size 0.114 x 0.071 x 0.015 mm^3^

Crystal colour colourless

Theta range for data collection 2.2 to 65.1 deg.

Index ranges -12≤h≤17, -22≤k≤24, -64≤l≤46

Reflections collected 112041

Independent reflections 28519 (R(int) = 0.1674)

Observed reflections 15402 (I > 2σ(I))

Absorption correction Semi-empirical from equivalents

Max. and min. transmission 0.97 and 0.69

Refinement method Full-matrix least-squares on F^2^

Data/restraints/parameters 28519 / 4059 / 1995

Goodness-of-fit on F^2^ 1.59

Final R indices (I>2sigma(I)) R1 = 0.136, wR2 = 0.315

Absolute structure parameter 0.22(3)

Largest diff. peak and hole 1.07 and -0.57 eÅ^-3^

**Crystallographic data for compound (*all-S*)*-*15:**

Crystals suitable for X-ray diffraction were obtained by layering a solution of (*all-S*)*-***15** in chloroform with methanol.

Identification code 2477524

Empirical formula C_91_H_61_Cl_21_O_6_

Formula weight 1994.84

Temperature 200(2) K

Wavelength 1.54178 Å

Crystal system orthorhombic

Space group P2_1_2_1_2_1_

Z 4

Unit cell dimensions a = 15.0209(3) Å α = 90 deg.

b = 22.1286(5) Å β = 90 deg.

c = 27.8509(8) Å γ = 90 deg.

Volume 9257.4(4) Å^3^

Density (calculated) 1.43 g/cm^3^

Absorption coefficient 6.10 mm^-1^

Crystal shape column

Crystal size 0.282 x 0.068 x 0.052 mm^3^

Crystal colour colourless

Theta range for data collection 3.2 to 61.1 deg.

Index ranges -16≤h≤9, -25≤k≤24, -30≤l≤31

Reflections collected 56861

Independent reflections 13663 (R(int) = 0.0639)

Observed reflections 9945 (I > 2σ(I))

Absorption correction Semi-empirical from equivalents

Max. and min. transmission 0.98 and 0.85

Refinement method Full-matrix least-squares on F^2^

Data/restraints/parameters 13663 / 1587 / 1106

Goodness-of-fit on F^2^ 1.37

Final R indices (I>2sigma(I)) R1 = 0.116, wR2 = 0.312

Absolute structure parameter 0.028(10)

Largest diff. peak and hole 1.06 and -0.74 eÅ^-3^

**Crystallographic data for compound (*all-M*)*-*****7:**

Crystals suitable for X-ray diffraction were obtained by layering a solution of (*all-M*)*-***7** in CHCl_3_ with ethanol.

CCDC 2477525

Empirical formula C_78_H_36_

Formula weight 973.07

Temperature 200(2) K

Wavelength 1.54178 Å

Crystal system orthorhombic

Space group I222

Z 8

Unit cell dimensions a = 22.6862(5) Å α = 90 deg.

b = 24.4646(4) Å β = 90 deg.

c = 25.4645(5) Å γ = 90 deg.

Volume 14133.0(5) Å^3^

Density (calculated) 0.92 g/cm^3^

Absorption coefficient 0.40 mm^-1^

Crystal shape plank

Crystal size 0.145 x 0.076 x 0.028 mm^3^

Crystal colour dark violet

Theta range for data collection 2.5 to 56.0 deg.

Index ranges -22≤h≤24, -26≤k≤20, -27≤l≤27

Reflections collected 48778

Independent reflections 9198 (R(int) = 0.0550)

Observed reflections 5979 (I > 2σ(I))

Absorption correction Semi-empirical from equivalents

Max. and min. transmission 0.99 and 0.52

Refinement method Full-matrix least-squares on F^2^

Data/restraints/parameters 9198 / 783 / 703

Goodness-of-fit on F^2^ 1.05

Final R indices (I>2sigma(I)) R1 = 0.072, wR2 = 0.213

Absolute structure parameter 7.7(4)

Largest diff. peak and hole 0.17 and -0.14 eÅ^-3^

# GPC and HPLC Chromatograms


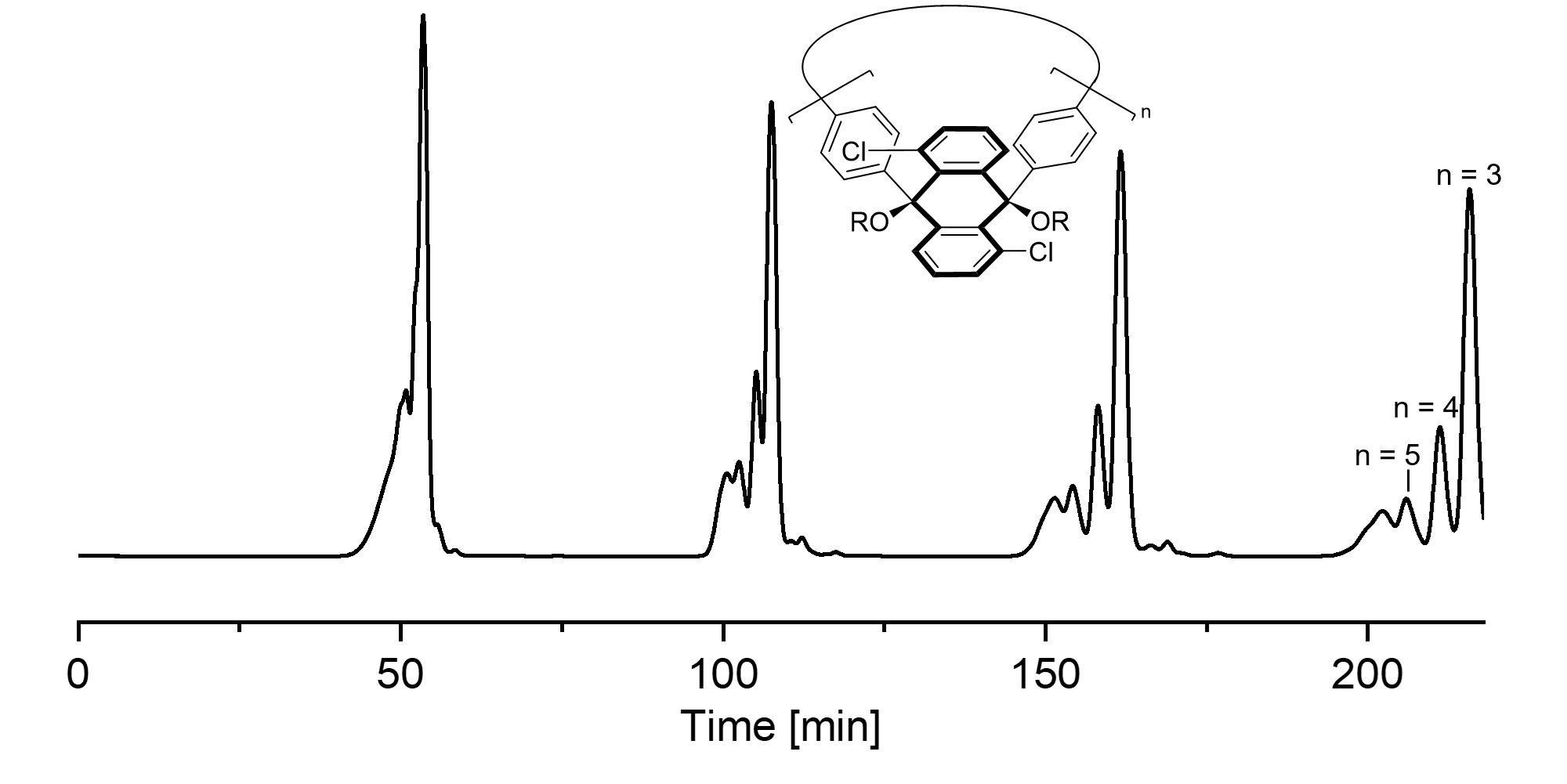


**Figure S97:** GPC chromatogram of the macrocyclization (4 × SDV 100 Å and 1 × SDV 500 Å, 5 mL/min, dichloromethane). While n = 3 and 4, could be isolated, n=5 was formed in too small amounts for isolation and full characterization and therefore identified only by MALDI-TOF MS.


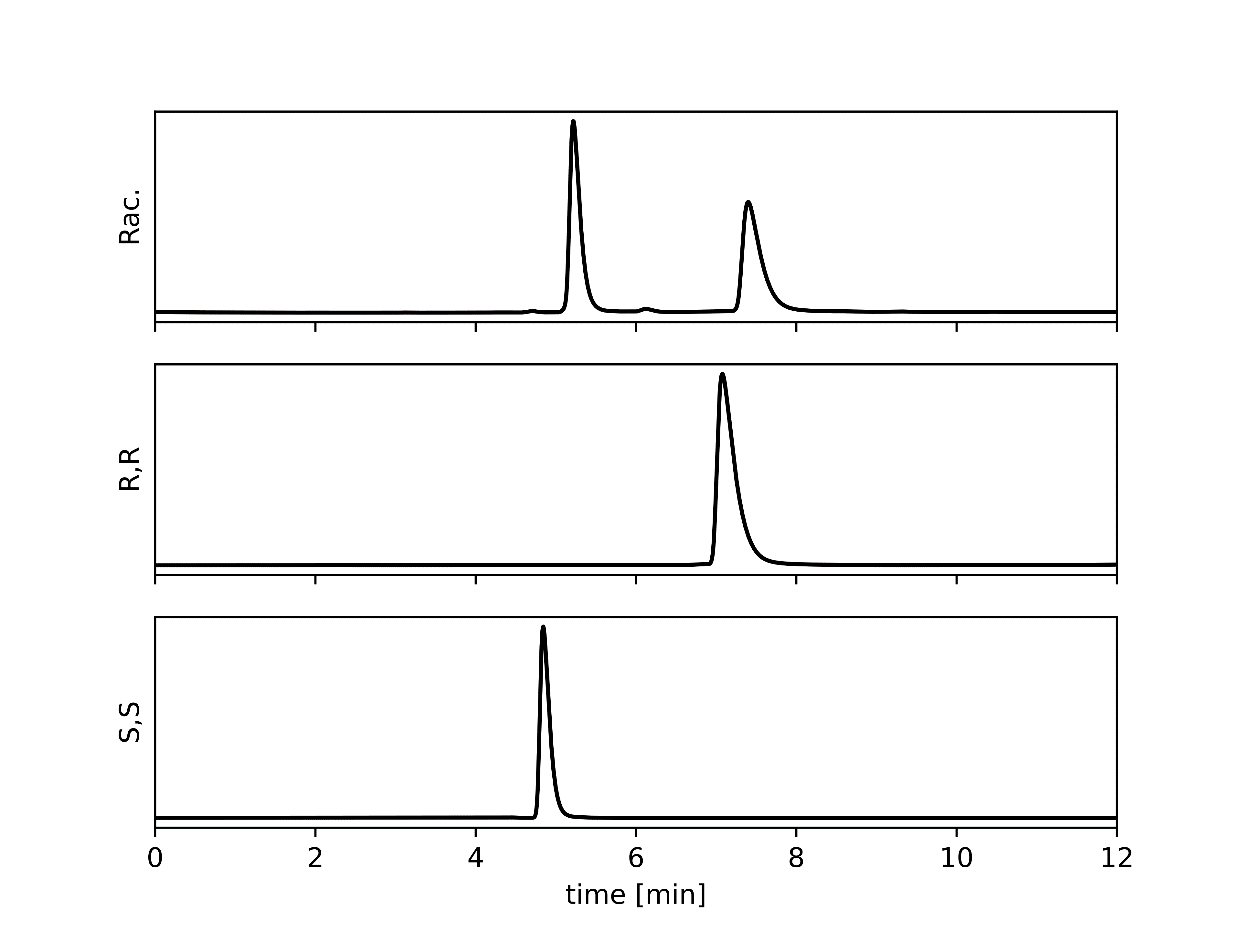


**Figure S98:** analytical HPLC chromatogram of **11** (Chiralpak IB, 1 mL/min, *n*-heptane).


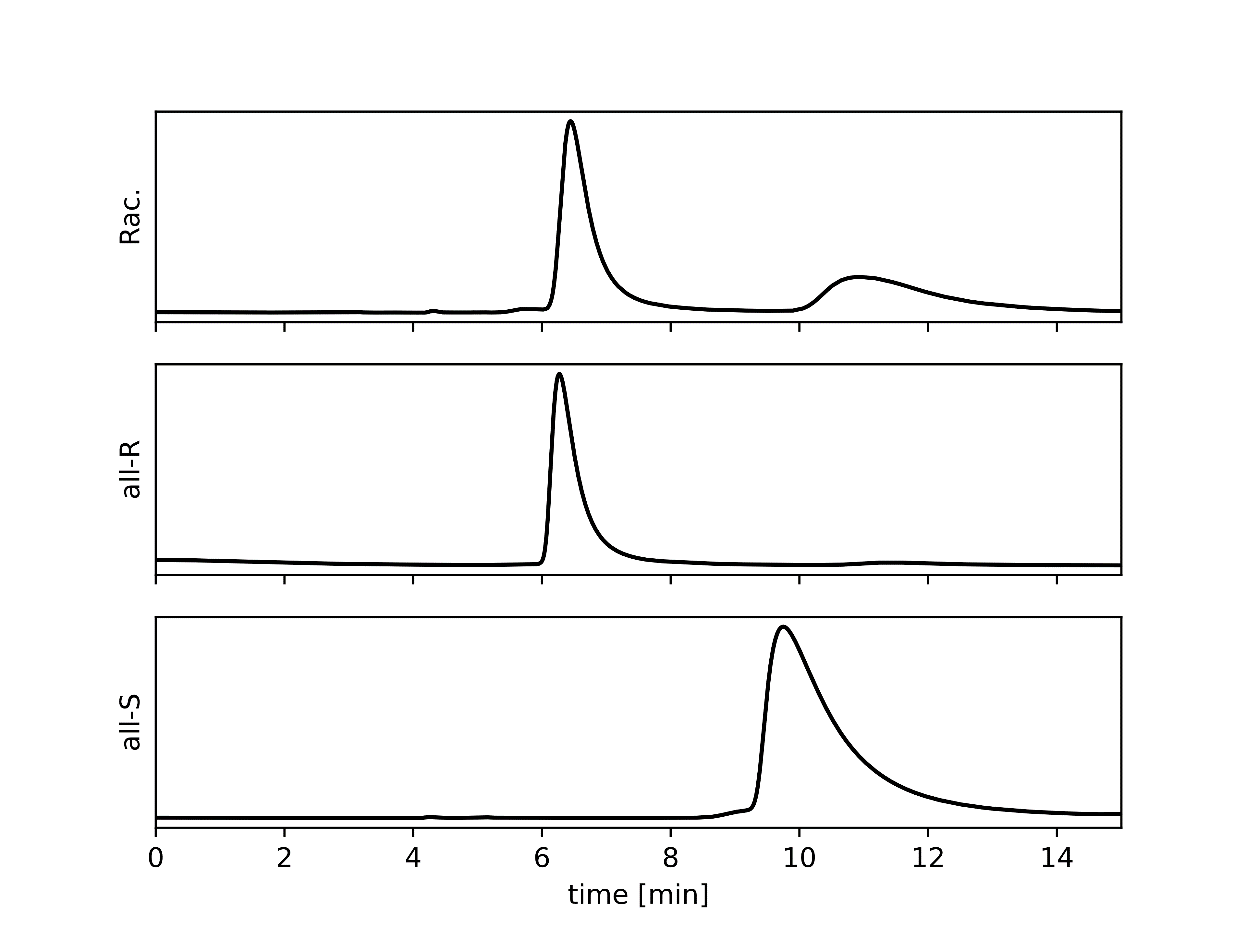


**Figure S99:** analytical HPLC chromatogram of **112** (Chiralpak IB, 1 mL/min, *n*-heptane).


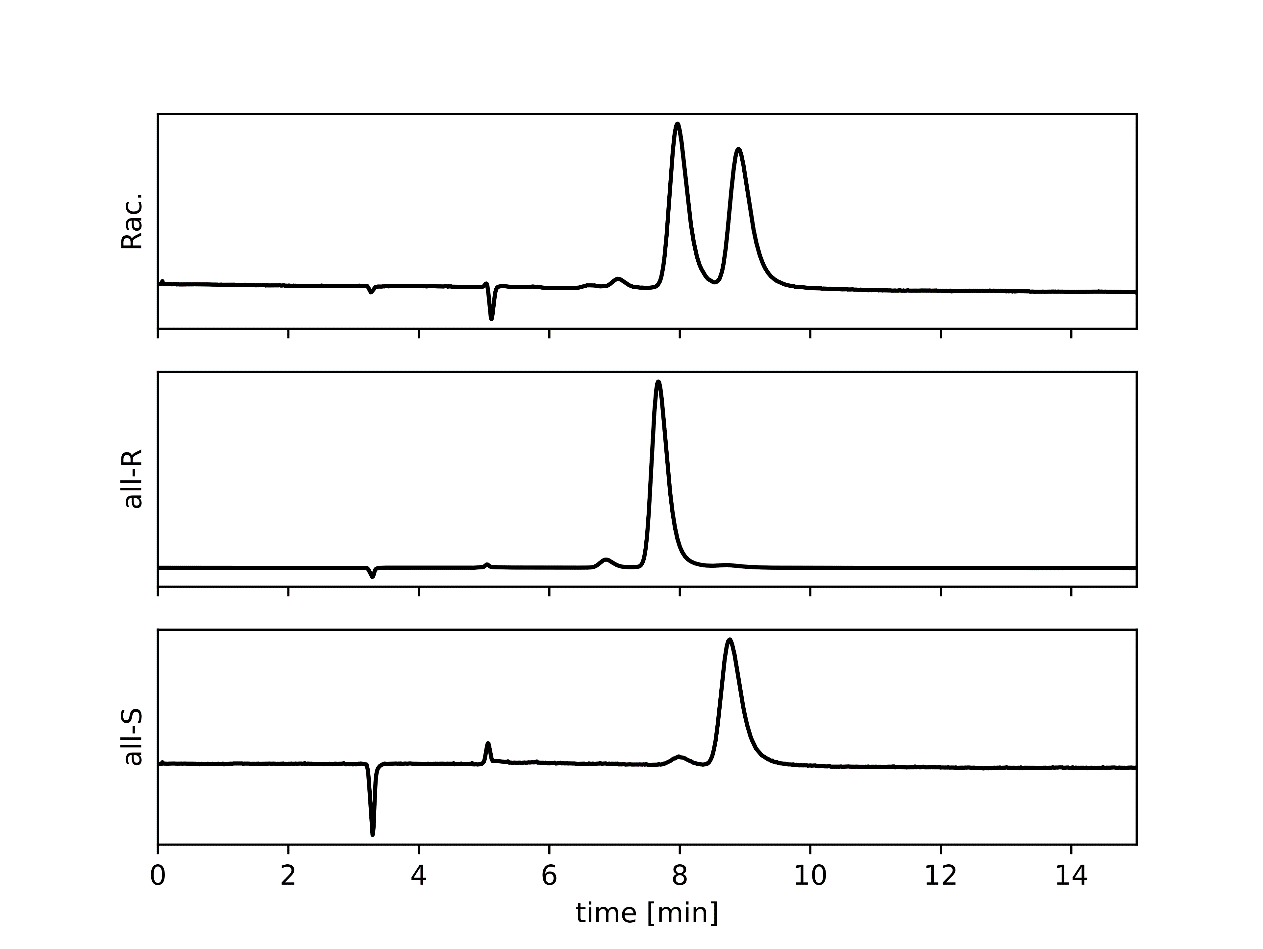


**Figure S100:** analytical HPLC chromatogram of **13** (Chiralpak IB, 1 mL/min, 20% dichloromethane/*n*-heptane).

**Figure S101:** analytical HPLC chromatogram of **15** (Chiralpak IB, 1 mL/min, dichloromethane).

**Figure S102:** analytical HPLC chromatogram of **7** (Chiralpak IB, 1 mL/min, 45% dichloromethane/n-heptane).

# Computational details

## XYZ-Coordinates

**XYZ coordinates of** **5:**

**Element X Y Z**

C -3.59725 0.87221 -5.06677

C -2.86623 -0.29130 -5.13235

C -1.43940 -0.25365 -5.16980

C -0.84835 1.03915 -5.15847

C -1.61591 2.23936 -5.06982

C -2.98348 2.15765 -5.02501

C -0.65341 3.35042 -5.01348

C 0.65873 2.79858 -5.10119

C 1.75115 3.65760 -5.06440

C 1.54074 5.03187 -4.90497

C 0.25774 5.57636 -4.76265

C -0.84613 4.71063 -4.83328

C -0.53337 -1.33411 -5.17170

C 0.84835 -1.03915 -5.15847

C 1.43940 0.25365 -5.16980

C 0.53337 1.33411 -5.17170

C 1.61591 -2.23936 -5.06982

C 2.98348 -2.15765 -5.02501

C 3.59725 -0.87221 -5.06677

C 2.86623 0.29130 -5.13235

C 0.65341 -3.35042 -5.01348

C -0.65873 -2.79858 -5.10119

C -1.75115 -3.65760 -5.06440

C -1.54074 -5.03187 -4.90497

C -0.25774 -5.57636 -4.76265

C 0.84613 -4.71063 -4.83328

C -3.64760 -4.09392 1.34414

C -2.95929 -3.72539 2.47656

C -1.58497 -4.07666 2.64009

C -1.00340 -4.83416 1.58741

C -1.72193 -5.19287 0.40798

C -3.03687 -4.82621 0.28535

C -0.76956 -5.89155 -0.46677

C 0.48406 -5.95806 0.21026

C 1.56325 -6.54622 -0.43962

C 1.40064 -7.03678 -1.74138

C 0.18126 -6.94443 -2.42617

C -0.91144 -6.36453 -1.75933

C -0.71264 -3.73613 3.69348

C 0.62502 -4.18381 3.61813

C 1.20169 -4.95763 2.57426

C 0.33482 -5.28192 1.50974

C 1.37864 -3.69701 4.72914

C 2.70881 -4.01490 4.82432

C 3.29879 -4.81917 3.80617

C 2.58678 -5.27228 2.71960

C 0.45920 -2.87536 5.53305

C -0.82110 -2.91348 4.90762

C -1.86149 -2.17375 5.45823

C -1.61974 -1.38036 6.58324

C -0.35687 -1.30772 7.18453

C 0.68349 -2.08588 6.65106

C -3.29879 4.81917 3.80617

C -2.58678 5.27228 2.71960

C -1.20169 4.95763 2.57426

C -0.62502 4.18381 3.61813

C -1.37864 3.69701 4.72914

C -2.70881 4.01490 4.82432

C -0.45920 2.87536 5.53305

C 0.82110 2.91348 4.90762

C 1.86149 2.17375 5.45823

C 1.61974 1.38036 6.58324

C 0.35687 1.30772 7.18453

C -0.68349 2.08588 6.65106

C -0.33482 5.28192 1.50974

C 1.00340 4.83416 1.58741

C 1.58497 4.07666 2.64009

C 0.71264 3.73613 3.69348

C 1.72193 5.19287 0.40798

C 3.03687 4.82621 0.28535

C 3.64760 4.09392 1.34414

C 2.95929 3.72539 2.47656

C 0.76956 5.89155 -0.46677

C -0.48406 5.95806 0.21026

C -1.56325 6.54622 -0.43962

C -1.40064 7.03678 -1.74138

C -0.18126 6.94443 -2.42617

C 0.91144 6.36453 -1.75933

H -4.68043 0.81287 -5.03131

H -3.38120 -1.24345 -5.14354

H -3.60357 3.04515 -4.94746

H 2.76565 3.28273 -5.14001

H 2.40702 5.68487 -4.85131

H -1.84982 5.10303 -4.70295

H 3.60357 -3.04515 -4.94746

H 4.68043 -0.81287 -5.03131

H 3.38120 1.24345 -5.14354

H -2.76565 -3.28273 -5.14001

H -2.40702 -5.68487 -4.85131

H 1.84982 -5.10303 -4.70295

H -4.68930 -3.80636 1.24349

H -3.46619 -3.15717 3.24580

H -3.61410 -5.07036 -0.60078

H 2.53695 -6.61370 0.03268

H 2.26493 -7.46354 -2.24149

H -1.85850 -6.24742 -2.27465

H 3.32041 -3.66160 5.64872

H 4.35152 -5.07056 3.88838

H 3.08601 -5.86942 1.96697

H -2.84896 -2.17057 5.01128

H -2.42938 -0.76844 6.96848

H 1.67875 -2.03070 7.08243

H -4.35152 5.07056 3.88838

H -3.08601 5.86942 1.96697

H -3.32041 3.66160 5.64872

H 2.84896 2.17057 5.01128

H 2.42938 0.76844 6.96848

H -1.67875 2.03070 7.08243

H 3.61410 5.07036 -0.60078

H 4.68930 3.80636 1.24349

H 3.46619 3.15717 3.24580

H -2.53695 6.61370 0.03268

H -2.26493 7.46354 -2.24149

H 1.85850 6.24742 -2.27465

C 0.87644 -9.72270 -4.08619

C -0.46569 -9.74688 -3.79403

H 1.55255 -9.56745 -3.25191

H -0.74420 -9.63487 -2.75094

C 0.52819 -8.08215 -6.54594

C -0.80921 -8.36917 -6.40998

H 0.78338 -7.10333 -6.93999

H -1.50559 -7.58085 -6.67718

C 0.80921 8.36917 -6.40998

C -0.52819 8.08215 -6.54594

H 1.50559 7.58085 -6.67718

H -0.78338 7.10333 -6.93999

C 0.46569 9.74688 -3.79403

C -0.87644 9.72270 -4.08619

H 0.74420 9.63487 -2.75094

H -1.55255 9.56745 -3.25191

C 1.12888 1.24852 10.28733

C -0.19645 1.57690 10.43931

H 1.72513 1.89394 9.64969

H -0.55232 2.43921 9.88477

C 0.19645 -1.57690 10.43931

C -1.12888 -1.24852 10.28733

H 0.55232 -2.43921 9.88477

H -1.72513 -1.89394 9.64969

Pt 0.00000 -7.55114 -4.35952

Pt -0.00000 -0.00000 8.69985

Pt 0.00000 7.55114 -4.35952

C 1.40403 9.74978 -6.20132

H 0.79651 10.49442 -6.71655

H 2.38253 9.77995 -6.68716

C 1.59346 10.14172 -4.71800

H 1.77148 11.22296 -4.64487

H 2.50136 9.66325 -4.34069

C -1.68401 9.04822 -6.43492

H -2.56976 8.46186 -6.17527

H -1.89476 9.49095 -7.41761

C -1.50978 10.17381 -5.38960

H -2.49767 10.58517 -5.16758

H -0.93576 10.99911 -5.81205

C 1.91375 0.23057 11.08084

H 2.34886 0.70673 11.96963

H 2.76110 -0.07635 10.46147

C 1.12731 -1.03102 11.50633

H 0.56524 -0.83744 12.42046

H 1.85013 -1.80928 11.76358

C -1.12731 1.03102 11.50633

H -1.85013 1.80928 11.76358

H -0.56524 0.83744 12.42046

C -1.91375 -0.23057 11.08084

H -2.76110 0.07635 10.46147

H -2.34886 -0.70673 11.96963

C 1.50978 -10.17381 -5.38960

H 0.93576 -10.99911 -5.81205

H 2.49767 -10.58517 -5.16758

C 1.68401 -9.04822 -6.43492

H 1.89476 -9.49095 -7.41761

H 2.56976 -8.46186 -6.17527

C -1.59346 -10.14172 -4.71800

H -1.77148 -11.22296 -4.64487

H -2.50136 -9.66325 -4.34069

C -1.40403 -9.74978 -6.20132

H -2.38253 -9.77995 -6.68716

H -0.79651 -10.49442 -6.71655

**XYZ coordinates of** **6:**

**Element X Y Z**

C -4.61306 6.69476 1.61796

C -5.02082 6.92808 2.90591

C -6.13408 6.20023 3.41769

C -6.81395 5.26995 2.66610

C -6.42288 4.99418 1.32079

C -5.31250 5.73211 0.82929

C -6.99514 4.08308 0.40932

C -6.43176 3.99565 -0.88304

C -5.31805 4.73090 -1.37314

C -4.74047 5.63625 -0.45926

C -7.13413 3.03515 -1.67194

C -6.72649 2.80126 -2.95948

C -5.61214 3.52831 -3.47085

C -4.92922 4.45639 -2.71946

C -8.10923 3.12453 0.45525

C -8.99406 2.74778 1.45791

C -9.96297 1.77501 1.18910

C -10.07539 1.15661 -0.06478

C -9.16049 1.52083 -1.06829

C -8.20128 2.48897 -0.81854

C -3.61210 6.58057 -0.49660

C -2.67483 6.90375 -1.47261

C -1.68711 7.85311 -1.19367

C -1.61983 8.51589 0.03957

C -2.56437 8.18395 1.02639

C -3.53768 7.23028 0.76940

C -7.08524 -3.17900 1.39321

C -6.79471 -2.83896 2.68880

C -5.69199 -3.47049 3.33458

C -4.90792 -4.40961 2.70566

C -5.17348 -4.79327 1.35603

C -6.27629 -4.15058 0.73011

C -4.48508 -5.72929 0.55715

C -4.94956 -5.94514 -0.75972

C -6.04742 -5.29762 -1.38789

C -6.72837 -4.35276 -0.59285

C -4.15317 -6.93091 -1.41702

C -4.44971 -7.27709 -2.71001

C -5.54752 -6.64021 -3.35830

C -6.32063 -5.68905 -2.73357

C -3.32466 -6.61351 0.75065

C -2.45272 -6.81714 1.81522

C -1.41078 -7.74073 1.68676

C -1.22489 -8.49310 0.51932

C -2.10115 -8.28011 -0.55861

C -3.12701 -7.35311 -0.45138

C -7.87676 -3.45635 -0.79325

C -8.68880 -3.18572 -1.88745

C -9.72242 -2.25091 -1.76190

C -9.97429 -1.56803 -0.56273

C -9.12783 -1.82065 0.53198

C -8.10451 -2.74777 0.42362

C 4.83105 -6.90594 1.53125

C 5.44931 -7.40441 2.64879

C 6.66764 -6.80736 3.08552

C 7.24793 -5.74890 2.42552

C 6.63880 -5.19755 1.25769

C 5.42372 -5.80533 0.84135

C 7.08479 -4.12590 0.45629

C 6.29890 -3.76009 -0.65928

C 5.07901 -4.36272 -1.07090

C 4.63721 -5.43847 -0.27406

C 6.89072 -2.66207 -1.35269

C 6.26287 -2.14988 -2.45815

C 5.03678 -2.73767 -2.88530

C 4.46093 -3.80068 -2.22897

C 8.24050 -3.21644 0.47947

C 9.32944 -3.08347 1.33298

C 10.28489 -2.09220 1.08368

C 10.17336 -1.21098 0.00255

C 9.07100 -1.34101 -0.86058

C 8.12550 -2.32611 -0.62883

C 3.48509 -6.35254 -0.30358

C 2.39081 -6.47216 -1.15373

C 1.43988 -7.46778 -0.91607

C 1.55955 -8.37831 0.14475

C 2.66021 -8.24427 1.01033

C 3.60380 -7.25076 0.79583

C 6.64429 2.71441 0.99556

C 5.99742 1.97670 1.95268

C 4.73791 2.43480 2.43840

C 4.14736 3.59046 1.98266

C 4.78424 4.38695 0.98284

C 6.03758 3.91132 0.50958

C 4.33165 5.58529 0.39434

C 5.13886 6.18404 -0.59977

C 6.38781 5.70495 -1.07815

C 6.84679 4.51165 -0.48142

C 4.52710 7.37719 -1.08976

C 5.16087 8.09965 -2.06772

C 6.41380 7.63548 -2.56336

C 7.01227 6.48833 -2.09536

C 3.14714 6.44252 0.55551

C 2.02524 6.36680 1.37439

C 1.04542 7.35944 1.29416

C 1.16038 8.45586 0.42551

C 2.29117 8.52162 -0.40873

C 3.26537 7.53643 -0.34937

C 8.03469 3.65935 -0.63541

C 9.16148 3.73568 -1.44637

C 10.14168 2.74247 -1.34798

C 10.04107 1.64467 -0.47370

C 8.88701 1.58120 0.33687

C 7.91334 2.56144 0.26827

H -4.51573 7.64924 3.54104

H -6.45281 6.38886 4.43795

H -7.65476 4.74548 3.10189

H -7.22760 2.07477 -3.59116

H -5.29168 3.33669 -4.48987

H -4.08798 4.97973 -3.15563

H -8.94500 3.18616 2.44830

H -10.62916 1.48111 1.99452

H -9.19392 1.02489 -2.03239

H -2.68292 6.41994 -2.44268

H -0.94282 8.06988 -1.95183

H -2.52271 8.65961 2.00211

H -7.37926 -2.09967 3.22694

H -5.46431 -3.19494 4.35931

H -4.08166 -4.85871 3.24180

H -3.86887 -8.02003 -3.24782

H -5.77807 -6.91818 -4.38186

H -7.14611 -5.23810 -3.26951

H -2.55402 -6.26049 2.73990

H -0.72049 -7.86691 2.51377

H -1.96765 -8.83139 -1.48526

H -8.53331 -3.67721 -2.84128

H -10.32704 -2.03995 -2.63837

H -9.26522 -1.27012 1.45619

H 5.03265 -8.23779 3.20591

H 7.15191 -7.20543 3.97160

H 8.17523 -5.33284 2.79858

H 6.67351 -1.30805 -3.00614

H 4.54080 -2.32560 -3.75802

H 3.52629 -4.20750 -2.59297

H 9.45665 -3.73296 2.19158

H 11.12370 -2.01235 1.76516

H 8.95096 -0.65538 -1.69008

H 2.25281 -5.79978 -1.99265

H 0.57813 -7.52849 -1.57057

H 2.77074 -8.91096 1.86040

H 6.41838 1.05440 2.33962

H 4.22765 1.84419 3.19233

H 3.18713 3.89094 2.38169

H 4.73054 9.01087 -2.47159

H 6.91035 8.21058 -3.33853

H 7.96497 6.17891 -2.50602

H 1.88854 5.54521 2.06807

H 0.16375 7.26936 1.91782

H 2.40174 9.33784 -1.11624

H 9.29549 4.54864 -2.15151

H 11.00732 2.83191 -1.99695

H 8.75790 0.74974 1.02026

Pt 0.23011 -9.89737 0.37855

Pt -11.50846 -0.24230 -0.40804

Pt -0.22711 9.94094 0.41348

Pt 11.56650 0.20223 -0.37414

C 12.91310 -1.34398 -1.09976

H 12.23958 -2.16272 -1.33796

C 13.36646 1.81187 -0.06747

H 12.82457 2.68940 0.26652

C 1.29261 11.76736 0.28197

C 1.14172 11.40096 1.59625

H 2.11999 11.31568 -0.25367

H 1.87397 10.70926 2.00041

C -1.62149 11.64666 -0.30714

C -1.77807 11.59714 1.05737

H -2.33688 11.08844 -0.90292

H -2.59004 10.98003 1.42718

C -13.20065 1.07853 0.48899

H -12.61943 1.80735 1.04337

C -13.03496 -1.63976 -1.48124

H -12.36709 -2.34064 -1.96955

C -1.24497 -11.68821 -0.02767

C -1.11349 -11.52763 1.33098

H -2.06902 -11.16259 -0.49893

C 1.67663 -11.46367 -0.54965

H -1.85888 -10.91343 1.82645

C 1.81498 -11.61872 0.80715

H 2.38902 -10.81159 -1.04487

H 2.61248 -11.05602 1.27977

C 14.37008 0.52739 -2.02348

H 15.21068 0.30049 -1.36157

H 14.80079 0.91573 -2.95033

C 13.50452 1.59296 -1.40137

H 13.03525 2.29605 -2.08203

C 13.18551 -0.44467 0.90487

H 12.68324 -0.71208 1.82991

C 13.77120 -1.60438 0.12940

H 13.54318 -2.56485 0.59371

H 14.85503 -1.55700 -0.02900

C 0.61211 12.93569 -0.40583

H 0.46742 13.75067 0.30381

H 1.28967 13.32642 -1.16925

C -0.72406 12.57348 -1.09125

H -0.50876 12.08387 -2.04506

H -1.26944 13.49330 -1.34178

C 0.21981 12.02358 2.61735

H 0.02870 11.26666 3.38284

H 0.73758 12.84557 3.12956

C -1.13301 12.52827 2.06711

H -1.02119 13.52316 1.63537

H -1.81811 12.64808 2.91016

C -14.37041 -1.21475 0.67380

H -15.25624 -1.10983 0.04625

H -14.64813 -1.91830 1.46248

C -13.24419 -1.82721 -0.13768

H -12.69365 -2.63440 0.33386

C -13.27650 1.25933 -0.86991

H -12.71763 2.09234 -1.28323

C -14.28270 0.59784 -1.79286

H -14.50919 1.28981 -2.60774

H -15.22335 0.45021 -1.26088

C -13.80240 -0.73317 -2.41256

H -13.14668 -0.51016 -3.25879

H -14.66214 -1.27191 -2.83302

C -14.01981 0.13592 1.33673

H -13.44754 -0.05822 2.24813

H -14.94304 0.63365 1.66239

C 13.59661 -0.76976 -2.32451

H 14.29558 -1.51409 -2.73568

H 12.85247 -0.57485 -3.10251

C 13.95429 0.87978 0.98144

H 13.82569 1.32370 1.96935

H 15.02982 0.72962 0.82912

C 0.79803 -12.27020 -1.47592

H 1.35461 -13.14059 -1.84833

H 0.59577 -11.64718 -2.35136

C -0.54724 -12.73612 -0.87488

H -0.40937 -13.64905 -0.29500

H -1.21054 -13.00812 -1.69976

C 1.16543 -12.69670 1.65400

H 1.06749 -13.61429 1.07333

H 1.84138 -12.93995 2.47768

C -0.19822 -12.29188 2.25713

H -0.02197 -11.65904 3.13137

H -0.71306 -13.18631 2.63279

**XYZ coordinates of** **12:**

**Element X Y Z**

C 3.82983 0.10830 -0.47196

C 4.59883 -0.96849 -0.01617

C 5.90963 -0.70487 0.40129

C 5.65426 1.65071 -0.07700

C 4.35299 1.39662 -0.50922

C 5.75863 3.82123 -1.31865

C 4.88594 4.91037 -1.23985

C 4.92442 4.83729 1.31291

C 5.81665 3.76455 1.23419

C 5.77045 4.06792 -3.75258

C 4.92316 5.16695 -3.64900

C 4.47613 5.56807 -2.40539

C 5.15143 4.78437 3.74856

C 6.06254 3.73735 3.64448

C 6.37589 3.22762 2.39984

C 2.78130 5.18712 0.06723

C 2.26212 3.96632 0.49715

C 0.89327 3.72215 0.45860

C 0.00000 4.69833 0.00287

C 0.53048 5.92596 -0.41358

C 2.77684 -2.63467 0.45860

C 4.06887 -2.34916 0.00287

C 4.86680 -3.42239 -0.41358

C 3.10153 -5.00224 0.06723

C 2.30387 -3.94221 0.49715

C 1.72701 -6.68331 1.31291

C 0.35187 -6.91964 1.23419

C 0.42997 -6.89774 -1.31865

C 1.80953 -6.68653 -1.23985

C 1.56767 -6.85345 3.74856

C 0.20537 -7.11899 3.64448

C -0.39274 -7.13549 2.39984

C 0.63769 -7.03131 -3.75258

C 2.01313 -6.84706 -3.64900

C 2.58403 -6.66048 -2.40539

C -1.39757 -5.72209 -0.07700

C -0.96698 -4.46811 -0.50922

C -1.82113 -3.37088 -0.47196

C -3.13815 -3.49846 -0.01617

C -3.56526 -4.76545 0.40129

C -2.00870 3.26258 -0.47196

C -1.46068 4.46695 -0.01617

C -2.34438 5.47033 0.40129

C -4.25669 4.07138 -0.07700

C -3.38600 3.07149 -0.50922

C -6.18860 3.07651 -1.31865

C -6.69547 1.77616 -1.23985

C -6.65142 1.84603 1.31291

C -6.16852 3.15509 1.23419

C -6.40814 2.96340 -3.75258

C -6.93629 1.68011 -3.64900

C -7.06016 1.09241 -2.40539

C -6.71910 2.06909 3.74856

C -6.26791 3.38164 3.64448

C -5.98315 3.90787 2.39984

C -5.88283 -0.18488 0.06723

C -4.56599 -0.02410 0.49715

C -3.67011 -1.08748 0.45860

C -4.06887 -2.34916 0.00287

C -5.39727 -2.50358 -0.41358

H 2.82575 -0.06978 -0.84232

H 6.52524 -1.51957 0.76724

H 3.74813 2.19780 -0.91412

H 6.11565 3.71521 -4.71614

H 4.60430 5.69227 -4.54235

H 3.79724 6.40308 -2.31913

H 4.86749 5.18660 4.71274

H 6.50865 3.31573 4.53831

H 7.06250 2.39872 2.31267

H 2.91978 3.20791 0.90209

H 0.51029 2.77660 0.82781

H -0.13828 6.69750 -0.77981

H 2.14946 -1.83023 0.82781

H 5.86934 -3.22899 -0.77981

H 1.31824 -4.13256 0.90209

H 2.05798 -6.80867 4.71274

H -0.38282 -7.29453 4.53831

H -1.45390 -7.31566 2.31267

H 0.15964 -7.15391 -4.71614

H 2.62750 -6.83357 -4.54235

H 3.64661 -6.49004 -2.31913

H 0.02929 -4.34487 -0.91412

H -1.47331 -2.41228 -0.84232

H -4.57860 -4.89124 0.76724

H -1.35244 2.48206 -0.84232

H -1.94664 6.41081 0.76724

H -3.77741 2.14707 -0.91412

H -6.27529 3.43870 -4.71614

H -7.23180 1.14131 -4.54235

H -7.44385 0.08697 -2.31913

H -6.92547 1.62207 4.71274

H -6.12584 3.97879 4.53831

H -5.60860 4.91694 2.31267

H -4.23803 0.92465 0.90209

H -2.65976 -0.94638 0.82781

H -5.73106 -3.46850 -0.77981

C 2.57792 -6.45215 0.07036

C 6.23452 3.07897 -0.07627

C 4.29876 5.45862 0.07036

C -0.45080 -6.93874 -0.07627

C -5.78373 3.85977 -0.07627

C -6.87669 0.99353 0.07036

O 3.72051 -7.29367 0.06382

O 4.45625 6.86890 0.06382

O -8.17676 0.42478 0.06382

O -1.27675 -8.09202 -0.06732

O 7.64626 2.94031 -0.06732

O -6.36952 5.15170 -0.06732

C 2.30505 -6.62825 2.59797

C 6.16387 3.40361 -2.60291

C 4.58771 5.31036 2.59797

C -0.13432 -7.03987 -2.60291

C -6.02955 3.63626 -2.60291

C -6.89276 1.31790 2.59797

C 4.39248 -4.72587 -0.38143

H 5.03481 -5.54432 -0.67583

C 6.42884 0.58151 0.37130

H 7.45350 0.76028 0.66676

C 1.89649 6.16693 -0.38143

H 2.28412 7.13243 -0.67583

C -2.71082 -5.85830 0.37130

H -3.06833 -6.83506 0.66676

C -3.71803 5.27679 0.37130

H -4.38517 6.07478 0.66676

C -6.28896 -1.44106 -0.38143

H -7.31893 -1.58811 -0.67583

Cl 4.00517 -6.24978 2.83924

Cl 7.18063 1.98933 -2.84617

Cl 3.40988 6.59347 2.83924

Cl -1.86750 -7.21327 -2.84617

Cl -5.31313 5.22394 -2.84617

Cl -7.41505 -0.34369 2.83924

Si 5.68854 7.99952 0.20170

Si -1.10650 -9.75534 -0.20341

Si 4.08351 -8.92618 0.20170

Si 9.00162 3.91941 -0.20341

Si -7.89512 5.83592 -0.20341

Si -9.77206 0.92667 0.20170

C 8.70038 5.32651 -1.42440

C 9.71384 6.47963 -1.37556

H 7.69452 5.72056 -1.26632

H 8.67498 4.89177 -2.42973

H 9.46812 7.25345 -2.10829

H 9.73193 6.95814 -0.39480

H 10.73090 6.14250 -1.58918

C 10.31703 2.72742 -0.82656

C 11.72760 3.32072 -0.96459

H 9.98162 2.32584 -1.78772

H 10.33760 1.86994 -0.14383

H 12.45797 2.55837 -1.25026

H 11.76306 4.10013 -1.72998

H 12.07817 3.76953 -0.03041

C 9.49967 4.56341 1.50116

C 10.03557 3.48150 2.45144

H 8.64285 5.06154 1.96280

H 10.25856 5.34417 1.37119

H 10.26202 3.89159 3.43987

H 9.31202 2.67514 2.59464

H 10.95280 3.02562 2.07030

C 7.06381 7.35872 1.31587

C 8.20439 8.34661 1.60444

H 7.46914 6.45734 0.85145

H 6.62569 7.02150 2.25945

H 9.02326 7.85767 2.14005

H 8.62492 8.77485 0.69112

H 7.86495 9.17797 2.22611

C 4.80819 9.50517 0.91086

C 5.59072 10.82739 0.86561

H 4.51441 9.27564 1.94013

H 3.86578 9.61325 0.36082

H 4.98721 11.66070 1.23676

H 6.49426 10.78826 1.47800

H 5.90382 11.08399 -0.15001

C 6.36801 8.40883 -1.51115

C 5.36813 9.10182 -2.44914

H 6.73066 7.48849 -1.97857

H 7.25506 9.04210 -1.38793

H 5.80259 9.28667 -3.43583

H 4.47142 8.49659 -2.59953

H 5.04030 10.06588 -2.05255

C -8.96308 4.87150 -1.42440

C -10.46844 5.17262 -1.37556

H -8.80141 3.80337 -1.26632

H -8.57389 5.06687 -2.42973

H -11.01573 4.57291 -2.10829

H -10.89189 4.94903 -0.39480

H -10.68500 6.22198 -1.58918

C -7.52053 7.57110 -0.82656

C -8.73963 8.49604 -0.96459

H -7.00504 7.48142 -1.78772

H -6.78822 8.01766 -0.14383

H -8.44460 9.50973 -1.25026

H -9.43235 8.13705 -1.72998

H -9.30359 8.57524 -0.03041

C -10.46627 1.31044 -1.51115

C -10.56648 0.09803 -2.44914

H -9.85055 2.08468 -1.97857

H -11.45822 1.76202 -1.38793

H -10.94379 0.38185 -3.43583

H -9.59397 -0.37593 -2.59953

H -11.23746 -0.66792 -2.05255

C -10.63581 -0.58857 0.91086

C -12.17216 -0.57199 0.86561

H -10.29015 -0.72823 1.94013

H -10.25820 -1.45876 0.36082

H -12.59207 -1.51129 1.23676

H -12.59004 0.23006 1.47800

H -12.55093 -0.42913 -0.15001

C -9.90474 2.43808 1.31587

C -11.33057 2.93191 1.60444

H -9.32679 3.23979 0.85145

H -9.39364 2.22726 2.25945

H -11.31657 3.88553 2.14005

H -11.91170 3.08197 0.69112

H -11.88083 2.22226 2.22611

C -8.70186 5.94525 1.50116

C -8.03286 6.95031 2.45144

H -8.70484 4.95416 1.96280

H -9.75747 6.21209 1.37119

H -8.50123 6.94137 3.43987

H -6.97274 6.72688 2.59464

H -8.09667 7.97259 2.07030

C 5.82762 -8.91660 0.91086

C 6.58143 -10.25541 0.86561

H 5.77574 -8.54741 1.94013

H 6.39243 -8.15448 0.36082

H 7.60485 -10.14940 1.23676

H 6.09578 -11.01832 1.47800

H 6.64711 -10.65486 -0.15001

C 0.26270 -10.19800 -1.42440

C 0.75460 -11.65224 -1.37556

H 1.10689 -9.52393 -1.26632

H -0.10110 -9.95864 -2.42973

H 1.54761 -11.82636 -2.10829

H 1.15996 -11.90717 -0.39480

H -0.04589 -12.36448 -1.58918

C -2.79650 -10.29852 -0.82656

C -2.98797 -11.81676 -0.96459

H -2.97658 -9.80726 -1.78772

H -3.54939 -9.88760 -0.14383

H -4.01337 -12.06810 -1.25026

H -2.33072 -12.23718 -1.72998

H -2.77458 -12.34477 -0.03041

C 2.84093 -9.79680 1.31587

C 3.12618 -11.27852 1.60444

H 1.85765 -9.69713 0.85145

H 2.76796 -9.24877 2.25945

H 2.29332 -11.74321 2.14005

H 3.28679 -11.85682 0.69112

H 4.01588 -11.40024 2.22611

C -0.79781 -10.50865 1.50116

C -2.00272 -10.43181 2.45144

H 0.06200 -10.01569 1.96280

H -0.50109 -11.55626 1.37119

H -1.76079 -10.83297 3.43987

H -2.33927 -9.40201 2.59464

H -2.85613 -10.99821 2.07030

C 4.09826 -9.71927 -1.51115

C 5.19834 -9.19985 -2.44914

H 3.11989 -9.57317 -1.97857

H 4.20316 -10.80412 -1.38793

H 5.14120 -9.66853 -3.43583

H 5.12255 -8.12066 -2.59953

H 6.19716 -9.39796 -2.05255

**XYZ coordinates of 1****4:**

**Element X Y Z**

C 0.86595 6.15876 1.83255

C 1.69192 6.40564 0.71746

C 3.03179 6.05365 0.69851

H 3.62699 6.26277 -0.18027

C 3.57571 5.39507 1.79550

H 4.61908 5.10464 1.79222

C 2.77417 5.09216 2.87849

H 3.19392 4.57191 3.72592

C 1.42690 5.47090 2.91426

C -0.71620 5.73851 4.27998

C -1.49188 5.62394 5.45195

C -2.76023 6.17219 5.55067

H -3.31519 6.06694 6.47423

C -3.30713 6.82869 4.45238

H -4.30291 7.25325 4.51601

C -2.58261 6.91794 3.27989

H -3.00883 7.40505 2.41552

C -1.29142 6.38533 3.18468

Cl 1.07973 7.12876 -0.76582

Cl -0.92717 4.74327 6.86454

C 0.44963 3.54379 4.20887

C -0.72813 2.96581 3.73155

H -1.52928 3.58792 3.35753

C -0.90471 1.58728 3.73301

H -1.82474 1.16852 3.33938

C 0.09457 0.73466 4.21032

C 1.27206 1.31800 4.69825

H 2.04778 0.68189 5.11128

C 1.44159 2.69779 4.70786

H 2.33292 3.12466 5.14793

C -1.32058 5.49640 0.87328

C -0.86595 4.18032 0.82690

H 0.01163 3.88689 1.38194

C -1.54401 3.21404 0.10486

H -1.18573 2.19136 0.12023

C -2.71381 3.52676 -0.59566

C -3.15212 4.85671 -0.57876

H -4.03628 5.13462 -1.14362

C -2.45802 5.83122 0.13704

H -2.79010 6.86135 0.11612

C -3.96179 -2.39287 -2.19685

C -3.35671 -2.54419 -0.93088

C -2.18114 -3.25075 -0.75662

H -1.75592 -3.33467 0.23219

C -1.55533 -3.82334 -1.85955

H -0.62749 -4.36461 -1.72855

C -2.13079 -3.69447 -3.10811

H -1.65468 -4.14494 -3.96641

C -3.32765 -2.98798 -3.28992

C -5.19452 -2.21540 -4.86124

C -5.86607 -2.16342 -6.09854

C -7.05643 -1.47296 -6.26149

H -7.54107 -1.46856 -7.22959

C -7.59163 -0.77160 -5.18518

H -8.51456 -0.21550 -5.30725

C -6.93385 -0.77829 -3.97022

H -7.33725 -0.22889 -3.13141

C -5.74754 -1.50062 -3.79481

Cl -4.00155 -1.79050 0.52458

Cl -5.21624 -2.92682 -7.54331

C -2.80626 -2.01951 -5.50075

C -2.89545 -0.62940 -5.45526

H -3.78380 -0.15048 -5.06726

C -1.81469 0.16281 -5.81465

H -1.87583 1.23243 -5.65093

C -0.61593 -0.40850 -6.25484

C -0.60181 -1.79596 -6.46832

H 0.27912 -2.26485 -6.89334

C -1.67888 -2.58947 -6.09481

H -1.64169 -3.66233 -6.23252

C -4.58373 -0.02079 -2.09995

C -3.26002 0.29067 -2.41709

H -2.63675 -0.42343 -2.93407

C -2.69187 1.48602 -2.00996

H -1.64966 1.68431 -2.23646

C -3.42542 2.41179 -1.25989

C -4.77534 2.13656 -1.01565

H -5.36109 2.82927 -0.41979

C -5.34497 0.93241 -1.42495

H -6.35953 0.69160 -1.13578

C -0.61947 6.50458 1.80577

C 0.65258 5.07726 4.18627

C -5.13418 -1.43739 -2.38979

C -3.83152 -2.88432 -4.74357

C -0.86595 -6.15876 1.83255

C -1.69192 -6.40564 0.71746

C -3.03179 -6.05365 0.69851

H -3.62699 -6.26277 -0.18027

C -3.57571 -5.39507 1.79550

H -4.61908 -5.10464 1.79222

C -2.77417 -5.09216 2.87849

H -3.19392 -4.57191 3.72592

C -1.42690 -5.47090 2.91426

C 0.71620 -5.73851 4.27998

C 1.49188 -5.62394 5.45195

C 2.76023 -6.17219 5.55067

H 3.31519 -6.06694 6.47423

C 3.30713 -6.82869 4.45238

H 4.30291 -7.25325 4.51601

C 2.58261 -6.91794 3.27989

H 3.00883 -7.40505 2.41552

C 1.29142 -6.38533 3.18468

Cl -1.07973 -7.12876 -0.76582

Cl 0.92717 -4.74327 6.86454

C -0.44963 -3.54379 4.20887

C 0.72813 -2.96581 3.73155

H 1.52928 -3.58792 3.35753

C 0.90471 -1.58728 3.73301

H 1.82474 -1.16852 3.33938

C -0.09457 -0.73466 4.21032

C -1.27206 -1.31800 4.69825

H -2.04778 -0.68189 5.11128

C -1.44159 -2.69779 4.70786

H -2.33292 -3.12466 5.14793

C 1.32058 -5.49640 0.87328

C 0.86595 -4.18032 0.82690

H -0.01163 -3.88689 1.38194

C 1.54401 -3.21404 0.10486

H 1.18573 -2.19136 0.12023

C 2.71381 -3.52676 -0.59566

C 3.15212 -4.85671 -0.57876

H 4.03628 -5.13462 -1.14362

C 2.45802 -5.83122 0.13704

H 2.79010 -6.86135 0.11612

C 3.96179 2.39287 -2.19685

C 3.35671 2.54419 -0.93088

C 2.18114 3.25075 -0.75662

H 1.75592 3.33467 0.23219

C 1.55533 3.82334 -1.85955

H 0.62749 4.36461 -1.72855

C 2.13079 3.69447 -3.10811

H 1.65468 4.14494 -3.96641

C 3.32765 2.98798 -3.28992

C 5.19452 2.21540 -4.86124

C 5.86607 2.16342 -6.09854

C 7.05643 1.47296 -6.26149

H 7.54107 1.46856 -7.22959

C 7.59163 0.77160 -5.18518

H 8.51456 0.21550 -5.30725

C 6.93385 0.77829 -3.97022

H 7.33725 0.22889 -3.13141

C 5.74754 1.50062 -3.79481

Cl 4.00155 1.79050 0.52458

Cl 5.21624 2.92682 -7.54331

C 2.80626 2.01951 -5.50075

C 2.89545 0.62940 -5.45526

H 3.78380 0.15048 -5.06726

C 1.81469 -0.16281 -5.81465

H 1.87583 -1.23243 -5.65093

C 0.61593 0.40850 -6.25484

C 0.60181 1.79596 -6.46832

H -0.27912 2.26485 -6.89334

C 1.67888 2.58947 -6.09481

H 1.64169 3.66233 -6.23252

C 4.58373 0.02079 -2.09995

C 3.26002 -0.29067 -2.41709

H 2.63675 0.42343 -2.93407

C 2.69187 -1.48602 -2.00996

H 1.64966 -1.68431 -2.23646

C 3.42542 -2.41179 -1.25989

C 4.77534 -2.13656 -1.01565

H 5.36109 -2.82927 -0.41979

C 5.34497 -0.93241 -1.42495

H 6.35953 -0.69160 -1.13578

C 0.61947 -6.50458 1.80577

C -0.65258 -5.07726 4.18627

C 5.13418 1.43739 -2.38979

C 3.83152 2.88432 -4.74357

O 1.46880 5.41223 5.29917

O -0.85882 7.80939 1.31170

O 3.81099 4.18397 -5.30445

O -3.81099 -4.18397 -5.30445

O -6.18061 -1.67172 -1.46098

O 6.18061 1.67172 -1.46098

O 0.85882 -7.80939 1.31170

O -1.46880 -5.41223 5.29917

Si -2.01977 -6.84271 6.00352

Si 2.01977 6.84271 6.00352

Si 4.60901 5.63505 -4.99488

Si -4.60901 -5.63505 -4.99488

Si 0.34529 -9.35518 1.75537

Si -0.34529 9.35518 1.75537

Si -7.13377 -2.98983 -1.03694

Si 7.13377 2.98983 -1.03694

C -2.31162 -6.30696 7.78670

C -3.35927 -7.08902 8.59256

H -2.59035 -5.24769 7.74825

H -1.34596 -6.32737 8.30386

H -3.44401 -6.70381 9.61286

H -4.35105 -7.01759 8.13846

H -3.11309 -8.15129 8.66908

C 2.31162 6.30696 7.78670

C 3.35927 7.08902 8.59256

H 2.59035 5.24769 7.74825

H 1.34596 6.32737 8.30386

H 3.44401 6.70381 9.61286

H 4.35105 7.01759 8.13846

H 3.11309 8.15129 8.66908

C 4.36298 6.55106 -6.62302

C 4.42460 8.08505 -6.58246

H 3.39016 6.23171 -7.01422

H 5.09799 6.16148 -7.33664

H 4.29111 8.51505 -7.57939

H 3.63988 8.50072 -5.94512

H 5.38094 8.45061 -6.19945

C -4.36298 -6.55106 -6.62302

C -4.42460 -8.08505 -6.58246

H -3.39016 -6.23171 -7.01422

H -5.09799 -6.16148 -7.33664

H -4.29111 -8.51505 -7.57939

H -3.63988 -8.50072 -5.94512

H -5.38094 -8.45061 -6.19945

C -0.93080 -9.37977 6.88389

H -0.16053 -10.14543 6.75141

H -0.86301 -9.03075 7.91748

H -1.89862 -9.87186 6.77037

C 0.93080 9.37977 6.88389

H 0.16053 10.14543 6.75141

H 0.86301 9.03075 7.91748

H 1.89862 9.87186 6.77037

C 7.35474 6.55388 -4.80775

H 8.39347 6.30302 -4.57270

H 7.33828 6.92783 -5.83463

H 7.06623 7.38189 -4.15686

C -7.35474 -6.55388 -4.80775

H -8.39347 -6.30302 -4.57270

H -7.33828 -6.92783 -5.83463

H -7.06623 -7.38189 -4.15686

C -3.62470 -7.35701 5.14606

H -3.45025 -7.33077 4.06679

C 3.62470 7.35701 5.14606

H 3.45025 7.33077 4.06679

C 3.76169 6.50939 -3.54783

H 3.65559 5.78680 -2.73465

C -3.76169 -6.50939 -3.54783

H -3.65559 -5.78680 -2.73465

C -1.98980 -10.60913 2.95669

H -4.36932 -6.57242 5.33238

H -3.06879 -10.55784 3.12398

H -1.79369 -11.52498 2.39533

H -1.51667 -10.71943 3.93535

C 1.98980 10.60913 2.95669

H 4.36932 6.57242 5.33238

H 3.06879 10.55784 3.12398

H 1.79369 11.52498 2.39533

H 1.51667 10.71943 3.93535

C -6.82360 -5.87943 -0.73109

H -2.73119 -6.73217 -3.85101

H -6.13042 -6.70055 -0.52823

H -7.53836 -5.85300 0.09341

H -7.37896 -6.14559 -1.63303

C 6.82360 5.87943 -0.73109

H 2.73119 6.73217 -3.85101

H 6.13042 6.70055 -0.52823

H 7.53836 5.85300 0.09341

H 7.37896 6.14559 -1.63303

C 1.42690 -11.53783 3.33817

H 2.11025 -11.84331 4.13591

H 0.43803 -11.92004 3.59792

H 1.74514 -12.05298 2.42792

C -1.42690 11.53783 3.33817

H -2.11025 11.84331 4.13591

H -0.43803 11.92004 3.59792

H -1.74514 12.05298 2.42792

C -9.72279 -4.05666 -1.79901

H -10.53617 -4.05988 -2.53039

H -9.41431 -5.09421 -1.65684

H -10.14472 -3.71088 -0.85120

C 9.72279 4.05666 -1.79901

H 10.53617 4.05988 -2.53039

H 9.41431 5.09421 -1.65684

H 10.14472 3.71088 -0.85120

C 0.66633 -10.33928 0.17866

C -0.19195 -11.59652 -0.02974

H 1.73030 -10.60475 0.14745

H 0.51099 -9.64801 -0.65501

H 0.05115 -12.09242 -0.97386

H -0.04975 -12.33188 0.76634

H -1.25688 -11.35148 -0.05983

C -0.66633 10.33928 0.17866

C 0.19195 11.59652 -0.02974

H -1.73030 10.60475 0.14745

H -0.51099 9.64801 -0.65501

H -0.05115 12.09242 -0.97386

H 0.04975 12.33188 0.76634

H 1.25688 11.35148 -0.05983

C -7.78944 -2.45377 0.65037

C -8.14943 -3.56479 1.64798

H -8.65809 -1.80296 0.49096

H -7.01643 -1.81107 1.08323

H -8.50037 -3.14960 2.59714

H -8.93881 -4.22076 1.27179

H -7.28438 -4.19463 1.87349

C 7.78944 2.45377 0.65037

C 8.14943 3.56479 1.64798

H 8.65809 1.80296 0.49096

H 7.01643 1.81107 1.08323

H 8.50037 3.14960 2.59714

H 8.93881 4.22076 1.27179

H 7.28438 4.19463 1.87349

C -6.07611 -4.54906 -0.90548

H -5.42318 -4.61227 -1.77848

H -5.39615 -4.38494 -0.06563

C -1.48655 -9.35497 2.22485

H -1.69725 -8.47571 2.83873

H -2.06627 -9.20204 1.30845

C 1.48655 9.35497 2.22485

H 1.69725 8.47571 2.83873

H 2.06627 9.20204 1.30845

C 6.07611 4.54906 -0.90548

H 5.42318 4.61227 -1.77848

H 5.39615 4.38494 -0.06563

C -4.42525 -7.78204 -2.99989

H -5.42043 -7.57536 -2.60291

H -4.53374 -8.55796 -3.76011

H -3.83290 -8.20794 -2.18446

C -4.20794 -8.72889 5.52079

H -3.52574 -9.53696 5.25182

H -4.41522 -8.81846 6.58886

H -5.14755 -8.91479 4.99190

C 4.20794 8.72889 5.52079

H 3.52574 9.53696 5.25182

H 4.41522 8.81846 6.58886

H 5.14755 8.91479 4.99190

C 4.42525 7.78204 -2.99989

H 5.42043 7.57536 -2.60291

H 4.53374 8.55796 -3.76011

H 3.83290 8.20794 -2.18446

C 0.73518 8.22202 5.89072

H -0.25507 7.78917 6.04848

H 0.72336 8.60768 4.86774

C -6.43811 -5.33050 -4.64423

H -6.78115 -4.53746 -5.31446

H -6.54200 -4.92243 -3.63620

C -0.73518 -8.22202 5.89072

H 0.25507 -7.78917 6.04848

H -0.72336 -8.60768 4.86774

C 6.43811 5.33050 -4.64423

H 6.78115 4.53746 -5.31446

H 6.54200 4.92243 -3.63620

C -1.43525 10.01076 3.15472

H -1.18002 9.51543 4.09613

H -2.45872 9.68939 2.93298

C -8.56060 -3.16248 -2.26193

H -8.18774 -3.50002 -3.23296

H -8.93853 -2.14913 -2.44000

C 1.43525 -10.01076 3.15472

H 1.18002 -9.51543 4.09613

H 2.45872 -9.68939 2.93298

C 8.56060 3.16248 -2.26193

H 8.18774 3.50002 -3.23296

H 8.93853 2.14913 -2.44000

**XYZ coordinates of** **13:**

**Element X Y Z**

C 1.45001 3.61844 0.45459

C 0.73919 4.74365 0.01944

C 1.47506 5.87169 -0.36461

C 3.56266 4.74050 0.08785

C 2.83847 3.61917 0.49288

C 5.60524 3.99240 1.31949

C 6.29390 2.78048 1.24091

C 6.26014 2.85808 -1.31949

C 5.55492 4.06043 -1.24091

C 5.82118 3.90281 3.75316

C 6.51825 2.70265 3.65048

C 6.74607 2.14784 2.40571

C 6.29052 3.08989 -3.75316

C 5.59969 4.29364 -3.65048

C 5.23312 4.76835 -2.40571

C 5.88672 0.71511 -0.08785

C 4.55352 0.64860 -0.49288

C 3.85867 -0.55347 -0.45459

C 4.47771 -1.73167 -0.01944

C 5.82256 -1.65840 0.36461

C -1.45001 3.61844 -0.45459

C -0.73919 4.74365 -0.01944

C -1.47506 5.87169 0.36461

C -3.56266 4.74050 -0.08785

C -2.83847 3.61917 -0.49288

C -5.60524 3.99240 -1.31949

C -6.29390 2.78048 -1.24091

C -6.26014 2.85808 1.31949

C -5.55492 4.06043 1.24091

C -5.82118 3.90281 -3.75316

C -6.51825 2.70265 -3.65048

C -6.74607 2.14784 -2.40571

C -6.29052 3.08989 3.75316

C -5.59969 4.29364 3.65048

C -5.23312 4.76835 2.40571

C -5.88672 0.71511 0.08785

C -4.55352 0.64860 0.49288

C -3.85867 -0.55347 0.45459

C -4.47771 -1.73167 0.01944

C -5.82256 -1.65840 -0.36461

C 2.40866 -3.06497 0.45459

C 3.73852 -3.01198 0.01944

C 4.34750 -4.21328 -0.36461

C 2.32406 -5.45561 0.08785

C 1.71505 -4.26777 0.49288

C 0.65490 -6.85048 1.31949

C -0.73898 -6.84091 1.24091

C -0.65490 -6.85048 -1.31949

C 0.73898 -6.84091 -1.24091

C 0.46934 -6.99270 3.75316

C -0.91856 -6.99629 3.65048

C -1.51295 -6.91619 2.40571

C -0.46934 -6.99270 -3.75316

C 0.91856 -6.99629 -3.65048

C 1.51295 -6.91619 -2.40571

C -2.32406 -5.45561 -0.08785

C -1.71505 -4.26777 -0.49288

C -2.40866 -3.06497 -0.45459

C -3.73852 -3.01198 -0.01944

C -4.34750 -4.21328 0.36461

H 0.90914 2.74513 0.80281

H 0.95239 6.75580 -0.71344

H 3.35561 2.74715 0.87269

H 5.61905 4.35222 4.71716

H 6.87177 2.20446 4.54647

H 7.28525 1.21521 2.31769

H 6.57866 2.69013 -4.71716

H 5.34501 4.84889 -4.54647

H 4.69502 5.70161 -2.31769

H 4.05691 1.53247 -0.87269

H 2.83192 -0.58523 -0.80281

H 6.32689 -2.55311 0.71344

H -0.90914 2.74513 -0.80281

H -0.95239 6.75580 0.71344

H -3.35561 2.74715 -0.87269

H -5.61905 4.35222 -4.71716

H -6.87177 2.20446 -4.54647

H -7.28525 1.21521 -2.31769

H -6.57866 2.69013 4.71716

H -5.34501 4.84889 4.54647

H -4.69502 5.70161 2.31769

H -4.05691 1.53247 0.87269

H -2.83192 -0.58523 0.80281

H -6.32689 -2.55311 -0.71344

H 1.92278 -2.15990 0.80281

H 5.37450 -4.20269 -0.71344

H 0.70130 -4.27962 0.87269

H 0.95961 -7.04235 4.71716

H -1.52676 -7.05336 4.54647

H -2.59023 -6.91681 2.31769

H -0.95961 -7.04235 -4.71716

H 1.52676 -7.05336 -4.54647

H 2.59023 -6.91681 -2.31769

H -0.70130 -4.27962 -0.87269

H -1.92278 -2.15990 -0.80281

H -5.37450 -4.20269 0.71344

C -5.09642 4.70700 -0.07494

C 5.09642 4.70700 0.07494

C 6.62459 2.06013 -0.07494

C -6.62459 2.06013 0.07494

C 1.52817 -6.76713 0.07494

C -1.52817 -6.76713 -0.07494

C -8.89728 2.86456 0.11177

H -9.90466 2.45946 0.03026

H -8.71975 3.56139 -0.71442

H -8.80347 3.40246 1.06068

C -6.92942 6.27299 -0.11177

H -7.08228 7.34796 -0.03026

H -7.44412 5.77083 0.71442

H -7.34835 5.92280 -1.06068

C 6.92942 6.27299 0.11177

H 7.08228 7.34796 0.03026

H 7.44412 5.77083 -0.71442

H 7.34835 5.92280 1.06068

C 8.89728 2.86456 -0.11177

H 9.90466 2.45946 -0.03026

H 8.71975 3.56139 0.71442

H 8.80347 3.40246 -1.06068

C 1.96786 -9.13755 0.11177

H 2.82238 -9.80741 0.03026

H 1.27562 -9.33221 -0.71442

H 1.45512 -9.32526 1.06068

C -1.96786 -9.13755 -0.11177

H -2.82238 -9.80741 -0.03026

H -1.27562 -9.33221 0.71442

H -1.45512 -9.32526 -1.06068

O -5.52830 6.07336 -0.05608

O 8.02384 1.75097 -0.05608

O -2.49553 -7.82433 -0.05608

O -8.02384 1.75097 0.05608

O 5.52830 6.07336 0.05608

O 2.49553 -7.82433 0.05608

C -5.37178 4.53012 -2.60244

C 5.37178 4.53012 2.60244

C 6.60909 2.38704 -2.60244

C -6.60909 2.38704 2.60244

C 1.23731 -6.91716 2.60244

C -1.23731 -6.91716 -2.60244

C -2.86384 5.87209 0.33128

H -3.41318 6.76180 0.60738

C 2.86384 5.87209 -0.33128

H 3.41318 6.76180 -0.60738

C 6.51730 -0.45589 0.33128

H 7.56248 -0.42500 0.60738

C -6.51730 -0.45589 -0.33128

H -7.56248 -0.42500 -0.60738

C 3.65346 -5.41620 -0.33128

H 4.14930 -6.33680 -0.60738

C -3.65346 -5.41620 0.33128

H -4.14930 -6.33680 0.60738

Cl -4.48164 6.02637 -2.83842

Cl 4.48164 6.02637 2.83842

Cl 7.45980 0.86803 -2.83842

Cl -7.45980 0.86803 2.83842

Cl 2.97817 -6.89439 2.83842

Cl -2.97817 -6.89439 -2.83842

**XYZ coordinates of** **15:**

**Element X Y Z**

C 6.36969 1.93127 1.11712

C 5.68669 2.42506 -0.00416

C 5.41865 1.55309 -1.06977

C 5.66526 0.19553 -0.92323

C 6.19919 -0.31278 0.27415

C 6.64586 0.56644 1.25021

C 5.35361 -2.09946 -1.02951

C 4.67499 -3.26099 -1.38037

C 4.49499 -3.58664 1.02951

C 5.16160 -2.41817 1.38037

C 5.20779 -0.92201 -1.76390

C 4.53142 -0.93857 -2.98238

C 3.96589 -2.13861 -3.40944

C 4.00007 -3.27929 -2.60170

C 3.40238 -4.04907 1.76390

C 3.07854 -3.45504 2.98238

C 3.83504 -2.36525 3.40944

C 4.83998 -1.82452 2.60170

C 3.37047 -5.21227 -0.27415

C 2.83238 -6.03870 -1.25021

C 1.51231 -6.48195 -1.11712

C 0.74318 -6.13734 0.00416

C 1.36431 -5.46923 1.06977

C 2.66329 -5.00403 0.92323

C 4.85738 4.55068 -1.11712

C 4.94351 3.71229 0.00416

C 4.05434 3.91614 1.06977

C 3.00197 4.80849 0.92323

C 2.82872 5.52505 -0.27415

C 3.81347 5.47226 -1.25021

C 0.85862 5.68609 1.02951

C -0.48661 5.67916 1.38037

C -0.85862 5.68609 -1.02951

C 0.48661 5.67916 -1.38037

C 1.80541 4.97108 1.76390

C 1.45289 4.39361 2.98238

C 0.13085 4.50386 3.40944

C -0.83991 5.10380 2.60170

C -1.80541 4.97108 -1.76390

C -1.45289 4.39361 -2.98238

C -0.13085 4.50386 -3.40944

C 0.83991 5.10380 -2.60170

C -2.82872 5.52505 0.27415

C -3.81347 5.47226 1.25021

C -4.85738 4.55068 1.11712

C -4.94351 3.71229 -0.00416

C -4.05434 3.91614 -1.06977

C -3.00197 4.80849 -0.92323

C -1.51231 -6.48195 1.11712

C -0.74318 -6.13734 -0.00416

C -1.36431 -5.46923 -1.06977

C -2.66329 -5.00403 -0.92323

C -3.37047 -5.21227 0.27415

C -2.83238 -6.03870 1.25021

C -4.49499 -3.58664 -1.02951

C -5.16160 -2.41817 -1.38037

C -5.35361 -2.09946 1.02951

C -4.67499 -3.26099 1.38037

C -3.40238 -4.04907 -1.76390

C -3.07854 -3.45504 -2.98238

C -3.83504 -2.36525 -3.40944

C -4.83998 -1.82452 -2.60170

C -5.20779 -0.92201 1.76390

C -4.53142 -0.93857 2.98238

C -3.96589 -2.13861 3.40944

C -4.00007 -3.27929 2.60170

C -6.19919 -0.31278 -0.27415

C -6.64586 0.56644 -1.25021

C -6.36969 1.93127 -1.11712

C -5.68669 2.42506 0.00416

C -5.41865 1.55309 1.06977

C -5.66526 0.19553 0.92323

H 6.62492 2.61053 1.92374

H 4.87946 1.92206 -1.93527

H 7.14591 0.19837 2.13956

H 4.40637 -0.03350 -3.56748

H 3.43313 -2.17438 -4.35393

H 3.45426 -4.16130 -2.90897

H 2.23219 -3.79928 3.56748

H 3.59964 -1.88599 4.35393

H 5.33092 -0.91083 2.90897

H 3.40116 -6.28772 -2.13956

H 1.05168 -7.04261 -1.92374

H 0.77518 -5.18676 1.93527

H 5.57325 4.43209 -1.92374

H 4.10428 3.26470 1.93527

H 3.74475 6.08935 -2.13956

H 2.17418 3.83278 3.56748

H -0.16651 4.06037 4.35393

H -1.87666 5.07213 2.90897

H -2.17418 3.83278 -3.56748

H 0.16651 4.06037 -4.35393

H 1.87666 5.07213 -2.90897

H -3.74475 6.08935 2.13956

H -5.57325 4.43209 1.92374

H -4.10428 3.26470 -1.93527

H -1.05168 -7.04261 1.92374

H -0.77518 -5.18676 -1.93527

H -3.40116 -6.28772 2.13956

H -2.23219 -3.79928 -3.56748

H -3.59964 -1.88599 -4.35393

H -5.33092 -0.91083 -2.90897

H -4.40637 -0.03350 3.56748

H -3.43313 -2.17438 4.35393

H -3.45426 -4.16130 2.90897

H -7.14591 0.19837 -2.13956

H -6.62492 2.61053 -1.92374

H -4.87946 1.92206 1.93527

C 1.44396 6.18198 -0.30685

C 6.07573 -1.84048 0.30685

C 4.63177 -4.34150 -0.30685

C -1.44396 6.18198 0.30685

C -4.63177 -4.34150 0.30685

C -6.07573 -1.84048 -0.30685

C -1.97840 8.31310 -0.66146

H -2.07331 9.35091 -0.34392

H -1.27525 8.25179 -1.49656

H -2.95946 7.94288 -0.98062

C 1.97840 8.31310 0.66146

H 2.07331 9.35091 0.34392

H 1.27525 8.25179 1.49656

H 2.95946 7.94288 0.98062

C 8.18856 -2.44321 -0.66146

H 9.13478 -2.87992 -0.34392

H 7.78388 -3.02150 -1.49656

H 8.35847 -1.40847 -0.98062

C 6.21016 -5.86990 0.66146

H 7.06147 -6.47099 0.34392

H 6.50864 -5.23029 1.49656

H 5.39900 -6.53441 0.98062

C -6.21016 -5.86990 -0.66146

H -7.06147 -6.47099 -0.34392

H -6.50864 -5.23029 -1.49656

H -5.39900 -6.53441 -0.98062

C -8.18856 -2.44321 0.66146

H -9.13478 -2.87992 0.34392

H -7.78388 -3.02150 1.49656

H -8.35847 -1.40847 0.98062

O 1.49186 7.60097 -0.46660

O 5.83670 -5.09248 -0.46660

O -7.32856 -2.50849 -0.46660

O -1.49186 7.60097 0.46660

O 7.32856 -2.50849 0.46660

O -5.83670 -5.09248 0.46660

**XYZ coordinates of** **7:**

**Element X Y Z**

C -1.34556 6.26498 -1.34783

C -0.73794 6.23736 -0.07770

C -1.53789 5.84168 1.01366

C -2.79365 5.30240 0.80178

C -3.28654 5.10408 -0.52055

C -2.59418 5.69210 -1.57882

C -4.33603 4.07624 -0.45828

C -4.50571 3.74312 0.90679

C -5.22813 2.63315 1.42458

C -5.69815 1.71699 0.45828

C -5.49449 2.03050 -0.90679

C -4.89444 3.21112 -1.42458

C -3.63399 4.52251 1.72518

C -3.62352 4.32488 3.08141

C -4.47133 3.32287 3.63475

C -5.22614 2.48727 2.84496

C -5.73360 0.88588 -1.72518

C -5.55722 0.97562 -3.08141

C -5.11336 2.21085 -3.63475

C -4.76711 3.28234 -2.84496

C -6.06353 0.29418 0.52055

C -6.22660 -0.59942 1.57882

C -6.09841 -1.96720 1.34783

C -5.77069 -2.47960 0.07770

C -5.82799 -1.58899 -1.01366

C -5.98883 -0.23183 -0.80178

C 1.34556 6.26498 1.34783

C 0.73794 6.23736 0.07770

C 1.53789 5.84168 -1.01366

C 2.79365 5.30240 -0.80178

C 3.28654 5.10408 0.52055

C 2.59418 5.69210 1.57882

C 4.33603 4.07624 0.45828

C 4.50571 3.74312 -0.90679

C 5.22813 2.63315 -1.42458

C 5.69815 1.71699 -0.45828

C 5.49449 2.03050 0.90679

C 4.89444 3.21112 1.42458

C 3.63399 4.52251 -1.72518

C 3.62352 4.32488 -3.08141

C 4.47133 3.32287 -3.63475

C 5.22614 2.48727 -2.84496

C 5.73360 0.88588 1.72518

C 5.55722 0.97562 3.08141

C 5.11336 2.21085 3.63475

C 4.76711 3.28234 2.84496

C 6.06353 0.29418 -0.52055

C 6.22660 -0.59942 -1.57882

C 6.09841 -1.96720 -1.34783

C 5.77069 -2.47960 -0.07770

C 5.82799 -1.58899 1.01366

C 5.98883 -0.23183 0.80178

C -4.75285 -4.29778 -1.34783

C -5.03275 -3.75776 -0.07770

C -4.29010 -4.25269 1.01366

C -3.19519 -5.07057 0.80178

C -2.77699 -5.39827 -0.52055

C -3.63241 -5.09268 -1.57882

C -1.36212 -5.79323 -0.45828

C -0.98878 -5.77362 0.90679

C 0.33369 -5.84427 1.42458

C 1.36212 -5.79323 0.45828

C 0.98878 -5.77362 -0.90679

C -0.33369 -5.84427 -1.42458

C -2.09961 -5.40838 1.72518

C -1.93369 -5.30050 3.08141

C -0.64203 -5.53372 3.63475

C 0.45903 -5.76961 2.84496

C 2.09961 -5.40838 -1.72518

C 1.93369 -5.30050 -3.08141

C 0.64203 -5.53372 -3.63475

C -0.45903 -5.76961 -2.84496

C 2.77699 -5.39827 0.52055

C 3.63241 -5.09268 1.57882

C 4.75285 -4.29778 1.34783

C 5.03275 -3.75776 0.07770

C 4.29010 -4.25269 -1.01366

C 3.19519 -5.07057 -0.80178

H -0.79288 6.65148 -2.19619

H -1.11163 5.79492 2.00814

H -2.96713 5.64238 -2.59452

H -2.96800 4.89235 3.73406

H -4.48065 3.18467 4.71083

H -5.80982 1.70146 3.30686

H -5.72090 0.12419 -3.73406

H -4.99833 2.28802 -4.71083

H -4.37842 4.18072 -3.30686

H -6.37002 -0.25158 2.59452

H -6.15680 -2.63909 2.19619

H -5.57437 -1.93476 -2.00814

H 0.79288 6.65148 2.19619

H 1.11163 5.79492 -2.00814

H 2.96713 5.64238 2.59452

H 2.96800 4.89235 -3.73406

H 4.48065 3.18467 -4.71083

H 5.80982 1.70146 -3.30686

H 5.72090 0.12419 3.73406

H 4.99833 2.28802 4.71083

H 4.37842 4.18072 3.30686

H 6.37002 -0.25158 -2.59452

H 6.15680 -2.63909 -2.19619

H 5.57437 -1.93476 2.00814

H -5.36391 -4.01240 -2.19619

H -4.46273 -3.86016 2.00814

H -3.40288 -5.39081 -2.59452

H -2.75290 -5.01654 3.73406

H -0.51768 -5.47269 4.71083

H 1.43140 -5.88218 3.30686

H 2.75290 -5.01654 -3.73406

H 0.51768 -5.47269 -4.71083

H -1.43140 -5.88218 -3.30686

H 3.40288 -5.39081 2.59452

H 5.36391 -4.01240 2.19619

H 4.46273 -3.86016 -2.00814

## TDDFT Calculations

**Figure S103:** experimental absorption spectrum and TDDFT calculated (PBE1PBE/6-311G(d), GD3BJ dispersion correction, dichloromethane solvation) absorption spectrum of **7** in dichloromethane.

**Table S1:** Excited state energies, oscillator strengths and orbital contributions for **7** calculated by TDDFT (PBE1PBE/6-311G(d), GD3BJ dispersion correction, dichloromethane solvation).

| No. | Wavelength [nm] | Osc. Strength | Symmetry | Major contribs | Minor contribs |
| --- | --- | --- | --- | --- | --- |
| 1 | 774 | 0,0004 | Singlet-A2 | HOMO→LUMO (96%) | H‑2→L+1 (2%), H-1→L+2 (2%) |
| 2 | 612 | 0,3823 | Singlet-E | HOMO→L+1 (93%) | H-2→LUMO (2%) |
| 3 | 612 | 0,3824 | Singlet-E | HOMO→L+2 (93%) | H-1→LUMO (2%) |
| 4 | 542 | 0,3372 | Singlet-E | H-1→LUMO (95%) | HOMO→L+2 (2%) |
| 5 | 542 | 0,3374 | Singlet-E | H-2→LUMO (95%) | HOMO→L+1 (2%) |
| 6 | 491 | 0 | Singlet-?Sym | H-2→L+2 (44%), H-1→L+1 (44%) | H-6→LUMO (4%), H-4→L+2 (2%), H-3→L+1 (2%) |
| 7 | 487 | 0,0067 | Singlet-?Sym | H-2→L+1 (48%), H-1→L+2 (47%) | HOMO→LUMO (4%) |
| 8 | 475 | 0,0015 | Singlet-?Sym | H-2→L+1 (43%), H-1→L+2 (44%) | H-7→LUMO (3%), H-6→L+2 (2%), H-4→LUMO (2%), HOMO→L+1 (2%) |
| 9 | 475 | 0,0016 | Singlet-?Sym | H-2→L+2 (44%), H-1→L+1 (43%) | H-8→LUMO (3%), H-6→L+1 (2%), H-3→LUMO (2%), HOMO→L+2 (2%) |
| 10 | 462 | 0,0498 | Singlet-?Sym | H-5→L+1 (12%), H-4→LUMO (68%) | H-8→L+2 (2%), H-7→LUMO (3%), H-7→L+1 (2%), H-4→L+1 (4%), H-3→L+2 (4%), HOMO→L+1 (2%) |

**Figure S104:** experimental CD spectrum and TDDFT calculated (PBE1PBE/6-311G(d), GD3BJ dispersion correction, dichloromethane solvation) CD spectrum of **7** in dichloromethane.

## AICD and NICS Calculations

**Figure S105:**  π-only AICD plot (isovalue: 0.02) of **7** calculated at HF/6-311G(d) level of theory.

**Figure S106:** NICS values of **7** calculated at HF/6-311G(d) level of theory.

**Figure S107** ICS surface (isovalue: 5) calculated at HF/6-311G(d) level of theory.

**Figure S108:** NICS contour plot (at *z* = 0) of the benzene (red) and anthracene (blue) subunits of **7** (levels: -8: solid, -6: dashed, -4 dashed dots, -2: dotted). The probe points for the NICS values of ring A and ring D are shown as red dots and the xy-locations of the carbon atoms as black dots. calculated at HF/6-311G(d) level of theory.

## StrainViz Calculations

StrainViz calculations were performed on the optimized structures for all compounds at the B3LYP/6-311G(d) level of theory with GD3BJ dispersion correction. For the platinum atoms the LANL2DZ basis set and ECP were used.^[S82-83]^ For the visualization of the bond strain in all molecules the same color scale from 0.00 to 2.84 kcal/mol was used.

**Figure S109:** StrainViz calculation of **12**. The total Strain energy is 1.44 kcal/mol.

**Figure S110:** StrainViz calculation of **14**. The total Strain energy is 15.78 kcal/mol.

**Figure S111:** StrainViz calculation of **13**. The total Strain energy is 0.62 kcal/mol.

**Figure S112:** StrainViz calculation of **15**. The total Strain energy is 37.67 kcal/mol.

**Figure S113:** StrainViz calculation of **7**. The total Strain energy is 65.99 kcal/mol.

**Figure S114:** StrainViz calculation of **5**. The total Strain energy is 5.46 kcal/mol.

**Figure S115:** StrainViz calculation of **6**. The total Strain energy is 1.84 kcal/mol.

# References

[S1] G. R. Fulmer, A. J. M. Miller, N. H. Sherden, H. E. Gottlieb, A. Nudelman, B. M. Stoltz, J. E. Bercaw, K. I. Goldberg, *Organometallics* **2010**, *29*, 2176-2179.

[S2] L. Krause, R. Herbst-Irmer, G. M. Sheldrick, D. Stalke, *J. Appl. Crystallogr.* **2015**, *48*, 3-10.

[S3] G. M. Sheldrick, *Acta Crystallogr., Sect. A:Found. Adv.* **2015**, *71*, 3-8.

[S4] G. M. Sheldrick, *Acta Crystallogr., Sect. C:Struct. Chem.* **2015**, *71*, 3-8.

[S5] M. J. Frisch, G. W. Trucks, H. B. Schlegel, G. E. Scuseria, M. A. Robb, J. R. Cheeseman, G. Scalmani, V. Barone, G. A. Petersson, H. Nakatsuji, X. Li, M. Caricato, A. V. Marenich, J. Bloino, B. G. Janesko, R. Gomperts, B. Mennucci, H. P. Hratchian, J. V. Ortiz, A. F. Izmaylov, J. L. Sonnenberg, Williams, F. Ding, F. Lipparini, F. Egidi, J. Goings, B. Peng, A. Petrone, T. Henderson, D. Ranasinghe, V. G. Zakrzewski, J. Gao, N. Rega, G. Zheng, W. Liang, M. Hada, M. Ehara, K. Toyota, R. Fukuda, J. Hasegawa, M. Ishida, T. Nakajima, Y. Honda, O. Kitao, H. Nakai, T. Vreven, K. Throssell, J. A. Montgomery Jr., J. E. Peralta, F. Ogliaro, M. J. Bearpark, J. J. Heyd, E. N. Brothers, K. N. Kudin, V. N. Staroverov, T. A. Keith, R. Kobayashi, J. Normand, K. Raghavachari, A. P. Rendell, J. C. Burant, S. S. Iyengar, J. Tomasi, M. Cossi, J. M. Millam, M. Klene, C. Adamo, R. Cammi, J. W. Ochterski, R. L. Martin, K. Morokuma, O. Farkas, J. B. Foresman, D. J. Fox, Gaussian 16 Rev. C.01, Wallingford, CT, **2016**.

[S6] P. Hohenberg, W. Kohn, *Phys. Rev.* **1964**, *136*, B864-B871.

[S7] W. Kohn, L. J. Sham, *Phys. Rev.* **1965**, *140*, A1133-A1138.

[S8] R. G. Parr, W. Yang, *Density-functional theory of atoms and molecules*, 1^st^ ed., Oxford Univ. Press, New York, NY, **1994**.

[S9] W. Koch, *A Chemist's Guide to Density Functional Theory*, 2^nd^ ed., Wiley-VCH, Weinheim, **2001**.

[S10] S. H. Vosko, L. Wilk, M. Nusair, *Can. J. Phys.* **1980**, *58*, 1200–1211.

[S11] C. Lee, W. Yang, R. G. Parr, *Phys. Rev. B: Condens. Matter Mater. Phys.* **1988**, *37*, 785–789.

[S12] A. D. Becke, *J. Chem. Phys.* **1993**, *98*, 5648–5652.

[S13] P. J. Stephens, F. J. Devlin, C. F. Chabalowski, M. J. Frisch, *J. Phys. Chem.* **1994**, *98*, 11623–11627.

[S14] S. Grimme, S. Ehrlich, L. Goerigk, *J. Comput. Chem.* **2011**, *32*, 1456-1465.

[S15] R. Krishnan, J. S. Binkley, R. Seeger, J. A. Pople, *J. Chem. Phys.* **1980**, *72*, 650-654.

[S16] A. D. McLean, G. S. Chandler, *J. Chem. Phys.* **1980**, *72*, 5639-5648.

[S17] M. M. Francl, W. J. Pietro, W. J. Hehre, J. S. Binkley, M. S. Gordon, D. J. DeFrees, J. A. Pople, *J. Chem. Phys.* **1982**, *77*, 3654-3665.

[S18] L. A. Curtiss, M. P. McGrath, J.-P. Blaudeau, N. E. Davis, R. C. Binning, L. Radom, *J. Chem. Phys.* **1995**, *103*, 6104-6113.

[S19] M. N. Glukhovtsev, A. Pross, M. P. McGrath, L. Radom, *J. Chem. Phys.* **1995**, *103*, 1878-1885.

[S20] J.-P. Blaudeau, M. P. McGrath, L. A. Curtiss, L. Radom, *J. Chem. Phys.* **1997**, *107*, 5016-5021.

[S21] J. J. Stewart, *J. Mol. Model.* **2007**, *13*, 1173-1213.

[S22] R. Bauernschmitt, R. Ahlrichs, *Chem. Phys. Lett.* **1996**, *256*, 454–464.

[S23] M. E. Casida, C. Jamorski, K. C. Casida, D. R. Salahub, *J. Chem. Phys.* **1998**, *108*, 4439–4449.

[S24] R. E. Stratmann, G. E. Scuseria, M. J. Frisch, *J. Chem. Phys.* **1998**, *109*, 8218–8224.

[S25] C. van Caillie, R. D. Amos, *Chem. Phys. Lett.* **1999**, *308*, 249–255.

[S26] F. Furche, R. Ahlrichs, *J. Chem. Phys.* **2002**, *117*, 7433–7447.

[S27] G. Scalmani, M. J. Frisch, B. Mennucci, J. Tomasi, R. Cammi, V. Barone, *J. Chem. Phys.* **2006**, *124*, 94107.

[S28] T. Helgaker, P. Jørgensen, *J. Chem. Phys.* **1991**, *95*, 2595–2601.

[S29] K. L. Bak, P. Jørgensen, T. Helgaker, K. Ruud, H. J. r. A. Jensen, *J. Chem. Phys.* **1993**, *98*, 8873–8887.

[S30] K. L. Bak, A. E. Hansen, K. Ruud, T. Helgaker, J. Olsen, P. Jørgensen, *Theor. Chim. Acta* **1995**, *90*, 441–458.

[S31] J. Olsen, K. L. Bak, K. Ruud, T. Helgaker, P. Jørgensen, *Theor. Chim. Acta* **1995**, *90*, 421–439.

[S32] A. E. Hansen, K. L. Bak, *ENANTIOMER* **1999**, 455–476.

[S33] J. Autschbach, T. Ziegler, S. J. A. van Gisbergen, E. J. Baerends, *J. Chem. Phys.* **2002**, *116*, 6930–6940.

[S34] C. Adamo, V. Barone, *J. Chem. Phys.* **1999**, *110*, 6158-6170.

[S35] S. Miertuš, E. Scrocco, J. Tomasi, *Chem. Phys.* **1981**, *55*, 117–129.

[S36] S. Miertuš, J. Tomasi, *Chem. Phys.* **1982**, *65*, 239–245.

[S37] J. L. Pascual-ahuir, E. Silla, I. Tuñon, *J. Comput. Chem.* **1994**, *15*, 1127–1138.

[S38] M. Cossi, V. Barone, R. Cammi, J. Tomasi, *Chem. Phys. Lett.* **1996**, *255*, 327–335.

[S39] V. Barone, M. Cossi, J. Tomasi, *J. Chem. Phys.* **1997**, *107*, 3210–3221.

[S40] E. Cancès, B. Mennucci, J. Tomasi, *J. Chem. Phys.* **1997**, *107*, 3032–3041.

[S41] B. Mennucci, E. Cancès, J. Tomasi, *J. Phys. Chem. B* **1997**, *101*, 10506–10517.

[S42] B. Mennucci, J. Tomasi, *J. Chem. Phys.* **1997**, *106*, 5151–5158.

[S43] V. Barone, M. Cossi, *J. Phys. Chem. A* **1998**, *102*, 1995–2001.

[S44] V. Barone, M. Cossi, J. Tomasi, *J. Comput. Chem.* **1998**, *19*, 404–417.

[S45] M. Cossi, V. Barone, B. Mennucci, J. Tomasi, *Chem. Phys. Lett.* **1998**, *286*, 253–260.

[S46] R. Cammi, B. Mennucci, J. Tomasi, *J. Phys. Chem. A* **1999**, *103*, 9100–9108.

[S47] M. Cossi, V. Barone, M. A. Robb, *J. Chem. Phys.* **1999**, *111*, 5295–5302.

[S48] J. Tomasi, B. Mennucci, E. Cancès, *J. Mol. Struct. THEOCHEM* **1999**, *464*, 211–226.

[S49] R. Cammi, B. Mennucci, J. Tomasi, *J. Phys. Chem. A* **2000**, *104*, 5631–5637.

[S50] M. Cossi, V. Barone, *J. Chem. Phys.* **2000**, *112*, 2427–2435.

[S51] M. Cossi, V. Barone, *J. Chem. Phys.* **2001**, *115*, 4708–4717.

[S52] M. Cossi, N. Rega, G. Scalmani, V. Barone, *J. Chem. Phys.* **2001**, *114*, 5691–5701.

[S53] M. Cossi, G. Scalmani, N. Rega, V. Barone, *J. Chem. Phys.* **2002**, *117*, 43–54.

[S54] M. Cossi, N. Rega, G. Scalmani, V. Barone, *J. Comput. Chem.* **2003**, *24*, 669–681.

[S55] R. Cammi, *J. Chem. Phys.* **2009**, *131*, 164104.

[S56] R. Cammi, *Int. J. Quantum Chem.* **2010**, *110*, 3040–3052.

[S57] F. Lipparini, G. Scalmani, B. Mennucci, E. Cancès, M. Caricato, M. J. Frisch, *J. Chem. Phys.* **2010**, *133*, 014106.

[S58] G. Scalmani, M. J. Frisch, *J. Chem. Phys.* **2010**, *132*, 114110.

[S59] M. Caricato, *J. Chem. Theory Comput.* **2012**, *8*, 4494–4502.

[S60] N. M. O'Boyle, A. L. Tenderholt, K. M. Langner, *J. Comput. Chem.* **2008**, *29*, 839-845.

[S61] J. A. Gaunt, *Math. Proc. Camb. Phil. Soc.* **1928**, *24*, 328–342.

[S62] D. R. Hartree, *Math. Proc. Camb. Phil. Soc.* **1928**, *24*, 111–132.

[S63] J. C. Slater, *Phys. Rev.* **1928**, *32*, 339–348.

[S64] V. Fock, *Z. Phys.* **1930**, *61*, 126–148.

[S65] V. Fock, *Z. Phys.* **1930**, *62*, 795–805.

[S66] J. C. Slater, *Phys. Rev.* **1930**, *35*, 210–211.

[S67] D. R. Hartree, W. Hartree, *Proc. R. Soc. London, A* **1935**, *150*, 9–33.

[S68] C. C. J. Roothaan, *Rev. Mod. Phys.* **1951**, *23*, 69–89.

[S69] F. London, *J. Phys. Radium* **1937**, *8*, 397–409.

[S70] R. McWeeny, *Phys. Rev.* **1962**, *126*, 1028–1034.

[S71] R. Ditchfield, *Mol. Phys.* **1974**, *27*, 789–807.

[S72] K. Wolinski, J. F. Hinton, P. Pulay, *J. Am. Chem. Soc.* **1990**, *112*, 8251–8260.

[S73] J. R. Cheeseman, G. W. Trucks, T. A. Keith, M. J. Frisch, *J. Chem. Phys.* **1996**, *104*, 5497–5509.

[S74] S. Kloda, E. Kleinpeter, *J. Chem. Soc., Perkin Trans. 2* **2001**, 1893-1898.

[S75] T. Lu, F. Chen, *Journal of Computational Chemistry* **2012**, *33*, 580-592.

[S76] T. A. Keith, R. F. W. Bader, *Chem. Phys. Lett.* **1992**, *194*, 1–8.

[S77] T. A. Keith, R. F. W. Bader, *Chem. Phys. Lett.* **1993**, *210*, 223–231.

[S78] T. Lu, Q. Chen, *Theor. Chem. Acc.* **2020**, *139*.

[S79] R. Herges, D. Geuenich, *J. Phys. Chem. A* **2001**, *105*, 3214–3220.

[S80] D. Geuenich, K. Hess, F. Köhler, R. Herges, *Chem. Rev.* **2005**, *105*, 3758–3772.

[S81] C. E. Colwell, T. W. Price, T. Stauch, R. Jasti, *Chem. Sci.* **2020**, *11*, 3923-3930.

[S82] P. J. Hay, W. R. Wadt, *J. Chem. Phys.* **1985**, *82*, 299-310.

[S83] P. J. Hay, W. R. Wadt, *J. Chem. Phys.* **1985**, *82*, 270-283.

[S84] D. Hellwinkel, *Die systematische Nomenklatur der organischen Chemie*, 5^th^ ed., **2006**.
